# Synthesis and Applications of Carbohydrate-Based Organocatalysts

**DOI:** 10.3390/molecules26237291

**Published:** 2021-11-30

**Authors:** Elżbieta Wojaczyńska, Franz Steppeler, Dominika Iwan, Marie-Christine Scherrmann, Alberto Marra

**Affiliations:** 1 Faculty of Chemistry, Wrocław University of Science and Technology, Wybrzeże Wyspiańskiego 27, 50 370 Wrocław, Poland; elzbieta.wojaczynska@pwr.edu.pl (E.W.); franz.steppeler@pwr.edu.pl (F.S.); dominika.iwan@pwr.edu.pl (D.I.); 2 Institut de Chimie Moléculaire et des Matériaux d’Orsay (ICMMO), Université Paris-Saclay, Bâtiment 420, 91405 Orsay, France; marie-christine.scherrmann@universite-paris-saclay.fr; 3 Institut des Biomolécules Max Mousseron (IBMM-UMR 5247), Université de Montpellier, Pôle Chimie Balard Recherche, 1919 Route de Mende, 34293 Montpellier, France

**Keywords:** proline, cinchona alkaloids, thioureas, chitosan, phase-transfer catalysis, aldol reaction, Michael addition, Strecker reaction, Diels–Alder reaction, Darzens condensation

## Abstract

Organocatalysis is a very useful tool for the asymmetric synthesis of biologically or pharmacologically active compounds because it avoids the use of noxious metals, which are difficult to eliminate from the target products. Moreover, in many cases, the organocatalysed reactions can be performed in benign solvents and do not require anhydrous conditions. It is well-known that most of the above-mentioned reactions are promoted by a simple aminoacid, l-proline, or, to a lesser extent, by the more complex cinchona alkaloids. However, during the past three decades, other enantiopure natural compounds, the carbohydrates, have been employed as organocatalysts. In the present exhaustive review, the detailed preparation of all the sugar-based organocatalysts as well as their catalytic properties are described.

## 1. Introduction

For decades, the asymmetric syntheses have been conducted using metal complexes with chiral organic ligands: the titanium-catalysed epoxidation of alkenes and the osmium-catalysed dihydroxylation of alkenes being two of the most known examples of this approach [1]. However, despite the important role of metal catalysis in synthesis, it cannot be ignored that these methods require the use of noxious metals, which can contaminate the target organic compound (often a drug candidate), or are not orthogonal with many functional groups, or need to operate under strictly anhydrous and/or anaerobic conditions.

In order to address the above-mentioned issues, organocatalysis has been widely exploited since the beginning of the present century [2,3,4], and its profound impact on the asymmetric synthesis has been emphasized by the Nobel prize in Chemistry 2021, which has been awarded jointly to B. List and D. W. C. MacMillan. During the last twenty years, a considerable number of organocatalysed reactions have been developed, most of them performed in the presence of proline derivatives or cinchona alkaloids.

Nevertheless, non-functionalised natural carbohydrates (e.g., d-glucosamine and chitosan) and synthetic sugar derivatives (e.g., sugar thioureas, sugar ketones, sugar prolinamides and sugar crown ethers) have also been employed as organocatalysts in various enantioselective transformations, such as aldol reaction, epoxidation, the Mannich reaction, the Michael addition and the aza-Henry reaction. It is worth noting that carbohydrates are particularly well suited as a starting material for new organocatalysts because they are highly functionalized, enantiopure compounds that are easily available in monomeric, dimeric, and polymeric forms featuring larger chemical diversity than amino acids and other natural molecules.

A thorough literature survey revealed that more than two hundred articles dealing with synthetic applications of carbohydrate-based organocatalysts have been published over the last two decades; however, this body of work has been only partially highlighted in reviews. In particular, four reviews [5,6,7,8] were devoted to a single class of sugar-derived organocatalysts, i.e., the carbohydrate-containing crown ethers, while five survey articles on the asymmetric epoxidation of olefins described more [9,10] or less [11,12,13] extensively the properties of another family of carbohydrates, the sugar ketones.

Two reviews [14,15] were focused on carbohydrate-based ligands and chiral auxiliaries, while the use of sugars as organocatalysts was only reported in a very short section at the end of the paper. A book’s chapter published in 2013 [16] reviewed the synthesis and applications of a very limited number of sugar-based organocatalysts. Another short review [17] covered the synthetic applications of various families of sugar organocatalysts without describing the preparation of the latter. A recent, short review [18] was almost exclusively devoted to sugars as phosphorous, sulphur and nitrogen ligands, and the sugar-based organocatalysts were only mentioned in a one-page section at the end of the article.

On the other hand, the larger review [19] published the same year in the same journal, although fully focused on carbohydrate organocatalysis, described the applications of only a class of sugar-based organocatalysts, i.e., the aminosugars and their derivatives; however, the syntheses of these organocatalysts were not reported. Another 2016 review [20] described exclusively the application of sugar-based organocatalysts in aldol reaction without mention of other organocatalysed reactions. In a 2017 review [21], a one-page section was devoted to only polysaccharide-based organocatalysis.

Finally, three reviews [22,23,24] were completely focused on chitosan-based organocatalysts, whereas a more extensive 2020 review [25] described, in addition to the synthetic applications of chitosan, also those of other natural polysaccharide-based organocatalysts. In the present review, all the articles published to date (a few of them were published in the early 1990s) are critically described, including the detailed synthesis of each sugar-based organocatalyst as well as its chemical and stereochemical outcome.

## 2. Sugar Ureas and Thioureas

In their pioneering work, Jacobsen and co-workers showed that chiral urea or thiourea catalysts containing Schiff base can be used in asymmetric cyanation reaction of aldimines and ketimines [26,27]. A variety of bifunctional catalysts based on thioureas of chiral diamines have been developed by several groups, e.g., those led by Takemoto [28] and Nagasawa [29]. These organocatalysts are able to catalyse Michael, Mannich, aza-Henry, Morita–Baylis–Hillman, Strecker and many other reactions, thus, providing a variety of useful chiral building blocks.

The use of cheap, easily available and configurationally stable carbohydrates as the additional source of chirality in these assemblies was introduced in 2007 [30], and since then many modifications have been described, extending their applications in asymmetric transformations. Most of these compounds were obtained from a tetra-*O*-acetyl-d-glucopyranose unit bearing an isothiocyanate at the anomeric position that was reacted with amines to afford thioureas.

Less frequently, urea derivatives are used, typically prepared by a reaction of sugar azides reduced in situ with phosphine, CO_2_ and amine. In general, the proposed models explaining the stereoselectivity in the catalytic reaction involve interactions of reactants with (thio)urea and amine parts of the catalyst, the carbohydrate moiety being only responsible for providing the steric hindrance or a general chiral environment.

**Scheme 1:**
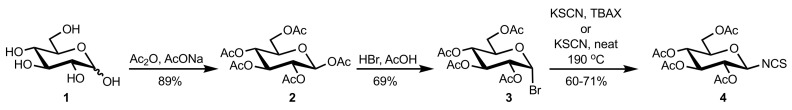
The preparation of d-glucopyranosyl isothiocyanate.

Anomeric sugar isothiocyanates were prepared from the corresponding glycosyl bromides by nucleophilic substitution with thiocyanate ion (Scheme 1). The starting carbohydrate can be acetylated with acetic anhydride in pyridine at room temperature to give a mixture of anomers (93% for d-mannose [31]) or by heating at 100 °C with acetic anhydride in the presence of sodium acetate (d-mannose 99%, d-lactose 95%, d-galactose 47%, [32]; and d-glucose 89% [33]). The bromination was performed by treatment of the sugar acetate with phosphorus bromide in the presence of water (six equiv.) to give the axial glycosyl bromide as a single anomer (85% yield for the mannose derivative [31]). A lower yield (69%) was reported [33] for the bromination of peracetylated glucose using a solution of HBr in acetic acid as described by Deniaud and co-workers [34].

Efficient and selective methods for the preparation of carbohydrate-based isothiocyanates were introduced by several groups. In 1984, de las Heras and co-workers described the reaction of sugar halides with potassium thiocyanate in acetonitrile in the presence of tetraalkylammonium salt (hydrogen sulfate, iodide or bromide) and 4Å molecular sieves [35]. In case of acylated substrates, the products were obtained as single stereoisomers with 1,2-*trans* configuration (α-d anomer for mannose, β-d for glucose, in 72% and 71% yields, respectively).

The use of tetrabutylammonium bromide under similar conditions allowed the synthesis of other derivatives, including disaccharide (cellobiose and lactose) isothiocyanates as reported by Deniaud and co-workers [34]. An interesting solvent-free modification was introduced by Lindhorst and Kieburg: peracetylated glycosyl bromides were melted with KSCN [36]. This fast (10 min) protocol led to various sugar isothiocyanates, including disaccharides (lactose, maltose and cellobiose) as pure anomers with a 41–74% yield (with an exception of galactose, which gave a 1:9 α/β mixture).

### 2.1. Michael Addition

Chiral amine-thiourea catalysts containing sugar moieties have been frequently applied in asymmetric Michael additions. The products, optically active nitroalkanes, can be readily transformed into synthetically valuable compounds, like nitrile oxides, amines, ketones and carboxylic acids for agricultural and pharmaceutical applications.

**Scheme 2:**
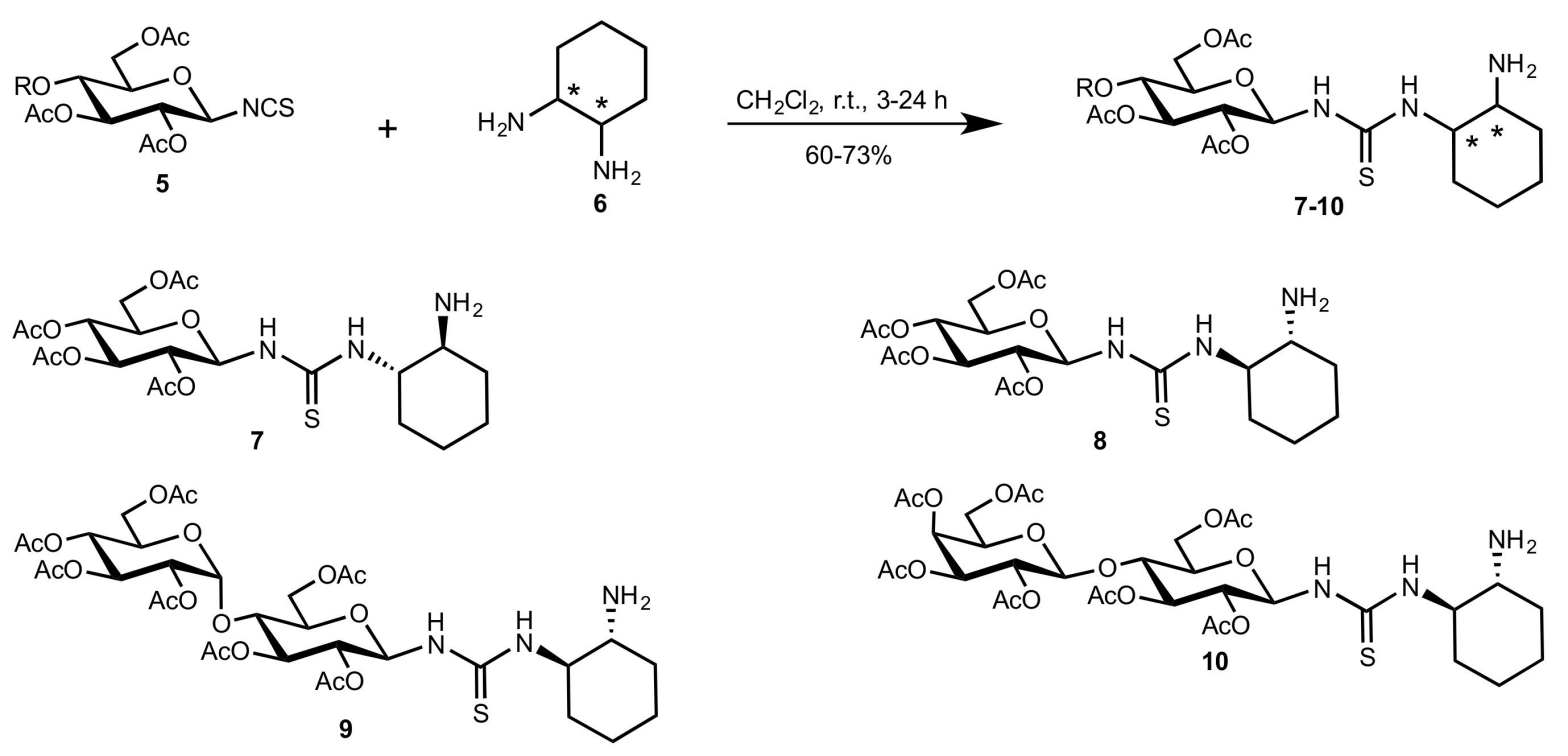
The synthesis of organocatalysts **7–10** (the asterisks indicate stereogenic carbon atoms).

The organocatalysts **7–10** were prepared [30] by the addition of (1*R*,2*R*)- or (1*S*,2*S*)-1,2-diaminocyclohexane (DACH) to glycosyl isothiocyanates (Scheme 2).

**Scheme 3:**
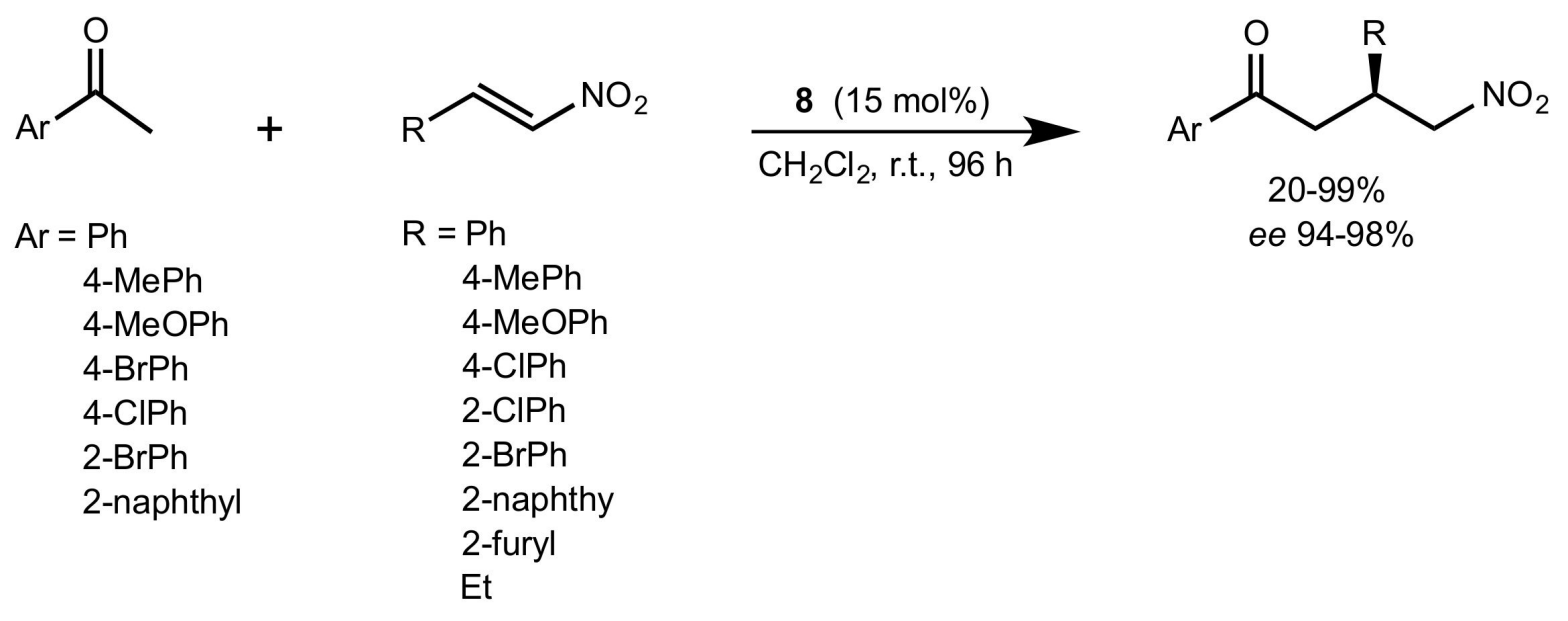
The Michael addition catalysed by **8**.

The conjugate addition of aromatic ketones to aromatic, heteroaromatic and aliphatic nitroolefins was performed in the presence of 15 mol% of catalysts (Scheme 3). Matching of (*R*,*R*)-configuration of 1,2-diaminocyclohexane with β-d-glucopyranose (catalyst **8**) enhanced the stereochemical control (enantiomeric excess up to 98%).

**Figure 1:**
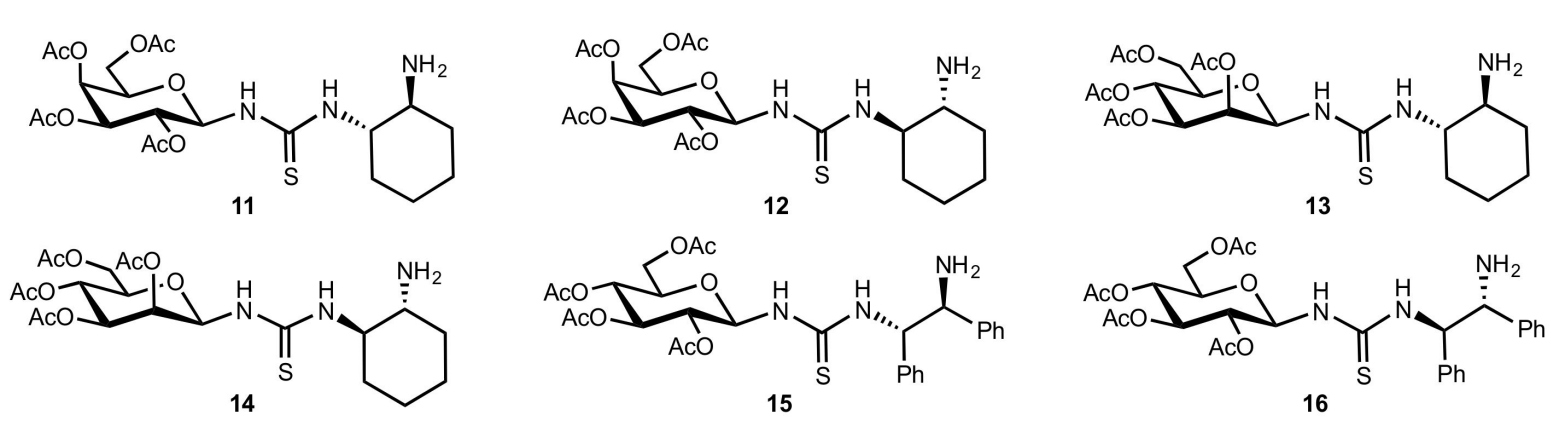
Sugar thiourea organocatalysts employed by Wu and co-workers.

The use of acetone in an asymmetric Michael addition with nitroolefins still remains a challenge for the organic chemists. To this end, Wu and co-workers employed the d-*gluco* configured catalysts **7**, **8** (see Scheme 2) as well as **15**, **16** bearing 1,2-diphenylethane-1,2-diamine (DPEN), the d-*galacto* thioureas **11**, **12** and the d-*manno* thioureas **13**, **14** (Figure 1) [37].

**Scheme 4:**
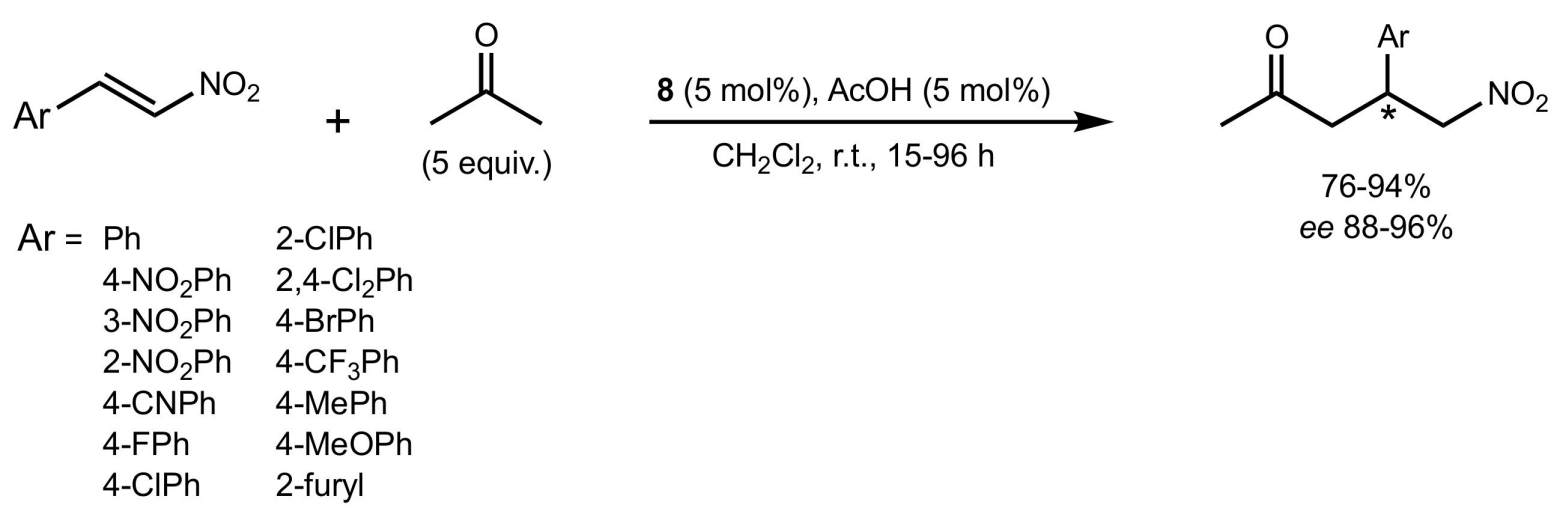
The Michael addition of acetone to nitrostyrenes catalysed by **8**.

Again, the product configuration was dependent on the stereochemistry of the diamine part, and a match between this moiety and the appended sugars on the reactivity and enantioselectivity of the Michael reaction was noted. The addition of acetic acid (5 mol%) was found to be essential for the good reactivity and stereochemical outcome. After preliminary screening of the different organocatalysts, the best results were obtained using 5 mol% of the sugar thiourea **8** in CH_2_Cl_2_ at room temperature (Scheme 4).

**Figure 2:**
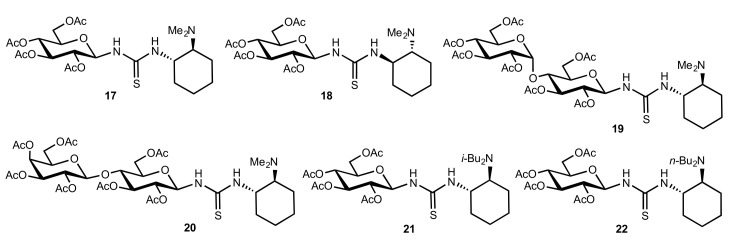
The structure of organocatalysts **17–22**.

An optimization of the addition of dimethyl malonates to various nitroalkenes catalysed by the sugar-thioureas **17–22** (Figure 2) was performed by the Ma’s group [38]. The catalysts were prepared from glycosyl isothiocyanates and *N*,*N*-disubstituted 1,2-diaminocyclohexane.

**Scheme 5:**
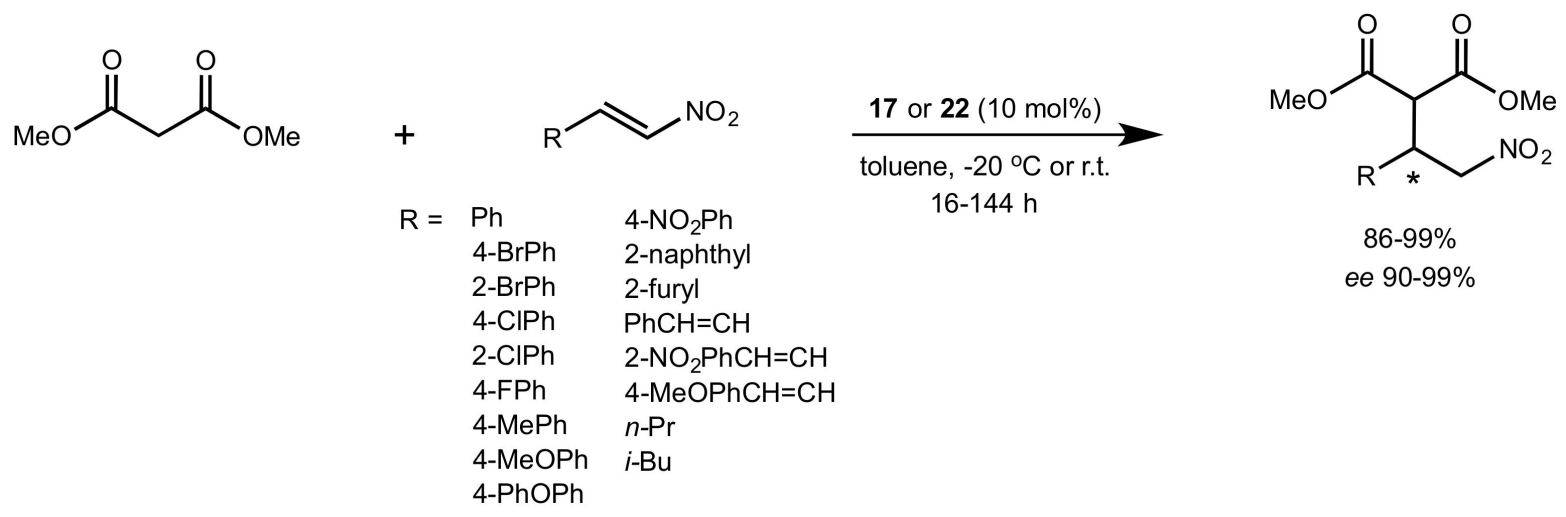
The Michael addition of methyl malonate to nitrostyrenes catalysed by **17** or **22**.

The best result (99% yield and 99% *ee*) was observed for the catalyst **22** containing the di-*n*-butyl (1*S*,2*S*)-1,2-diaminocyclohexane and β-d-glucopyranose moieties, using 10 mol% organocatalyst load and toluene as the solvent and performing the reaction at room temperature (Scheme 5). However, the time required to complete the reaction was long. On the other hand, similar yield and stereoselectivity but faster rates were observed when the catalyst **17** was used at −20 °C.

**Figure 3:**
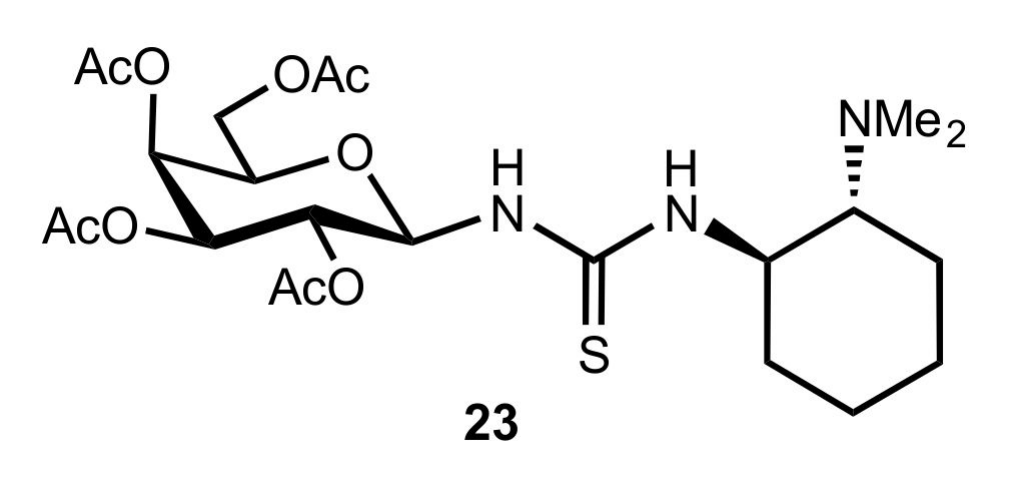
Galactose-based organocatalyst **23**.

The sugar thioureas **18**, **20** (see Figure 2) and **23** (Figure 3) were used in the Michael addition of acetylacetone with various nitroolefins [39]. (*R*,*R*)-*N*,*N*-Dimethyl-cyclohexane-1,2-diamine was synthesised from (*R*,*R*)-1,2-diaminocyclohexane by monoamine protection with phthalic anhydride and *N*,*N*-dimethylation, followed by deprotection.

**Scheme 6:**
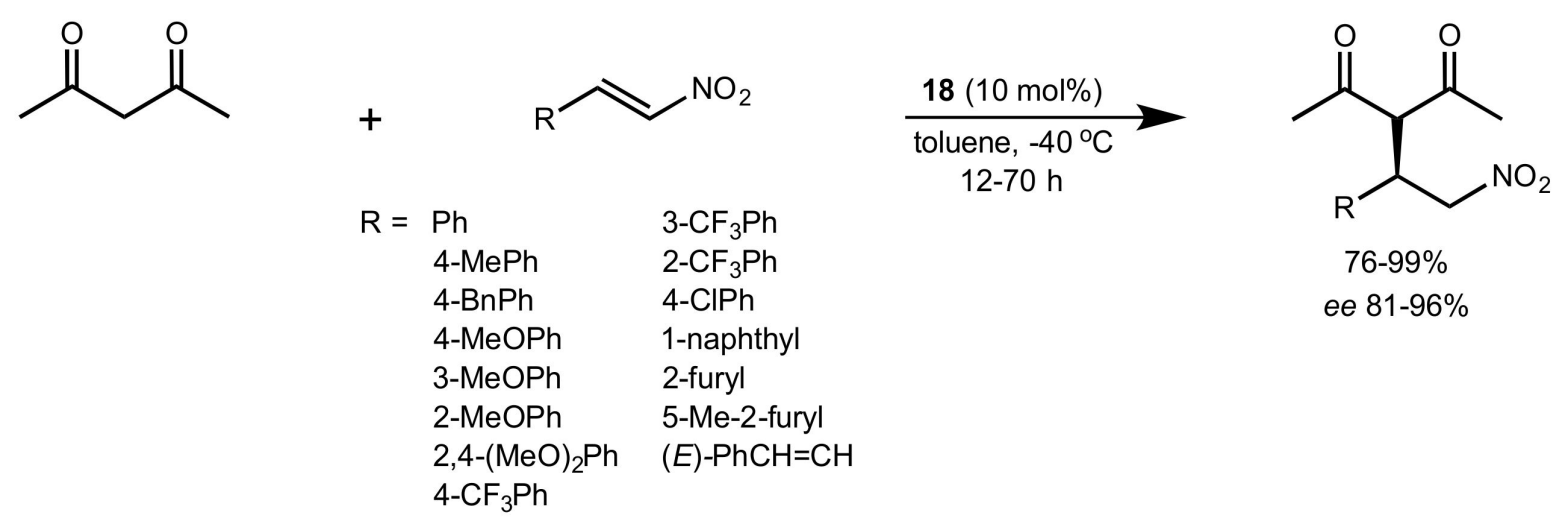
The Michael addition of acetylacetone to nitroolefins catalysed by **18**.

The three organocatalysts (10 mol%) were able to promote enantioselective addition at very low temperature, but the best results were found with **18**, which afforded excellent stereochemical outcomes, with up to 96% *ee* (Scheme 6). However, the reaction did not proceed neither for nitro-substituted aryl nitroolefin nor for the alkyl derivative.

**Scheme 7:**
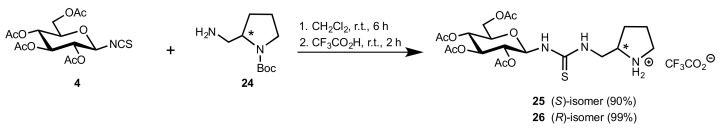
The synthesis of organocatalysts **25** and **26**.

An application of the bifunctional pyrrolidine-based glucosyl thioureas **25** and **26** (Scheme 7) in a highly stereoselective Michael addition of cyclohexanone to various nitroolefins was reported by Zhou’s group [40].

**Scheme 8:**
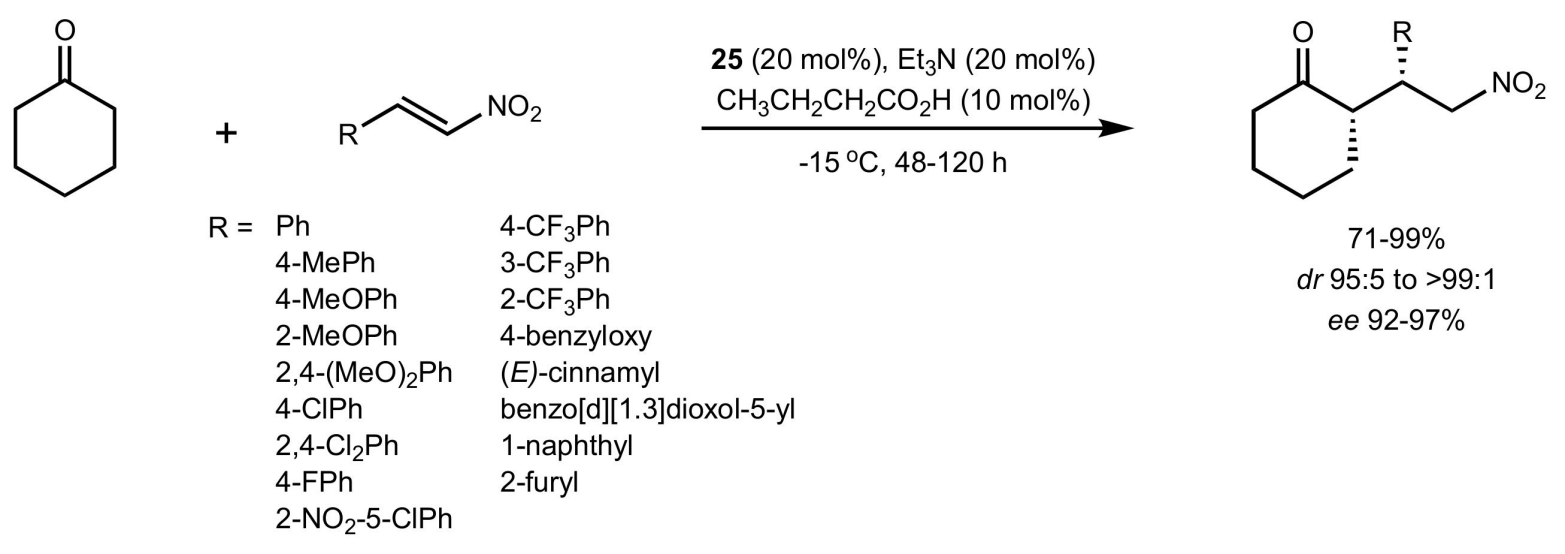
The Michael addition of cyclohexanone to nitroolefins catalysed by **25**.

Synthetically useful γ-nitroketones were obtained with excellent diastereo- (up to >99:1 *syn/anti* ratio) and enantioselectivity (up to 97% *ee*) under optimized conditions (Scheme 8). The use of organocatalyst **26** resulted in lower conversion after a prolonged time, albeit the stereoselectivity was high, and the opposite enantiomer of the adduct was obtained. As free bases were prone to decomposition, the catalysts were stored as trifluoroacetate salts and activated in situ by the addition of triethylamine.

**Figure 4:**
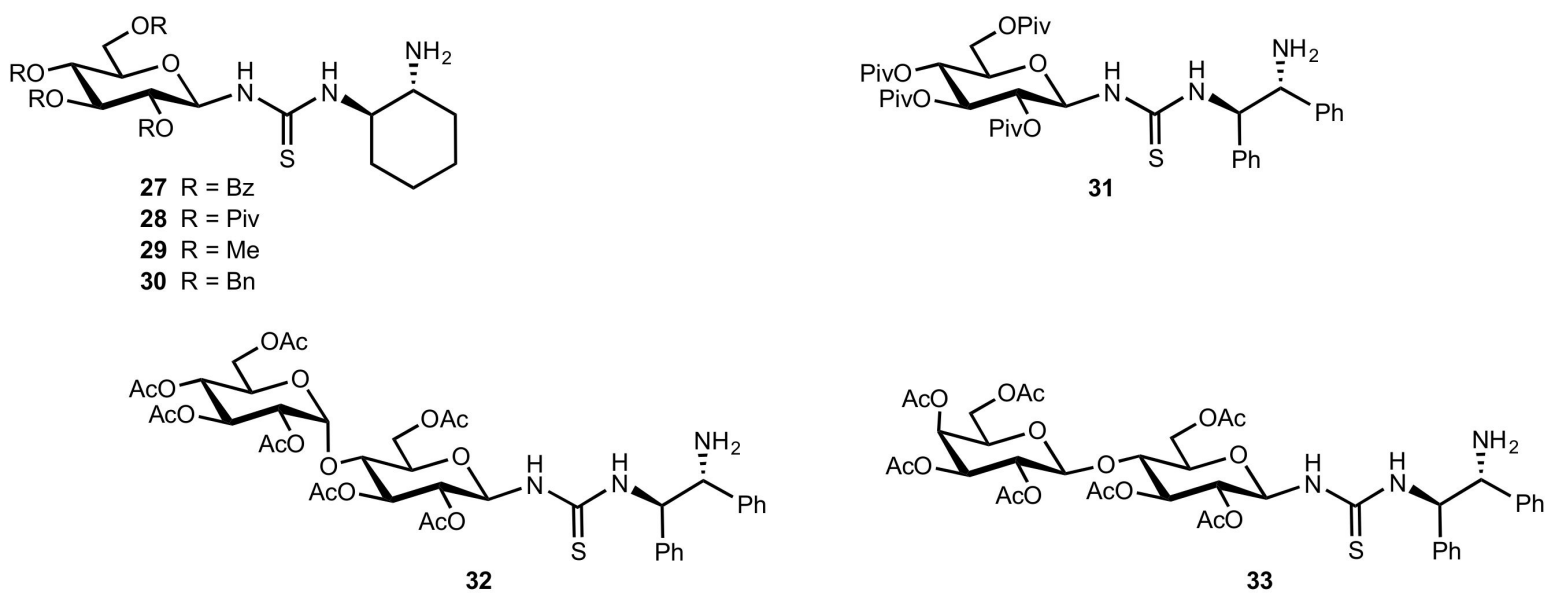
Bifunctional organocatalysts used in the Michael addition of ketones to nitrodienes.

In the enantioselective conjugate addition of ketones to nitrodienes, Ma and co-workers employed the sugar thioureas **8**, **15**, **16**, and **27–33** (Figure 4) prepared from chiral diamines and variously *O*-protected mono- and disaccharides [41].

**Scheme 9:**
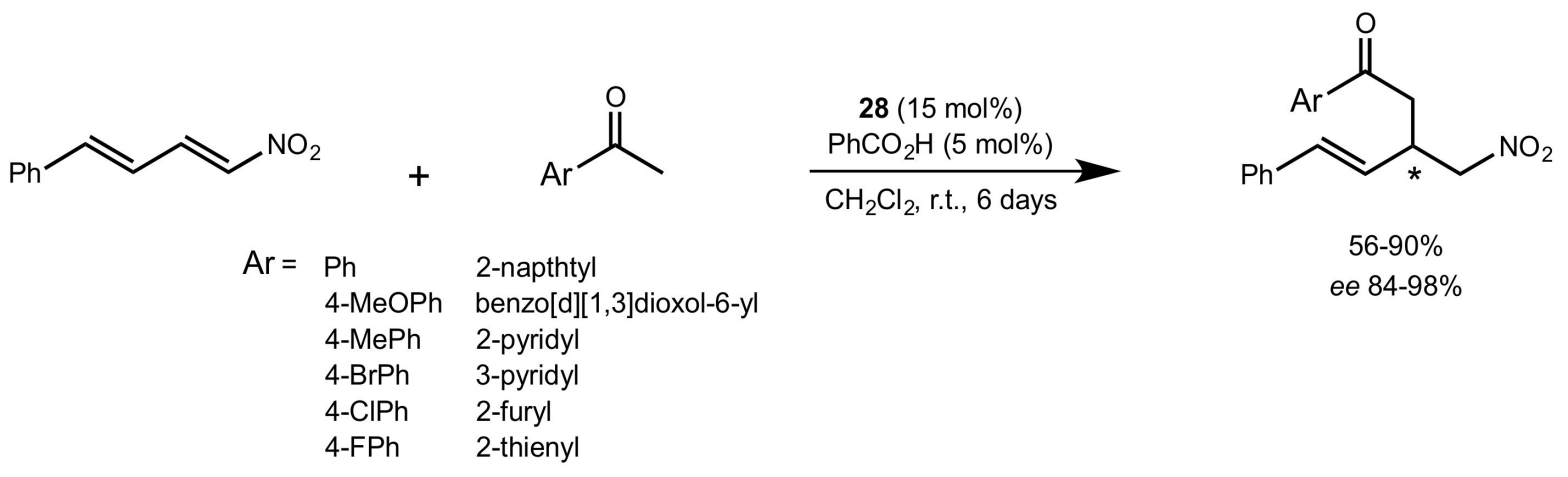
The Michael addition of aryl methyl ketones to nitrodienes catalysed by **28**.

Only the products of 1,4 addition were observed, and they were formed in moderate to high yield (48–98%) and high enantioselectivity (84–99% *ee*). The reaction was conducted with 15 mol% of the catalyst (**28** was found the most versatile) in dichloromethane at room temperature for 6 days, in the presence of 5 mol% of benzoic acid (Scheme 9 shows the addition of aryl methyl ketones). Transformation of the obtained unsaturated nitroketones into 5-substituted 3-pyrrolidinecarboxylic acids was also described.

**Scheme 10:**
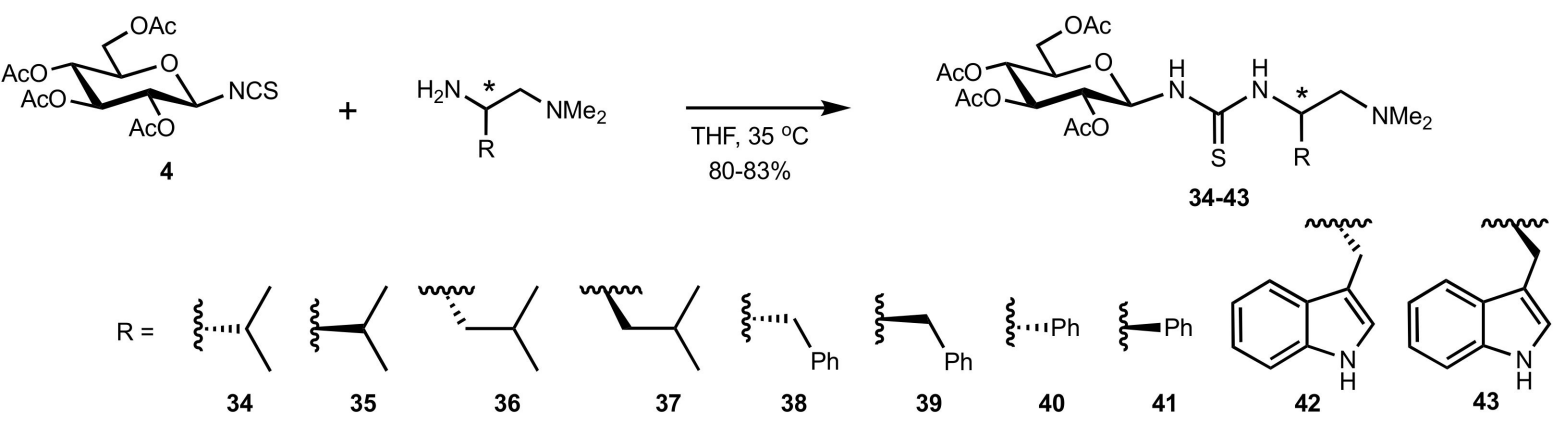
Preparation of catalysts containing carbohydrate and aminoacid moieties.

In a quest for efficient catalysts of Michael reactions, saccharide-derived thioureas were also combined with other chiral moieties. A study of the conjugate addition of acetylacetone to various nitroolefins was performed by Shao and co-workers [42,43]. They used fine-tunable organocatalysts **34–43,** which were synthesised by coupling primary-tertiary diamine obtained from inexpensive l-aminoacids and glucosyl isothiocyanate (Scheme 10).

**Scheme 11:**
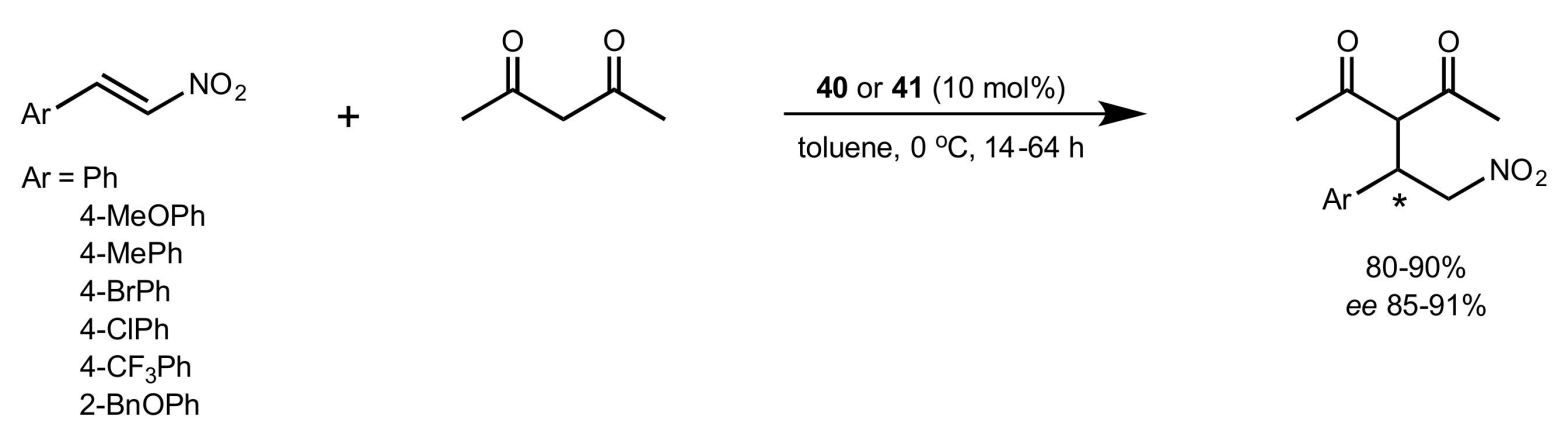
The Michael addition of acetylacetone to nitrostyrenes catalysed by **40** or **41**.

The matching of l-configured valine and d-sugar resulted in enantiomeric excess enhancing (up to 91%). Moreover, the aminoacid fragment was found to determine the stereochemical outcome of the process (Scheme 11). What is worth underlining, the use of “mismatched” organocatalysts and changing toluene to THF solvent gave the opposite enantiomeric adduct with the same *ee*.

**Figure 5:**
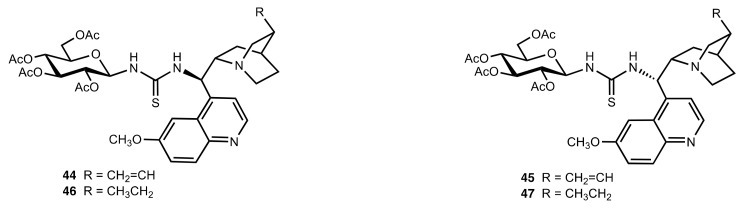
Sugar thioureas containing quinine, quinidine, dihydroquinine and dihydroquinidine.

Reddy and co-workers performed an evaluation of cytotoxicity of the Michael adducts obtained in the highly enantioselective reaction of 5-hydroxy-2-(hydroxymethyl)-4H-pyran-4-one (kojic acid) derivatives with β-nitroolefins [44]. Cinchona alkaloid-derived sugar thioureas **44–47** (Figure 5), prepared in the usual way, were tested as catalysts of the process. Unfortunately, the analytical and spectral data of compounds **45–47** were not reported in the article [44].

**Scheme 12:**
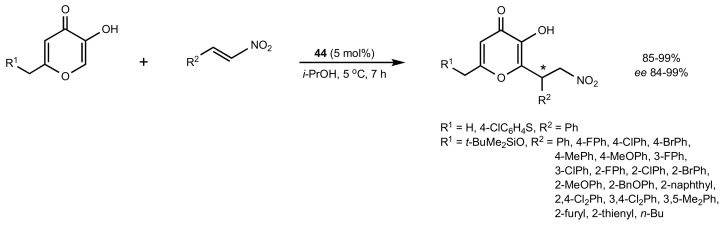
The Michael addition of kojic acid derivatives to nitroalkenes.

Preliminary experiments allowed establishing that **44** gave better results compared to the three other organocatalysts. In isopropanol as the solvent, the catalyst **44** (5 mol%) led, after 7 h at 5 °C, to the (*R*)-configured adducts in excellent yield (85–99%) and enantioselectivity (85–99% *ee*) starting from a variety of nitrostyrenes and even a *n*-butyl derivative (Scheme 12).

**Scheme 13:**
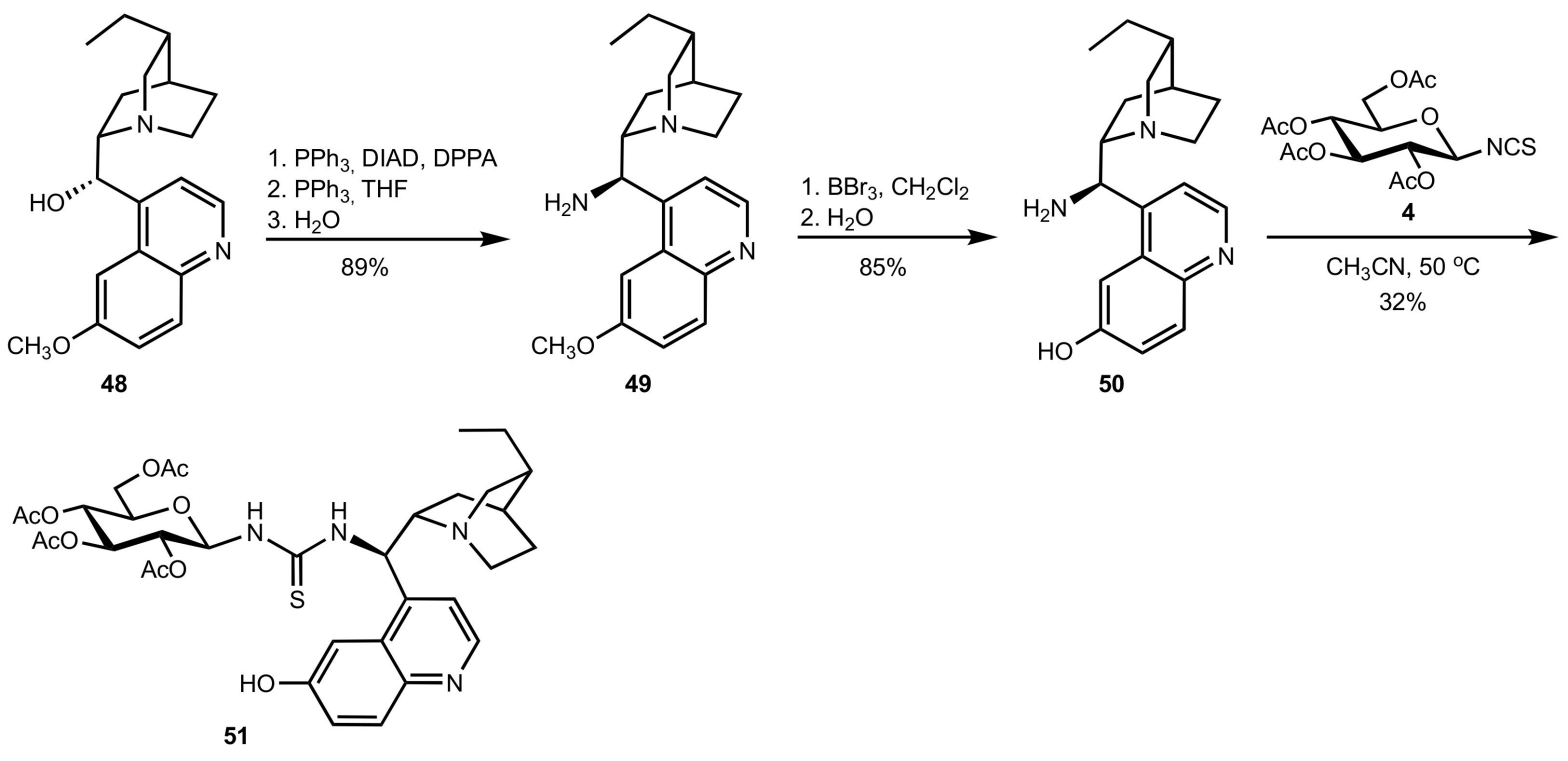
The synthesis of dihydroquinine-derived organocatalyst **51**.

The glucosyl thiourea organocatalyst **51** containing a cinchona alkaloid unit (Scheme 13) was also tested in the Michael reaction of pentane-2,4-dione to β-nitrostyrene [33]. The addition product was obtained in 40–42% yield and 59–64% *ee*, depending on the solvent used.

**Figure 6:**
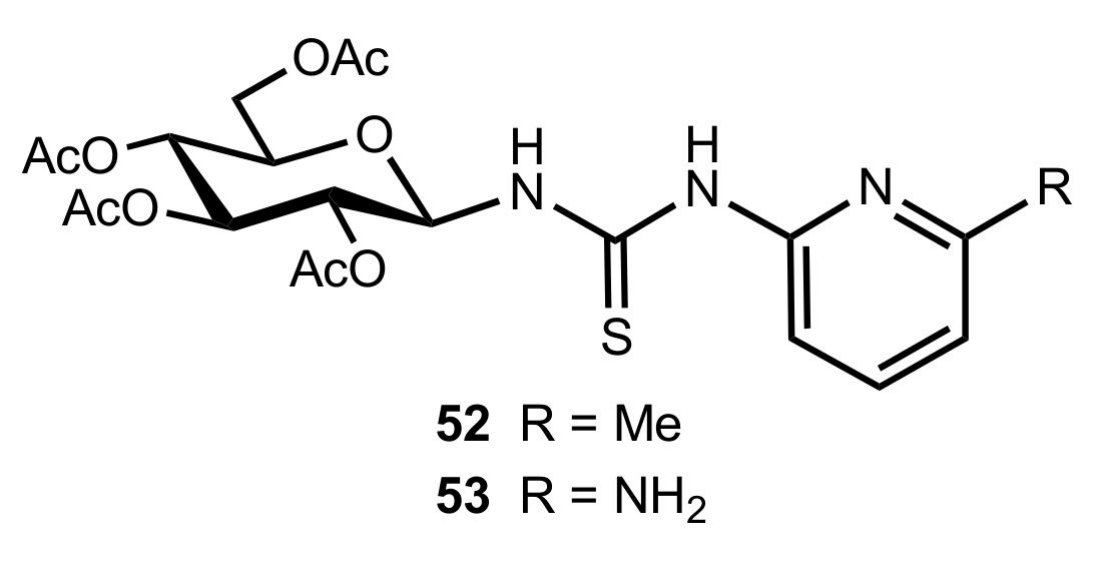
The structure of organocatalysts **52** and **53**.

Interestingly, the pyridine analogues **52** and **53** (Figure 6), easily prepared by reaction of **4** with 2-amino-6-methyl-pyridine or 2,6-diamino-pyridine, respectively, led to the racemic product in low yield.

**Scheme 14:**
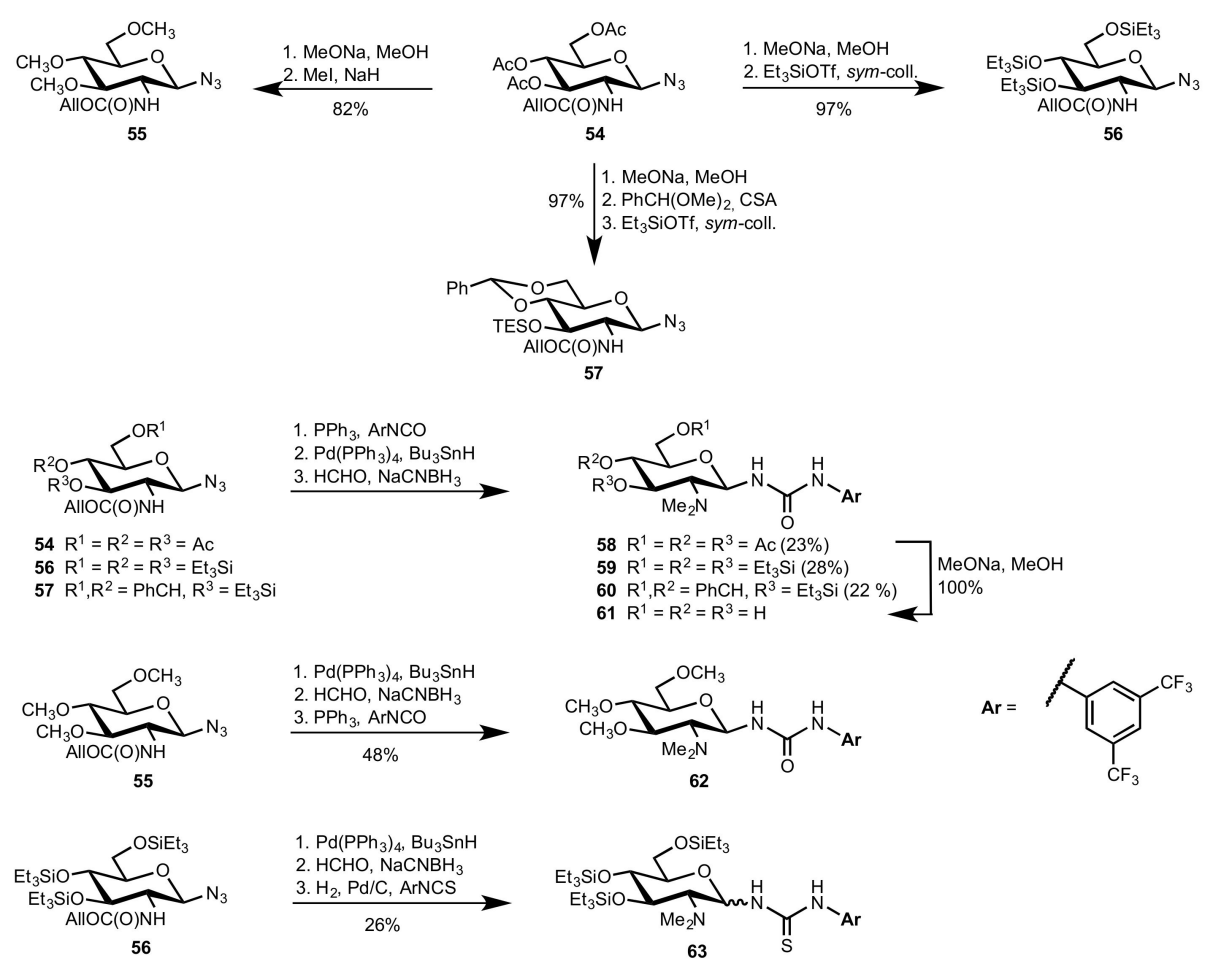
The synthesis of organocatalysts **58–63** (CSA = 10-camphorsulfonic acid; *sym*-coll. = *sym*-collidine, 2,4,6-trimethylpyridine).

Novel bifunctional organocatalysts **58–62** bearing a tertiary amino group and a urea moiety were prepared by Benaglia, Lay, and their co-workers (Scheme 14) [45]. They were all obtained from a common starting material, the known glycosyl azide **54** (for its synthesis from commercially available d-glucosamine hydrochloride see Scheme 117 in Section 5.8). This compound was first deacetylated and then methylated, silylated or converted into the 4,6-*O*-benzylidene derivative and then silylated to give **55**, **56** or **57**, respectively. The azide group of **54**, **56** and **57** was transformed into the amine function, and the latter reacted with bis-trifluoromethyl-phenylisocyanate.

The allyloxycarbonyl group of the resulting urea derivatives was removed with palladium(0)-tetrakis(triphenylphosphine) and tributyltin hydride to give the free amine group that was dimethylated by reductive amination to afford **58–60**. Standard deacetylation by transesterification of **58** led to the triol urea derivative **61**. In order to prepare the tri-*O*-methyl-sugar urea organocatalyst, the glycosyl azide **55** was first *N,N*-dimethylated and then converted into the anomeric urea derivative **62**. One thiourea organocatalyst was also prepared from **56;** however, partial epimerization of the transient amine occurred, and therefore **63** was obtained as a mixture of anomers.

**Scheme 15:**
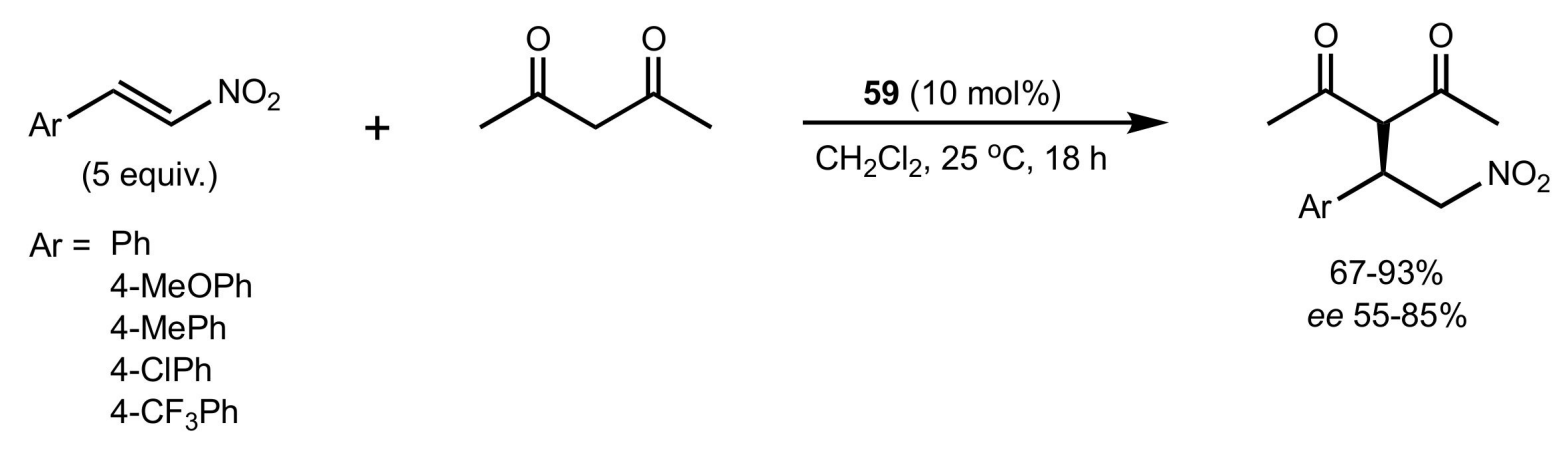
The Michael addition catalysed by **59**.

Application of the obtained derivatives in a reaction between acetylacetone and *trans*-β-nitrostyrene revealed persilylated catalyst **59** as the best candidate to optimization procedure; it was found that the chemical yield could be improved by the application of an excess of nitrostyrene (5 equiv.) and dichloromethane as solvent. Five different nitroolefins gave the corresponding adducts in good yield (up to 93%) and enantiomeric excess (up to 85%) (Scheme 15).

**Figure 7:**
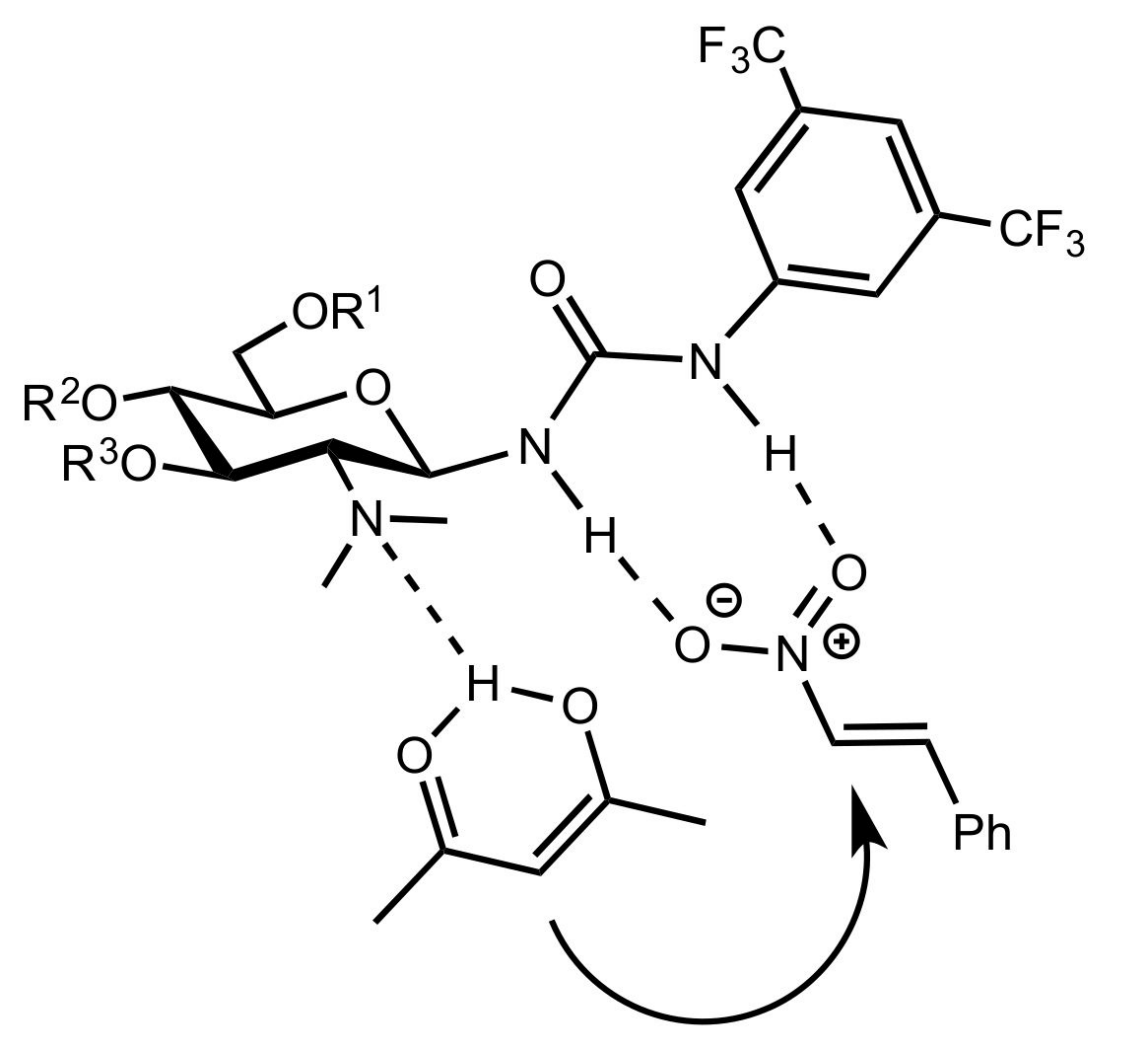
A proposed transition state explaining the stereoselectivity of the reaction.

Figure 7 shows the proposed interactions that account for the observed stereoselectivity, which was further substantiated by semiempirical (AM1) calculations.

**Scheme 16:**
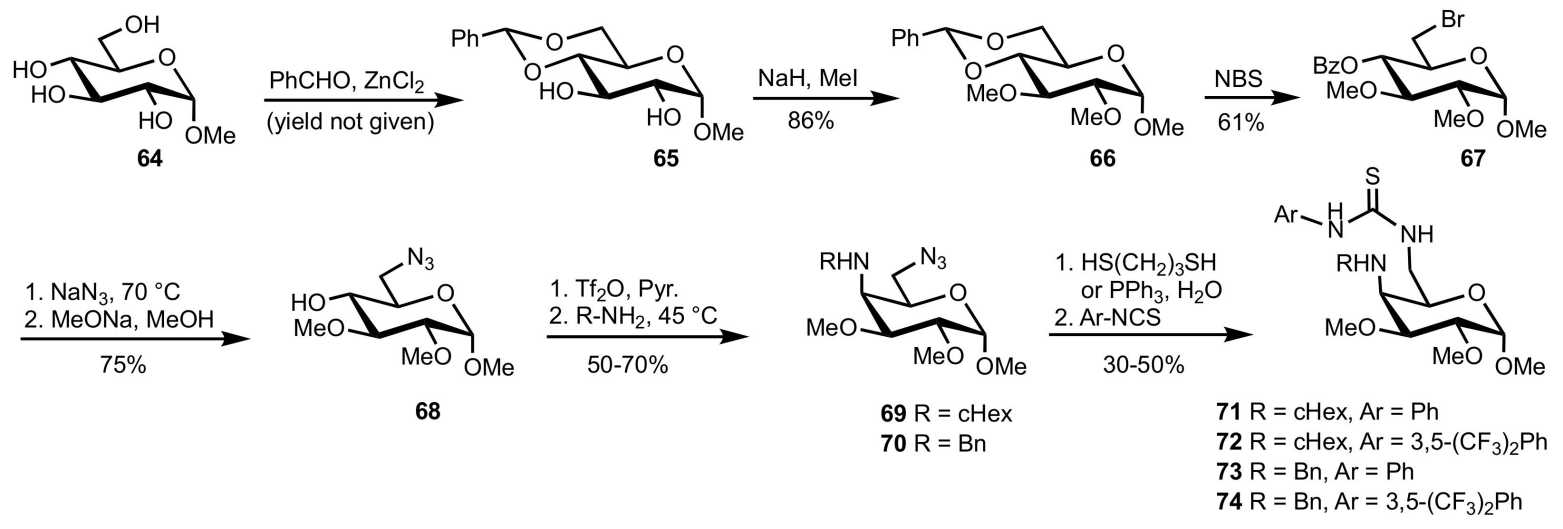
The synthesis of organocatalysts **71–74**.

Another class of bifunctional organocatalysts in which H-bond donor and Lewis base functionalities were combined in a one chiral molecular scaffold were tested in the Michael addition of acetylacetone to β-nitrostyrene [46]. The synthesis of 4-amino-6-thioureidosugar organocatalysts **71–74** (Scheme 16) started from the bromide derivative **67**, easily prepared from commercial methyl α-d-glucopyranoside as described [47].

The 6-bromo-glucopyranoside **67** was azidated and transformed into the 4-amino-6-azido-galactopyranoside **69** and **70** by removal of the benzoyl group, activation of the resulting alcohol as triflate and reaction with cyclohexyl- or benzylamine. Reduction of the azide function to amine and treatment with aryl isothiocyanates gave the sugar thioureas **71–74**. The activity of these organocatalysts was somewhat low, with the best yield being 70% and the *ee* 19%. It is worth noting that the catalysts bearing active groups in other positions (6-amino-4-thioureido- or 2-amino-3-thioureido-sugars) were found to be inactive in the Michael addition.

**Scheme 17:**
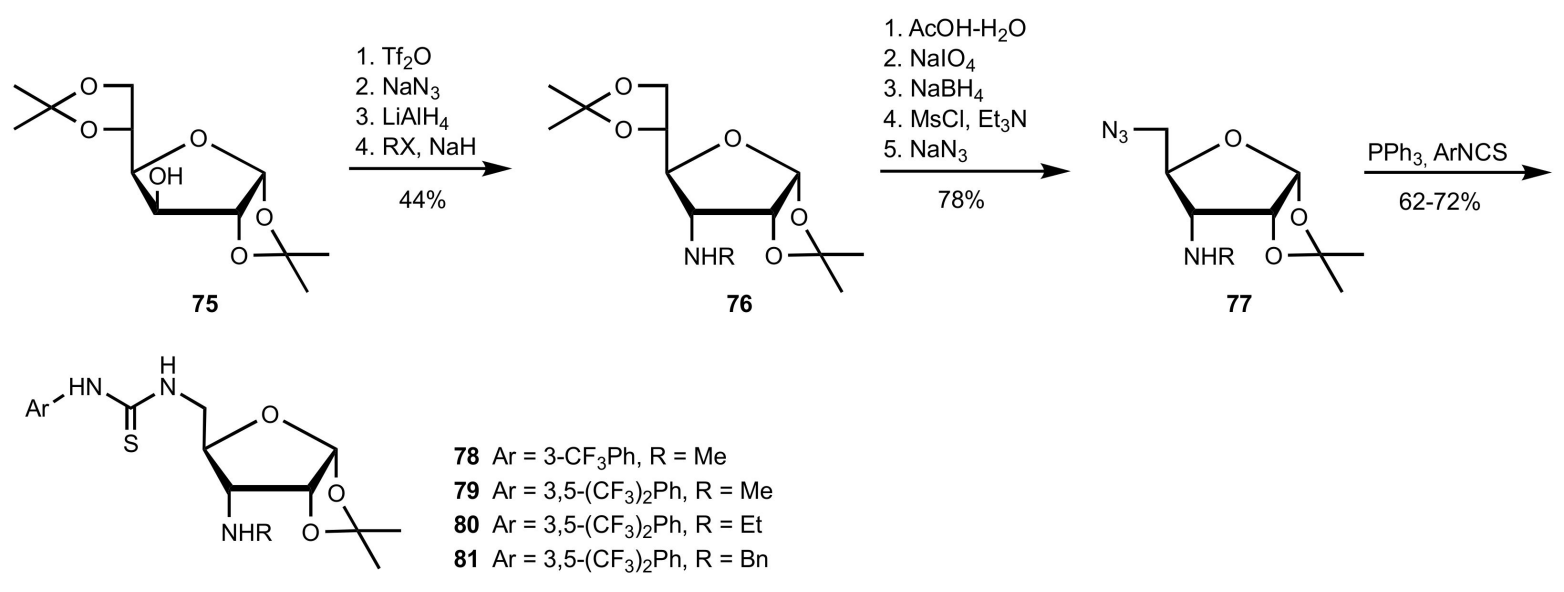
The synthesis of the organocatalysts **78–81**.

**Figure 8:**
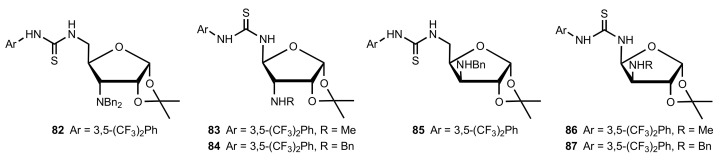
The structure of organocatalysts **82–87**.

Bifunctional thiourea organocatalysts **78–81** (Scheme 17) containing d-glucose diacetonide equipped with secondary amine groups were synthesised [48] from d-glucose diacetonide **75** (in the article erroneously drawn as an l-sugar). The sugar thioureas **82–87** (Figure 8) were prepared in a similar way from **75**. Unfortunately, the analytical and spectral data of all the precursors of **78–87** were missing.

**Scheme 18:**
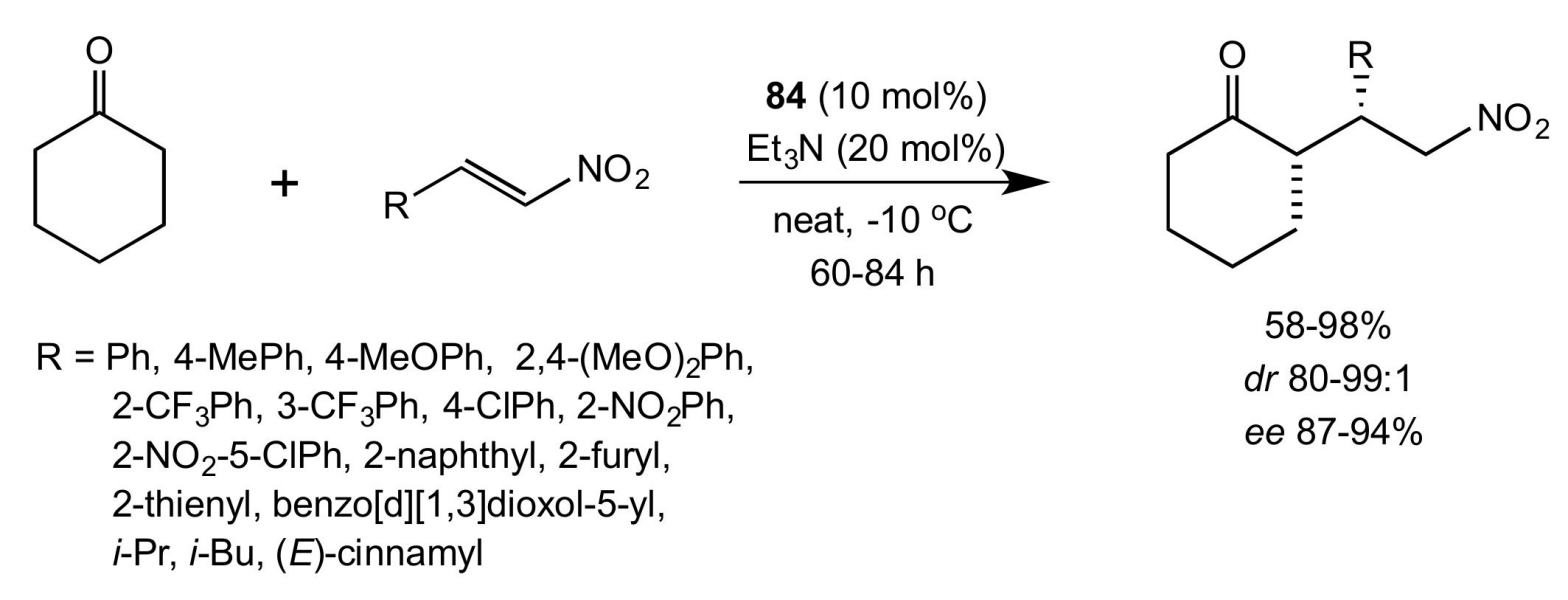
The Michael addition catalysed by **84**.

The Michael addition of cyclohexanone to aryl or alkyl nitroalkenes was performed with 10 mol% of the catalyst that gave the best preliminary results, i.e., **84**, and 20 mol% of triethylamine in the absence of solvent at −10 °C, for at least 60 h. The conversion of various *trans*-β-nitrostyrenes was generally high (85–98% yield), but for aliphatic substrates yield dropped down to ca. 60% (Scheme 18). Stereoselectivity was in most cases excellent (up to 99:1 *dr* and 95% *ee*). Cyclohexanone could be also replaced with other Michael donors, e.g., ketones and esters bearing electron-withdrawing groups. The outcome was supported by density functional theory (DFT) calculations, and the role of cyclohexanone serving both as solvent and reactant in the key step was shown.

**Scheme 19:**
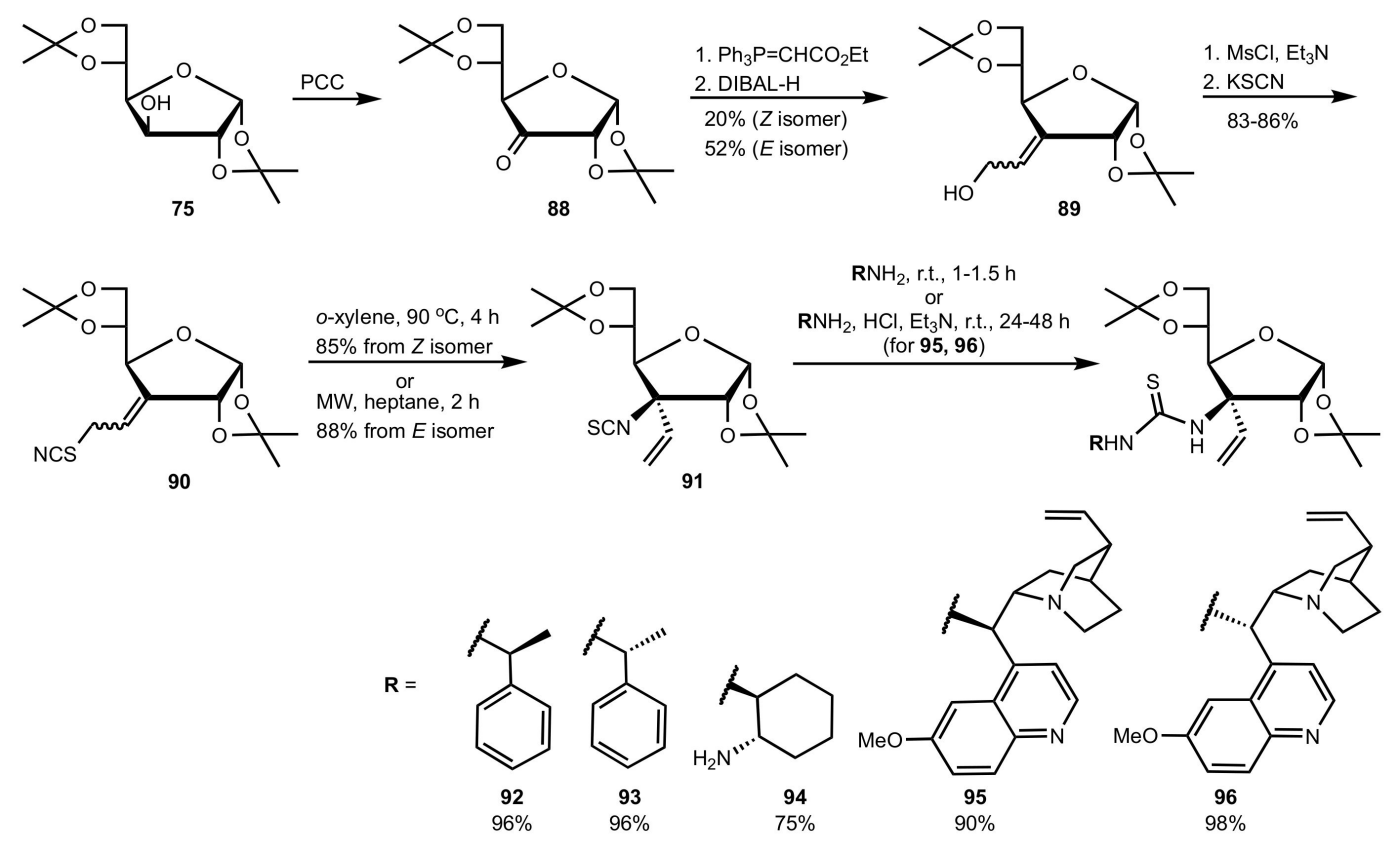
Preparation of catalysts **92–96**.

In 2020, the novel catalysts **92–96** (Scheme 19) based on a glucofuranose skeleton bearing a thiourea group at C-3 and various chiral amines were reported by Tvrdoňová and co-workers [49]. Their preparation made use of isothiocyanate **91**, which was synthesised as previously reported by them [50] starting from either *E* or *Z* isomer of allylic alcohol **89** (separated by column chromatography). The latter was obtained by oxidation (yield not given) of **75** with pyridinium chlorochromate (PCC) followed by Wittig olefination with [(ethoxycarbonyl)-methylene]triphenylphosphorane, and subsequent reduction with DIBAL-H as described [51,52,53]. The alcohol **89** was mesylated and reacted with potassium thiocyanate to give **90** that was submitted to thermal [3,3]-sigmatropic rearrangement to afford the isothiocyanate derivative **91**. Reaction of the latter with various primary amines led to **92–96**.

The sugar thioureas were employed to catalyse the Michael addition of acetylacetone to (*E*)-nitrostyrene. Preliminary screening showed that quinine-derived catalyst **95** (20 mol%) gave the optimal results in CH_2_Cl_2_ after 24 h at room temperature (up to 98% yield, and 88% *ee*, with (*R*)-adduct predominating) [49]. The enantioselectivity was maintained when the catalyst load was decreased to 5 mol%**,** though the yield was lower (87%).

**Scheme 20:**
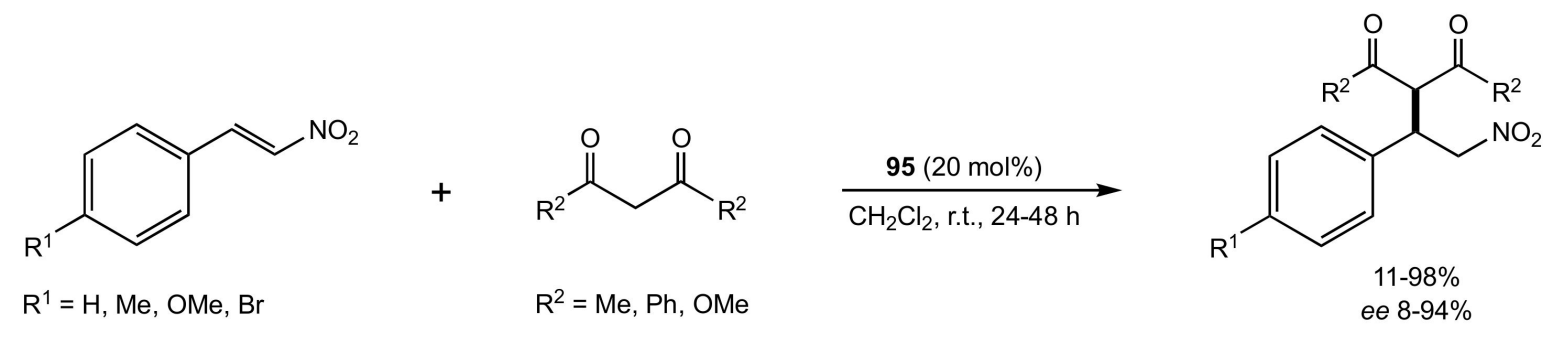
The Michael addition catalysed by **95**.

A series of nitrostyrenes were reacted with diketones in the presence of **95** to afford the corresponding adducts in high yield (73–98%) and *ee* (76–94%); however, the use of dimethyl malonate led to poor results (11–42% yield, 8–17% *ee*) (Scheme 20). When compound **95** was replaced by its epimer **96**, the (*S*)-configured adducts were formed, albeit in lower yield (31–85%) and enantiomeric excess (16–60%). The authors did not comment on the stability of their organocatalysts bearing a terminal double bond.

**Figure 9:**
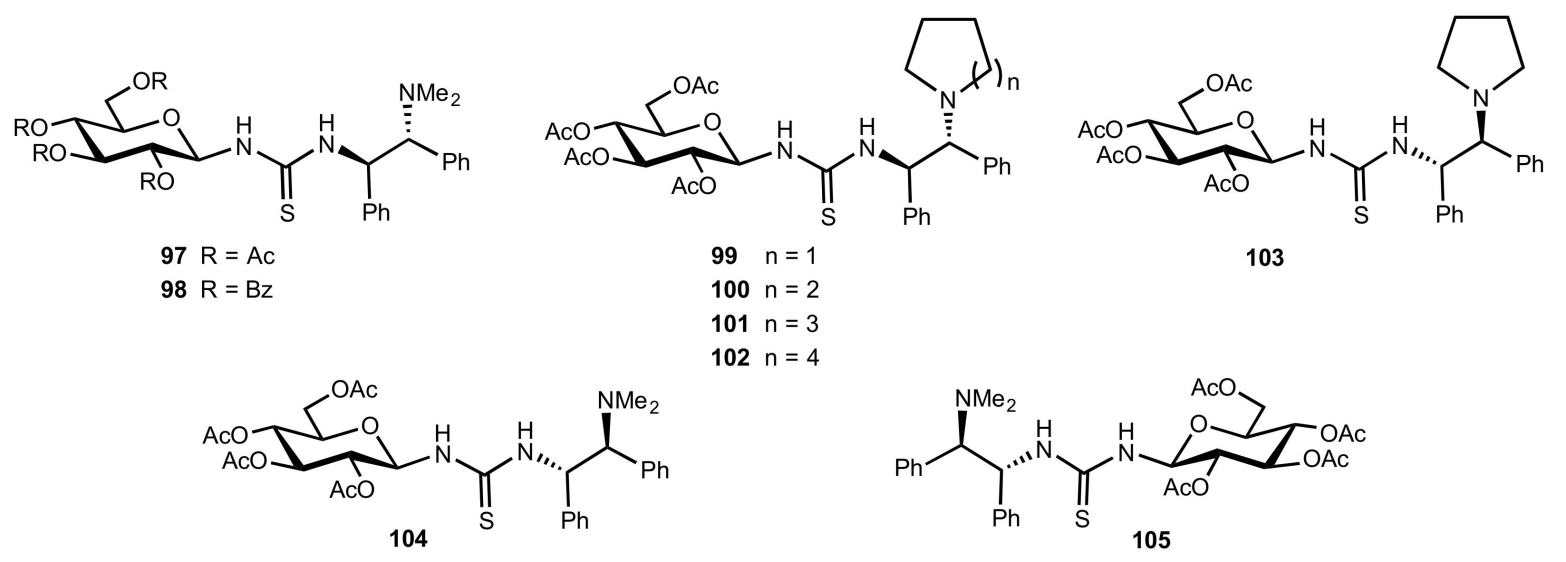
The structure of organocatalysts **97–105**.

Chiral fluorinated heterocycles have a great potential for application as components of biologically active compounds. The preparation of a series of fluorinated pyrazolone and isoxazolone derivatives described by Ma and co-workers was based on the one-pot sequential conjugate addition/dearomative fluorination reaction of 3-methyl-1-phenylpyrazolone [54] or 3-phenylisoxazol-5(4*H*)-one [55] with *trans*-β-nitrostyrene and *N*-fluorobenzenesulfonimide. Bifunctional glucose-based chiral tertiary amino-thiourea catalysts **17**, **18**, **21**, **22**, and **97–105** (Figure 9) were found to be efficient in the model reaction.

**Scheme 21:**
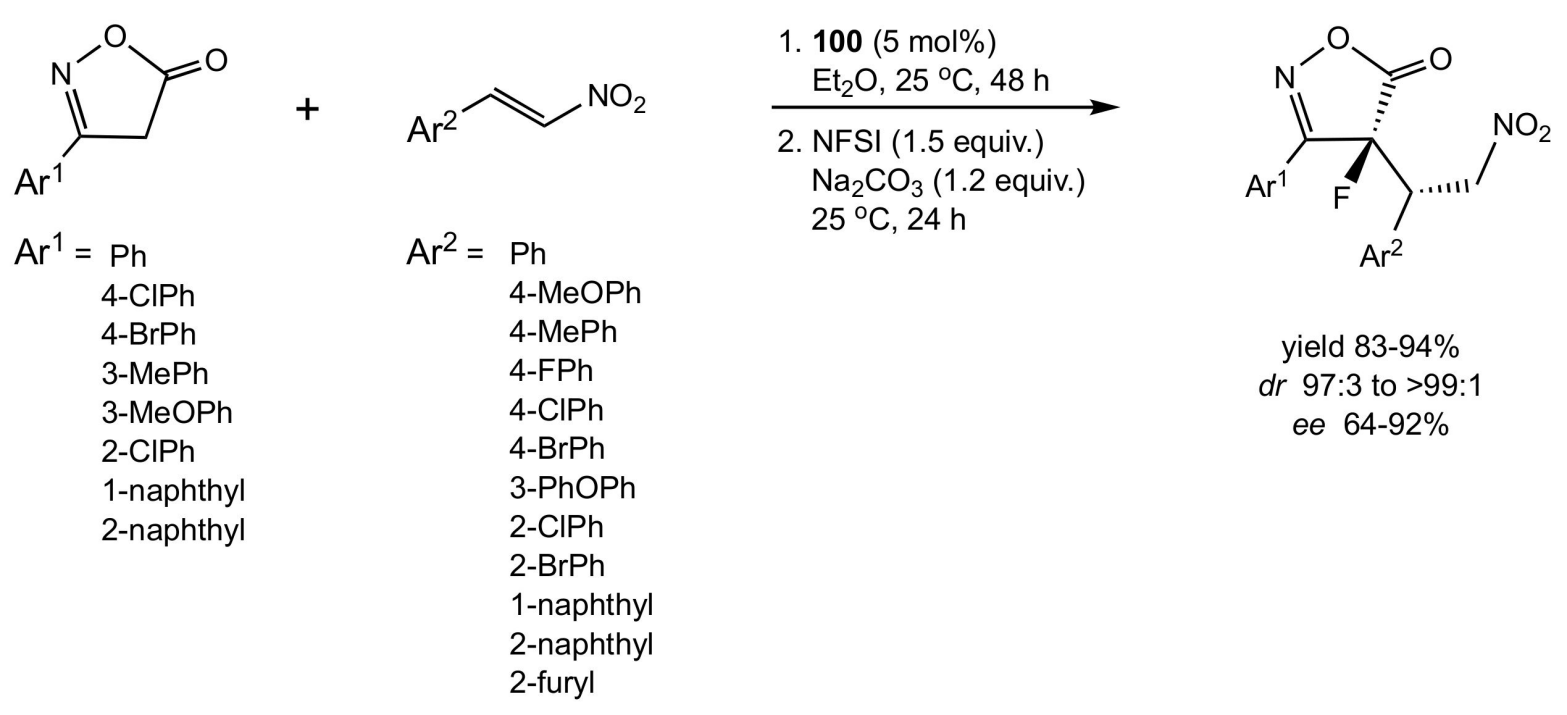
The synthesis of chiral fluorinated isoxazol-5(4*H*)-ones catalysed by **100** (NFSI = *N*-fluorobenzenesulfonimide).

In case of pyrazolone derivatives, catalyst **97** led to the best outcomes as, for the reaction conducted in toluene with benzoic acid additive, a 72–95% yield, up to >99:1 *dr* and 98% *ee* were found for 21 compounds [54]. The use of **105** (optical antipode of **104**), not surprisingly, resulted in a reversed asymmetric induction. In case of isoxazolones, toluene or diethyl ether were used as solvents affording the adducts in 80–93% yield and *dr* better than 97:3 [55]. The enantioselectivity was variable, with the maximum *ee* value of 86% for catalyst **100** containing a piperidine ring. Using this catalyst, nineteen fluorinated isoxazolones were prepared in 83–94% yield, in most cases as single diastereomers with 64–92% *ee* (Scheme 21).

**Figure 10:**
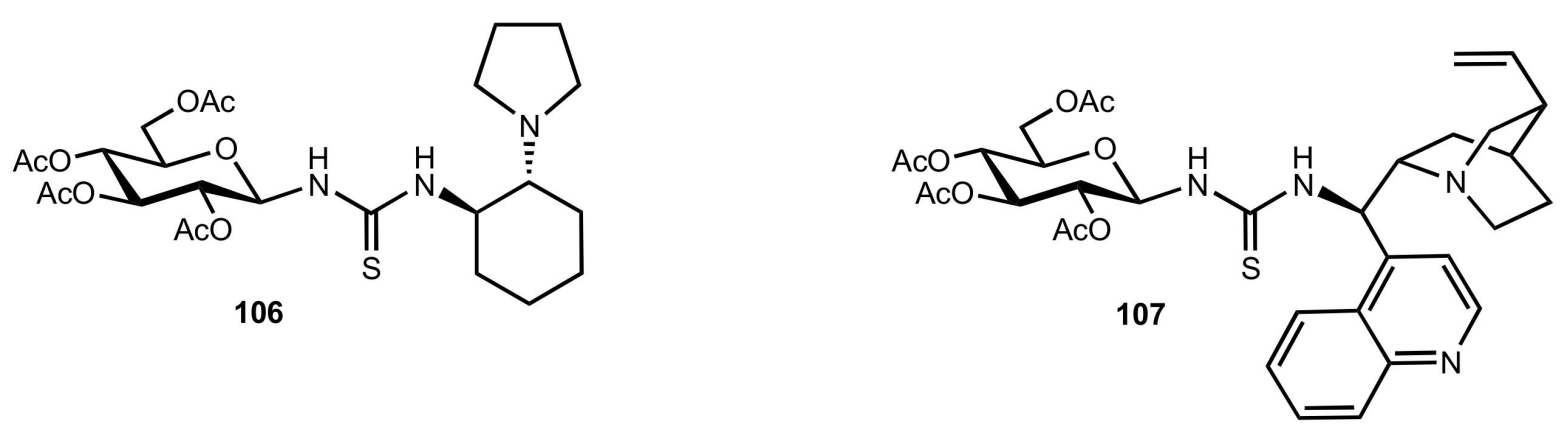
The structure of organocatalysts **106** and **107**.

Miao and co-workers developed an asymmetric synthesis of spiro[chroman-3,3′-pyrazoles] through an oxa-Michael-Michael cascade reaction [56]. Various thiourea-amine catalysts, including the glucose derivatives **18** and **106–107** (Figure 10) were exploited for the stereoselective construction of quaternary stereocenters.

**Scheme 22:**
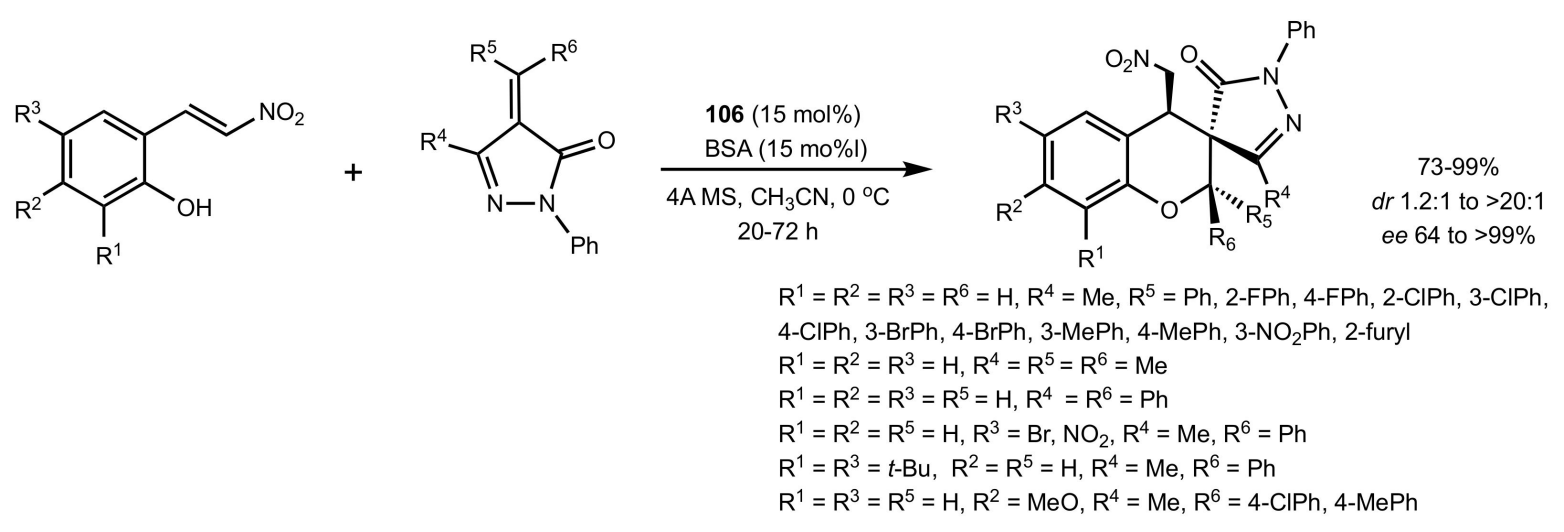
The asymmetric synthesis of spiro[chroman-3,3′-pyrazols] catalysed by **106**.

Catalyst **106** was chosen for the further optimization of the reaction conditions: acetonitrile was identified as the optimal solvent and benzenesulfonic acid (BSA) as the most efficient additive. Moreover, the stereoselectivity was increased by the addition of 4 Å molecular sieves. The scope of the reaction was checked with various (*E*)-nitrostyrenes and pyrazolone derivatives (Scheme 22), and the products were isolated in high to excellent yield (73–99%), variable diastereoselectivity (up to >20:1 *dr*), and, in most cases, high enantioselectivity (up to >99% *ee*).

### 2.2. Aldol Reaction

**Figure 11:**
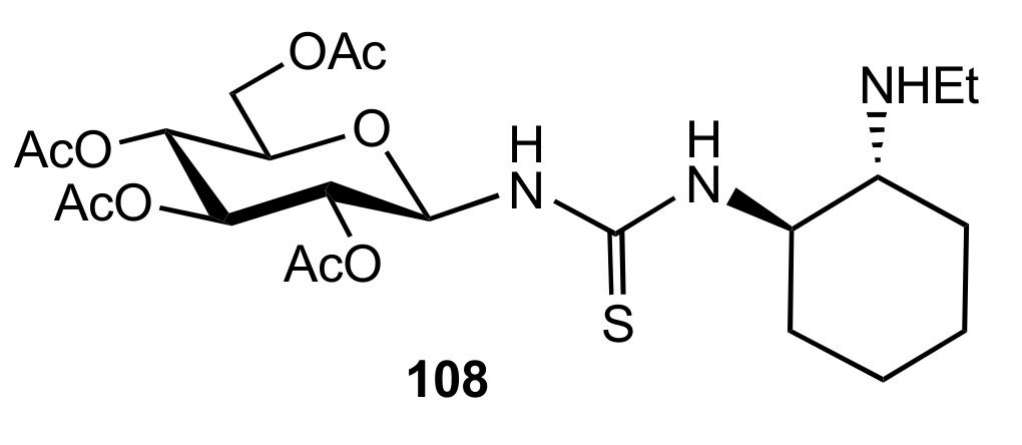
The structure of organocatalyst **108**.

Bifunctional thioureas containing carbohydrate moieties were also able to catalyse other stereoselective transformations leading to the extension of carbon skeleton, such as the aldol reaction. Chiral primary (**7–10**, **15**, **16**, **18**) and secondary (**108**, Figure 11) amine-thioureas were used by Ma’s group for the aldol reaction of acetophenone and trifluoroacetaldehyde methyl hemiacetal [57].

**Scheme 23:**
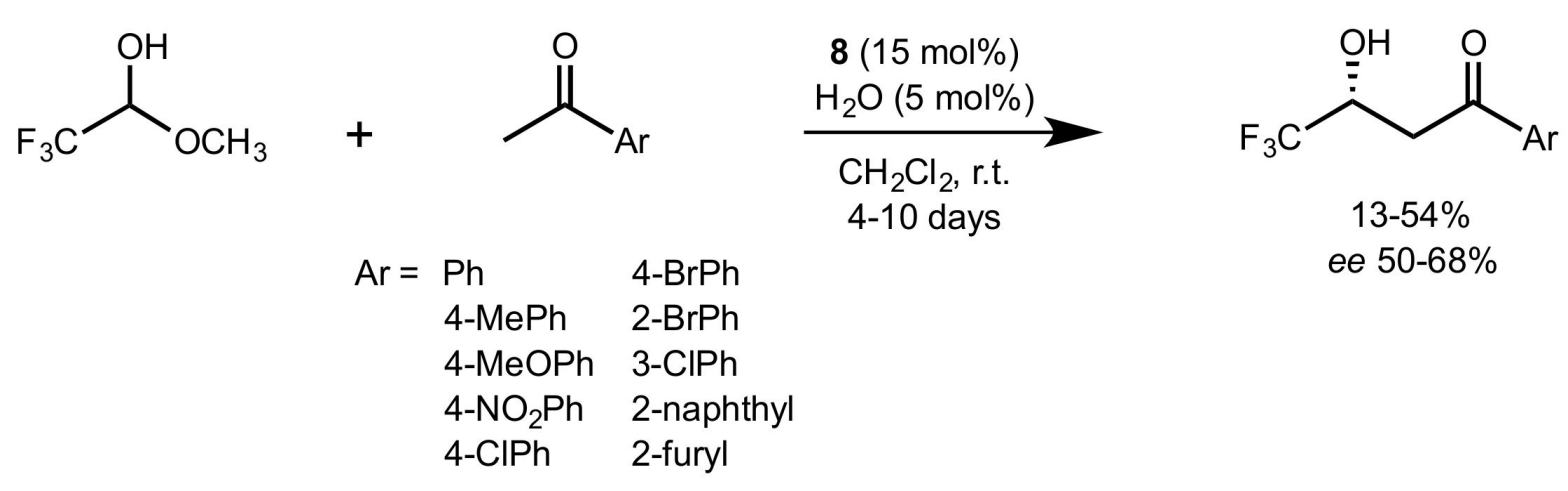
The aldol reaction of trifluoroacetaldehyde methyl hemiacetal with aromatic ketones catalysed by **8**.

The combination of (*R*,*R*)-configured 1,2-diamine with a β-d-glucopyranose moiety afforded (*R*)-β-hydroxy-β-trifluoroalkyl ketones in up to 68% enantiomeric excess (Scheme 23). Replacement of 1,2-diaminocyclohexane (DACH) with 1,2-diphenylethylenediamine resulted in the decrease of enantioselectivity. Maltose and lactose-derived catalysts allowed retaining the *ee* at the 60% level, albeit the yield dropped down to ca. 10%. The presence of the carbohydrate part was found crucial for the stereochemistry of the aldol reaction.

The optimization of the reaction conditions proved that performing the reaction at room temperature, the use of dichloromethane as solvent with 5 mol% of water as the additive and a 15 mol% load of organocatalyst were optimal and improved the chemical yield of the reaction (up to 46%).

### 2.3. Mannich Reaction

The anomeric sugar-urea organocatalysts prepared by Benaglia, Lay, and their co-workers [45] (see Section 2.1) that were successfully employed in the addition of acetylacetone to *trans*-β-nitrostyrene (see Scheme 15), were also used to catalyse the Mannich reaction between diethyl malonate and *N*-Boc imine of benzaldehyde. In all cases, the (*R*)-configured adduct was preferentially formed; however, for the organocatalysts **59**, **60** and **63** (see Scheme 14) (10 mol%), moderate enantioselectivity (75–81% *ee*) and low yield (21–25%) were observed, while the sugar urea **62** (see Scheme 14) gave the adduct in a better yield (55%) but very low enantioselectivity (11% *ee*).

**Figure 12:**
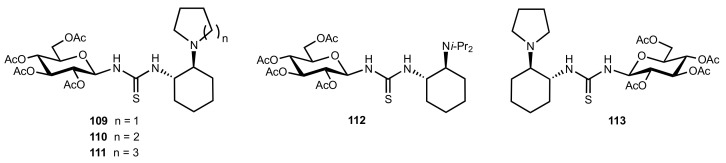
The structure of catalysts **109–113**.

Hydrogen-bond-directed decarboxylative Mannich condensation of 3-oxo-3-arylpropionic [58] or 3-oxo-3-aryloxypropionic acids [59] with cyclic trifluoromethyl ketimines was described by Ma and co-workers. This reaction, which does not occur without the catalyst, was accelerated by the addition of 10 mol% of sugar thioureas **7**, **17**, **22**, **97**, **99**, **106** as well as **109–113** (Figure 12) in THF. The configuration of the β-amino ester product was correlated to the stereochemical series of the carbohydrate moiety. An enantiomeric excess reversal was observed when an l-sugar organocatalyst instead of the d-sugar enantiomer was used.

**Scheme 24:**
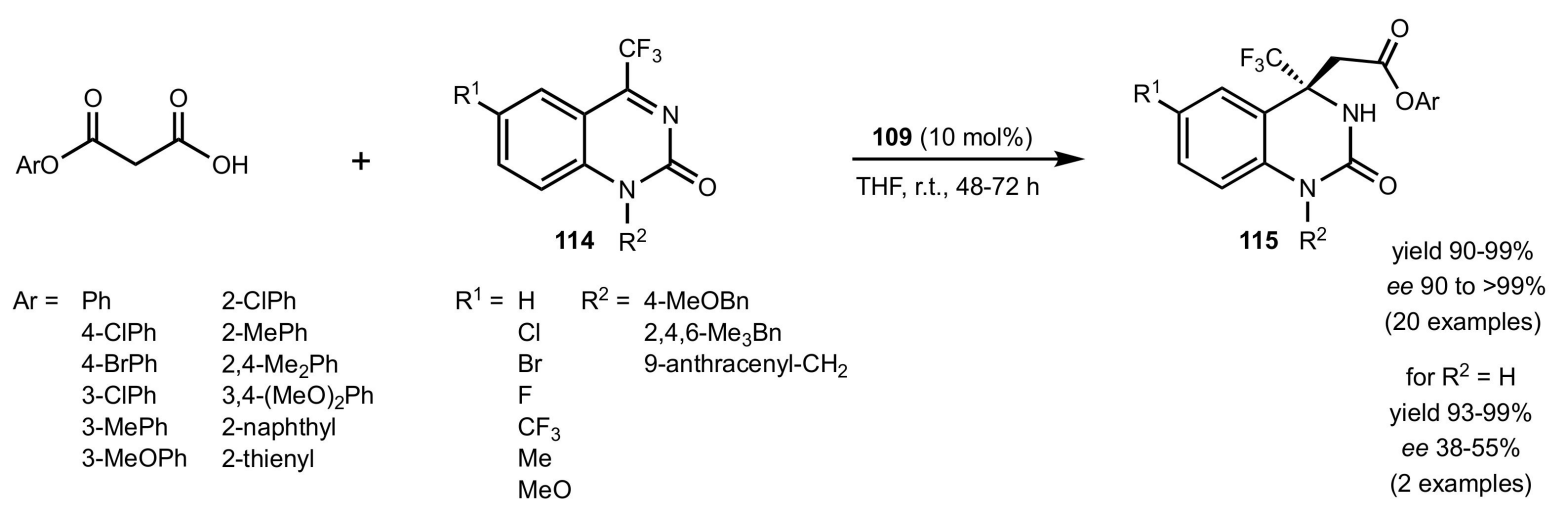
The decarboxylative Mannich reaction of malonic acid monoesters with trifluoromethyl cyclic ketimines.

Among the various sugar thioureas, catalyst **109** proved to be the most efficient. Yields were high (90–99%), and the enantioselectivity in most cases was excellent (90–99% *ee*) when the trifluoromethyl group was present in the structure of ketimine, such as in **114** (Scheme 24). The results were supported with theoretical calculations showing the possible interactions between substrates and the catalyst. The enantioenriched 3,4-dihydro-quinazolin-2(1*H*)-one derivatives **115** bearing a tetrasubstituted stereogenic center were demonstrated to be useful intermediates in the synthesis of the anti-HIV drug DPC 083 [59].

**Figure 13:**
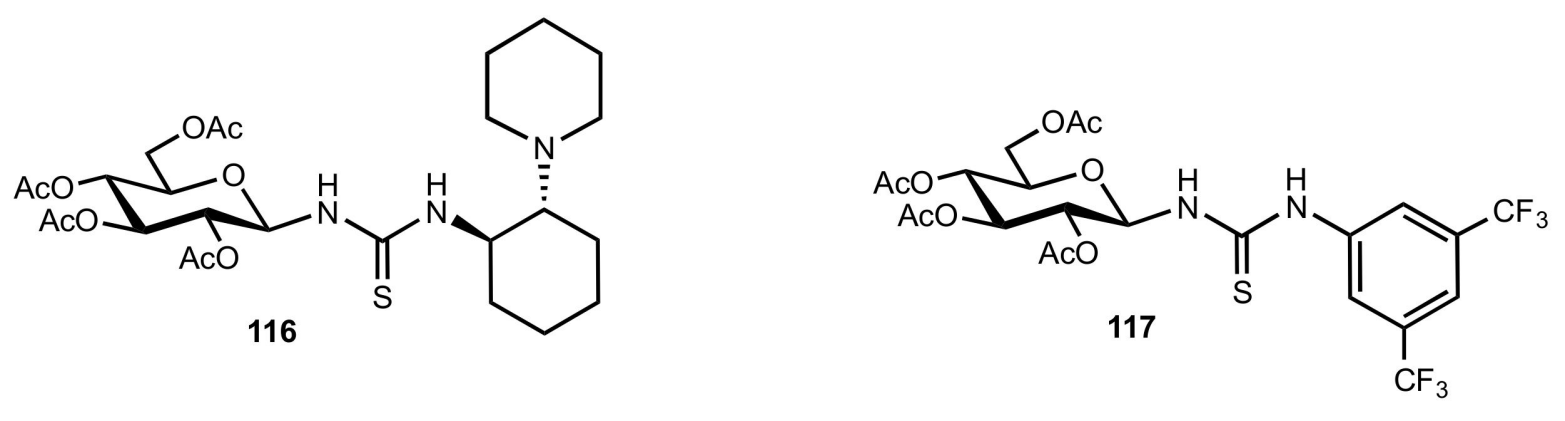
The structure of organocatalysts **116** and **117**.

Ma’s group also reported an asymmetric Mannich reaction of allylic ketones with cyclic *N*-sulfonyl l-iminoester catalysed by the sugar-derived tertiary amino-thioureas **17**, **18** (see Figure 2), **106** (see Figure 10), **116** (Figure 13) as well as the non-aminated sugar thiourea **117** (Figure 13) [60].

**Scheme 25:**
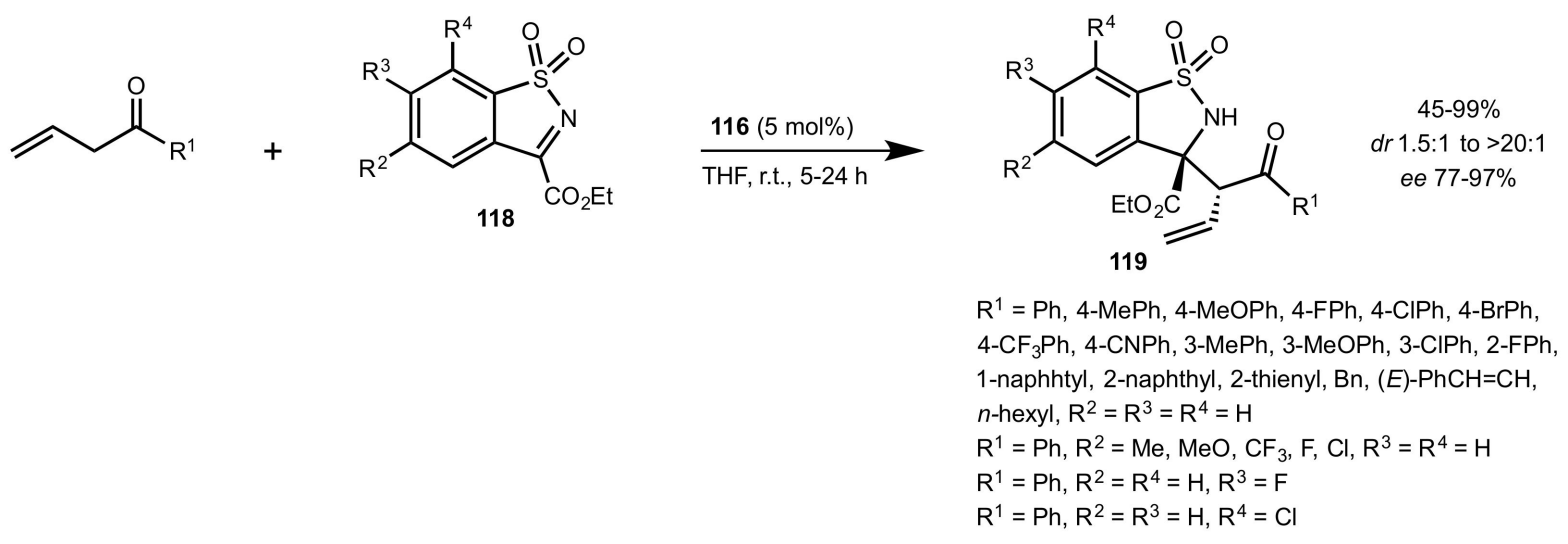
Mannich reaction of allylic ketones and *N*-sulfonyl ketimines.

The use of the sugar thiourea **116**, carrying the (*R*,*R*)-1,2-diaminocyclohexane moiety linked to the β-d-glucopyranose unit, gave the expected products **119** with high level of stereoselectivity (in most cases *dr* >20:1 and 85–97% *ee*) (Scheme 25). The possibility of performing the synthesis on a gram scale was also demonstrated. Tetrasubstituted α-amino esters obtained with high regio-, diastereo-, and enantioselectivity could serve as starting material for the preparation of chiral, non-racemic spiro- and tricyclic benzosultam derivatives.

**Figure 14:**
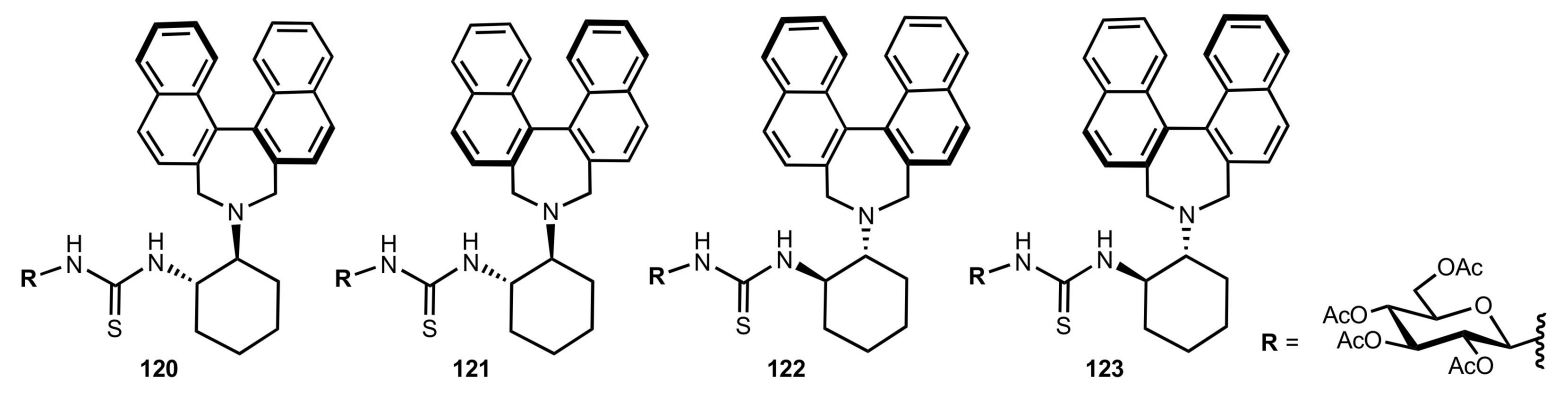
The structure of organocatalysts **120–123**.

Later on, the same research team developed a route to chiral quaternary α-aminophosphonates through the decarboxylative Mannich reaction of ketoacids and cyclic α-ketiminophosphonates [61]. The already known bifunctional thioureas **7**, **17** and **110** as well as the novel organocatalysts **120–123** (Figure 14) bearing a chiral axial binaphthyl part were used to promote this reaction, the latter leading to increased enantioselectivity in a model reaction.

**Scheme 26:**
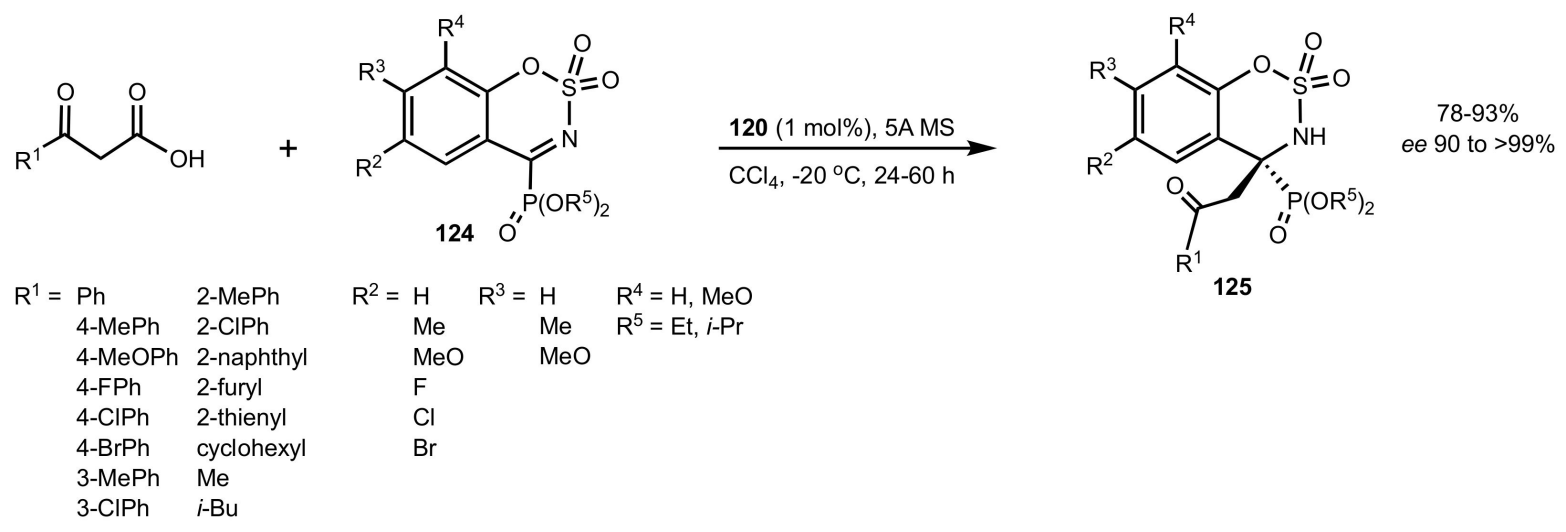
The synthesis of chiral phosphonates catalysed by **120**.

Compound **120** featuring the (*S*,*S*)-diamine part and (*R*)-configured binaphthyl moiety was chosen for the optimization of conditions. The use of CCl_4_ as the solvent and 5 Å molecular sieves (−20 °C, 18 h) improved the yield to 86% and the enantiomeric excess to 99%. The catalyst load could be reduced from 10 to 1 mol% with no loss of enantioselectivity and without a significant decrease of yield (although 46 h were required to complete the reaction). A wide substrate scope (Scheme 26), and possibility of performing the process on a gram scale were demonstrated. The reaction was also performed starting from five-membered cyclic α-ketiminophosphonates (three examples, 77–82% yield and 90–91% *ee*).

### 2.4. Aza–Henry (Nitro-Mannich) Reaction

**Scheme 27:**
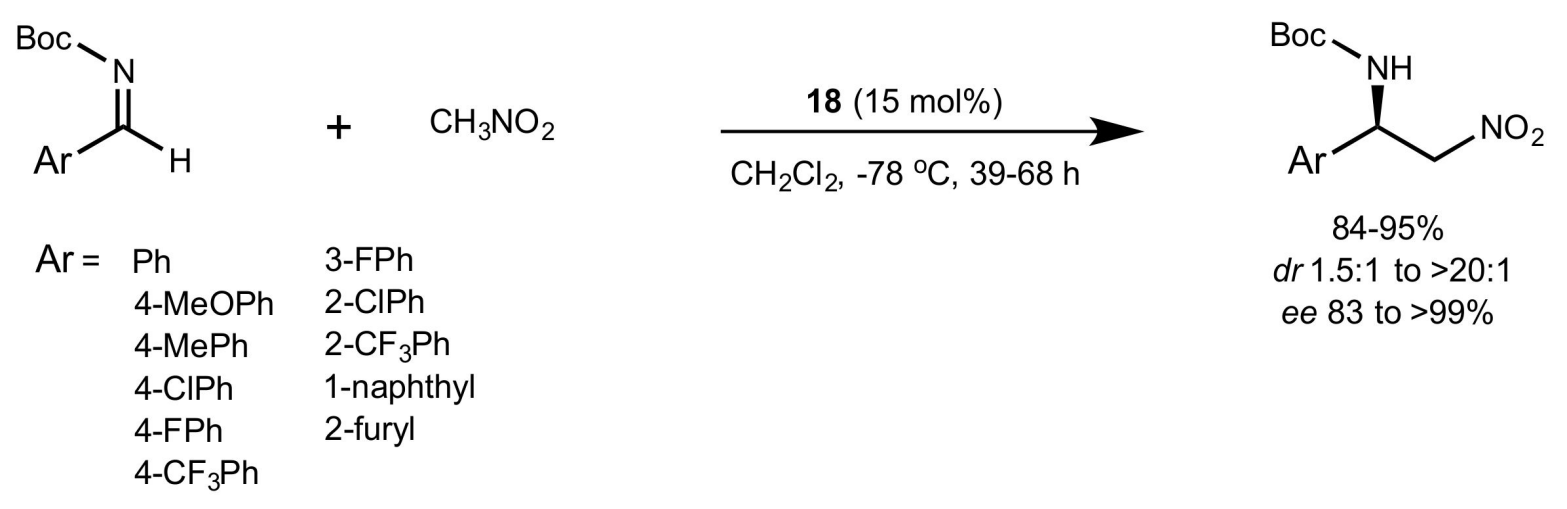
The Aza–Henry reaction catalysed by **18**.

The sugar thioureas derived from d-glucose **18**, d-galactose **23** and d-lactose **20** were also exploited for the nucleophilic addition of nitroalkanes to the C=N bond of imines (aza-Henry reaction) [62]. Aromatic imines bearing different *N*-protecting groups were reacted with nitromethane at low temperature in the presence of 15 mol% of organocatalyst. The best results were observed starting from *N*-Boc imines and using the sugar thiourea **18** at −78 °C (Scheme 27). In the case of nitroethane as the nucleophile, a higher temperature was required, whereas nitropropane did not afford the desired product.

**Figure 15:**
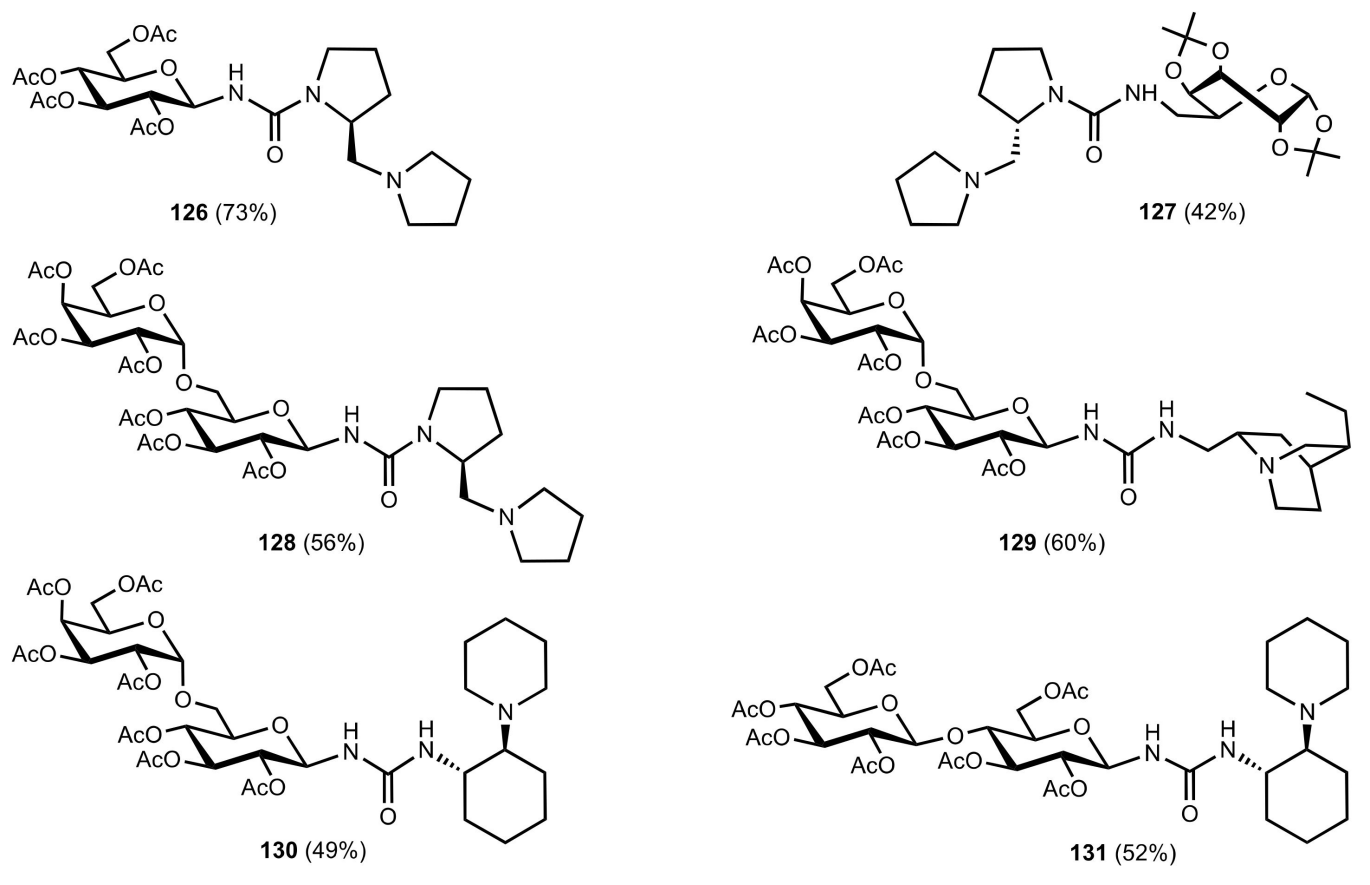
The structure of organocatalysts **126–131**.

The sugar urea organocatalysts **126–131** bearing tertiary amino groups were synthesised by Porwański and co-workers by treatment of sugar azides with triphenylphosphine and an appropriate chiral amine under CO_2_ bubbling (Figure 15) [63].

The tetra-*O*-acetyl-β-d-glucopyranosyl azide was prepared from the d-glucose via acetylation, bromination of anomeric position (as described above for the synthesis of isothiocyanates) and reaction with sodium azide at room temperature in DMF (81%) [64]. The 6-azido-6-deoxy-1,2:3,4-di-*O*-isopropylidene-α-d-galactopyranose, precursor of the urea **127**, was easily obtained from the corresponding 6-hydroxyl derivative by tosylation and nucleophilic substitution with sodium azide [65]. Finally, the 1,2-*trans* configured disaccharidic azides were prepared with a 75–85% yield by treating the corresponding β-d-octaacetates with trimethylsilyl azide in the presence of SnCl_4_ [66].

**Scheme 28:**
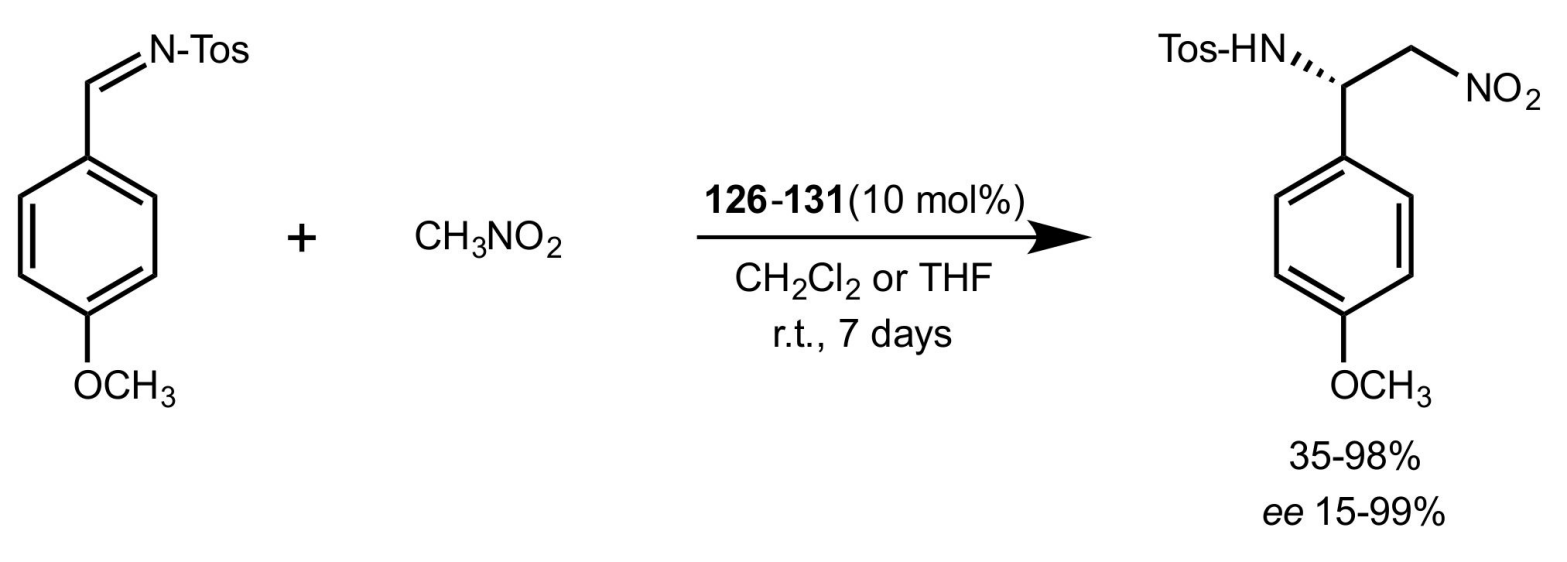
**The** Aza–Henry reaction catalysed by **126–131**.

The new organocatalysts were tested in the reaction of a single *N*-tosyl imine with nitromethane. The best results in this aza-Henry reaction were obtained with the melibiose-urea derivative **130** (10 mol%) in dichloromethane, which afforded the adduct at a 98% yield and 97% *ee* (Scheme 28) [63].

### 2.5. Morita–Baylis–Hillman Reaction

**Figure 16:**
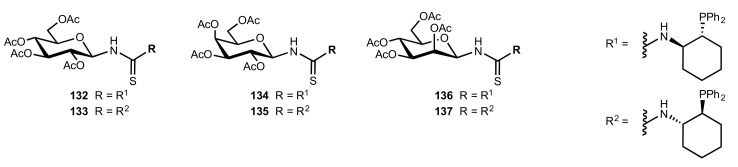
The structure of organocatalysts **132–137**.

Bifunctional phosphinothioureas were proved to be efficient catalysts of Morita–Baylis–Hillman (MBH) reaction. Wu and co-workers exploited the sugar thioureas **132–137** (Figure 16) containing an (*R*,*R*)- or (*S*,*S*)-*trans*-2-amino-1-(diphenylphosphino)cyclohexane moiety in the MBH reaction between acrylates and aldehydes [67].

**Scheme 29:**
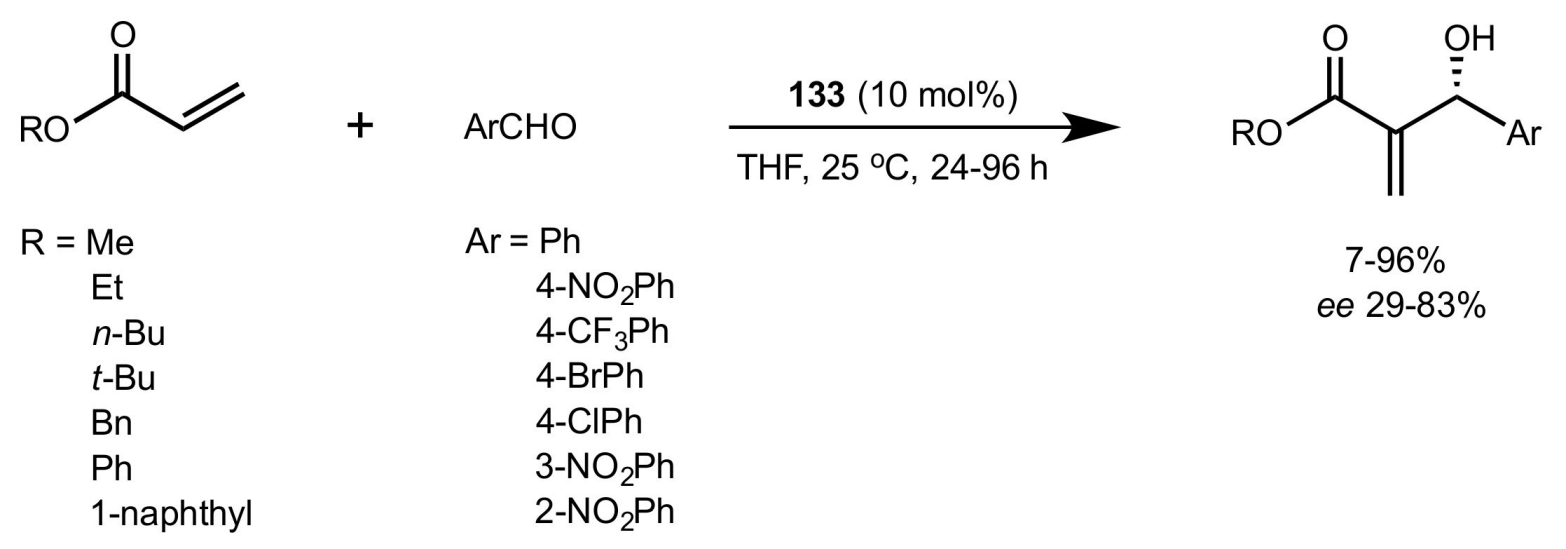
Morita–Baylis–Hillman reaction catalysed by **133**.

Chiral allylic alcohols—useful building blocks for asymmetric synthesis—were obtained in good to excellent yield and moderate to good enantioselectivity (68–83% *ee* in most cases) under optimized conditions (Scheme 29).

**Figure 17:**
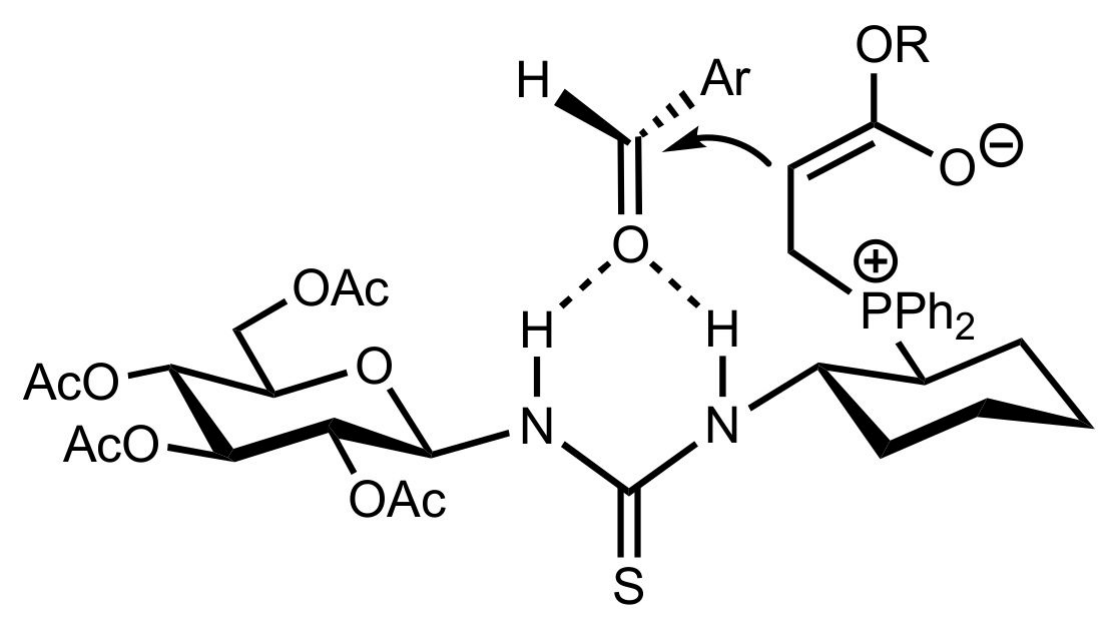
The proposed transition state for the Morita–Baylis–Hillman reaction catalysed by **133**.

In the transition state for the Morita–Baylis–Hillman reaction proposed by Wu and co-workers [67], the thiourea group forms a hydrogen bond with the oxygen of the aromatic aldehyde, whereas the phosphinoyl-associated enolate ion attacks the carbonyl group from the *si*-face to afford the (*R*)-configured product (Figure 17).

**Figure 18:**
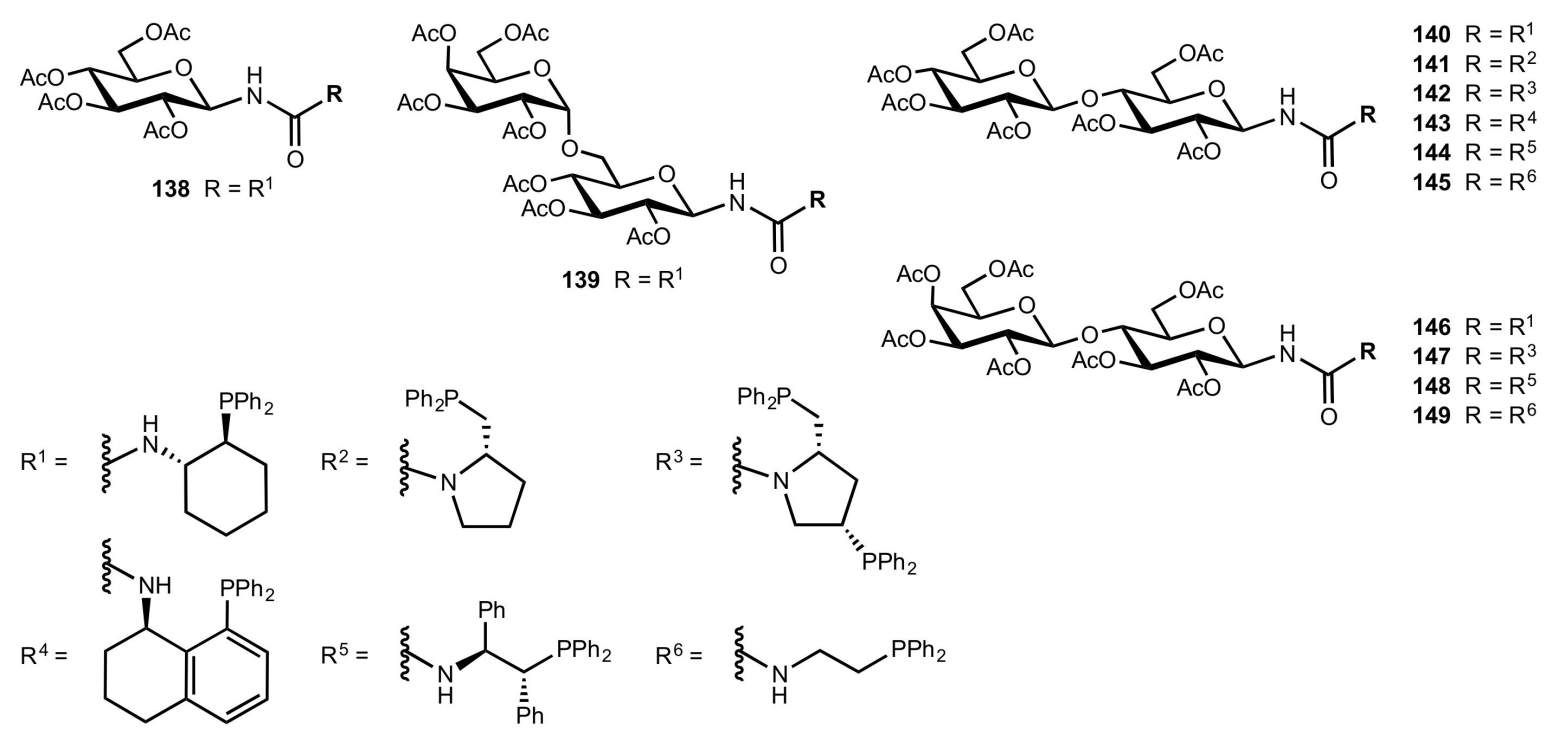
The structure of organocatalysts **138–149**.

Two years later, Porwański reported the synthesis of twelve sugar ureas containing a diphenylphosphinyl group and their application in the MBH reaction [68]. The new organocatalysts **138–149** (Figure 18) were prepared in 60–99% yield by reacting the peracetylated glucosyl, melibiosyl, lactosyl and cellobiosyl azides and the amines bearing phosphine function(s) with triphenylphosphine under CO_2_ bubbling.

**Scheme 30:**
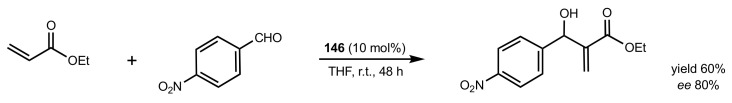
Morita–Baylis–Hillman reaction catalysed by **146**.

The twelve organocatalysts **138–149** were employed in a single Morita–Baylis–Hillman reaction involving ethyl acrylate and *p*-nitrobenzaldehyde. It was found that only **146** gave satisfactory results, whereas **141–144**, **147** and **148** were almost unreactive (Scheme 30). The organocatalysts **139**, **140** and **146** were also tested in the model aza-Henry reaction between an *N*-tosyl imine and nitromethane, but no enantioselectivity was observed.

### 2.6. Other Asymmetric Transformations

**Figure 19:**
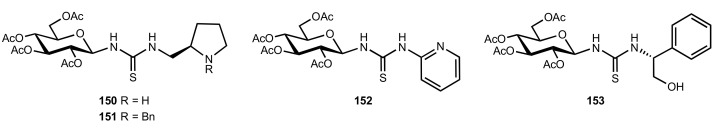
The structure of organocatalysts **150–153**.

Miao, Chen and their co-workers reported [69] the synthesis of functionalized 3,4-dihydropyrimidin-2(1*H*)-ones (DHPMs), important antiviral, antitumor, antibacterial and anti-inflammatory compounds, based on a three-component condensation between aldehydes, urea or thiourea and ethyl acetoacetate (Biginelli reaction). This condensation was catalysed by various sugar thioureas including the new catalysts **150–153** (Figure 19).

**Scheme 31:**
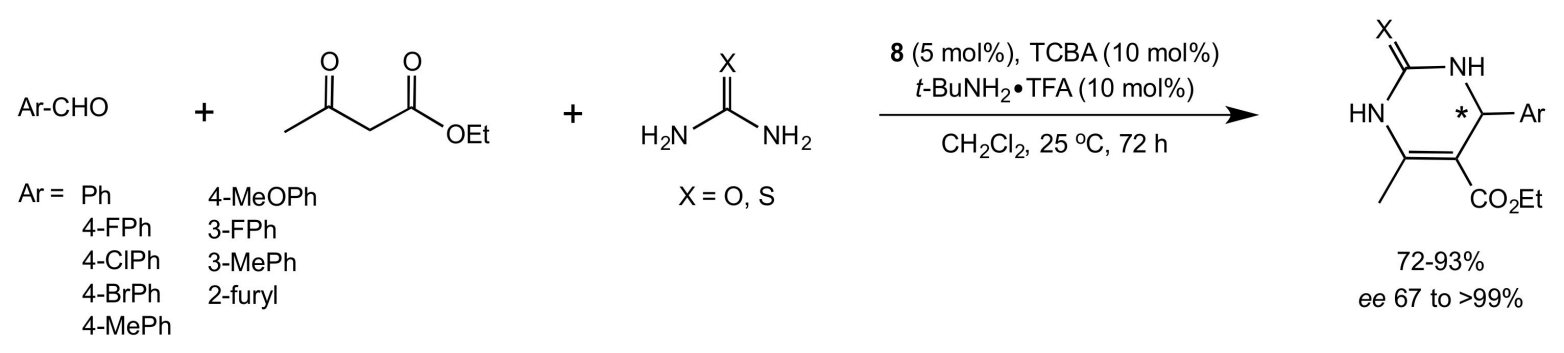
The Biginelli reaction catalysed by compound **8**.

DHPMs were obtained in high yields (72–93%) and enantiomeric excess (67 to >99%) when aryl aldehydes were used (Scheme 31), while butanal gave a 51% yield and only 15% *ee*. The optimized conditions included the use of catalyst **8** (5 mol%) and 2,4,6-trichlorobenzoic acid (TCBA) together with *t*-butylammonium trifluoroacetate as the most efficient additive. Changing the configuration of the amine moiety of the organocatalyst led to the product of opposite configuration.

**Scheme 32:**
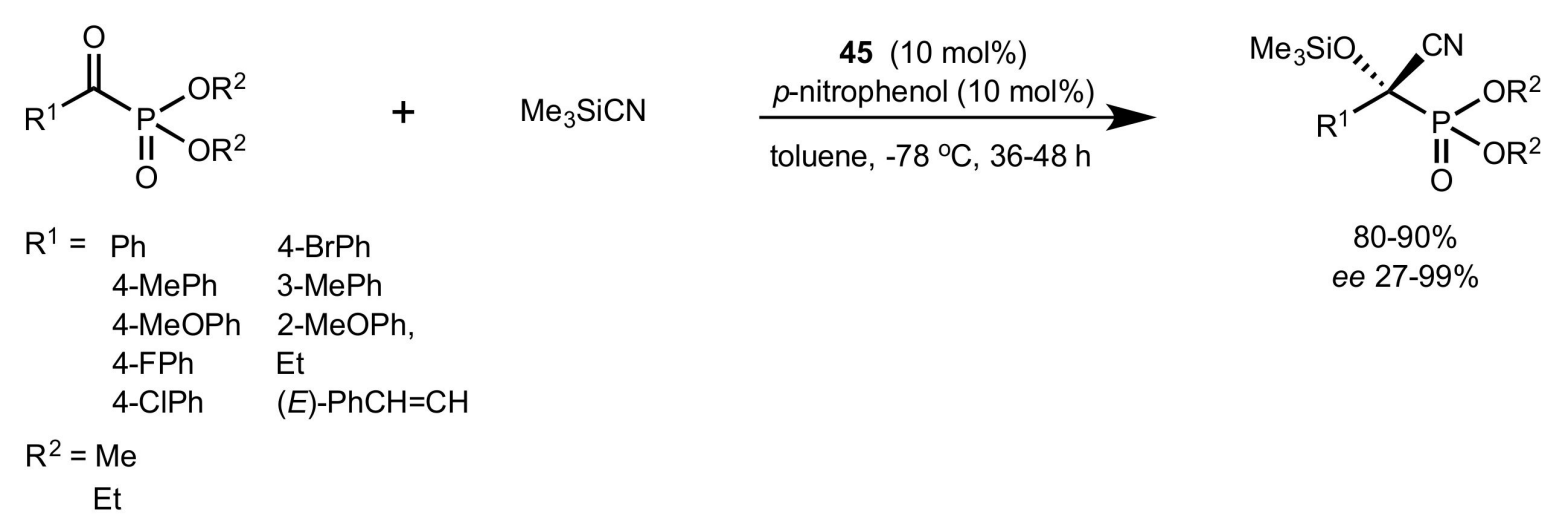
The asymmetric cyanation catalysed by **45**.

The combination of cinchona alkaloids and carbohydrates in the same organocatalyst (**44**, **45** and **107**) was useful for the asymmetric cyanation of α-ketophosphonates with Me_3_SiCN [70]. The reaction gave tertiary α-hydroxy phosphonates—very important products from the pharmaceutical point of view, in high yield and enantiomeric excess (Scheme 32). The presence of the tertiary amine thiourea unit was essential for the stereochemical outcome of the cyanation.

The best results were observed for reactions performed in toluene at −78 °C in the presence of 10 mol% of organocatalyst **45** and 10 mol% of an alcohol or phenol (*p*-nitrophenol was the best choice). Yields (80–90%) as well as the enantiomeric excesses (83–99%) were high in all cases with the exception of aliphatic α-ketophosphonates and *ortho*-substituted benzoyl phosphonates.

## 3. Sugar Ketones

Sugar-derived ketone organocatalysts have been largely employed in asymmetric reactions. The majority of publications on these organocatalysts were dealing with asymmetric epoxidation and the others with the asymmetric oxidation of disulfides. A vast amount of different organocatalysts have been synthesised, mainly from d-fructose and d-glucose, and modified over the past 25 years to satisfy the demands of particular transformations. Typically, the position 3 of the sugar is oxidised to produce the ketone function.

### 3.1. Asymmetric Epoxidation

In the total synthesis of chiral compounds, the asymmetric epoxidation is often an indispensable tool. Contrary to the metal-catalysed Sharpless epoxidation, the organocatalytic epoxidation by means of sugar ketones is a valuable approach for the metal-free synthesis of pharmaceutical active chiral compounds. Although various reviews have extensively covered this topic [9,10,11,12,13], for the comprehensiveness of the present review, we included previously described examples and reported the more recent achievements. Moreover, we added the detailed preparations of each organocatalysts from commercially available sugars. For sake of clarity, the results obtained by the three major research teams are described separately.

#### 3.1.1. Achievements Reported by Shi and Co-Workers

##### 3.1.1.1. Epoxidation of *Trans*-Alkenes

Epoxidation of simple *trans*-alkenes

**Scheme 33:**
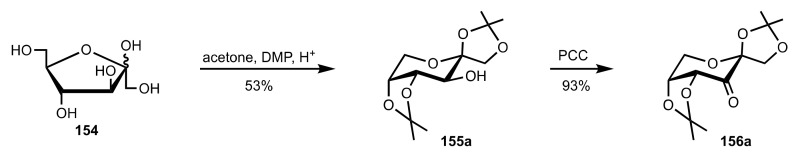
The synthesis of sugar ketone organocatalyst **156a** from d-fructose.

Readily available ketone organocatalysts derived from sugars were prepared and used by Shi and co-workers in 1996 [71], although the synthetic route was established earlier [72]. In a two-step preparation, ketone **156a** was obtained from d-fructose (**154**) by ketalization with acetone and 2,2′-dimethoxypropane (DMP) and subsequent oxidation with pyridinium chlorochromate (PCC) (Scheme 33).

**Scheme 34:**
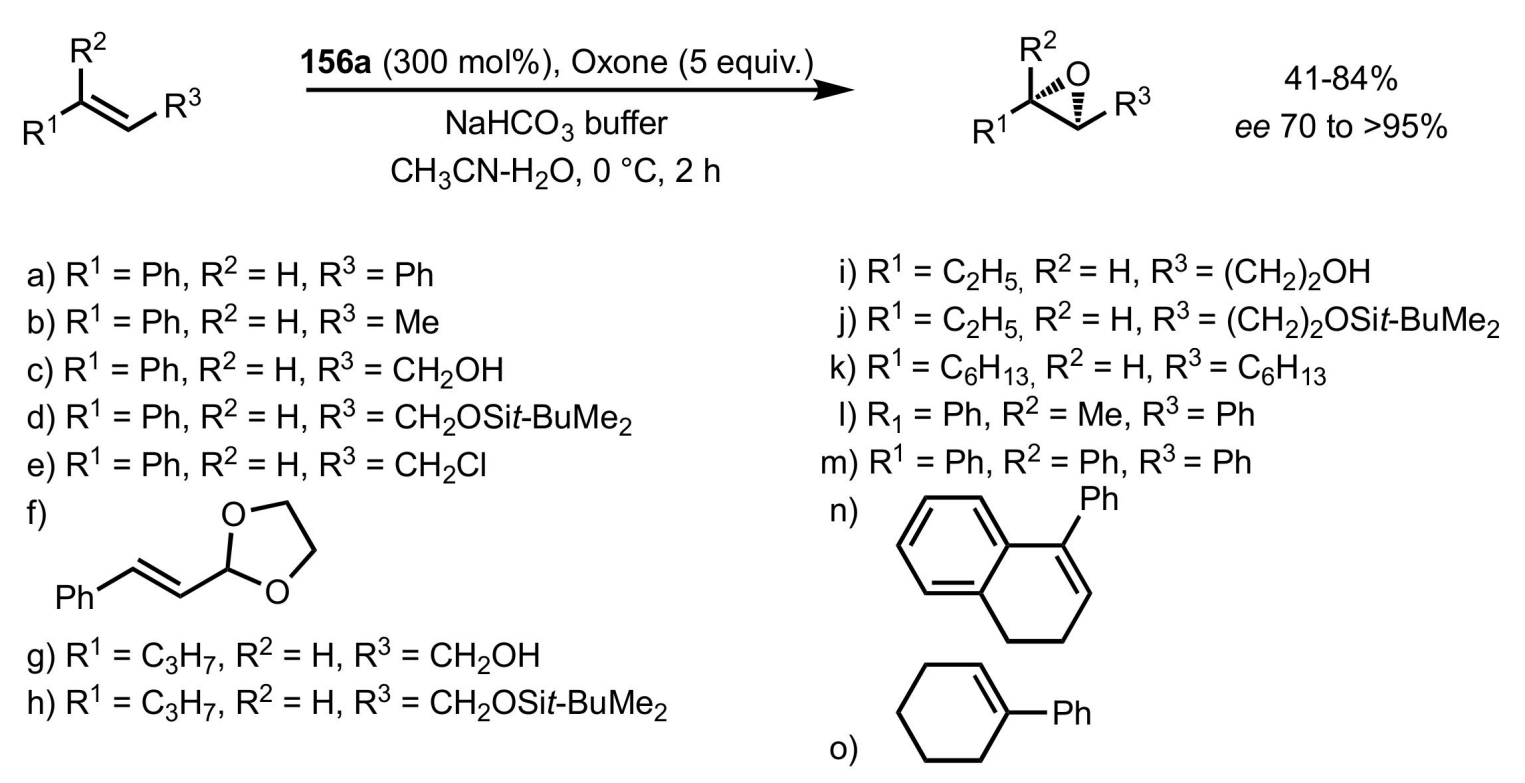
The first asymmetric epoxidations using sugar ketone organocatalyst **156a** reported by Shi and co-workers.

Good yields and high stereoselectivity were observed for the epoxidation of a variety of alkenes (Scheme 34). Although the yields and enantiomeric excesses were satisfactory, high concentrations of organocatalyst and co-catalyst (3 and 5 equiv., respectively) made the protocol impractical.

**Scheme 35:**
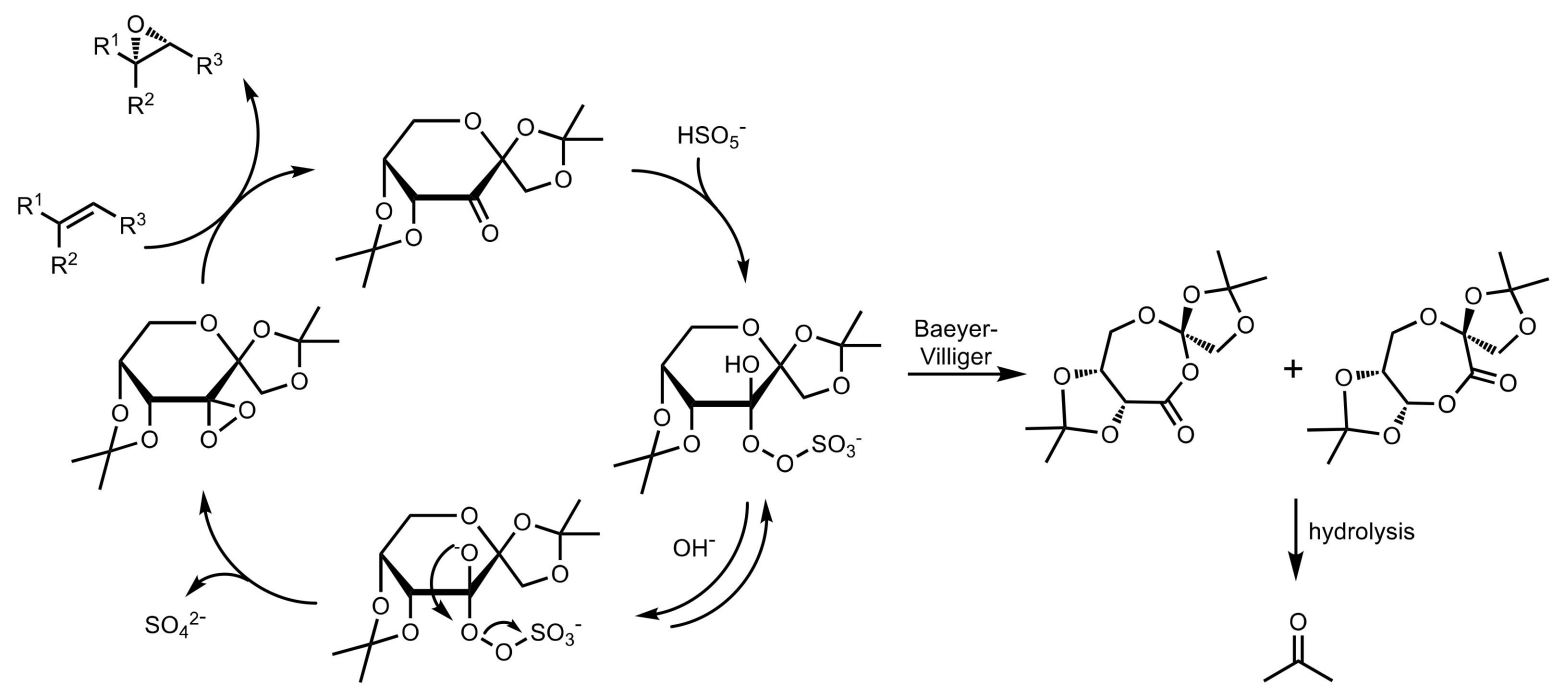
The formation of the active dioxirane species from Oxone in the asymmetric epoxidation.

Regardless of the rapid decomposition of Oxone at high pH (Scheme 35), positive effects were observed by increasing the pH: the catalytic load was lowered significantly (0.2–0.3 equiv. of organocatalyst and 1.38 equiv. of Oxone), and yields as well as enantiomeric excesses remained high and even increased (*trans*-stilbene: 97% *ee*) [73].

**Figure 20:**
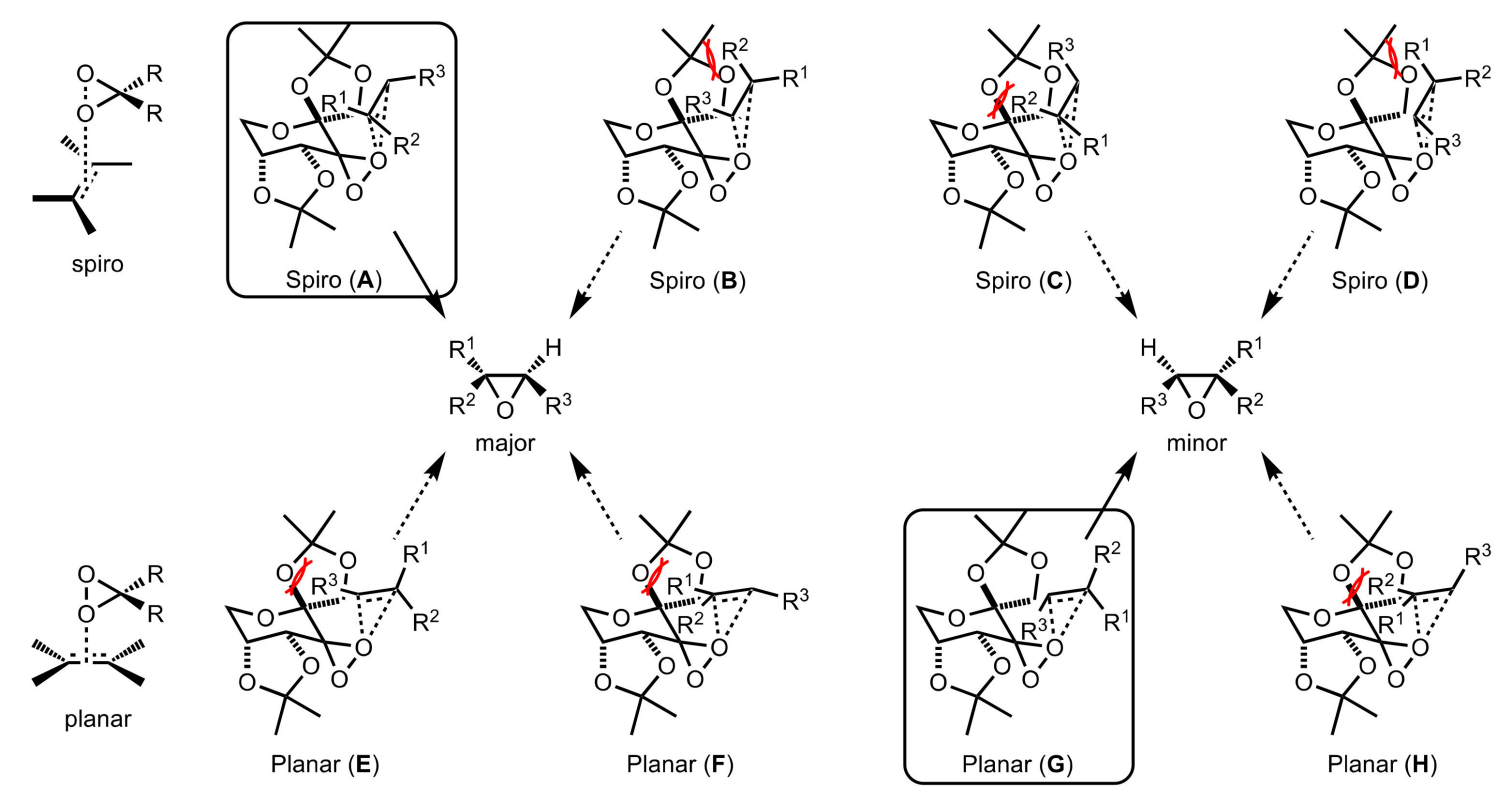
The proposed transition states **A–H** for the asymmetric epoxidation catalysed by **156a**.

Epoxidations by active dioxirane species are possible in two orientations, either spiro or planar, with respect to the vinyl group. All possible transition states are shown in Figure 20. Among them, only two of the transition states are not sterically hindered: spiro **A** and planar **G**, with the former preferred as revealed by the analysis of the structure of products [74].

**Scheme 36:**
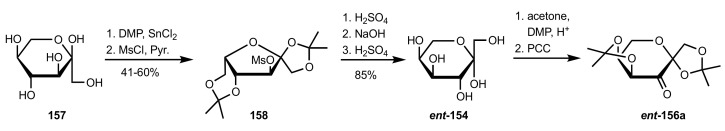
The synthesis of sugar ketone organocatalyst ***ent*-156a** from l-sorbose.

In the same paper [74] the authors also described the synthesis of ***ent*-156a** (Scheme 36), an enantiomer of **156a**, from l-fructose (***ent*-154**), obtained from l-sorbose (**157**) by ketalization, mesylation and a sequence of acid-base-acid treatment as already reported [75]. Unfortunately, the yields of the last two steps were not reported.

**Scheme 37:**
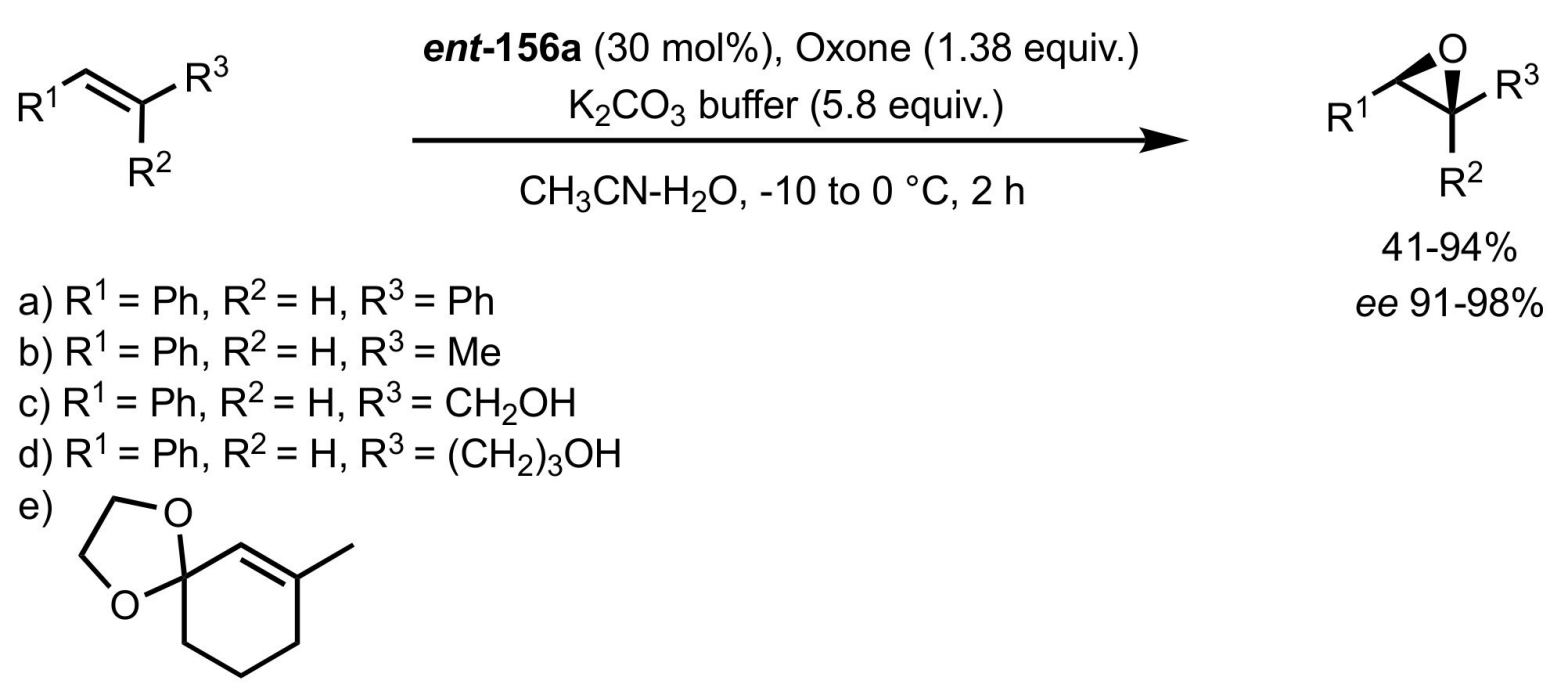
The asymmetric epoxidation catalysed by ***ent*-156a**.

Asymmetric epoxidation catalysed by ***ent*-156a** afforded the oxirane with the opposite configuration in equal enantiomeric excess (Scheme 37) [74,76].

Epoxidation of *trans*-enynes, -vinylesters and -vinylsilanes

**Scheme 38:**
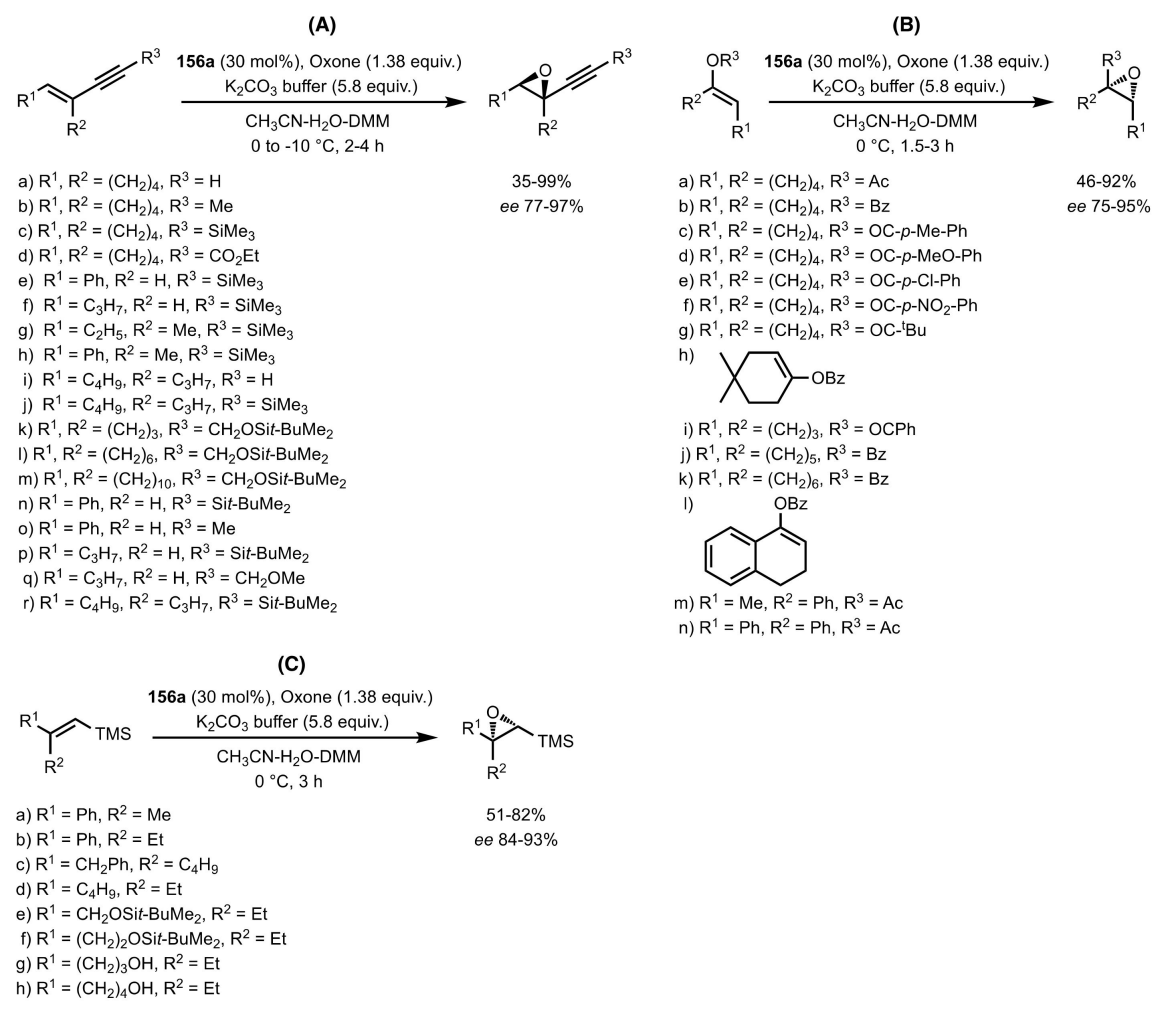
The epoxidation of enynes (**A**), vinylesters (**B**) and vinylsilanes (**C**) catalysed by **156a**.

Sugar ketone organocatalysts **156a** and its enantiomer ***ent*-156a** (in one example of enyne (*S,S*)), were applied with good yields and high chemo-, regio- and enantioselectivity in the asymmetric epoxidations of *trans*-olefins and trisubstituted alkenes. Substrates, such as enynes [77,78], vinylesters [79,80] and vinylsilanes [81] were tested (Scheme 38). Furthermore, an application in kinetic resolution of enantiomers was described [82]. By changing the oxidant from Oxone to nitrile/hydrogen peroxide a mild and inexpensive oxidizing system was achieved [83,84]. Acetonitrile proved to be the most efficient nitrile. In spite of promising results, no further approaches were made.

New organocatalysts for the epoxidation of *trans*-alkenes

**Scheme 39:**
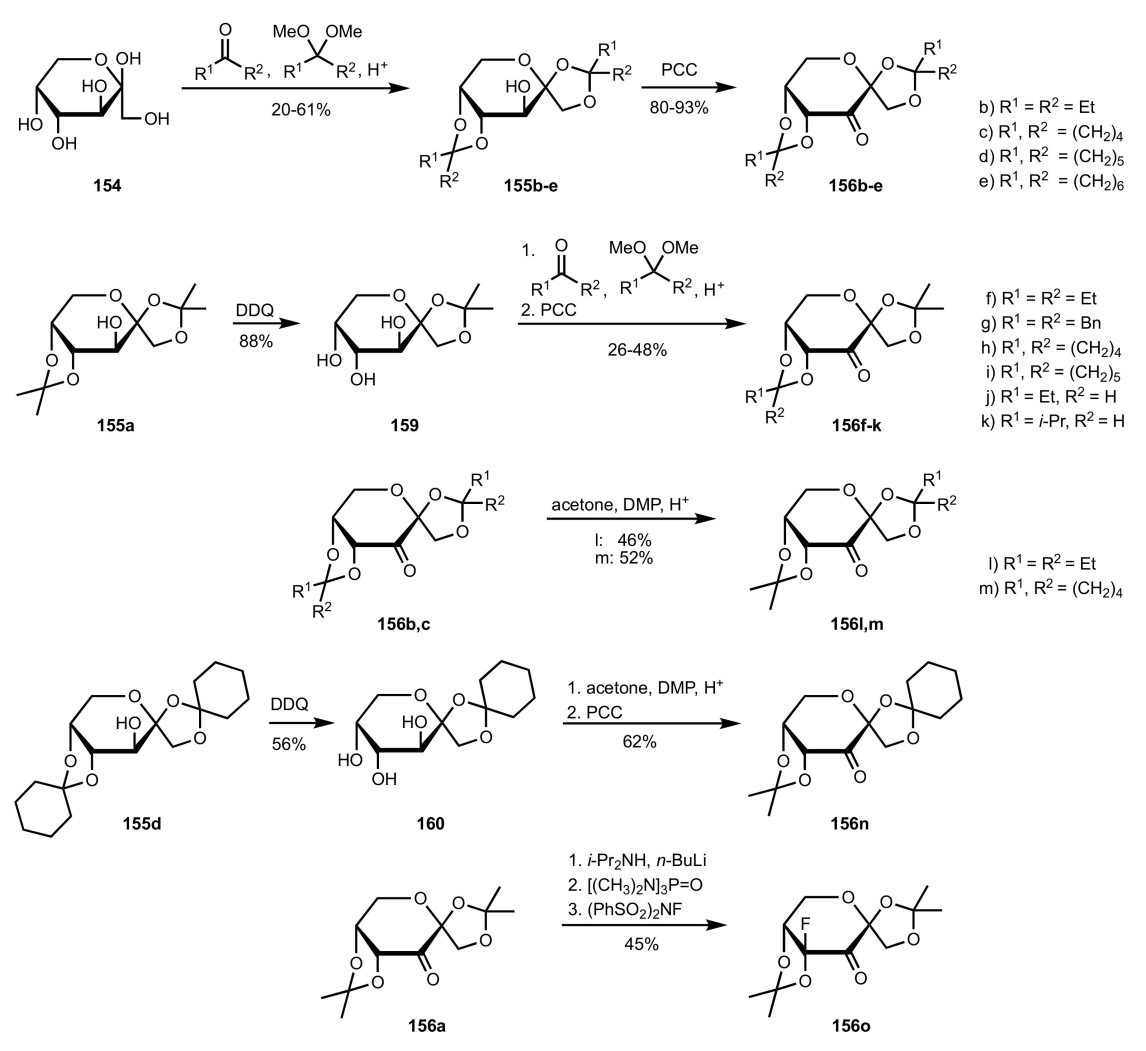
The synthesis of sugar ketone organocatalysts **156b-o** from d-fructose (**154**).

As sugar ketone **156a** was found inefficient in the enantioselective epoxidation of terminal olefins as well as *cis*-alkenes, a large number of new organocatalysts were prepared by Shi and co-workers starting from d-fructose [85]. The compounds **156b-e** were synthesised using various ketones and dimethoxyalkanes for the initial ketalization of the ketose, followed by standard oxidation (Scheme 39). A regioselective deprotection of **155a** using 2,3-dichloro-5,6-dicyano-*p*-benzoquinone (DDQ) [86], followed by ketalization with different ketones and oxidation, yielded catalysts **156f-k**.

A direct regioselective transketalization of ketones **156b** and **156c** using acetone and DMP led to the organocatalysts **156l,m**. Organocatalyst **156n** was obtained from alcohol **155d** as described for the synthesis of **156f-k**. Exploiting the electrophilic fluorination with *N*-fluoro-benzenesulfonimide developed by Differding and Ofner [87], the ketone **156o** was prepared from **156a** at a 45% overall yield.

**Scheme 40:**
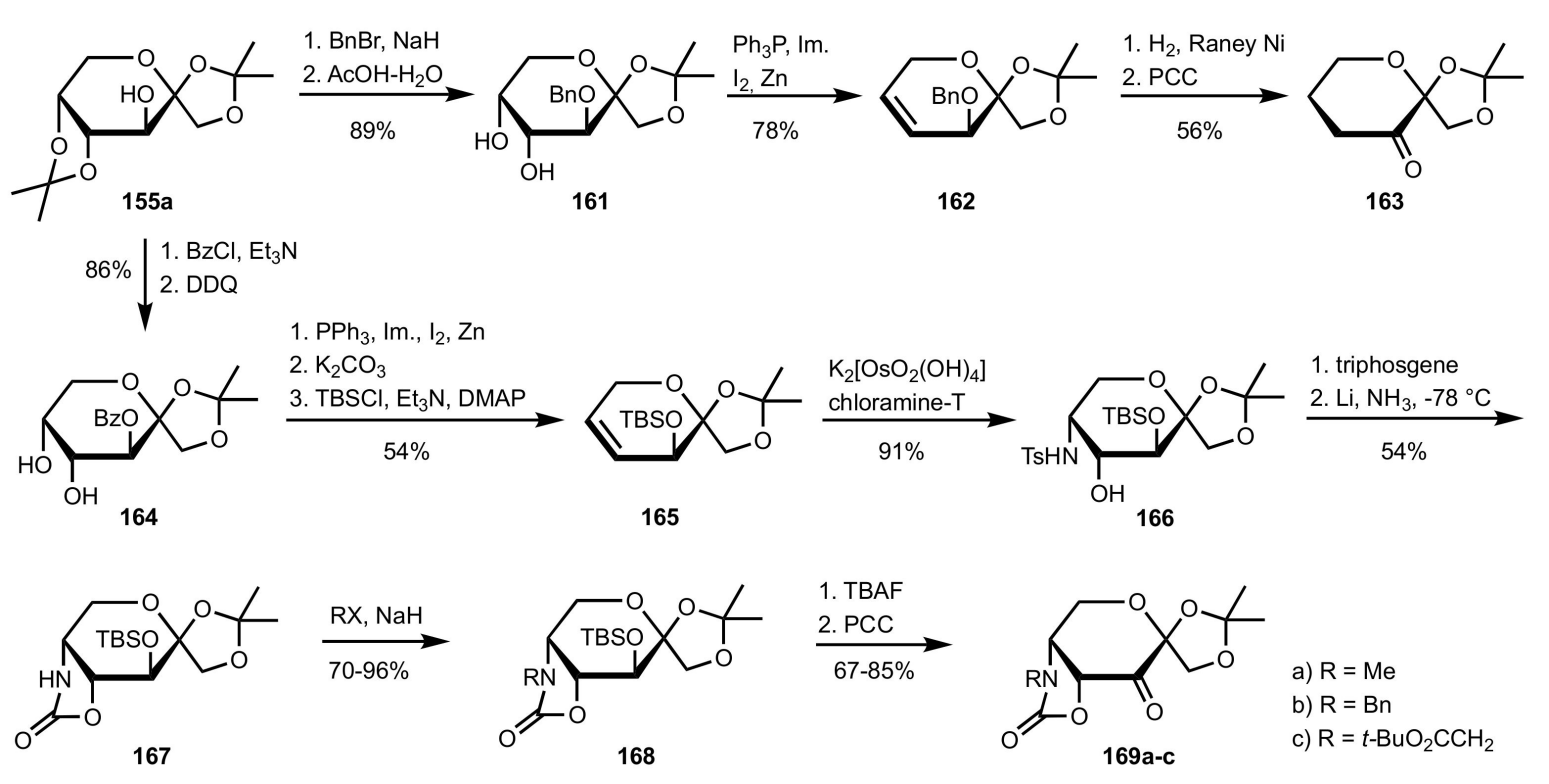
The synthesis of sugar ketone organocatalysts **161** and **169a-c** from **155a**.

Deoxygenated organocatalyst **163** was synthesised from **155a** through a five-step reaction sequence, including standard benzylation and regioselective hydrolysis of an isopropylidene group, to give the diol **161** (Scheme 40) [88]. Elimination of the vicinal hydroxyl groups [89,90], followed by hydrogenation of the double bond and oxidation of the alcohol afforded the ketone **163**.

On the other hand, benzoylation of **155a** and subsequent regioselective hydrolysis, elimination and conversion of the ester into silyl ether function led to the alkene **165**. Oxyamination of the latter [91,92] followed by carbamate formation using triphosgene and deprotection of the nitrogen atom by lithium in liquid ammonia yielded **167**. Upon *N*-alkylation with various alkyl halides, deprotection of the secondary alcohol and its oxidation, **167** led to **169a-c** (Scheme 40) [93].

**Scheme 41:**
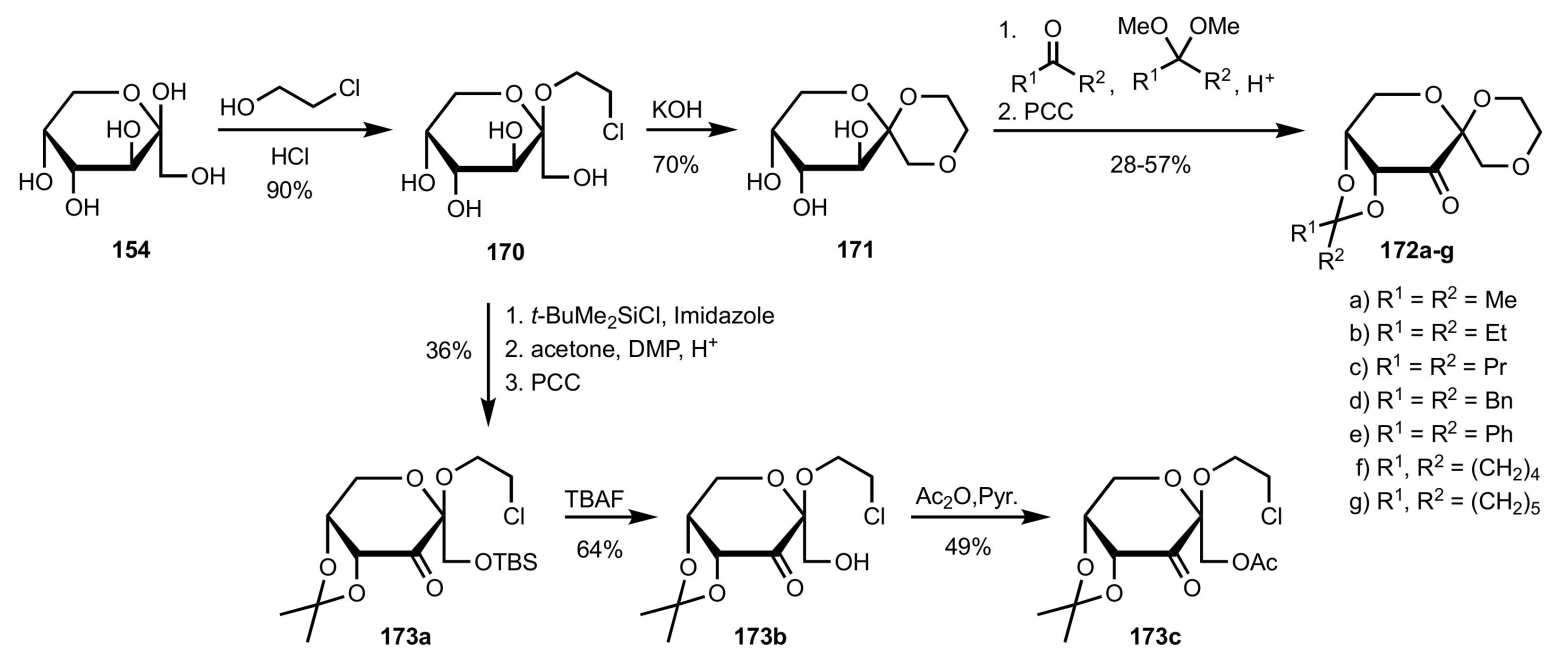
The synthesis of sugar ketone organocatalysts **172a-g** and **173a-c** from d-fructose.

The ketone organocatalysts **172a-g** were prepared from d-fructose by glycosidation with chloroethanol to give **170** [94] and subsequent ring closure leading to **171 [95]** (Scheme 41). Ketalization followed by oxidation of the OH-3 function afforded **172a-g**. Regioselective silylation of the primary hydroxyl group of **170** followed by ketalization and oxidation gave the organocatalyst **173a,** from which **173b** was obtained by deprotection of the primary alcohol and **173c** by acetylation of the latter (Scheme 41) [85].

**Scheme 42:**
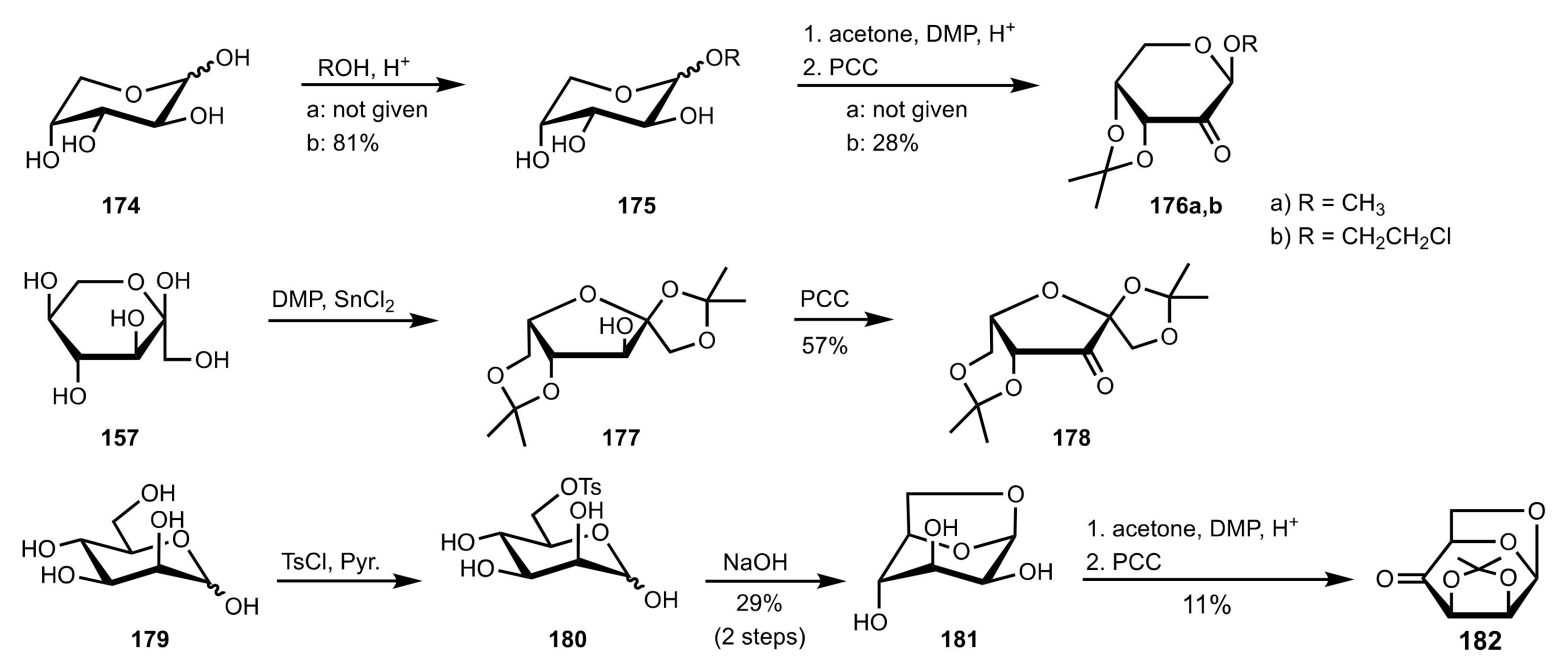
The synthesis of sugar ketone organocatalysts **176a-b**, **178** and **182**.

The two organocatalysts **176a,b** were synthesised [85] from d-arabinose (**174**) using a previously reported synthetic approach [96] (Scheme 42). Unfortunately, the yield was not reported for **176a**. The alcohol **177**, obtained by ketalization of l-sorbose (**157**) was oxidised with pyridinium chlorochromate (PCC) to give the ketone **178 [85]** (Scheme 42). The organocatalyst **182** was synthesised from d-mannose by regioselective tosylation and intramolecular nucleophilic substitution to afford the 1,6-anhydrosugar **181 [97]**. Subsequent ketalization and oxidation allowed the formation of **182** (Scheme 42).

**Scheme 43:**
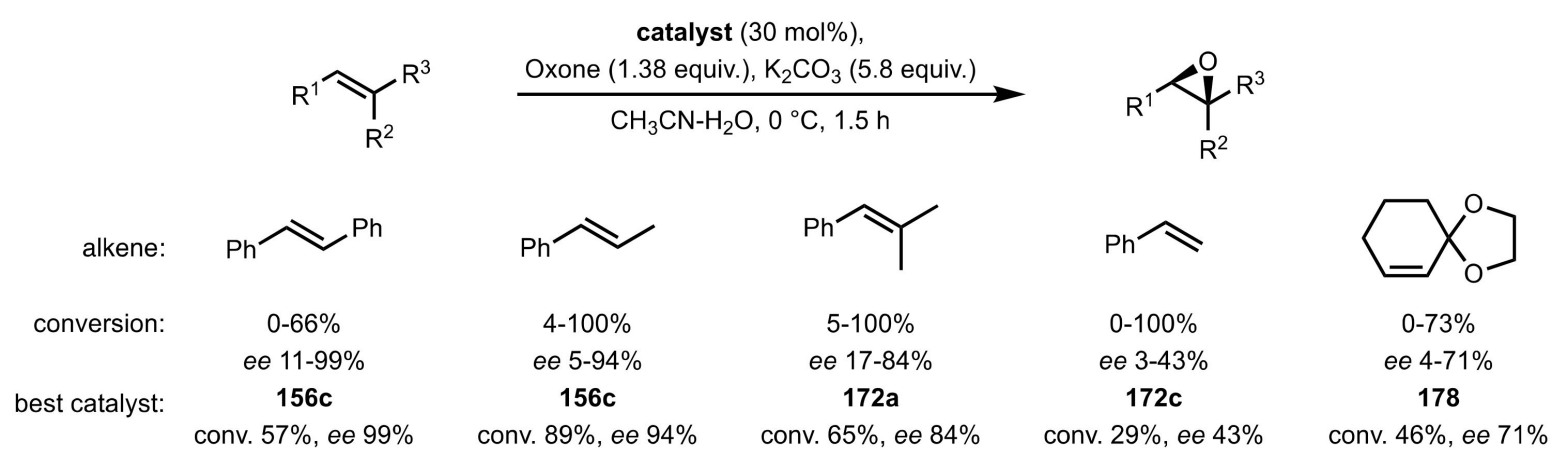
Model epoxidation reactions catalysed by the organocatalysts **156a-o**, **163**, **169a-c**, **172a-g**, **173a-c**, **176a,b**, **178**, **182** and **88**.

Although most of the new organocatalysts **156a-o**, **163**, **169a-c**, **172a-g**, **173a-c**, **176a-b**, **178**, **182** [85] as well as the ketone **88** (see Scheme 19) derived from 1,2:5,6-di-*O*-isopropylidene-d-glucofuranose displayed good results for the epoxidation of *trans*- and trisubstituted alkenes, no notable increase in efficiency for terminal and *cis*-alkenes was observed (Scheme 43).

##### 3.1.1.2. Epoxidation of *Cis*-Alkenes

Synthesis of nitrogen containing sugar ketones

**Scheme 44:**
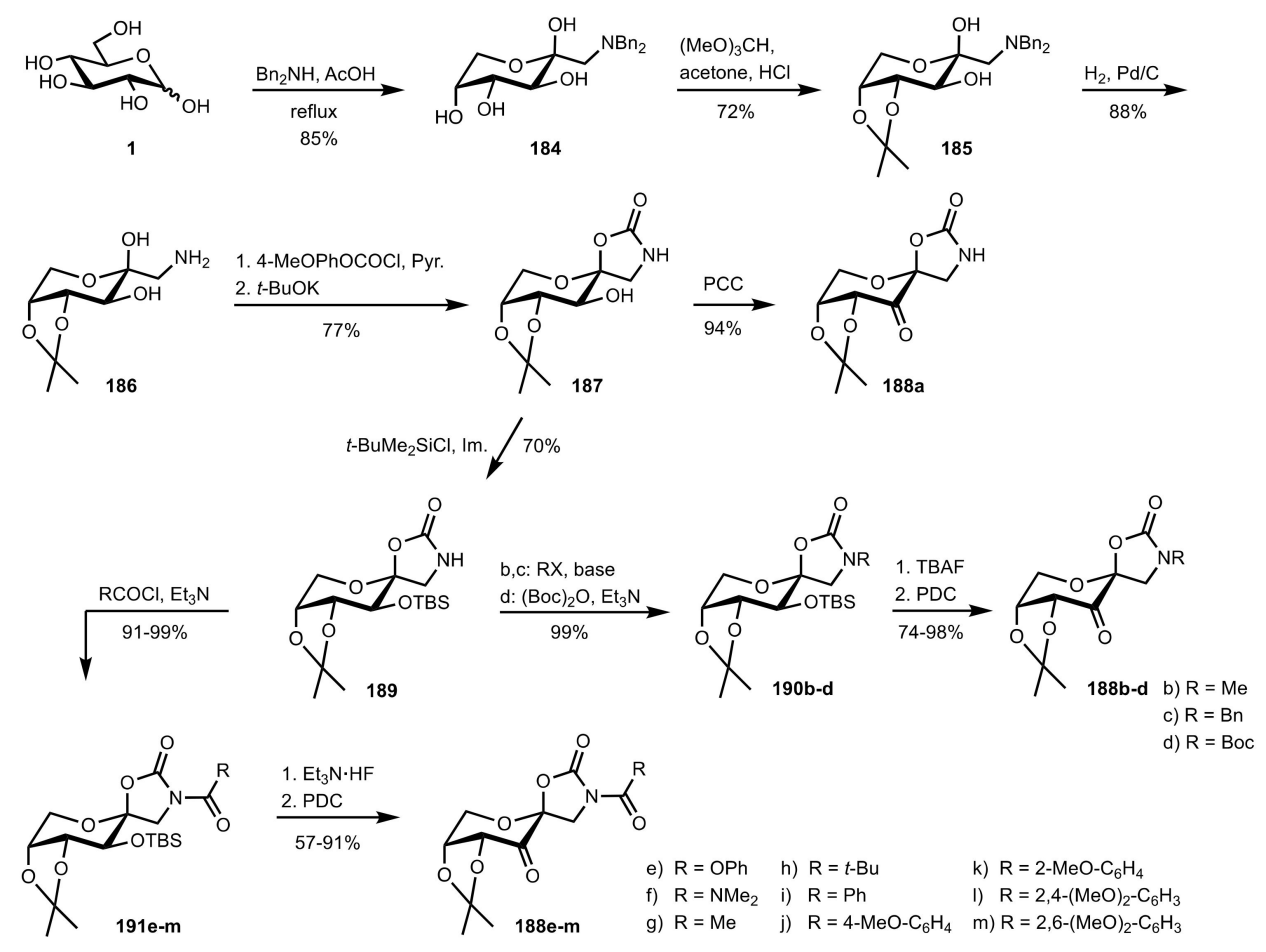
The synthesis of sugar ketone organocatalysts **188a-m** from d-glucose.

Various nitrogen-containing analogues of the sugar ketone organocatalysts **156a-o** and **172a-g** were synthesised and tested in asymmetric epoxidation reactions [98,99,100]. The treatment of d-glucose with dibenzylamine at high temperature led to the Amadori product **184**, which, after protection of the vicinal hydroxy groups and hydrogenolysis, gave the amine **186** (Scheme 44).

The latter was transformed into the oxazolidinone **187** and then oxidised with pyridinium chlorochromate (PCC) to afford **188a** [99,100]. Silylation of **187** and *N*-alkylation or Boc-protection led to **190b-d**. Upon desilylation of the secondary alcohol and subsequent oxidation with pyridinium dichromate (PDC), the organocatalysts **188b-d** were obtained [98,99,100,101]. The sugar ketones **188e-m** were obtained by *N*-acylation of **189**, desilylation and oxidation (Scheme 44) [100].

**Scheme 45:**
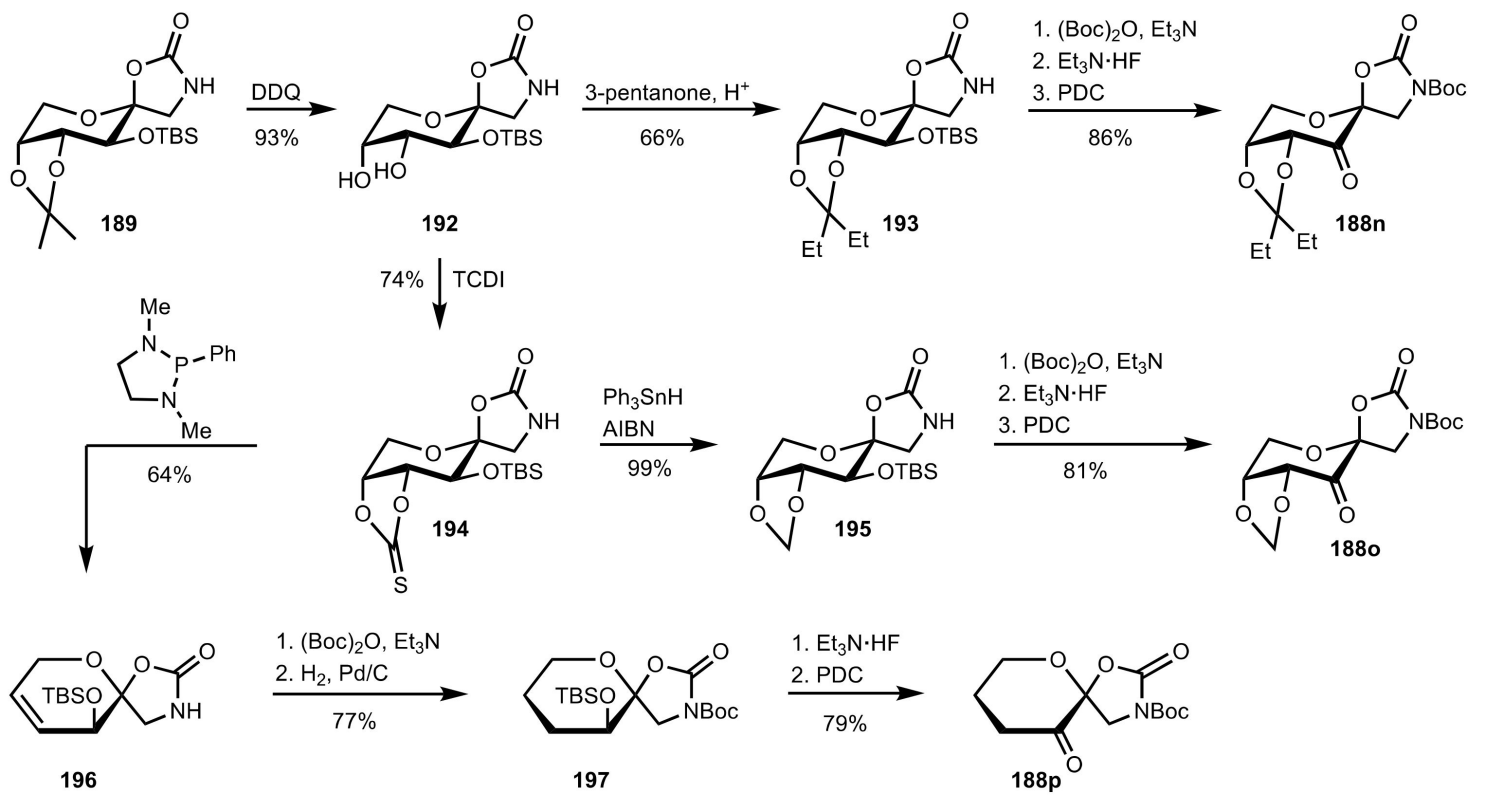
The synthesis of sugar ketone organocatalysts **188n-p**.

Organocatalyst **188n** was synthesised from **189** by isopropylidene removal with DDQ to give **192** and ketalization with 3-pentanone, followed by protection of the nitrogen atom, desilylation and oxidation of the hydroxy group (Scheme 45). The diol **192** was treated with 1,1′-thiocarbonyldiimidazole (TCDI) to form the 1,3-dioxane-2-thione **194** that was reduced to **195** by reaction with triphenyltin hydride in the presence of the radical initiator azobisisobutyronitrile (AIBN).

Compound **195** was protected as an *N*-Boc derivative, desilylated and oxidised to give the ketone **188o** (Scheme 45). The organocatalyst **188p**, an analogue of **164**, was obtained from **194** [100] by treatment with an excess of 1,3-dimethyl-2-phenyl-1,3,2-diaza-phospholidine [102], protection of the nitrogen atom, hydrogenation of the double bond and desilylation followed by oxidation with pyridinium dichromate (PDC) (Scheme 45).

**Scheme 46:**
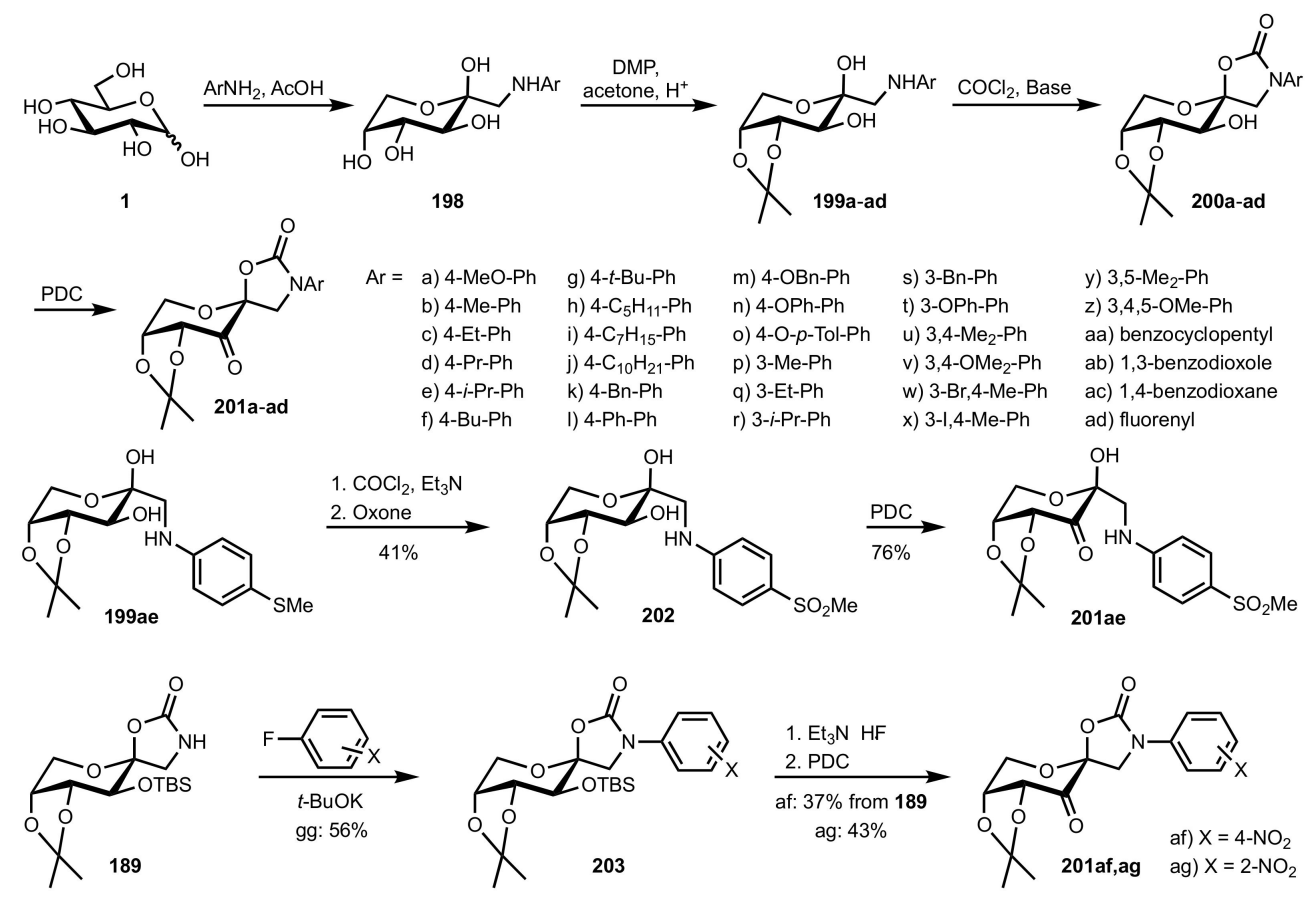
The synthesis of sugar ketone organocatalysts **201a-ag** from d-glucose.

Contrary to the above-mentioned synthetic approach leading to the organocatalysts **188a-o**, in the case of the ketones **201a-ee,** the aryl moieties were directly inserted during the Amadori rearrangement (Scheme 46) [103,104]. Then, ketalization, cyclisation and oxidation allowed obtaining **201a-ee** (for **201a,d-ad** the yields were not reported). Later on, the synthesis of **201b,c** was improved and made more convenient for large scale production [105].

Major changes involved the isolation of **199** as hydrogensulfate salt and use of 2,2,6,6-tetramethylpiperidine 1-oxyl radical (TEMPO) and NaOCl instead of pyridinium dichromate (PDC) as the oxidant. Upon treatment with Oxone, the thioether **199ae** was oxidised to the sulfone **202**, which was then oxidised to the ketone **201ae** (Scheme 46). The organocatalysts **201af,ag** were synthesised by *N*-arylation of **189** through aromatic nucleophilic substitution, desilylation and oxidation (Scheme 46).

Asymmetric epoxidation of *cis*-alkenes

**Scheme 47:**
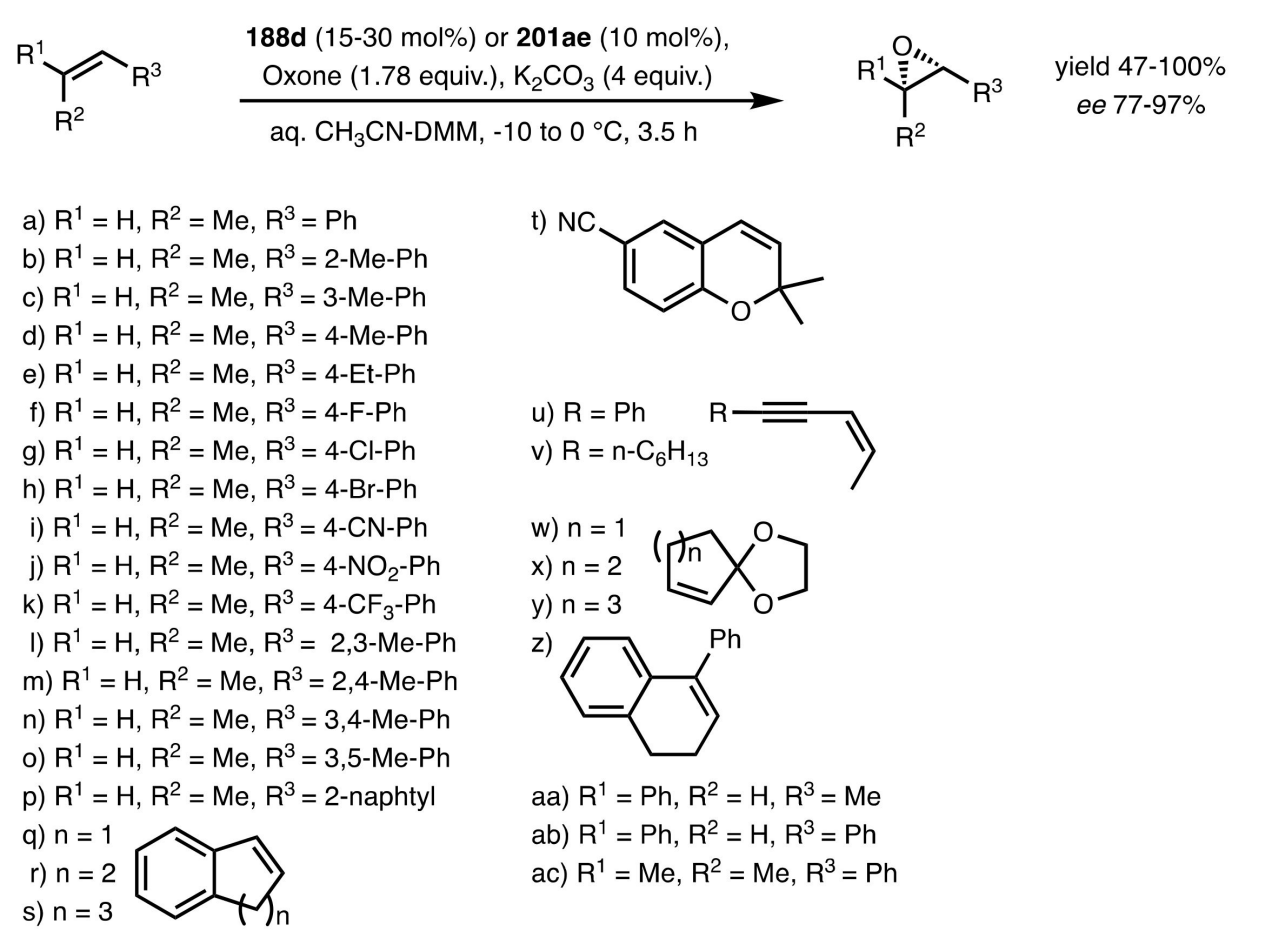
The epoxidation of alkenes (mainly *cis*) catalysed by **188d** or **201ae**.

The catalysts **188a-p** and **201a-ag** were employed for the asymmetric epoxidation of a large number of di- and trisubstituted alkenes (mainly *cis*-alkenes, see substrates **a–y**, Scheme 47), [98,100,103,106] as well as terminal alkenes (see Section 3.1.1.3) [99,100,103]. The previously synthesised organocatalysts were not able to perform the reaction in case of *cis*- and terminal alkenes, whereas the catalytic properties of **188d** and (partially) **201b,c,f,l,ae** were found to be strikingly different [98].

**Scheme 48:**
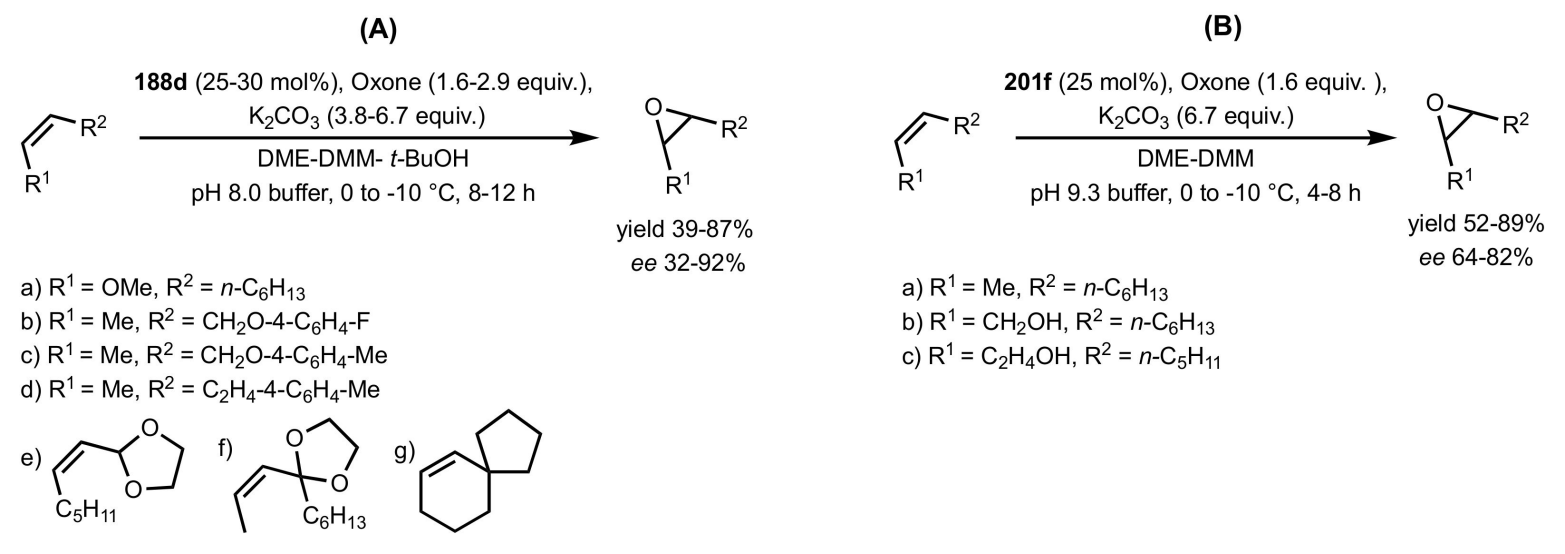
The epoxidation of non-conjugated alkenes catalysed by **188d** (**A**) and **201f** (**B**). DME = dimethoxyethane; DMM = dimethoxymethane.

To better understand the stereodifferentiation factors, Shi and co-workers studied the epoxidation of non-conjugated olefins catalysed by sugar ketones **188d** and **201f** [107]. The procedure did not differ from that outlined in the previous scheme with the exception of the pH value (8.0) of the reactions catalysed by **188d** (Scheme 48). Low to high yield (39–87%) and enantioselectivity (32–92% *ee*) were obtained.

Several conclusions have been drawn from the analysis of the epoxidation results [107], in particular that the enantioselectivity is mainly affected by the interactions in the transition state between the hydrophobic group of the olefin and the *N*-aryl substituent of the ketone. The *ee* values increase if the other substituent of the alkene is more hydrophilic (Scheme 48B).

In the case of ketone **188d**, allylic ethers appeared to be good substrates (71–79% yield and 61–86% *ee* for alkenes **a-c**, Scheme 48A), while their analogues lacking the *O*-allylic functionality led to epoxides with lower stereoselectivity [107]. It is likely that these results are due to attractions between electron lone pairs of substrates’ oxygen atom and oxazolidinones. On the other hand, they may be caused by repulsions between the lone pairs of oxygen in the alkene and the fused ketal of the sugar ketone catalyst.

Epoxidation of conjugated *cis*-dienes

**Scheme 49:**
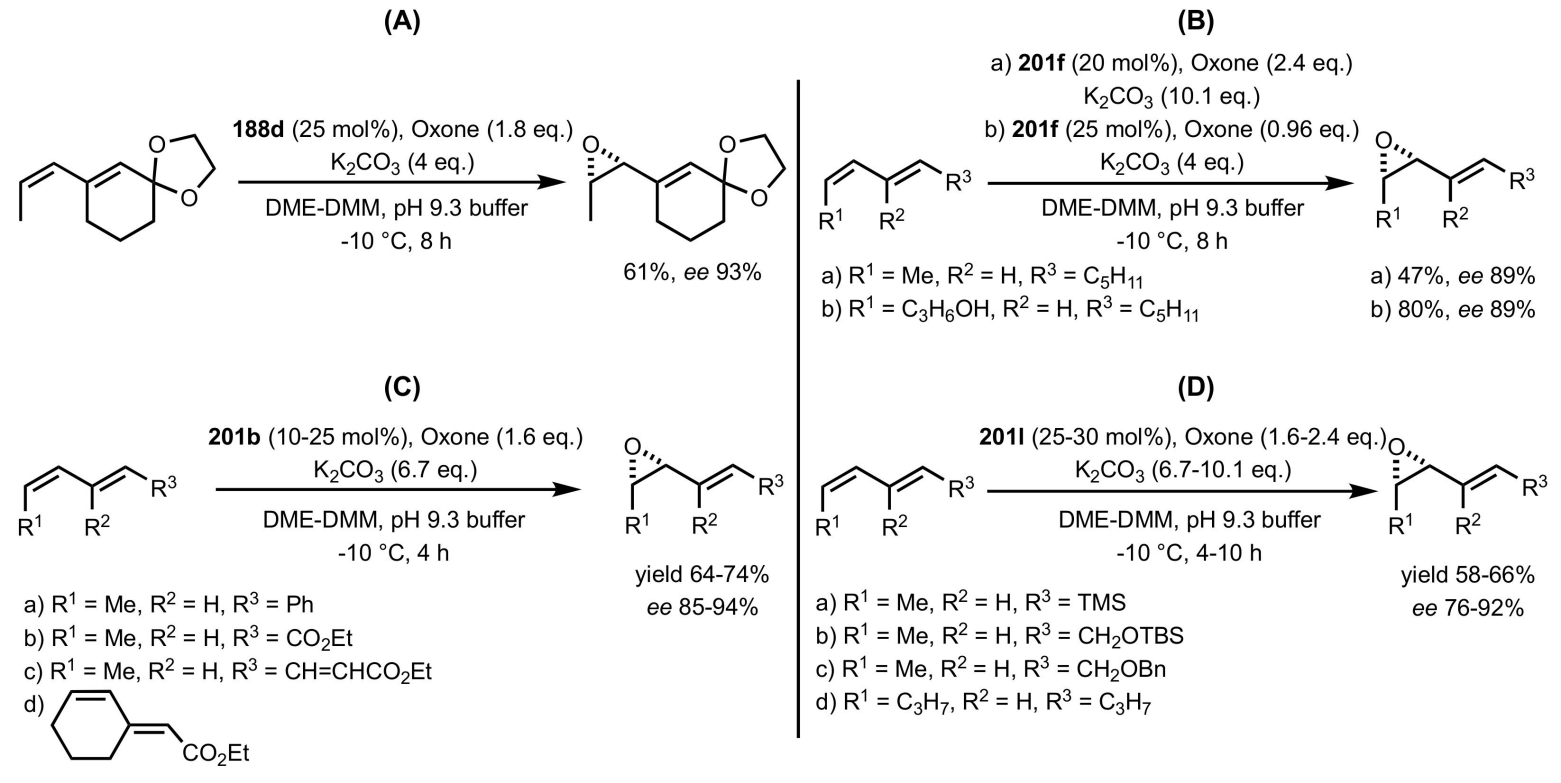
The epoxidation of conjugated dienes catalysed by **188d** (**A**), **201f** (**B**), **201b** (**C**) and **201l** (**D**).

Shi and co-workers developed an efficient synthesis of chiral vinyl epoxides [108,109] from a large number of conjugated dienes using glucose-derived ketones **188d**, **201b,f,l** as organocatalysts in 3:1 DME-DMM (Scheme 49) [110]. For each catalyst, the loading (10–30%), amount of Oxone and potassium carbonate (1.6–2.4 equiv., 4.0–10.1 equiv., respectively) and reaction time (4–10 h) were optimized. The reaction was found to be regioselective and stereospecific (*cis*-epoxides were obtained from *cis*-olefins), and further epoxidation of products was not observed.

The enantioselectivity depends on the character of the substituent in the aromatic ring of catalyst as well as on the hydrophilic vs. hydrophobic character of the substituent of the diene. A decrease of enantioselectivity was noticed for more hydrophobic R^1^ groups (Scheme 49), probably due to hydrophobic interactions between R^1^ and the substituent on the nitrogen atom of the catalyst. Consequently, it can be expected that the hydrophilic character of R^1^ and/or the hydrophobic R^3^ will increase the *ee* values.

Epoxidation of *cis*-enynes

**Scheme 50:**
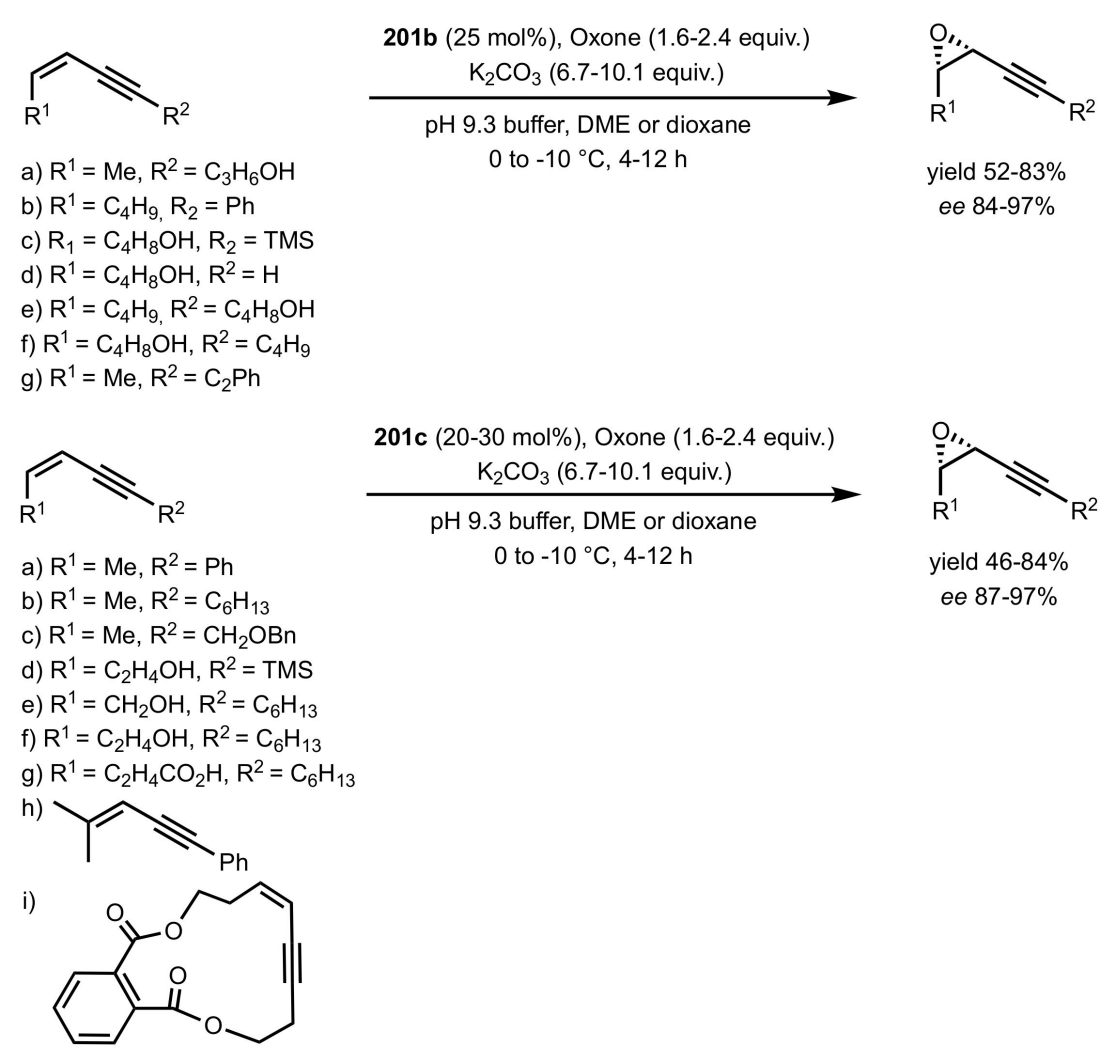
The epoxidation of conjugated *cis*-enynes by sugar ketones **201b,c**.

*Cis*-propargyl epoxides were also synthesised by the same researchers [111]. To expand the substrate scope, catalysts **201b,c** were used in place of **156a**. Enynes, prepared through Sonogashira coupling of vinyl halides and alkynes, were submitted to standard epoxidation in the presence of glucose-derived ketones **201b,c** as the organocatalysts. The corresponding epoxides were obtained in good to high yield (52–83% and 46–84%, respectively) and high to excellent enantioselectivity (84–97% and 87–97% *ee*, respectively) (Scheme 50).

The enantioselectivity was strongly dependent on the substituents in olefin (R^1^) and alkyne (R^2^) since more hydrophobic R^1^ group resulted in lower *ee* values. Enantioselectivity was higher in the case of increased polarity of R^1^ and lowered polarity of R^2^. Moreover, it was observed that catalysts **201b,c** were more enantioselective than **156a** in the epoxidation of certain trisubstituted enynes as well.

**Scheme 51:**
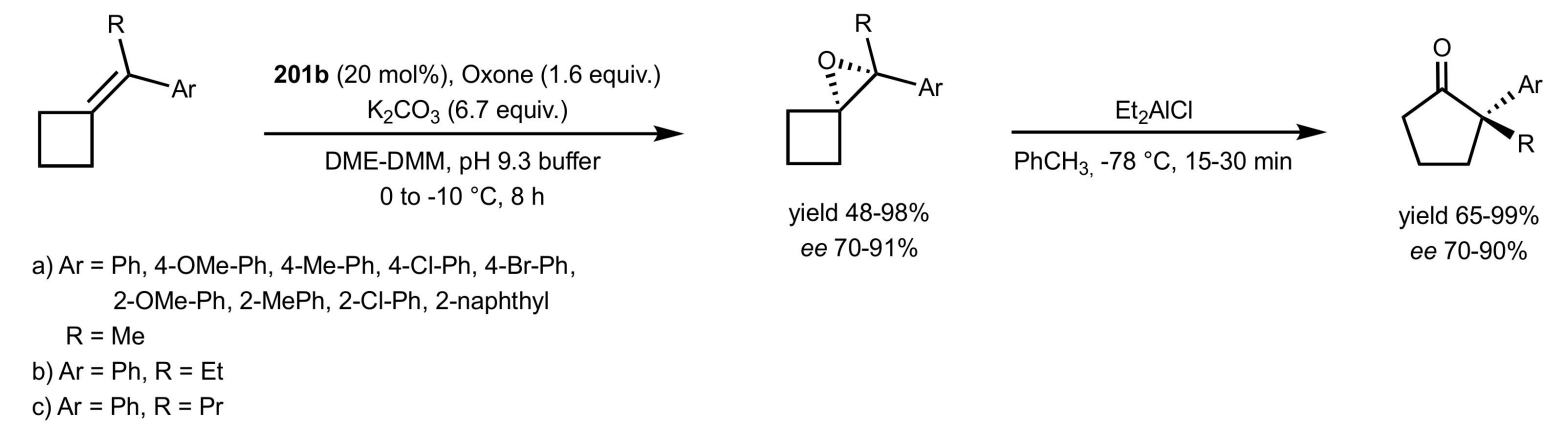
The epoxidation of 1-cyclobutylidene-1-phenylethane derivatives catalysed by **201b** and epoxide rearrangement.

Shi and co-workers expanded the substrate scope by using tetra-substituted benzylidene-cyclobutane derivatives that were efficiently epoxidized by **201b** to give, after epoxide rearrangement, 2-alkyl-2-arylcyclopentanones with moderate to excellent yield (46–98%) and generally high enantioselectivity (70–91% *ee*) (Scheme 51) [112].

##### 3.1.1.3. Epoxidation of Terminal Alkenes

**Scheme 52:**
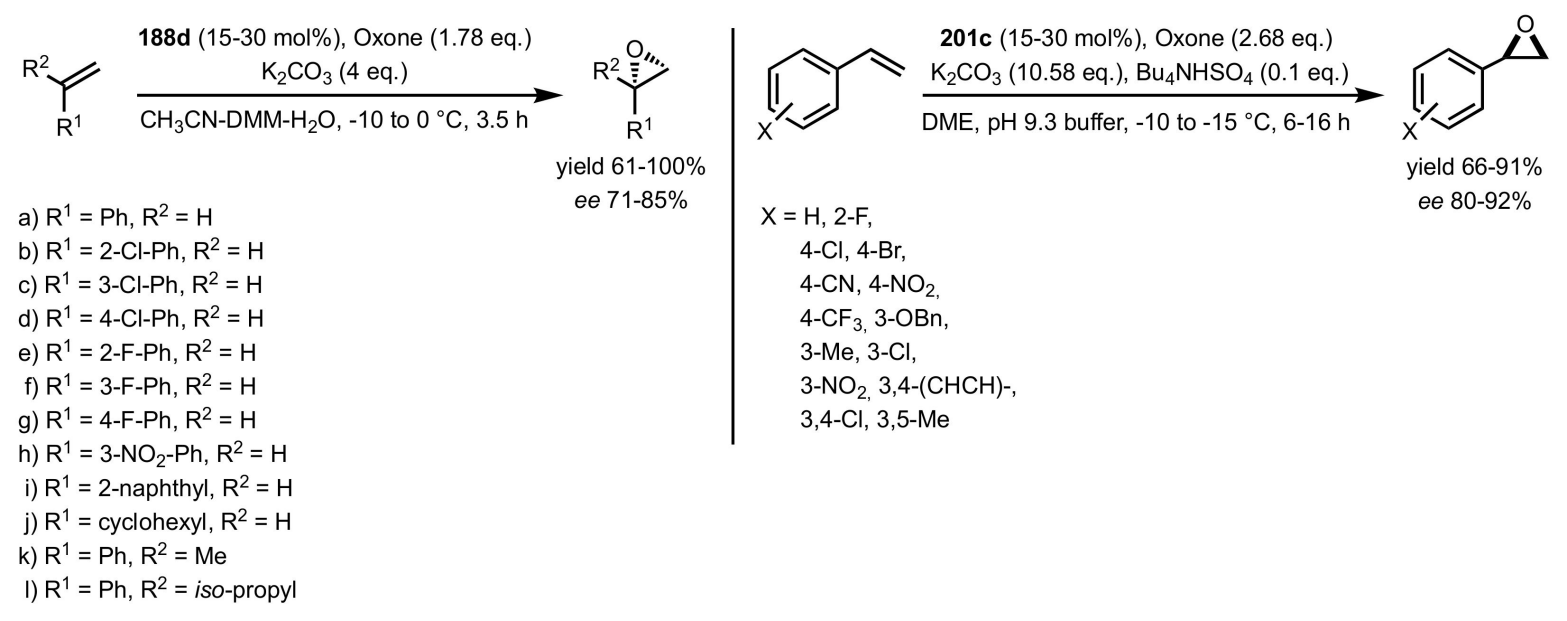
The epoxidation of terminal alkenes and styrenes catalysed by **188d** and **201c**.

The organocatalysts **188a-p** and **201a-ag**, which proved to be efficient in the asymmetric epoxidation of *cis*-alkenes, were also successfully employed for the epoxidation of various terminal alkenes, and the best results were obtained with the sugar ketones **188d** and **201c** (Scheme 52) [99,100,103,104].

**Scheme 53:**
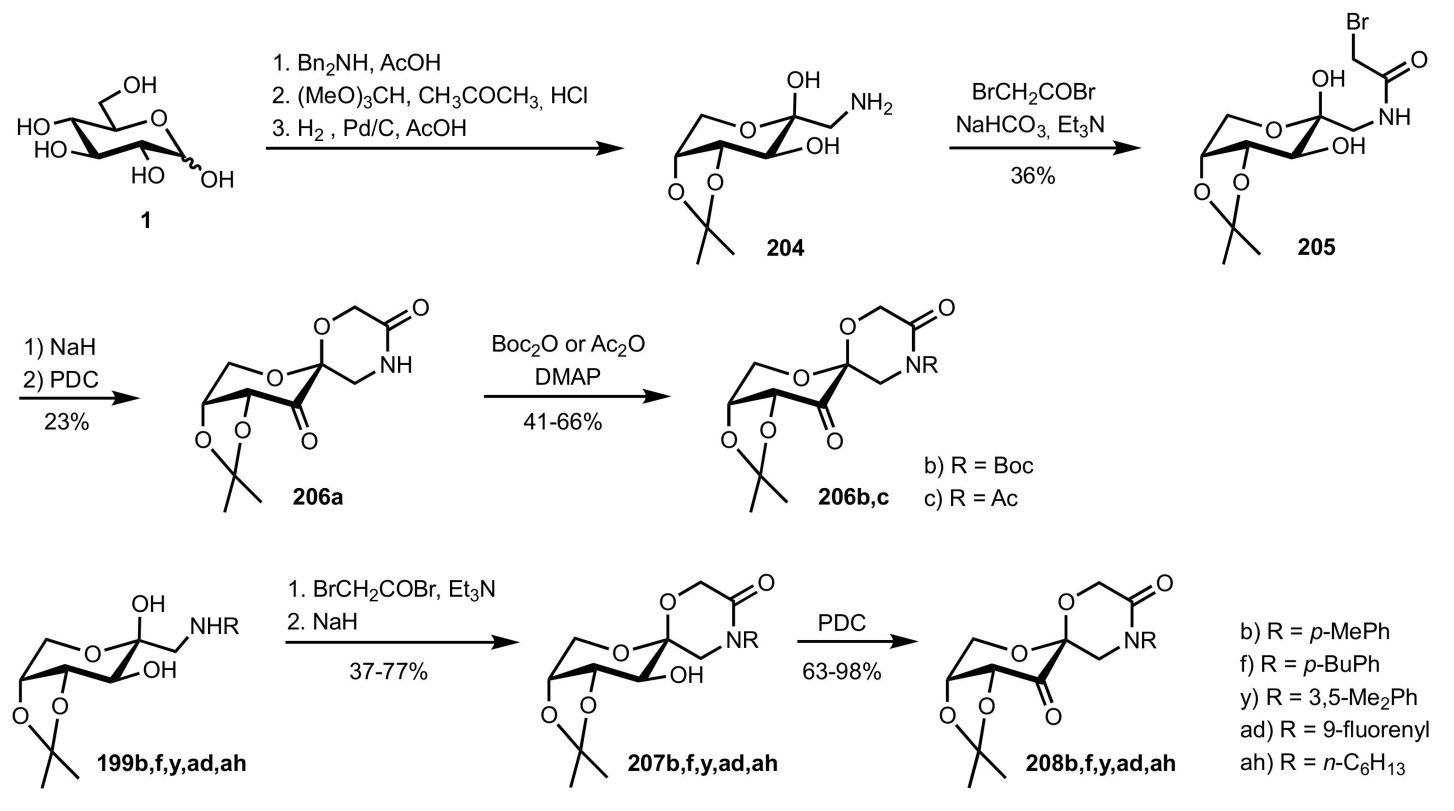
The synthesis of sugar ketone organocatalysts **206a-c** and **208b,f,y,ad,ah**.

Since the efficiency of the epoxidation of terminal olefins catalysed by the above-mentioned sugar ketones was not satisfying, Shi and co-workers prepared another series of organocatalysts [113]. Diol **204**, prepared from d-glucose as previously described [103], was amidated with 2-bromoacetyl bromide under basic conditions (Scheme 53) to give **205**, which was cyclized in the presence of NaH and then oxidised to afford the lactam ketone **206a** in low yield. Ketones **206b,c** were easily obtained by treating **206a** with an excess of Boc anhydride or acetic anhydride and catalytic amounts of DMAP. The *N*-alkylated sugar ketones **208** were synthesised starting from the previously reported [105] amino diols **199b,f,y,ad** (see Scheme 46) and the new derivative **199ah** as described for the synthesis of the lactam **206a** (Scheme 53).

**Scheme 54:**
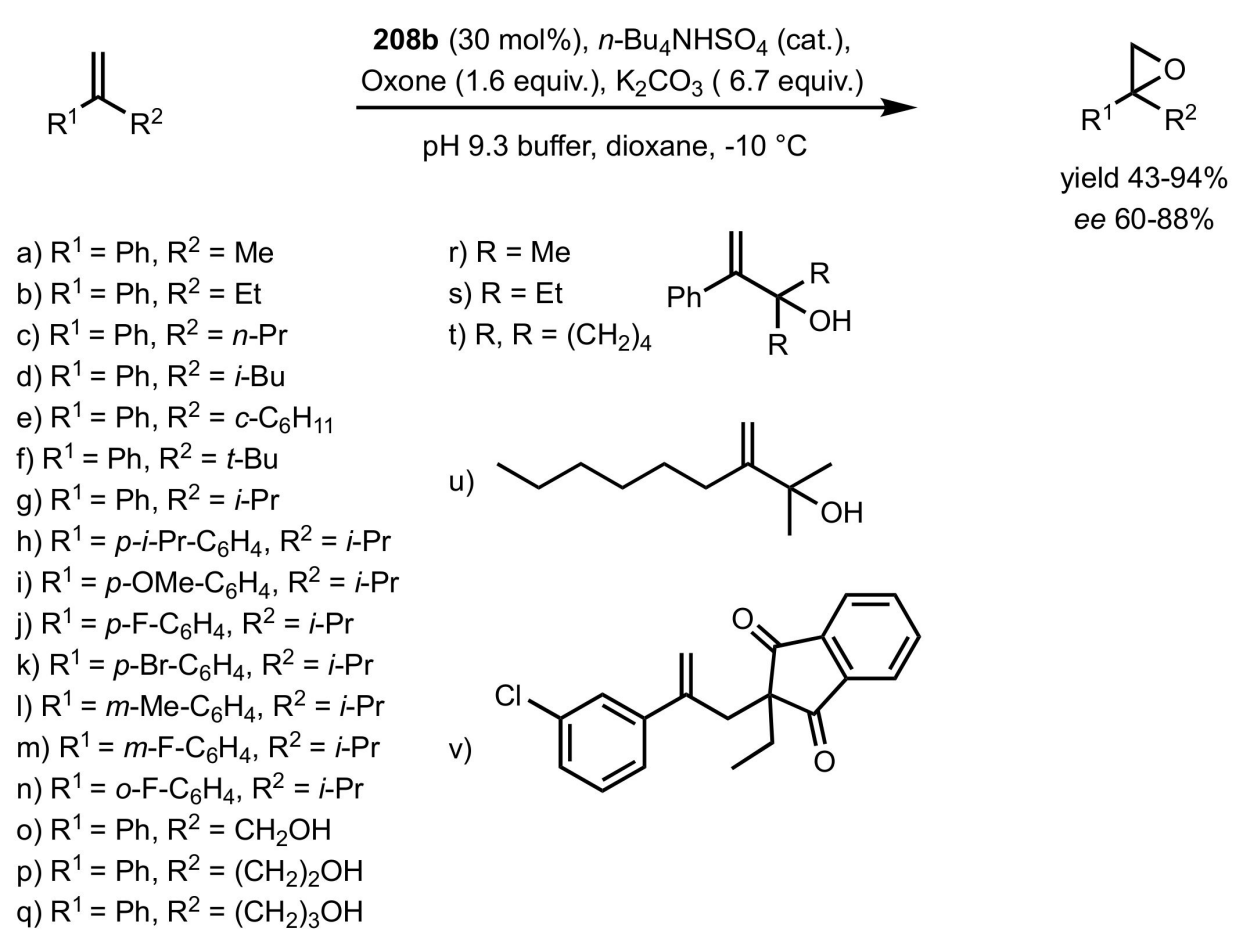
The epoxidation of 1,1-disubstituted terminal olefins catalysed by **208b**.

The preliminary epoxidation of α-isopropylstyrene catalysed by the sugar ketones **206** and **208** (see Scheme 53) showed that **208b** was one of the most efficient catalysts (94% yield and 84% *ee*); therefore, it was subjected to further investigations (Scheme 54). Interestingly, X-ray studies indicated that the carbonyl group and the *N*-phenyl group of **208b** are not coplanar as it was observed for its analogue **201b** (see Scheme 46). The epoxidation of a set of twenty-two 1,1-disubstituted alkenes by **208b** allowed establishing that substrates bearing bulkier alkyl substituents in the α-position led to higher enantioselectivity. Allylic and (bis)homoallylic alcohols turned out to be suitable substrates affording epoxides with up to 88% *ee* (with various configurations).

Since the absolute configuration of some products was known, it could be inferred that epoxidation of 1,1-disubstituted terminal olefins proceeds via planar transition state (see Figure 20). Additionally, the presence of bulky alkyl group on the alkene disfavors another competing spiro transition state resulting in higher enantioselectivity. The epoxidation of *cis*-olefins with **208b** led to results similar to those observed for **201b**, revealing attractions between amide group in carbohydrate-based catalyst and π-electrons in aryl group of the substrate in the spiro transition state (Figure 20). In turn, trisubstituted olefins are epoxidated more likely through planar TS.

##### 3.1.1.4. Organocatalysts for the Epoxidation of Tri- and Tetra-Substituted Alkenes

**Scheme 55:**
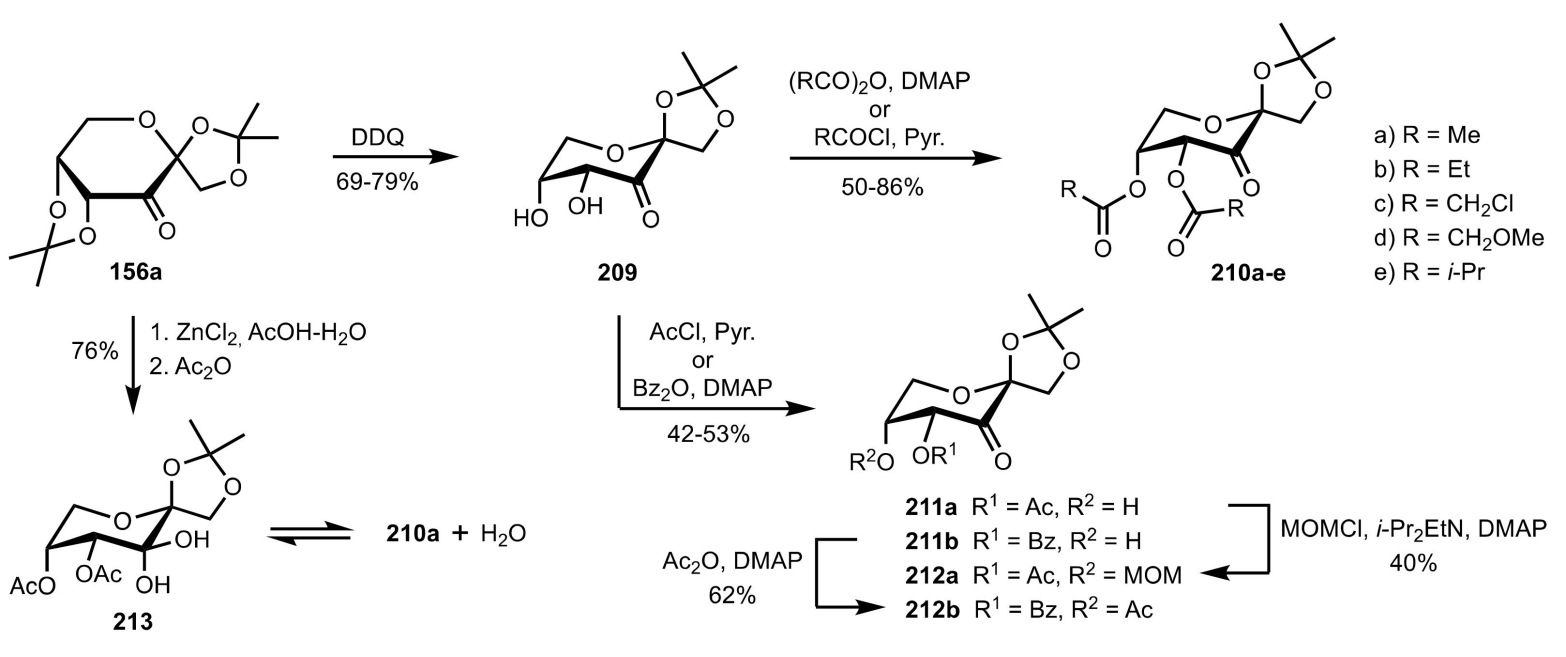
The synthesis of d-fructose-derived sugar ketones **210a-e**, **211a,b** and **212a,b**.

Following the synthesis of diacetal-ketones based on d-fructose skeleton and their application in epoxidation of alkenes [85,86,87], Shi and co-workers envisaged the preparation of a series of organocatalysts obtained by exchange of the acetal group in positions 4 and 5 of compound **156a** with one or two ester functions [114,115]. After the regioselective removal of an isopropylidene group in ketone **156a** to afford **209**, the diesters **210a-e** were easily synthesised by reaction with the appropriate anhydride or acyl chloride (Scheme 55).

The 4-acetyl (**211a**) and 4-benzoyl (**211b**) monoesters were alkylated (MOMCl, *i*-Pr_2_EtN) or acetylated, respectively, to obtain the 4,5-diprotected sugar ketones **212a** and **212b**. Finally, the diacetylated ketone **210a** could be also prepared exploiting an alternative two-step one-pot synthesis. The regioselective deketalization and acetylation of **156a** gave **213**, the hydrate form of the ketone **210a**, which, upon overnight stirring with Na_2_SO_4_ afforded the desired ketone **210a** (Scheme 55).

**Scheme 56:**
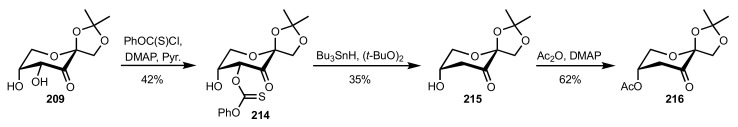
The synthesis of d-fructose-derived sugar ketones **216**.

The sugar ketone **216** was prepared by treating **209** (see Scheme 55) with *O*-phenylchlorothionoformate in the presence of base to afford **214**, which was deoxygenated under radical conditions (Bu_3_SnH and *t*-butyl peroxide) to afford **215** that was acetylated to give the 5-acetyl-ketone **216** (Scheme 56) [115].

**Scheme 57:**
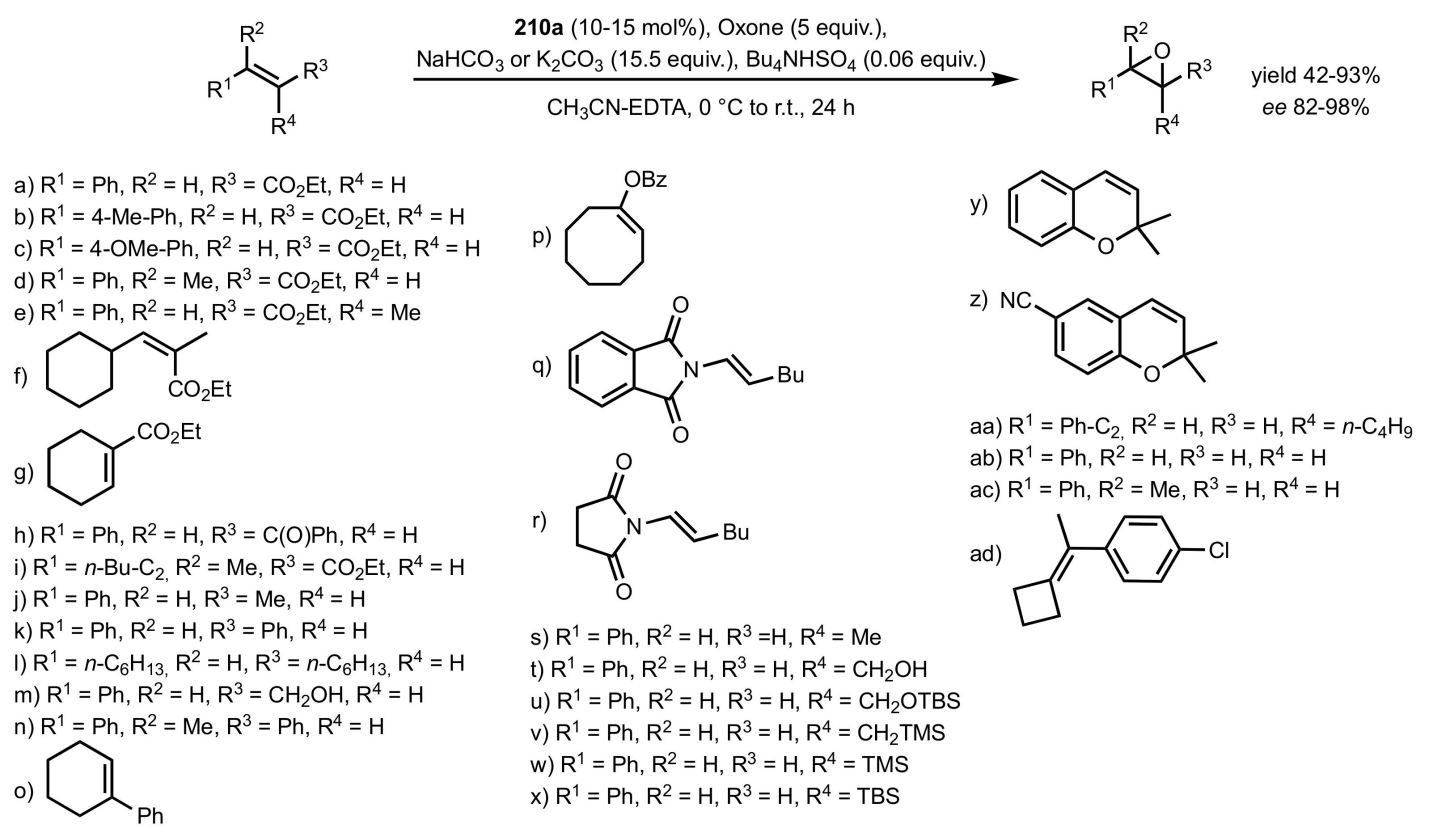
The epoxidation of alkenes catalysed by sugar ketone **210a**.

Catalysts **210a-e**, **211a**, **212a,b** and **216** were screened in asymmetric epoxidation of ethyl *trans*-cinnamate. The reactions afforded the desired epoxides with low to moderate conversions (1–47%) and low to high enantiomeric excess (29–95%), ketone **210a** being the most efficient (47% yield, 95% *ee*). Then, it was found that epoxidation of α,β-unsaturated esters catalysed by **210a** gave good results (up to 93% yield and 98% *ee*), whereas it was less effective in case of enones as the substrates (Scheme 57) [115]. Moreover, the increase of the pH from 7 to 8.75–9.50 allowed decreasing the amount of the catalyst from 20–30 to 10 mol% without a loss of activity.

Taking into consideration the epoxidation of many different olefins, including *trans*- and *cis*-alkenes, tri-, tetra-substituted and terminal alkenes, it was proven that diacetyl-ketone **210a**, although a potent organocatalyst for electron-deficient alkenes, was less efficient than the previously developed ketone **156a** and oxazolidinone ketones **188** and **201** [115]. Changing the acetyl functions in positions 4 and 5 of **210a** with other functions did not improve the efficiency of the catalysts. Therefore, for the majority of substrates (*trans*- and *cis*-alkenes, tri-, tetra-substituted and terminal alkenes), the oxazolidinone catalysts **188** were more effective.

#### 3.1.2. Achievements Reported by Shing and Co-Workers

**Scheme 58:**
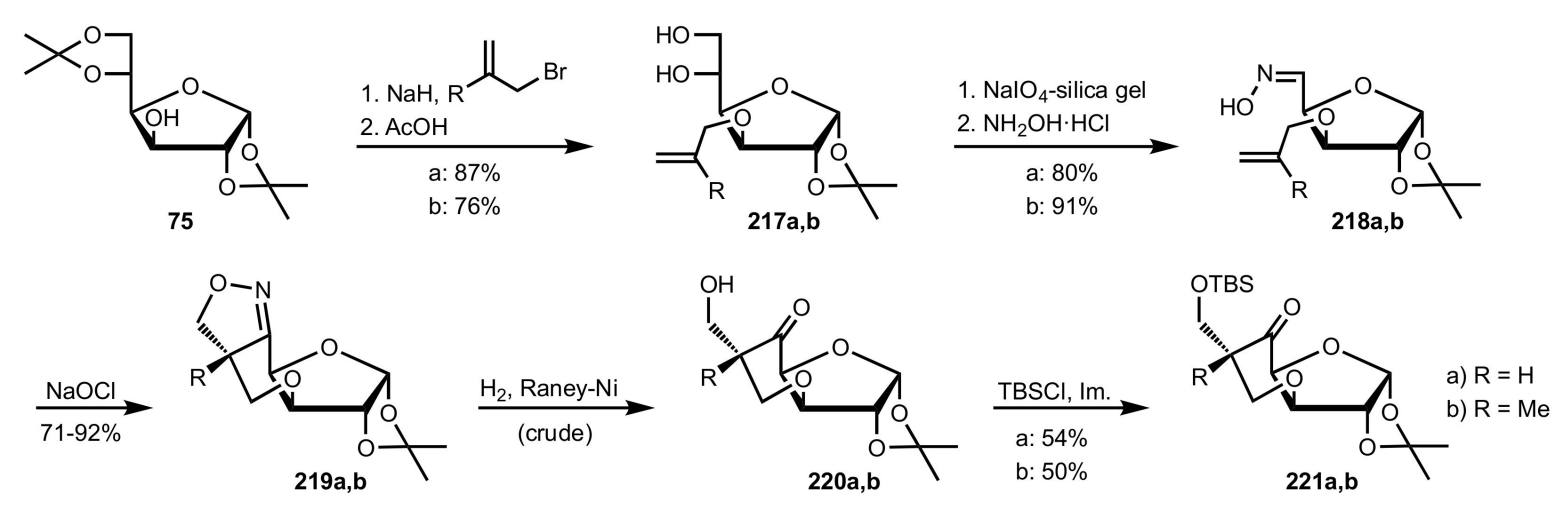
The synthesis of sugar organocatalysts **221a,b** from d-glucose diacetonide.

Nineteen novel organocatalysts were synthesised by Shing and co-workers in the 2002–2005 period starting from d-glucose [116] and l-arabinose [117,118,119]. Diacetone d-glucose **75** was first converted into the diol **217a,b** by alkylation and regioselective hydrolysis of an isopropylidene group (Scheme 58). Then, the latter was submitted to periodate oxidation, and the resulting aldehyde was transformed into the oximes **218a,b**. Upon oxidation, the transient nitrile oxide underwent intramolecular cycloaddition to give the isoxazolidines **219a,b** [120,121]. Reductive ring-opening and silylation afforded the organocatalysts **221a,b**.

**Scheme 59:**
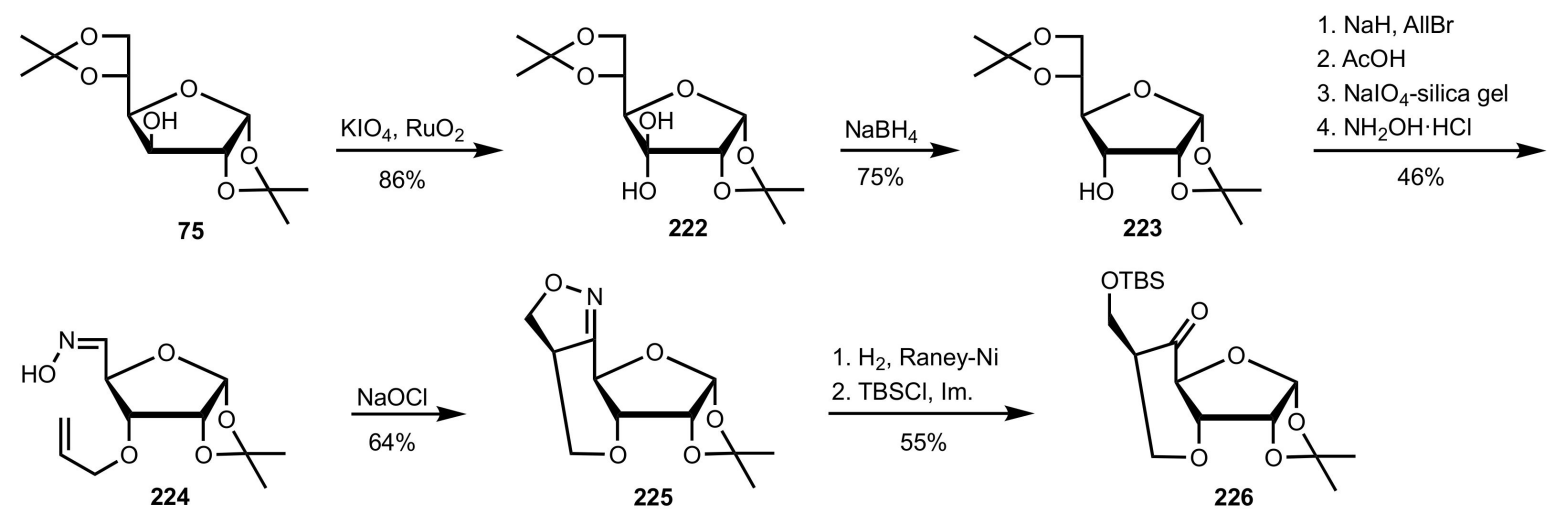
The synthesis of organocatalyst **226** from d-glucose diacetonide.

The C3-epimer of **221a**, i.e., **226** (Scheme 59), was prepared from **223** and, in turn, obtained from **75** by a two-step oxidation-reduction sequence (Scheme 59) [122]. Then, the same series of reactions outlined in the previous scheme allowed the synthesis of **226** in a satisfactory yield.

**Scheme 60:**
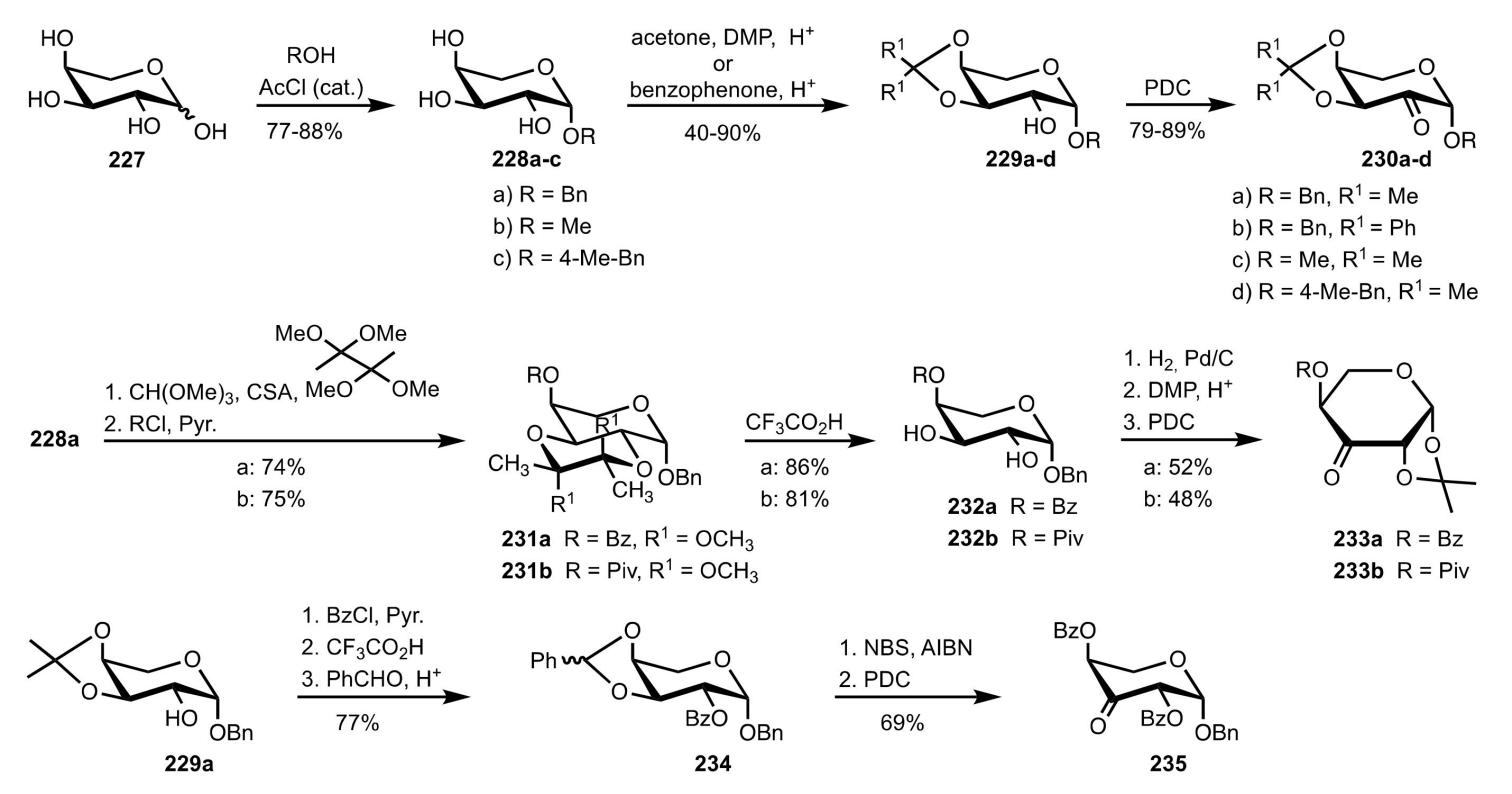
The synthesis of sugar ketone organocatalysts **230a-d**, **233a,b** and **235** from l-arabinose.

Other sugar ketone organocatalysts were synthesised from l-arabinose (**227**) (Scheme 60) through Fischer glycosidation and ketalization with acetone-2,2-dimethoxypropane or benzophenone to give the alcohols **229a-d**, which were then oxidised to the ketones **230a-d [117,118,119]**. Upon protection of the vicinal hydroxyl groups in positions 2 and 3 of **228a** using 2,2,3,3-tetramethoxybutane and trimethyl orthoformate, followed by the esterification of the remaining secondary alcohol, the bicyclic compounds **231a,b** were obtained.

Treatment of the latter with trifluoracetic acid afforded **232a,b**, which were submitted to hydrogenation, ketalization and oxidation to give the 3-keto-sugars **233a,b** (Scheme 60) [118]. Benzoylation of the secondary alcohol in **229a**, followed by removal of the isopropylidene protecting group and formation of a 3,4-*O*-benzylidene afforded **234**. A regioselective radical ring opening and oxidation of the resulting 3-OH function led to the sugar ketone **235** (Scheme 60) [118].

**Scheme 61:**
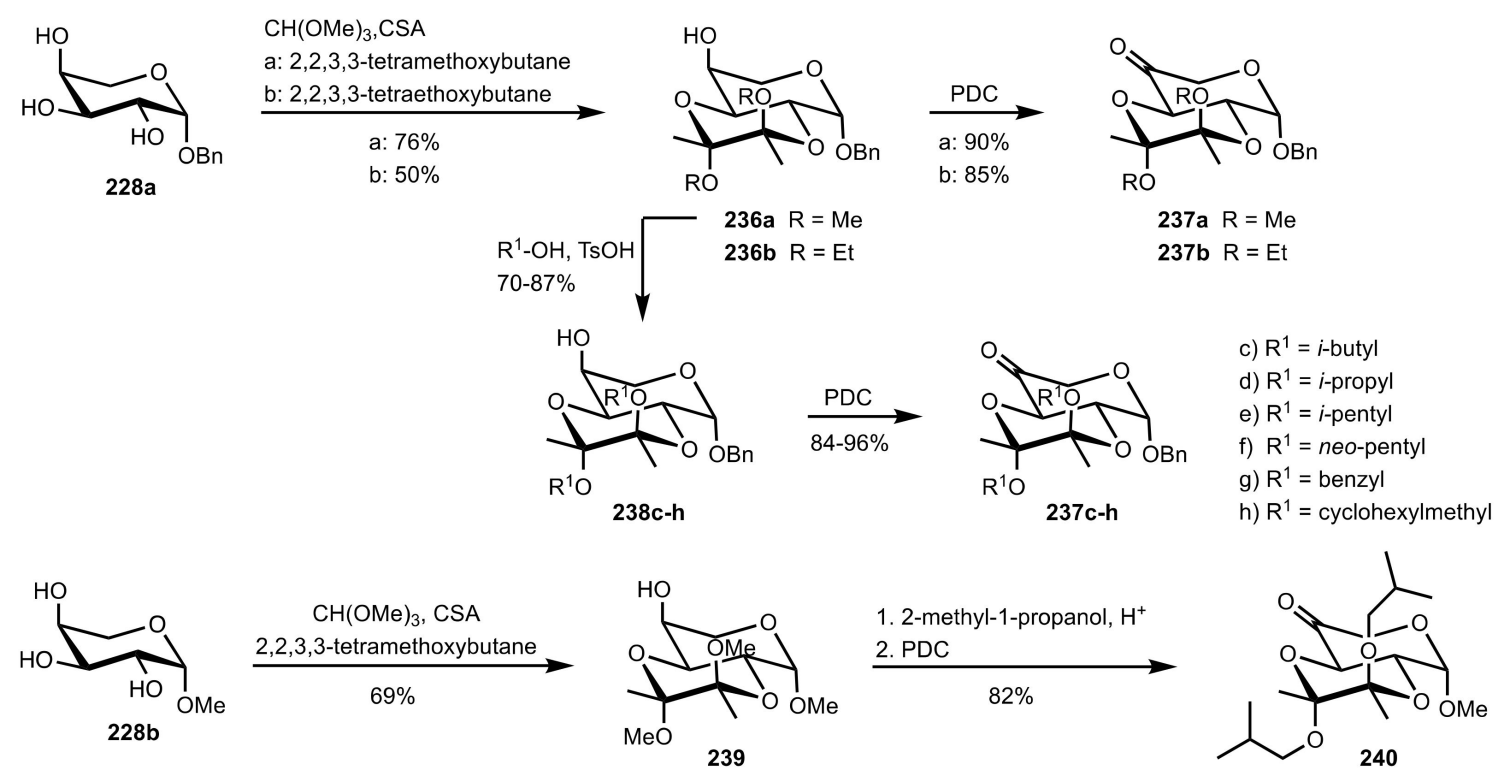
The synthesis of sugar ketone organocatalysts **237a-h** and **240**.

The same benzyl l-arabinoside **228a** was also employed for the synthesis of **237a,b** (Scheme 61). Treatment of **228a** with either 2,2,3,3-tetramethoxybutane (a) or 2,2,3,3-tetraethoxybutane (b) and trimethyl orthoformate, followed by oxidation of the resulting secondary alcohols **236a,b** gave **237a,b** [117]. The transketalization of **236a** with various alcohols in the presence of *p*-toluensulfonic acid afforded **238c-h**, which were oxidised with pyridinium dichromate (PDC) to give the organocatalysts **237c-h,** whereas the sugar ketone **240** was synthesised in a similar way starting from the methyl l-arabinoside **228b** (Scheme 61) [119].

**Scheme 62:**
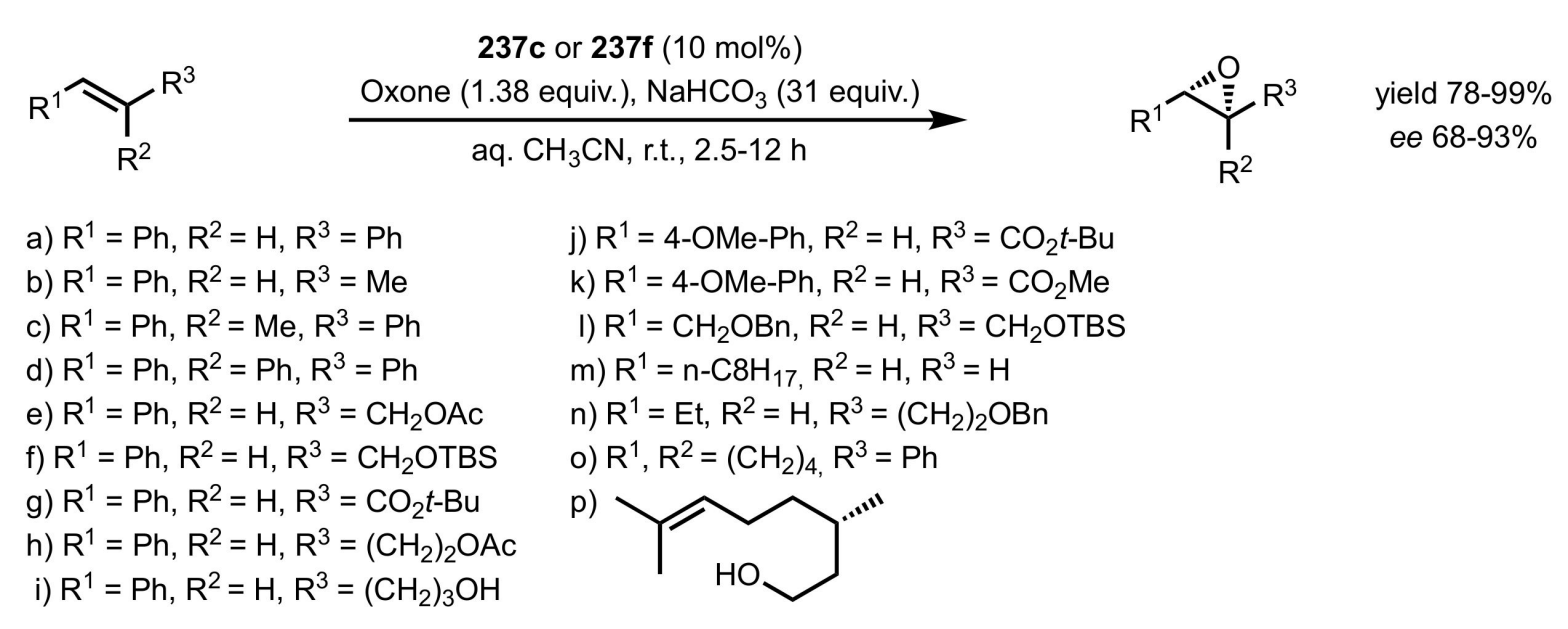
The epoxidation of alkenes catalysed by **237c** and **237f** [116,117,118,119].

The asymmetric epoxidation of various alkenes using catalysts **230a-d**, **233a,b**, **235**, **237a-h** and **240** led to good to high yields but mediocre enantiomeric excess. The catalysts **237c** and **237f** were the most powerful of the series (92% yield and 93% *ee*, 96% yield and 88% *ee*, respectively) (Scheme 62) [116,117,118,119], but still not as efficient as the catalysts developed by Shi and co-workers (see Section 3.1.1).

**Scheme 63:**
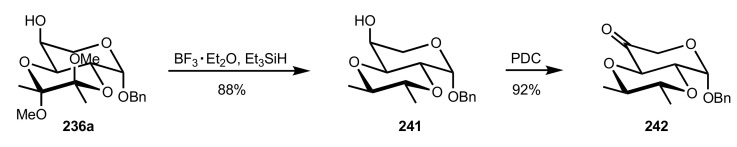
The synthesis of the arabinose-derived ketone **242**.

In 2006, the scope of the alkene epoxidation by arabinose-derived organocatalysts was studied by using more olefins, in particular the ethyl *cis*-cinnamate, and preparing an additional sugar ketone [123]. The organocatalyst **242**, featuring a dimethyl substituted dioxane moiety instead of a diacetal function, was obtained in high yield from **236a** (see Scheme 61) by hydride reduction in the presence of BF_3_·Et_2_O and standard oxidation (Scheme 63).

**Scheme 64:**
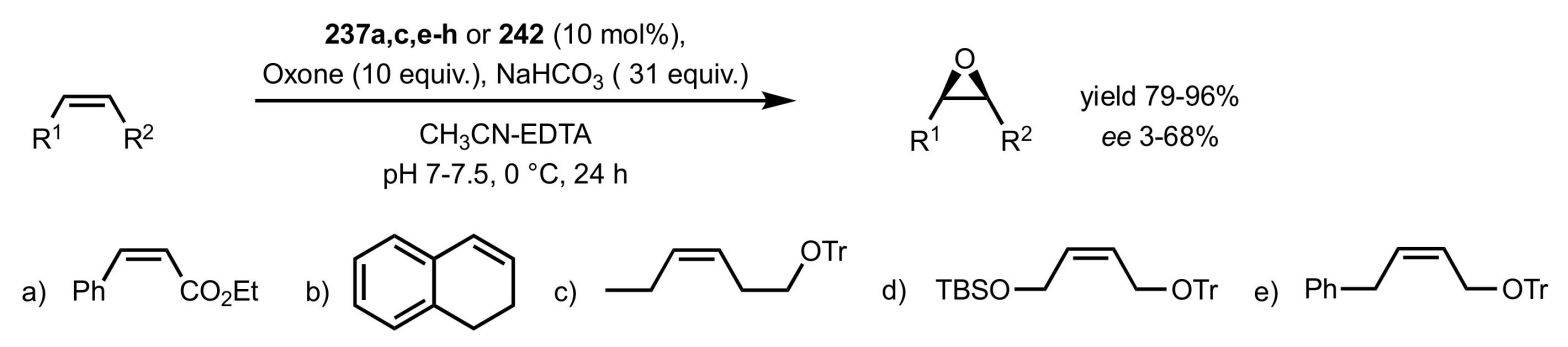
The epoxidation of *cis*-alkenes catalysed by **237a,c,e-h** and **242**.

Epoxidation of ethyl *cis*-cinnamate catalysed by **237a,c,e-h** and **245** led to the products in high yield (79–95%) but moderate (36–68%) enantioselectivity (Scheme 64) [123]. Changing acetonitrile to 1,4-dioxane or *t*-BuOH in the reaction catalysed by ketone **237a** decreased the enantiomeric excess. It is worth noting that the epoxidation of ethyl *cis*-cinnamate afforded ethyl (2*R*,3*R*)-3-phenylglycidate, which was then used as a starting material to prepare a protected Taxol side chain [123].

Surprisingly, the catalyst **237f** with the bulky *n*-pentoxy substituent turned out to be the least efficient for chirality induction, whereas for the epoxidation of *trans*-disubstituted and trisubstituted olefins the same ketone gave the best results [119]. The epoxidation assays proved that ketone **237a**, bearing the smallest group, was the most efficient (68% *ee*). Epoxidation of alkenes **b-e** (Scheme 64) in the presence of the organocatalysts **237a,f,g** and **242** took place with high yield (80–95%) and poor enantioselectivity (3–33% *ee*) [123].

**Scheme 65:**
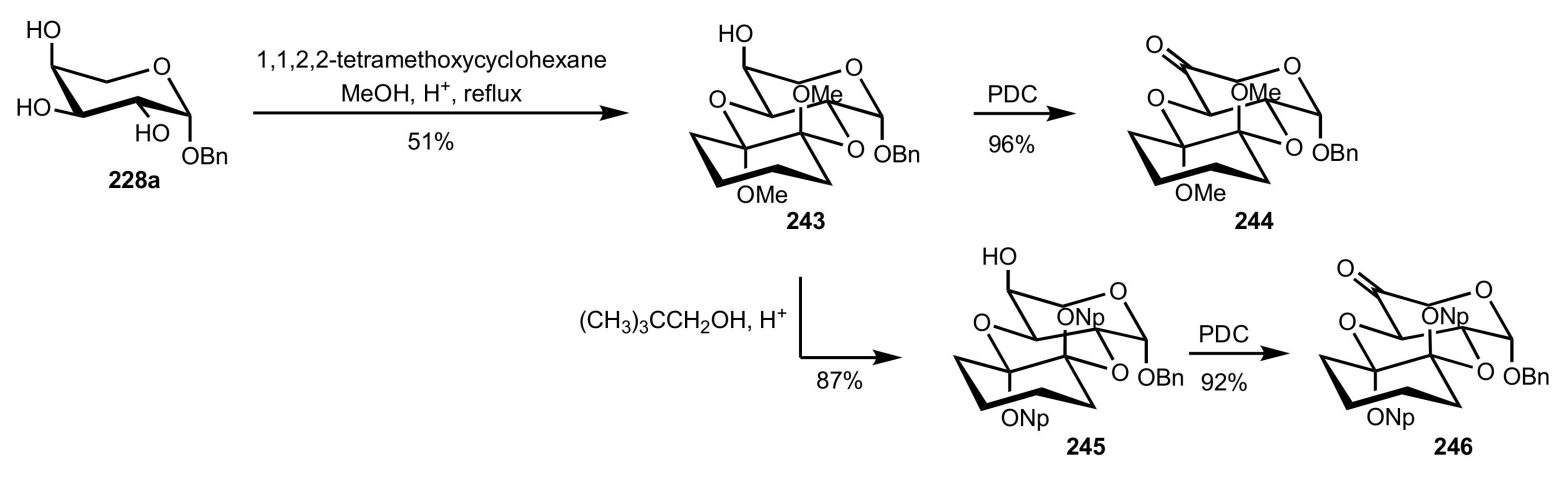
The synthesis of the arabinose-based ketones **244** and **246**.

Later, Shing and co-workers envisaged the synthesis of the sugar ketones **244** and **246** (Scheme 65) in order to study the influence of cyclic and acyclic diacetal protecting groups in the organocatalysts on epoxidation of various alkenes [124]. Benzyl arabinopyranoside **228a** (see Scheme 60) was treated with 1,1,2,2-tetramethoxycyclohexane instead of 2,2,3,3-tetratetramethoxybutane to give **243,** which was oxidised to ketone **244** (Scheme 65). To synthesize **246**, the exchange of methyl with neopentyl groups on the intermediate **243** was performed to afford **245**, which, upon oxidation with pyridinium dichromate (PDC), gave the ketone **246**.

**Scheme 66:**
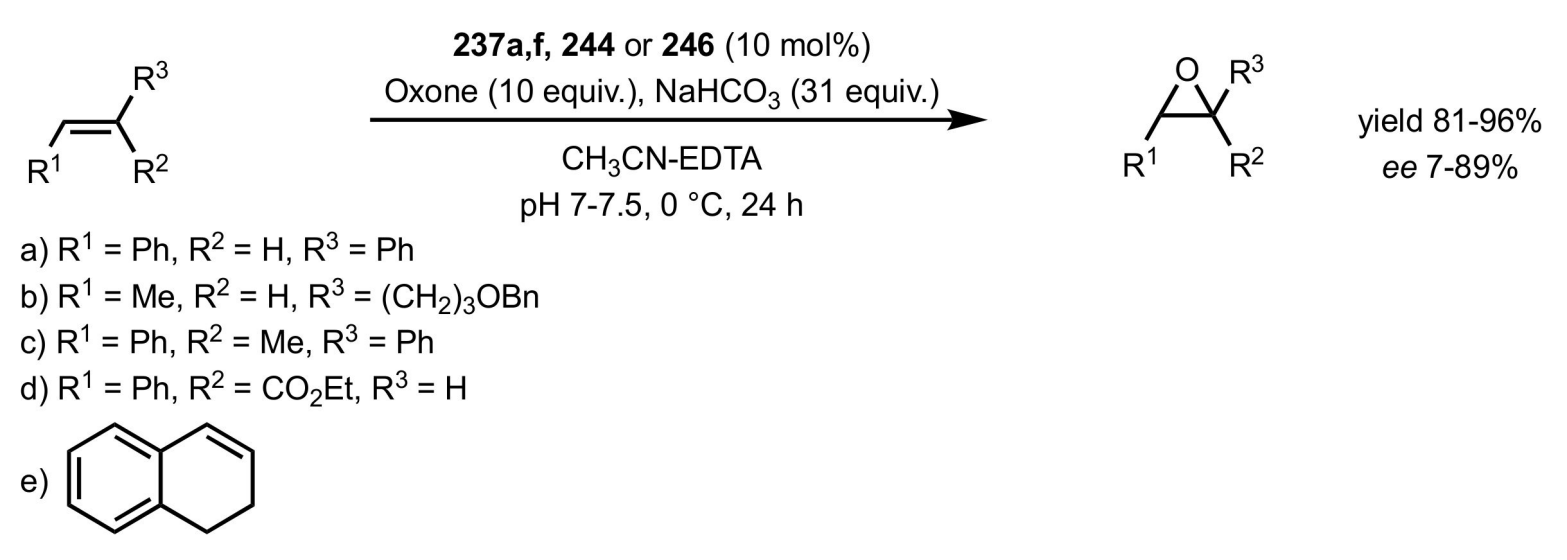
The epoxidation of *cis*- and *trans*-alkenes catalysed by ketones **237a,f**, **244** or **246**.

To compare the activity of the arabinose-derived ketones bearing cyclohexane-1,2-diacetal (**244**, **246**) or butane-1,2-diacetal (**237a,f**) protecting groups, the epoxidation of *cis*- and *trans*-alkenes was carried out (Scheme 66) under the same conditions described for the epoxidation of the ethyl *cis*-cinnamate [124]. It was observed that the exchange of the *trans*-diol protecting group did not significantly affect the enantioselectivity, with one exception. The epoxidation of (*E*)-1-benzyloxy-4-hexene catalysed by the neopentyl-substituted tricyclic ketone **246** took place with 61% enantiomeric excess, while that catalysed by the neopentyl-substituted bicyclic ketone **237f** gave the products with only 41% *ee*. These studies revealed that epoxidation of *cis*-olefins is favored by ketones with less bulky acetal alkoxy group, whereas the opposite conclusion is valid for *trans*-alkenes.

#### 3.1.3. Achievements Reported by Vega-Pérez, Iglesias-Guerra and Their Co-Workers

Based on both the reports of Shing [116,123,124] and Shi [11,113] regarding the dioxirane-mediated stereoselective epoxidation using carbohydrate-derived catalysts and their own experience in the application of sugar derivatives in asymmetric reactions [125,126,127], Vega-Pérez, Iglesias-Guerra and their co-workers synthesised new sugar-based ketones [128,129,130]. In addition to various unsymmetrical ketones bearing a single carbohydrate unit, one *C*_2_-symmetric sugar ketone was also prepared.

**Scheme 67:**
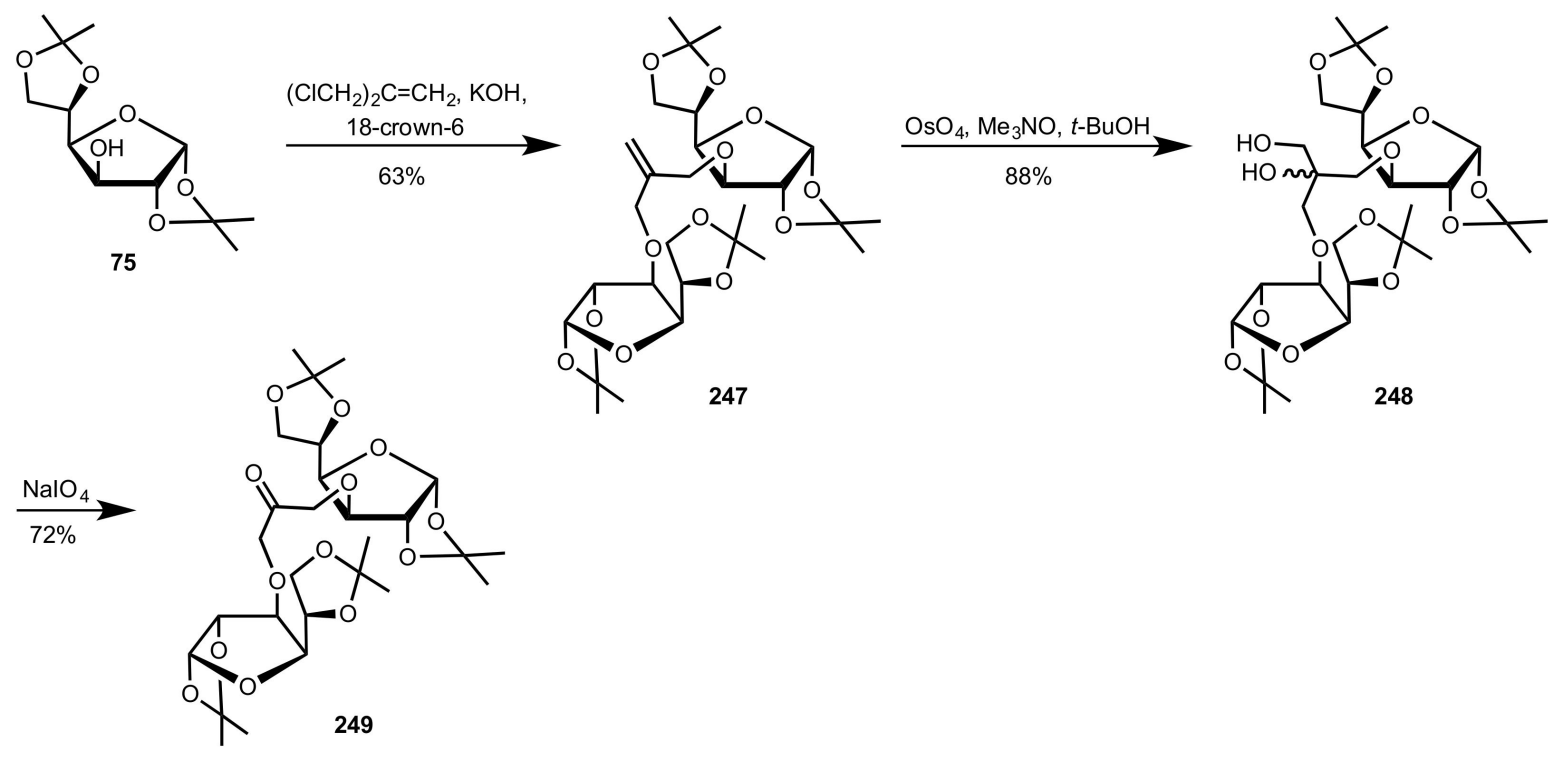
The synthesis of the *C*_2_-symmetric organocatalyst **249**.

The advantages of *C*_2_-symmetric catalysts have been widely described in the literature, e.g., enhanced rigidity of molecule and the same chiral environment, which may reduce the number of possible transition states [131,132,133,134]. The Vega-Pérez and Iglesias-Guerra teams prepared the *C*_2_-symmetric organocatalyst **249** (Scheme 67), which consisted of two functionalized d-glucose units 1,3-linked to an acetone moiety. Commercially available 1,2;5,6-di-*O*-isopropylidene-α-d-glucofuranose **75** was treated with potassium hydroxide, 18-crown-6 and 3-chloro-2-(chloromethyl)propene to obtain the symmetric derivative **247**.

**Scheme 68:**
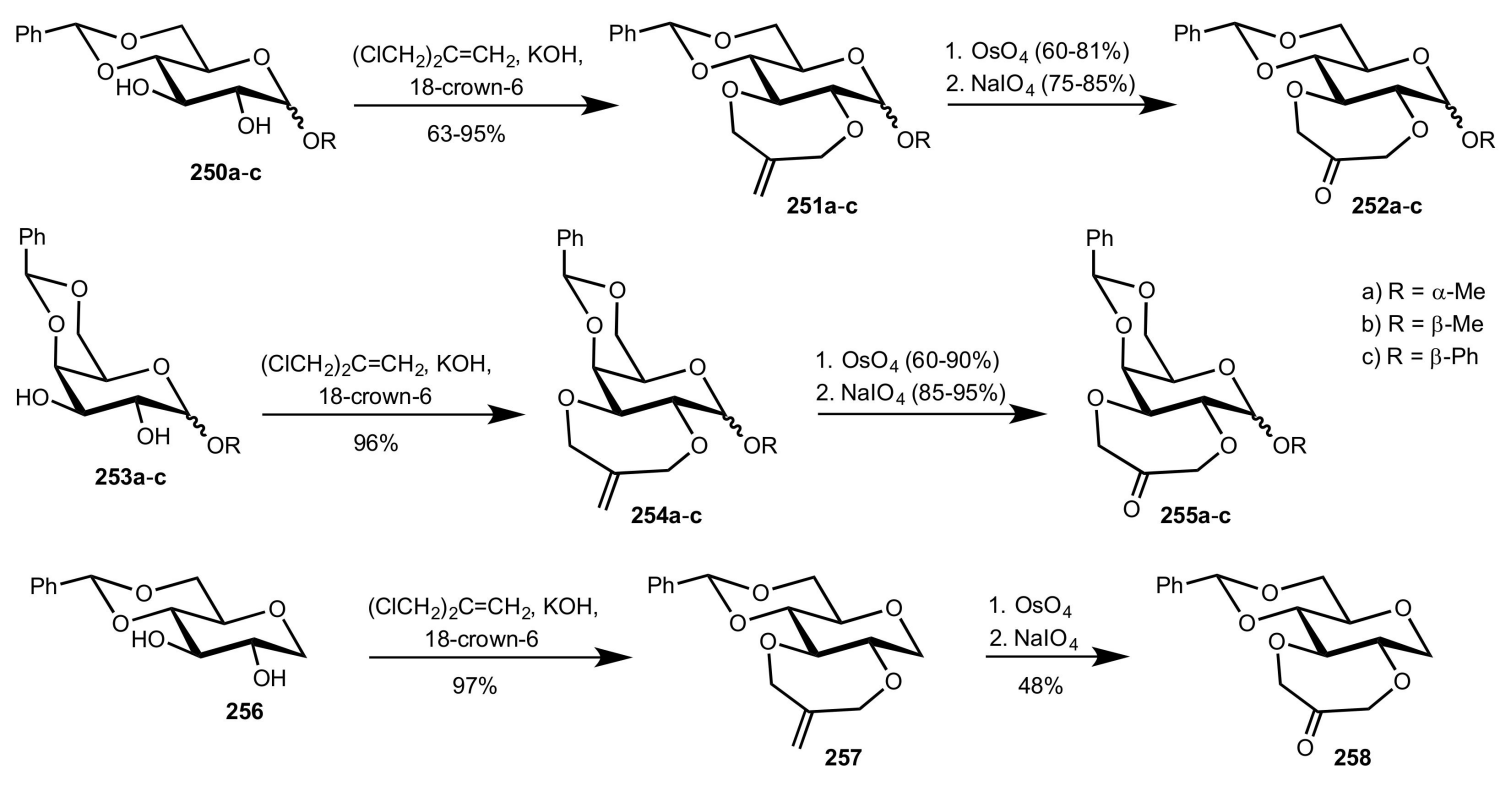
The synthesis of the d-*gluco* (**252a-c**), d-*galacto* (**255a-c**) and 1-deoxy-d-*gluco* (**258**) ketone organocatalysts.

The latter was submitted to *cis*-hydroxylation (trimethylamine *N*-oxide and osmium tetroxide) to give **248**, which, upon oxidative cleavage with sodium metaperiodate, afforded **249**. Unfortunately, the results of the epoxidation of (*E*)-stilbene and phenylcyclohexene catalysed by **249** showed that no enantioselectivity was induced. In comparison, the d-glucose-derived unsymmetrical catalyst **252a** (see Scheme 68) led to 74% *ee* for the same reaction. It is likely that the lack of stereoselectivity showed by *C*_2_-symmetric ketone **249** was due to the conformational flexibility, which differentiates a chiral environment around the dioxirane [128].

The α and β d-glucopyranose and d-galactopyranose ketones **252a-c** and **255a-c** (Scheme 68) were also synthesised applying the same reaction sequence described for the synthesis of **249** [128,129]. Moreover, aiming to check the influence of the anomeric stereocenter in glucose-derived catalysts **252a-c**, the authors prepared the 1-deoxygenated analogue **258** starting from the 4,6-*O*-benzylidene derivative **256** (Scheme 68).

**Scheme 69:**
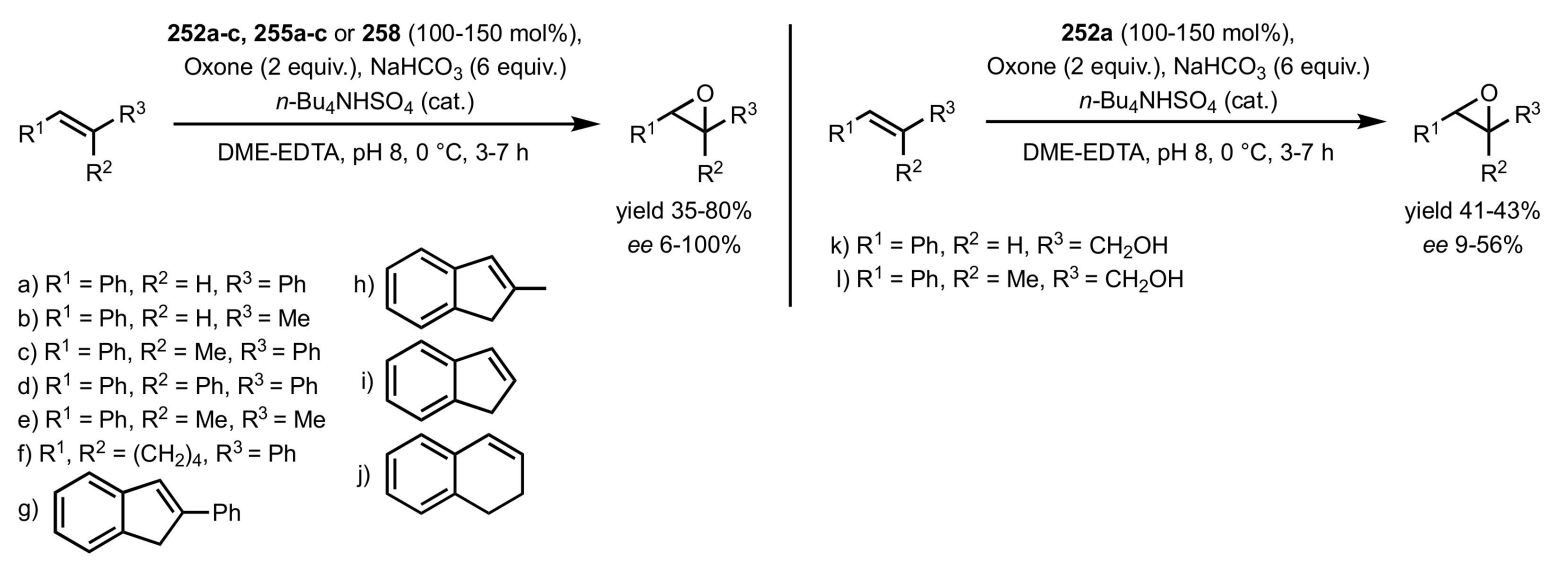
The epoxidation of aryl alkenes and allylic alcohols catalysed by **252a-c**, **255a-c** and **258**.

Various alkenes bearing aryl moiety were epoxidated by the sugar ketones **252a-c**, **255a-c** and **258** to give the (*S*)-configured products in satisfactory yield (35–80%) (Scheme 69). The investigations revealed that the α-anomeric organocatalysts led to better stereoselectivity than the β-counterparts (57–100% and 38–77% *ee*, respectively). Moreover, it was found that the deprotection of the α or β anomeric position slightly decreased the *ee* values, giving similar results in both cases (39–60% *ee*), and that galactose-derived ketones were better chirality inducers than glucose-derived ones.

Furthermore, the character of a substituent in anomeric position (aromatic vs. aliphatic) had no influence on the enantioselectivity in case of glucose-derived catalysts, whereas for galactose-derived analogues it was observed that aromatic groups led to lower enantiomeric excess [128,129]. Finally, the epoxidation of the more challenging *cis*-olefins **h-j** (Scheme 69) did not take place using either glucose- (**252b,c**) or galactose-derived (**255a**) catalysts [129].

Aiming to test the efficacy of catalyst **252a** in the epoxidation of allylic alcohols, two additional reactions were conducted with substrates **k** and **l** applying the above-mentioned procedure (Scheme 69) [128,129]. However, the isolated yield and enantioselectivity were modest (41–43% yield, 9–56% *ee*).

**Figure 21:**
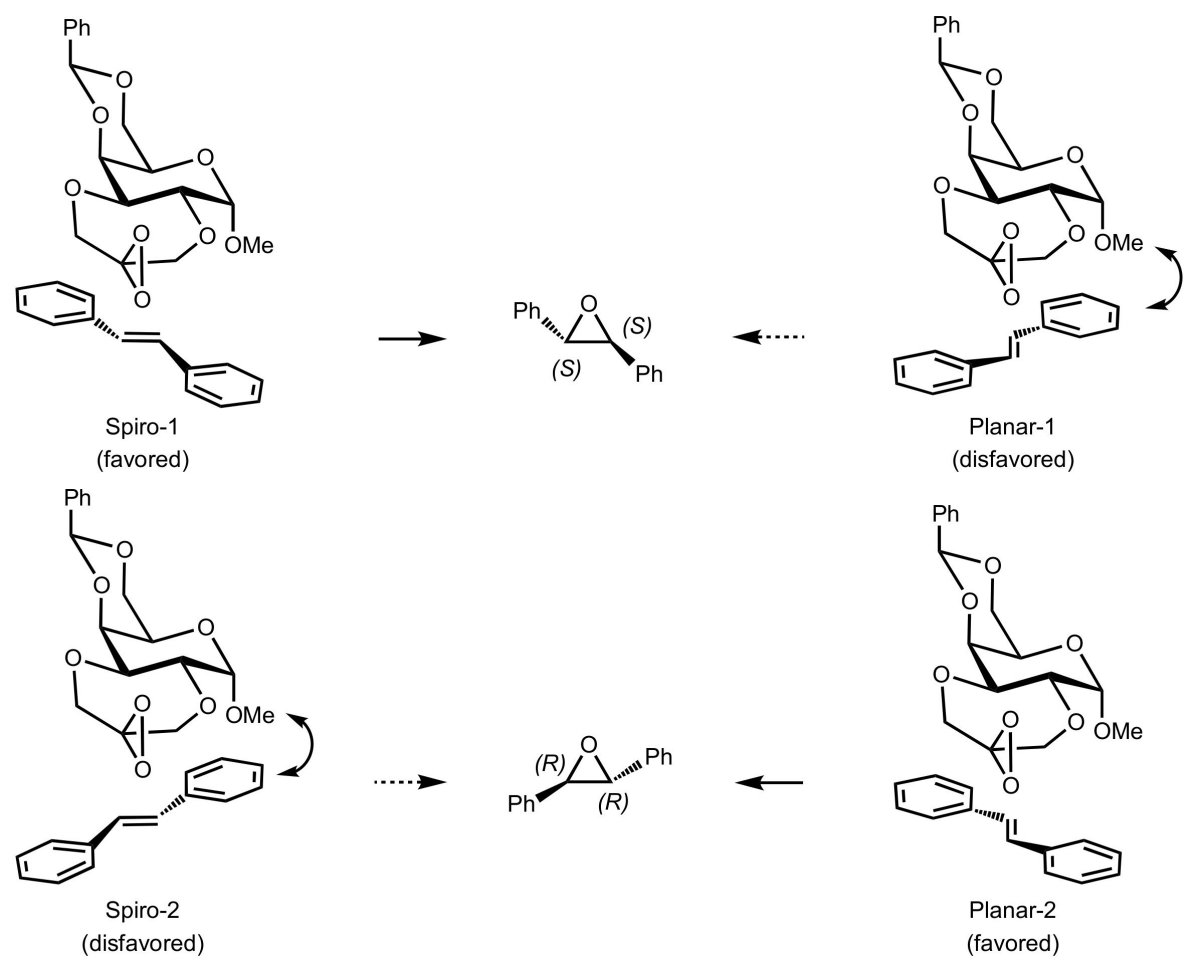
Competitive transition states.

Figure 21 shows the transition states proposed by Baumstark [135] for the epoxidation of *trans*-stilbene catalysed by ketone **255a**. It was noticed that (*S,S*)-stilbene oxide would be formed if the reaction proceeded via a spiro intermediate; whereas, for (*R*,*R*)-stilbene oxide the planar mode should be favored. Since the (*S*,*S*)-enantiomer is the major product, it is believed that the reaction proceeds over spiro transition state. Moreover, attack of *trans*-stilbene on the other oxygen atoms of dioxirane leads to formation of the (*S*,*S*)-enantiomer as well [129].

**Scheme 70:**
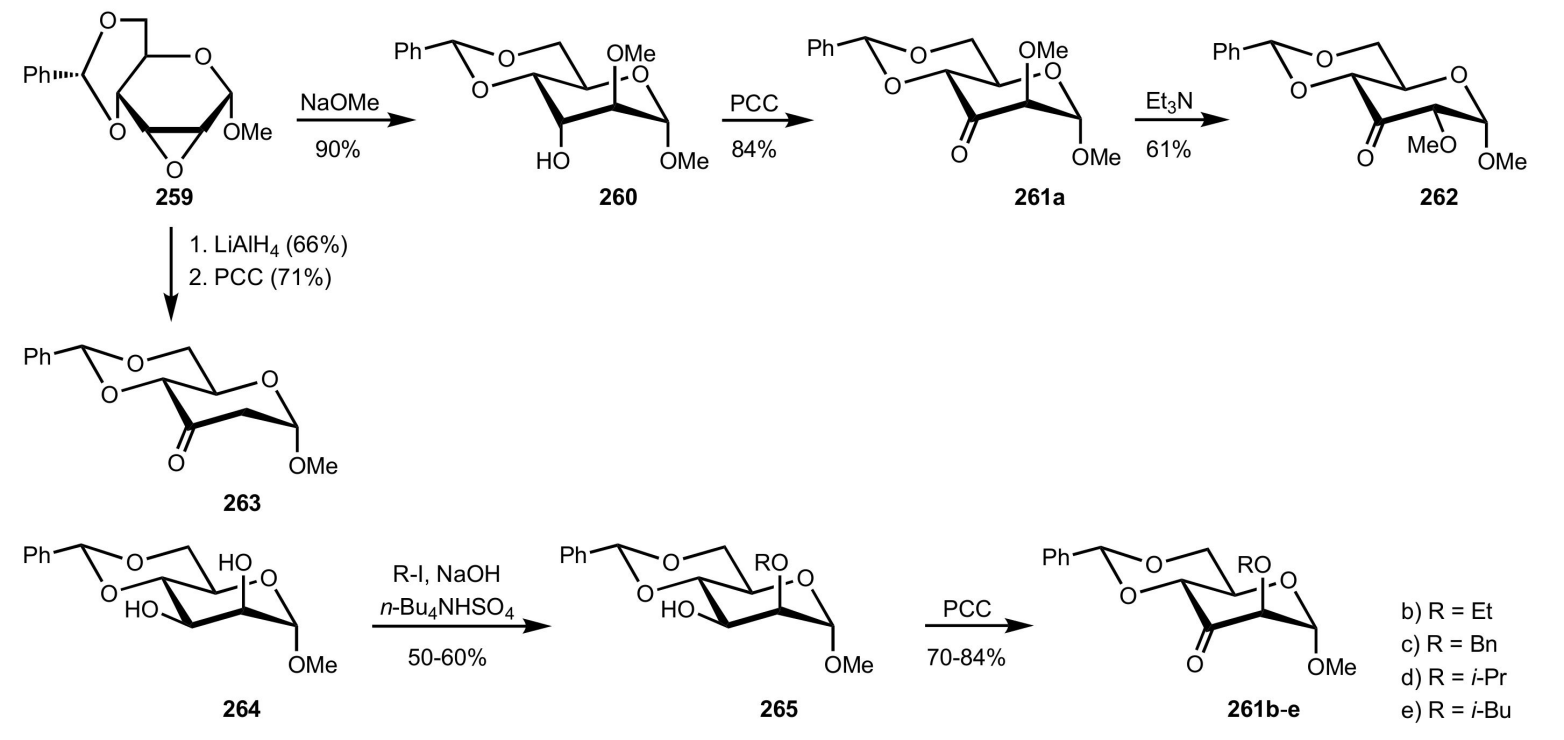
The synthesis of mannose-derived ketones **261a-e, 262** and **263**.

Continuing their studies on sugar ketones, Vega-Pérez, Iglesias-Guerra and their co-workers prepared organocatalysts with a modified sugar backbone, namely d-mannose derivatives [131]. The alcohol **260**, obtained through ring opening of the known [136] 2,3-anhydro-4,5-*O*-(*R*)-benzylidene-α-d-allopyranoside **259** by sodium methoxide, was oxidised to the corresponding ketone **261a** under standard conditions (Scheme 70). Its epimer **262** was synthesised by refluxing the latter and Et_3_N in ethanol, whereas the 2-deoxygenated derivative **263** was prepared by treatment of the epoxide **259** with LiAlH_4_ and oxidation of the resulting alcohol with pyridinium chlorochromate (PCC) (Scheme 70).

The epoxide opening reaction of **259** proceeded in high yield with NaOMe, but failed when other sodium alkoxides (i.e., ethoxide and isopropoxide) were employed. Therefore, in order to prepare the ketones **261b-e**, a new approach was developed. The diol **264**, easily obtained by reaction of methyl α-d-mannopyranoside with α,α-dimethoxytoluene and *p*-TSA [137], was regioselectively alkylated with various alkyl iodides in the presence of NaOH and *n*-Bu_4_NHSO_4_ to give **265**, which, after oxidation, afforded the products **261b-e** without epimerization (Scheme 70) [131].

**Scheme 71:**
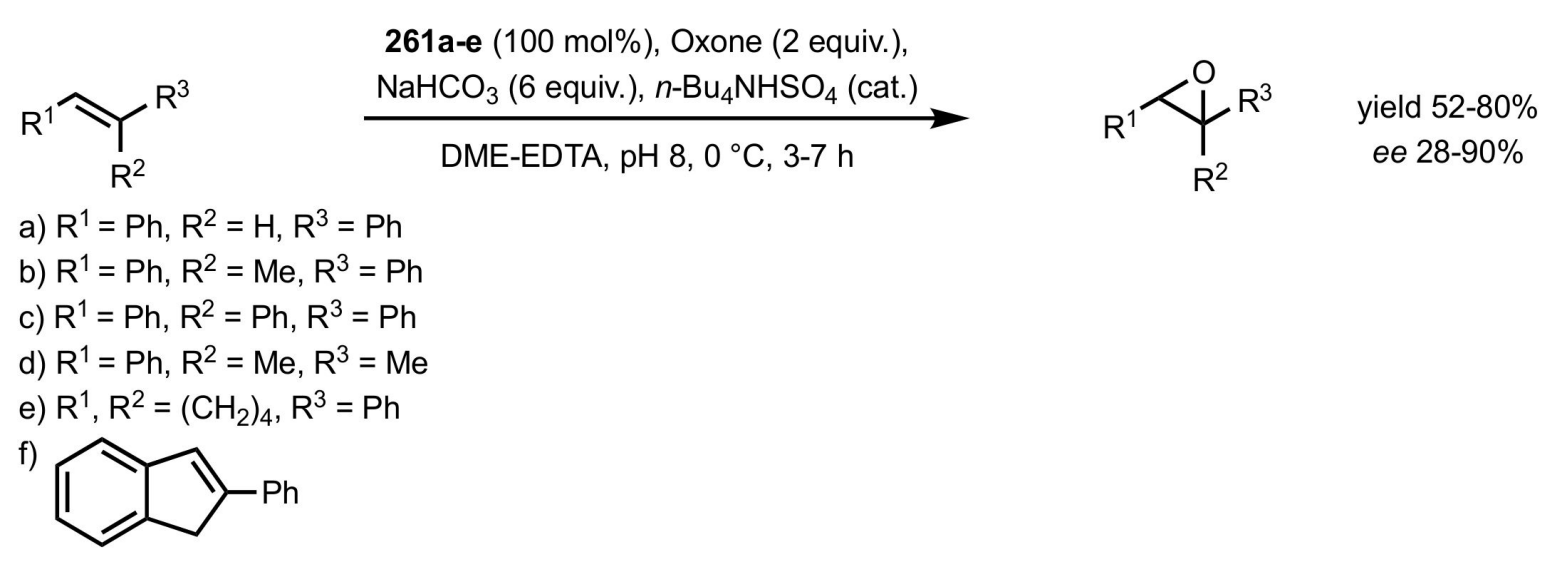
The epoxidation of *trans*- and trisubstituted alkenes catalysed by ketones **261a-e**.

To determine the influence of the 2-alkoxy group on the enantioselectivity, the sugar ketones **261a-e** were tested in the epoxidation of some *trans* and tri-substituted olefins (Scheme 71). It was found that the organocatalyst bearing the least bulky group (**261a**) was the most efficient (up to 90% *ee*), whereas the 2-*O*-isobutyl-ketone derivative **261e** exhibited the lowest enantioselectivity. The epoxidation of olefins **a-c** (Scheme 71) catalysed by the C-2 epimeric ketones **261a** and **262** showed that both catalysts led to formation of (*R*)- or (*R*,*R*)-enantiomers as main products, but **262** led to the products in lower yield and enantiomeric excess than **261a**.

The comparison of ketone **263** with **261a** resulted in a similar correlation. Since ketone **261a** emerged as the most effective catalyst, it was also employed for the epoxidation of two more demanding terminal (styrene) and *cis*-alkenes (dihydronaphthalene). However, the stereoselectivity was low (20% *ee*) in the case of styrene or totally absent with dihydronaphthalene as the substrate.

**Figure 22:**
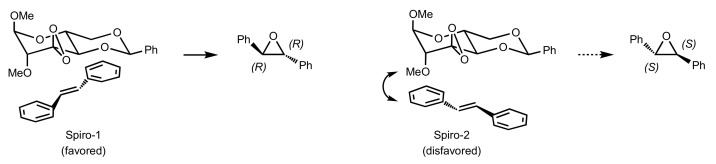
The proposed transition states for the epoxidation of *trans*-stilbene catalysed by **261a**.

A putative mechanism of the epoxidation of *trans*-stilbene catalysed by **261a** is presented in Figure 22. It is likely that the formation of (*R*,*R*)-epoxide proceeds by the spiro-1 transition state due to the easier access of the substrate to the equatorial oxygen atom of the catalyst dioxirane. The spiro-2 transition state is less favored due to the steric hindrance caused by phenyl group of the substrate and the anomeric methoxy group of the catalyst. As (*R*,*R*)-products were observed predominantly in this case, the results are in agreement with the proposed spiro-1 transition state.

**Scheme 72:**
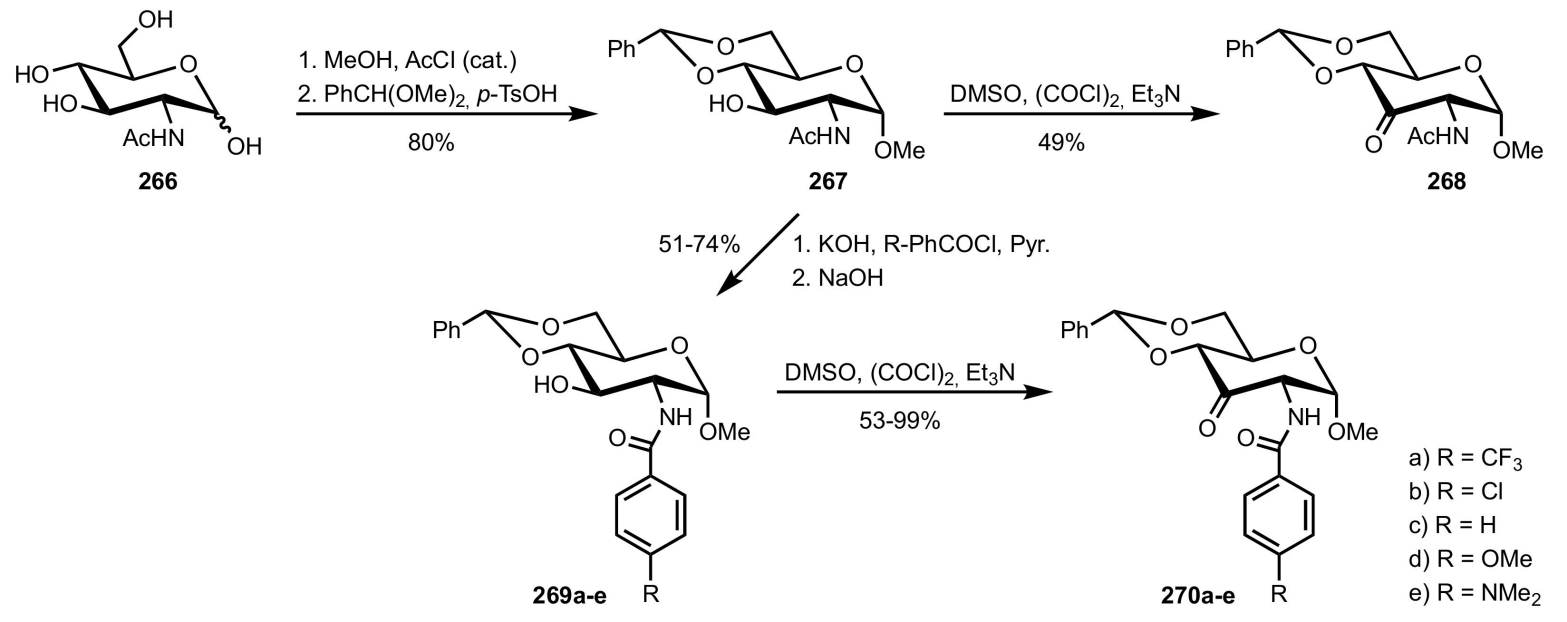
The synthesis of d-glucosamine-derived organocatalysts **268** and **270a-e**.

A similar approach was used by Davis and co-workers [138] who directed their efforts towards the design and synthesis of a set of *N*-acyl-glucosamine ketones for the epoxidation of challenging substrates [100,113], such as 2,2-disubstituted and terminal olefins. The commercially available *N*-acetyl-d-glucosamine **266** was first glycosidated with methanol and protected as 4,6-*O*-benzylidene acetal to give **267**, then the latter was submitted to the Swern oxidation to afford **268** (Scheme 72) [138]. The acetamido derivative **267** was also hydrolyzed under basic conditions, and the resulting 2-amino sugar was acylated with variously substituted benzoyl chlorides to give **269a-e**. Oxidation of these products afforded the desired ketones **270a-e** in a moderate to excellent yield (Scheme 72).

**Scheme 73:**
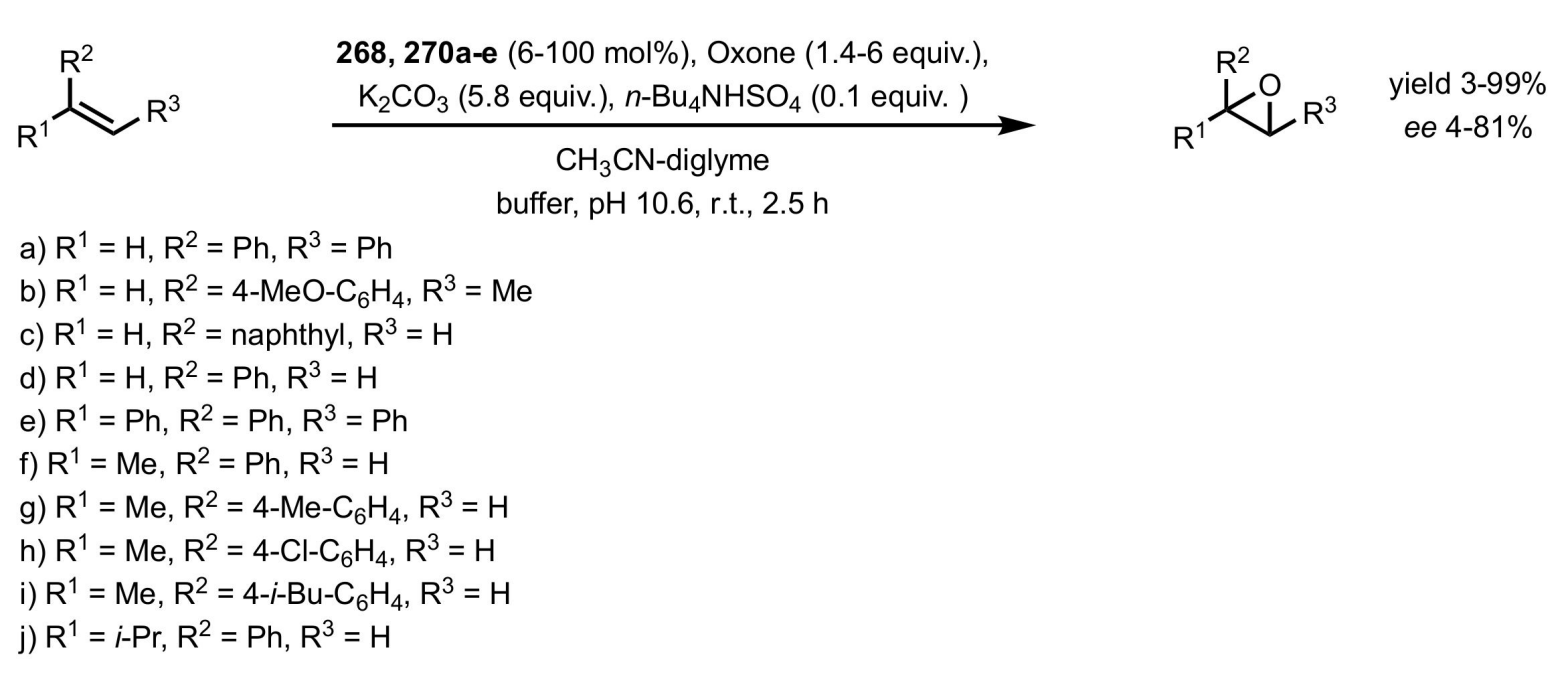
The epoxidation of 2,2-disubstituted and terminal alkenes catalysed by **268** and **270a-e**.

As mentioned above, the new organocatalysts **268** and **270a-e** were employed for the epoxidation of several 2,2-disubstituted and terminal olefins (Scheme 73). Although the enantiomeric excesses observed for the reaction with terminal alkenes were low (4–42%), for the styrene epoxidation the product was recovered in high yield (67–85%) and good enantioselectivity (66–81% *ee*) [138].

### 3.2. Enantioselective Oxidation of Disulfides

**Scheme 74:**
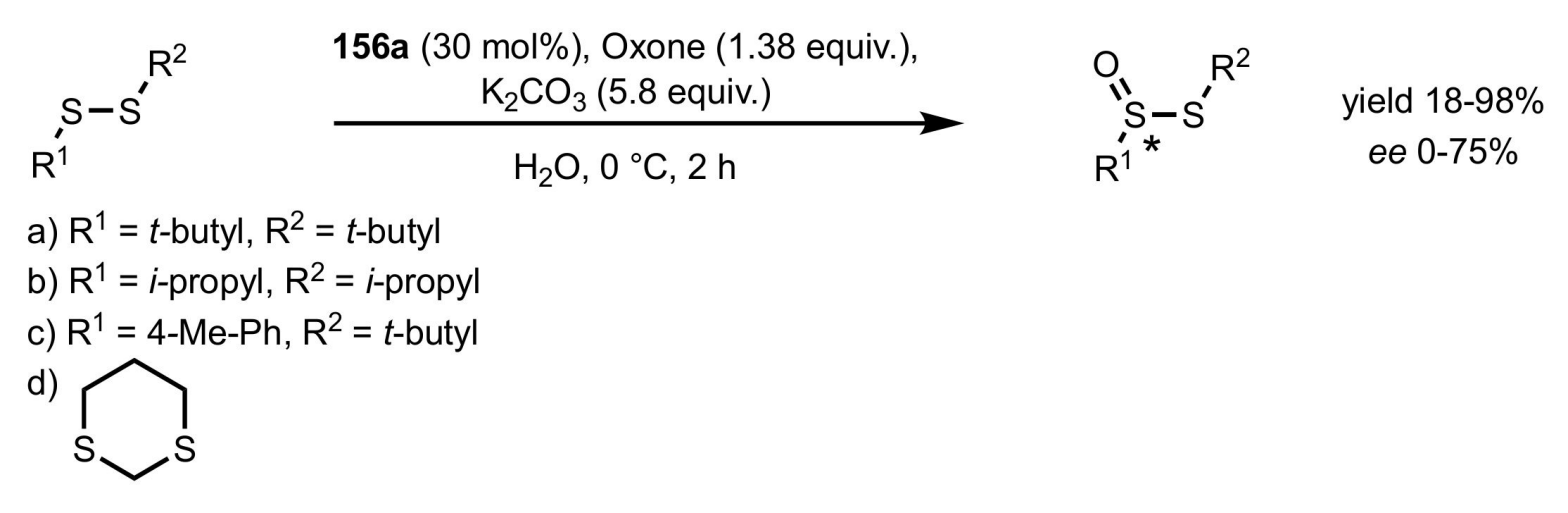
Enantioselective synthesis of thiosulfinates catalysed by **156a**.

In 2005, the sugar ketone organocatalyst **156a** (see Scheme 33) was exploited by Colonna and co-workers for the enantioselective synthesis of thiosulfinates by oxidation of disulfides with Oxone [139]. However, the reported study was limited to only four examples (Scheme 74).

**Scheme 75:**
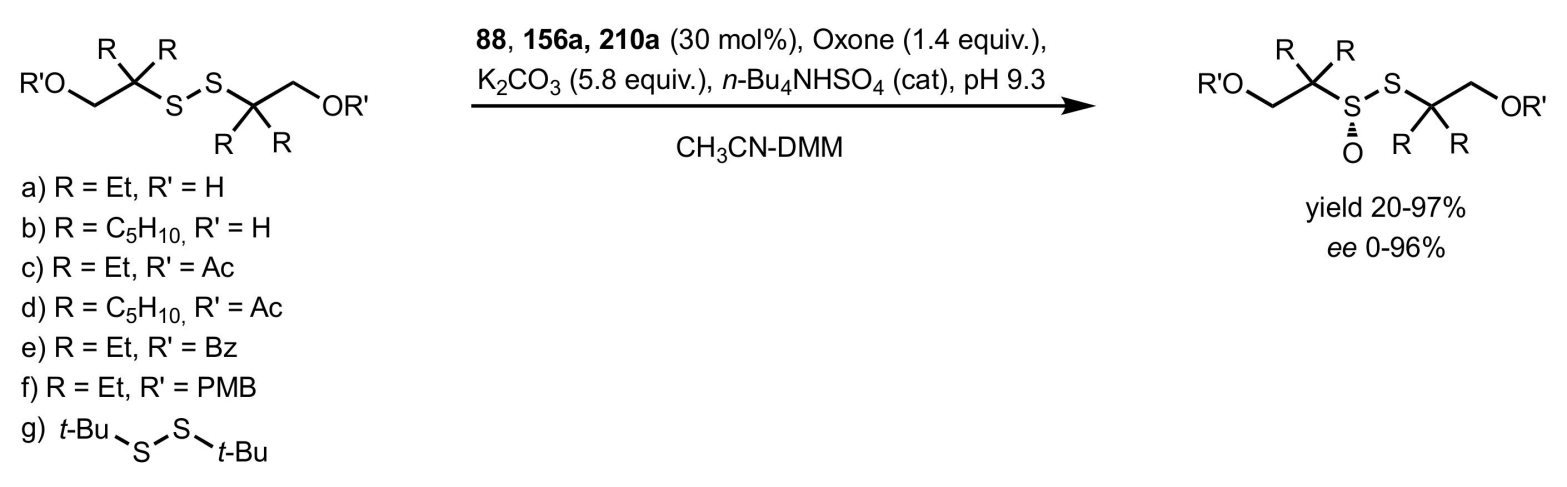
The synthesis of chiral sulfinyl derivatives catalysed by **88**, **156a** and **210a**.

The sugar ketones **88** (see Scheme 19), **156a** and **210a** (see Scheme 55) were also tested by Khiar, Fernández and their co-workers in the asymmetric oxidation of disulfides (Scheme 75) [140]. Screening of these catalysts in the synthesis of chiral thiosulfinate from di(*tert*-butyl disulfide) showed that the ketone **156a** was the most efficient (up to 97% yield and 96% *ee*). Interestingly, the d-glucose-derived ketone **88** did not induce enantioselectivity. More extensive studies showed that disulfides bearing electron-poor protecting groups gave better yields and enantioselectivity than those with electron-rich substituents.

Therefore, the results of two independent studies indicated that ketone **156a**, in addition to its broad application in the synthesis of chiral epoxides, may serve as a useful tool for asymmetric oxidation of disulfides.

## 4. Sugar Prolinamides

**Figure 23:**
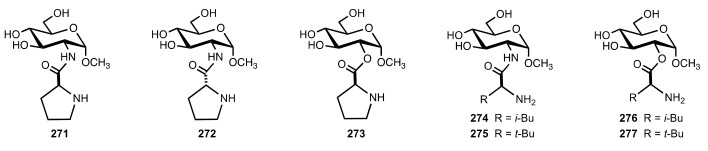
Sugar-proline and sugar-leucine derivatives prepared by Machinami and co-workers.

In 2007, Machinami and co-workers described the use of the sugar-proline amides **271**, **272** (Figure 23) and ester (**273**) as organocatalysts for the aldol reaction [141]. The sugar-leucine amide (**274**) and ester (**276**) as well as the *t*-butyl-leucine analogues **275** and **277** were also employed. The sugar amides were prepared by condensation of methyl 2-amino-2-deoxy-α-d-glucose with the corresponding amino acids in the presence of a carbodiimide, whereas the sugar esters were obtained by regioselective acylation of a protected methyl α-d-glucopyranoside. Unfortunately, only the analytical and spectral data of these new compounds were reported in the publication; the experimental procedures and yields were missing [141].

**Scheme 76:**
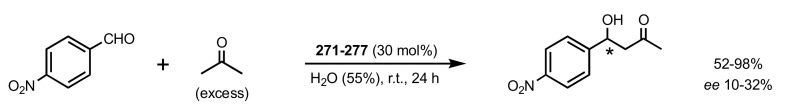
Aldol reaction catalysed by **271–277**.

The amides and esters **271–277** were used to catalyse exclusively the aldol reaction of *p*-nitrobenzaldehyde with acetone (reagent and solvent) in the presence of water (55% *v*/*v*) (Scheme 76). The aldol was obtained in good yield (HPLC analysis) but low enantiomeric excess; however, it was found that the organocatalysts **271**, **274**, **275** bearing an l-amino acid moiety led to the (*R*)-configured adduct while the d-amino acid derivative **272** gave the (*S*)-enantiomer. When the amount of water was decreased from 55 to 5%, significant increase in enantioselectivity was observed; however, the adducts were formed at a much lower yield.

**Scheme 77:**
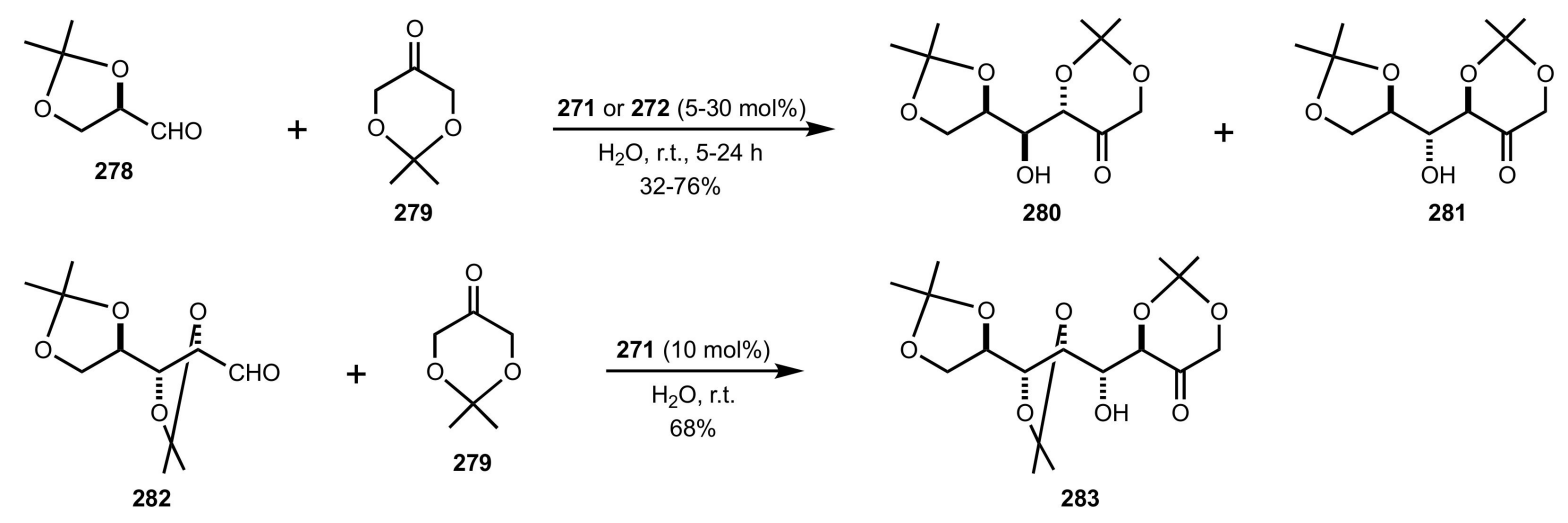
Aldol reactions of *aldehydo*-sugars catalysed by **271** or **272**.

A few years later, the same research team exploited [142] the sugar prolinamides **271** and **272** to catalyse the aldol reaction between the d-glyceraldehyde acetonide **278** and the dihydroxyacetone acetonide **279** in water (Scheme 77). After several hours at room temperature, the ketose **280** was the major adduct in the presence of **271** (**280**:**281** = 7.3:1), whereas the stereoisomer **281** was stereoselectively formed in the presence of **272** (**280**:**281** = 1:24).

The other possible stereoisomers (not shown) were obtained only when DMSO was used as the solvent. The reaction of **279** with the protected *aldehydo*-d-arabinose **282** catalysed by **271** afforded a 10:1 mixture of **283** and an unidentified stereoisomer, while the use of the organocatalyst **272** led to a mixture of the four possible stereoisomers. Interestingly, the aldol reaction of **279** with the enantiomer of **282** (i.e., the l-arabinose derivative) was extremely sluggish, and only trace amounts of the corresponding adducts were formed.

**Scheme 78:**
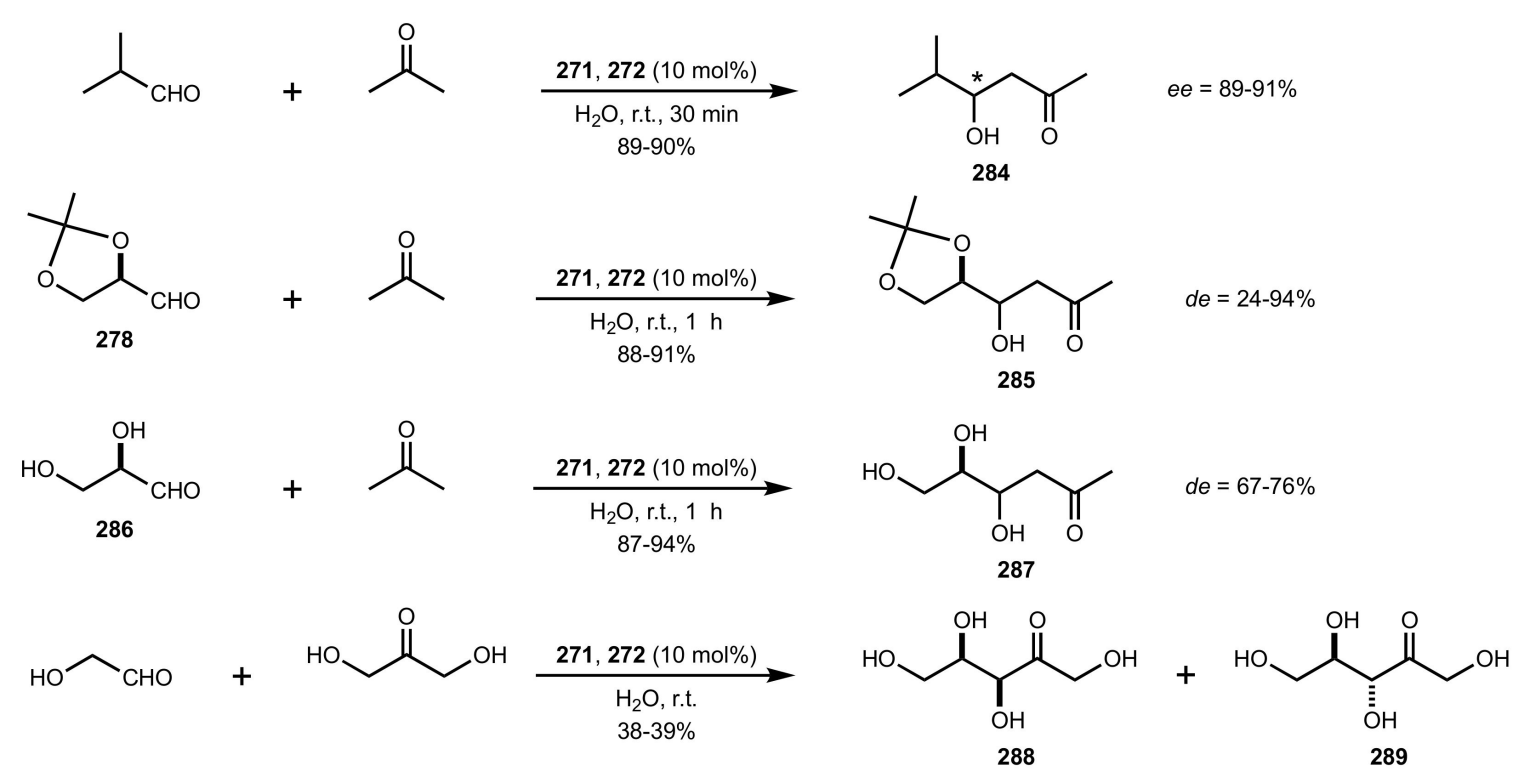
Aldol reactions catalysed by **271** and **272**.

In a more recent article, Miura and Machinami described [143] other examples of aldol reaction catalysed by sugar prolinamides **271** and **272** (Scheme 78). The reaction of isobutyraldehyde with acetone led to the (*R*)-enantiomer (90%, *ee* = 89%) when performed in the presence of l-prolinamide **271** and the (*S*)-enantiomer (89%, *ee* = 91%) when the d-prolinamide **272** was used. On the other hand, the reaction of d-glyceraldehyde acetonide **278** with acetone catalysed by **271** or **272** gave the aldol **285** in high yield but different stereoselectivity (**271**: *syn* aldol, *de* = 24%; **272**: *anti* aldol, *de* = 94%).

Starting from l-glyceraldehyde acetonide ***ent*-278** (not shown), both organocatalysts afforded the *anti* aldol in good yield (75–96) and diastereomeric excess (63–81%). The results with the unprotected d-glyceraldehyde **286** were similar to those observed for **278** since the aldol **287** was mainly *syn* (*de* = 67%) using **271** and mainly *anti* (*de* = 76%) when the reaction was catalysed by **272**. Finally, the reaction of glycolaldehyde with 1,3-dihydroxyacetone afforded the d-xylulose **288** (39%, *de* = 77%) in the presence of **271** and the d-ribulose **289** (38%, *de* = 81%) in the presence of **272**.

**Figure 24:**
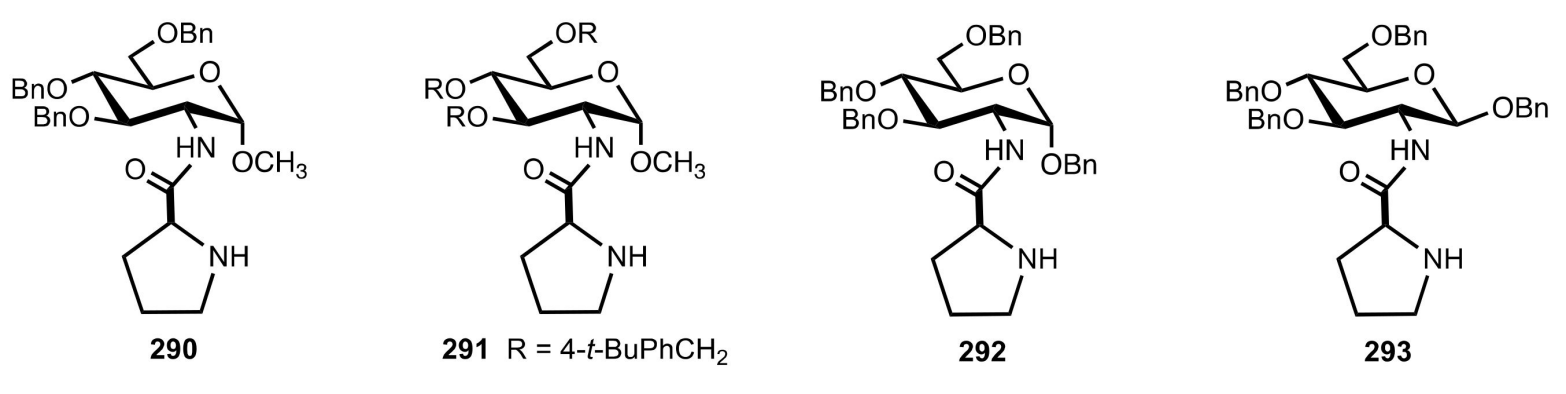
Sugar prolinamide organocatalysts prepared by Agarwal and Peddinti.

The *O*-protected d-glucosamine l-prolinamide derivatives **290–293** (Figure 24), closely related to the above-mentioned sugar prolinamide **271** (see Figure 23) described by Machinami and co-workers, were prepared and employed as organocatalysts by Agarwal and Peddinti [144,145,146]. However, in the first two articles, the synthesis and analytical data of the compounds **290–292** were not provided; whereas, in the last paper [146], they reported the detailed preparation and characterization of the d-glucosamine precursors to prolinamides **290–293**. Amazingly, the same syntheses and characterizations were already reported in an article published the previous year (see Scheme 128 in Section 6.2).

Moreover, in their 2012 paper [146], Agarwal and Peddinti claimed the first preparation of methyl 2-acetamido-3,4,6-tri-*O*-benzyl-2-deoxy-β-d-glucopyranoside, an intermediate for other sugar prolinamides, which, however, was an already known compound because it was prepared in 1974 by Jacquinet and Sinaÿ [147] (since then, other syntheses were made available by other researchers).

**Scheme 79:**
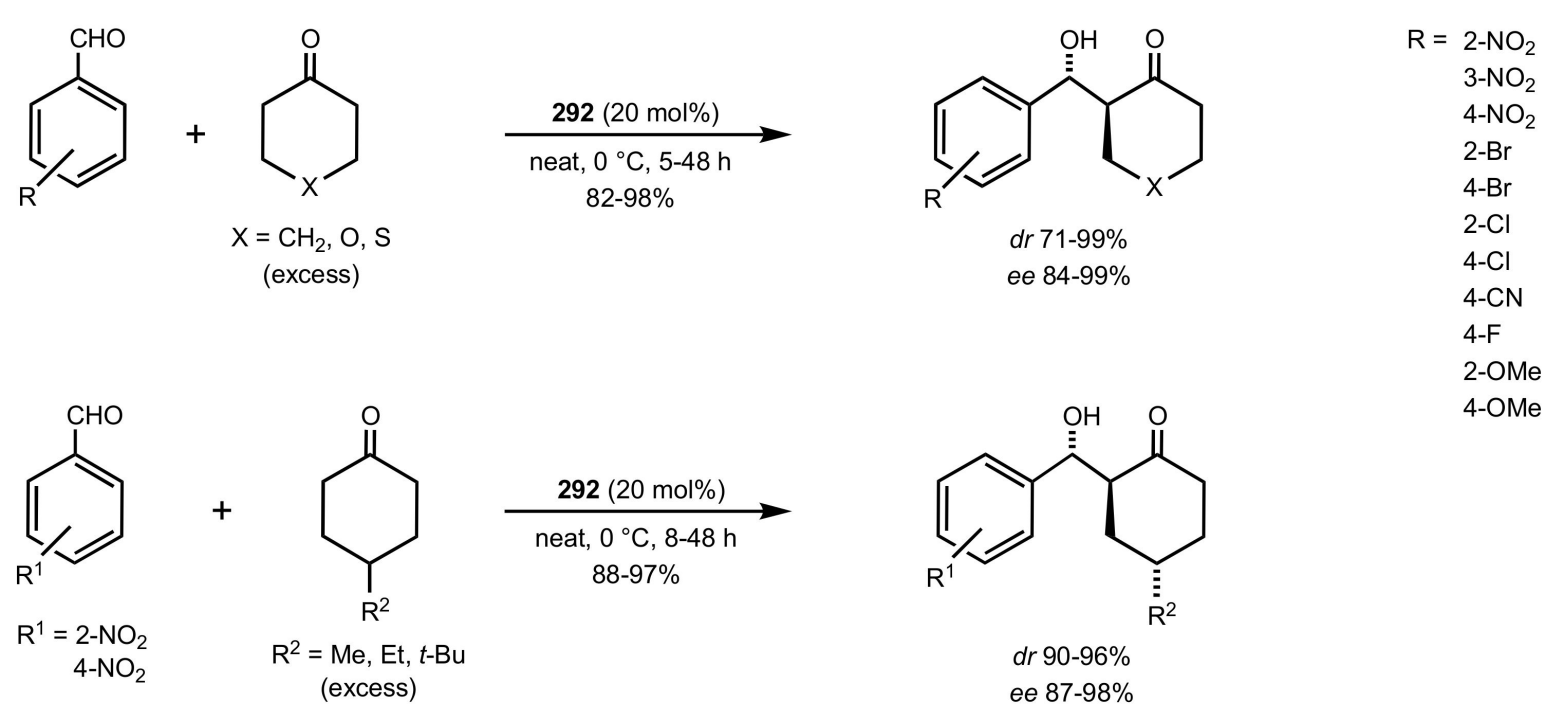
Aldol reactions catalysed by **292**.

Organocatalyst **292** was found to give the better results in model aldol reactions, therefore, it was used to promote [144,146] the reaction of a series of aromatic aldehydes with cyclohexanone, tetrahydropyran-4-one and tetrahydrothiopyran-4-one leading to the *anti* adduct in high yield and stereoselectivity (Scheme 79). When the same aldehydes were reacted with acetone or cyclopentanone, a much lower *anti*/*syn* diastereoselectivity was observed [146]. The reaction of *o*- or *p*-nitrobenzaldehyde with prochiral 4-alkyl-cyclohexanones gave the corresponding aldols in high diastereo- and enantioselectivity [146].

**Scheme 80:**
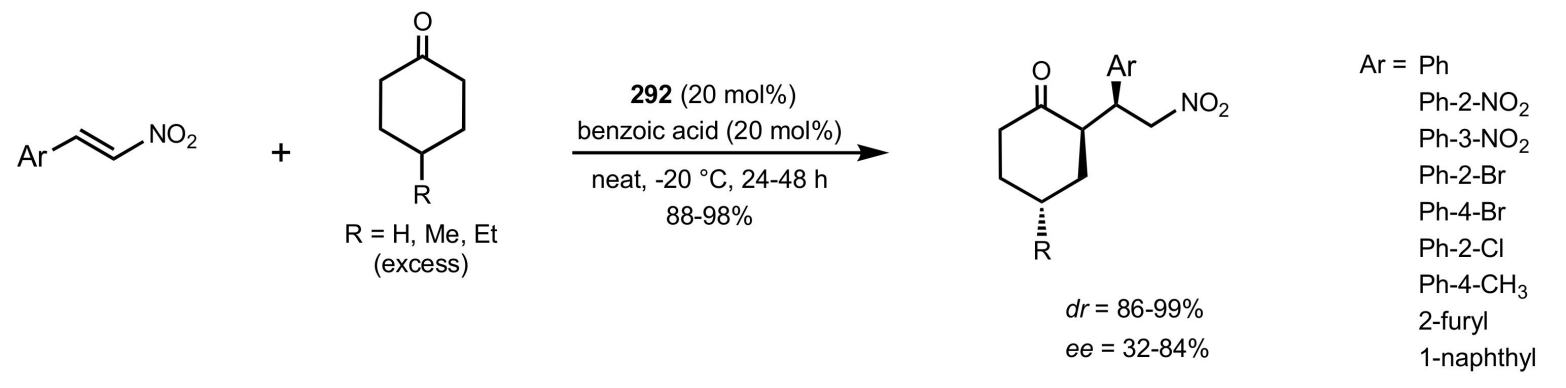
The Michael additions catalysed by **292**.

Agarwal and Peddinti employed the sugar prolinamide **292** also to catalyse the Michael addition at low temperature of cyclohexanone derivatives to a series of aromatic nitroalkenes [145]. The *syn* adduct was preferentially formed in variable enantiomeric excess (Scheme 80).

**Scheme 81:**
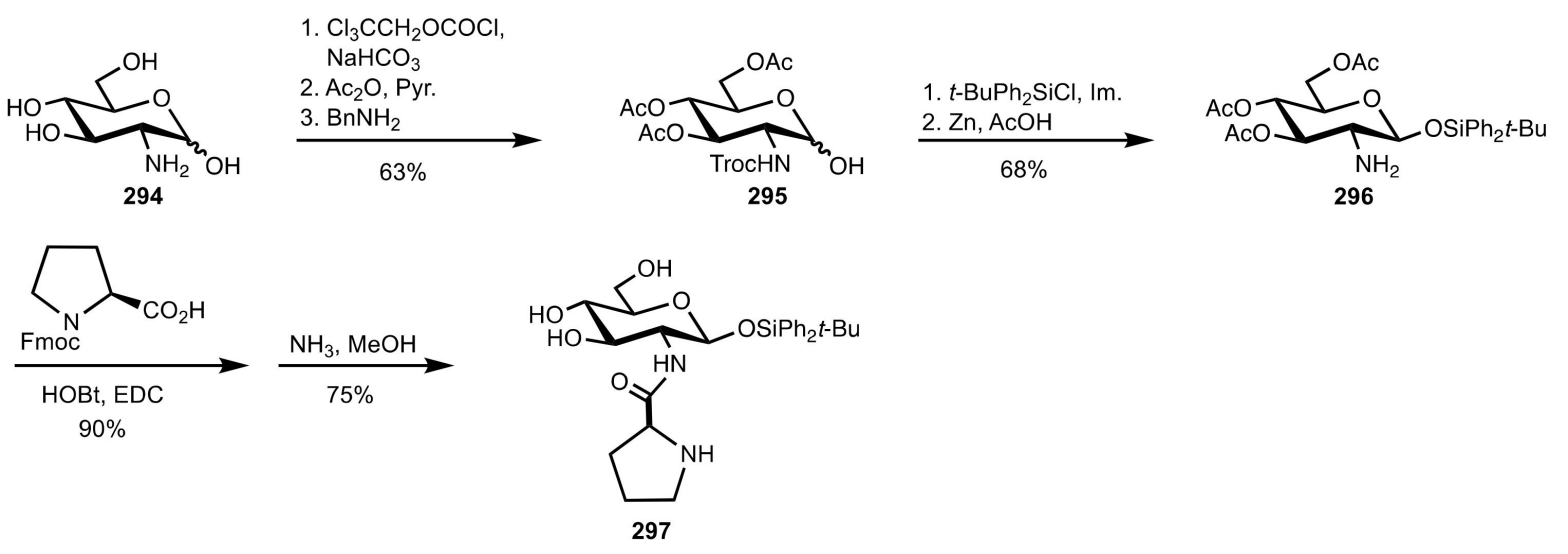
The synthesis of the sugar prolinamide organocatalyst **297**.

The sugar prolinamide **297** (Scheme 81), featuring three hydroxyl groups and a large hydrophobic group onto the carbohydrate moiety, was designed by Caputo and co-workers to catalyse the aldol reaction in aqueous media [148,149]. The synthesis of **297**, not reported in their first article (main text or supporting information), started from d-glucosamine (**294**), which was *N*-protected by reaction with trichloroethoxycarbonyl chloride, peracetylated and regioselectively deacetylated to afford the hemiacetal **295** [149]. This compound was silylated and then treated with zinc in acetic acid to remove the Troc protecting group.

The glucosamine derivative **296** was condensed with commercially available *N*-Fmoc-l-proline in the presence of hydroxybenzotriazole (HOBt) and *N*-ethyl-*N*′-(3-dimethylaminopropyl)carbodiimide (EDC) to give the corresponding amide, which was deacetylated in methanolic ammonia to afford **297** in good yield. It is worth noting that, in a 2011 article [148], the d-sugar l-prolinamide **297** was erroneously drawn as a l-sugar l-prolinamide stereoisomer.

**Scheme 82:**
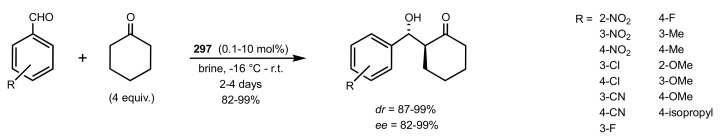
Aldol reactions catalysed by **297**.

As reported in both articles (identical experiments, schemes, tables and sentences) [148,149], the sugar prolinamide **297** was employed to catalyse the aldol reaction of aromatic aldehydes with cyclohexanone in brine as the solvent (Scheme 82). The *anti* aldol was obtained in high yield and excellent diastereo- and enantioselectivity. Although several reaction temperatures and catalyst loadings were tested, most of the reactions were performed at 4 °C for 48 h in the presence of 2 mol% of **297**.

**Scheme 83:**
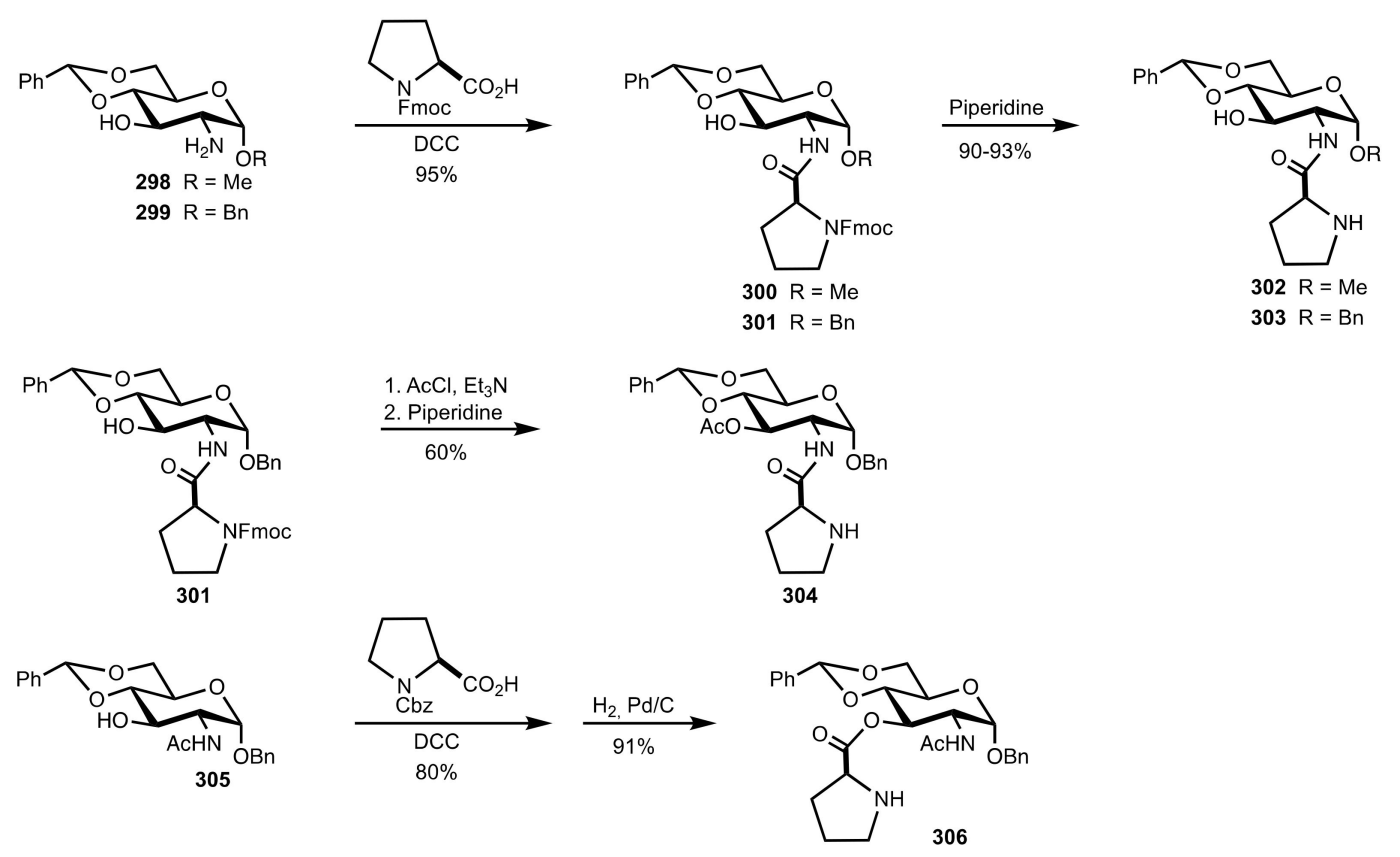
The synthesis of the sugar prolinamide organocatalysts **302–304** and **306**.

Shortly after the publication of the synthesis and application of *O*-protected sugar prolinamides **290–293** (see Figure 24), similar organocatalysts for the aldol reaction were reported by Zhang and co-workers [150]. However, in this case, the sugar prolinamides **302** and **303** (Scheme 83) featured a free hydroxyl group in order to perform the catalysis in water, and their properties were compared to the fully protected analogues **304** and **306**.

The organocatalysts **302** and **303** were easily prepared by coupling the 2-amino-glucosides **298** and **299** (see Section 6.2) with *N*-Fmoc-l-proline in the presence of dicyclohexylcarbodiimide (DCC) to give **300** and **301** followed by *N*-deprotection with piperidine. The 3-*O*-acetylated derivative **304** was synthesised from **301** through acetylation and *N*-deprotection, whereas **306** was prepared by condensation of the 2-acetamido-glucoside **305** (see Section 6.2) with *N*-Cbz-l-proline and hydrogenolysis [150].

**Scheme 84:**
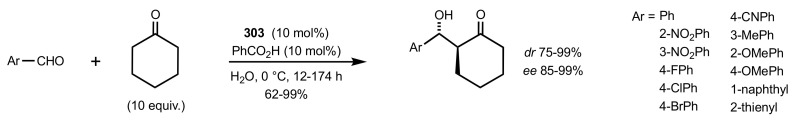
Aldol reactions catalysed by **303**.

As expected, a model aldol reaction allowed for finding that the free hydroxyl derivatives **302–303**, in particular the benzyl glucoside **303**, were more efficient organocatalysts than the fully protected analogues **304** and **306** [150]. Therefore, a series of aromatic and heteroaromatic aldehydes were condensed in water with an excess of cyclohexanone to give, after as many as 174 h at 0 °C, the corresponding *anti* aldol in high diastereo- and enantioselectivity (Scheme 84). Similar good results were observed for the aldol reaction of 4-nitrobenzaldehyde and cyclopentanone or butanone (not shown). Moreover, the sugar prolinamide **303** could also be recycled five times without a significant decrease in the stereoselectivity.

**Scheme 85:**
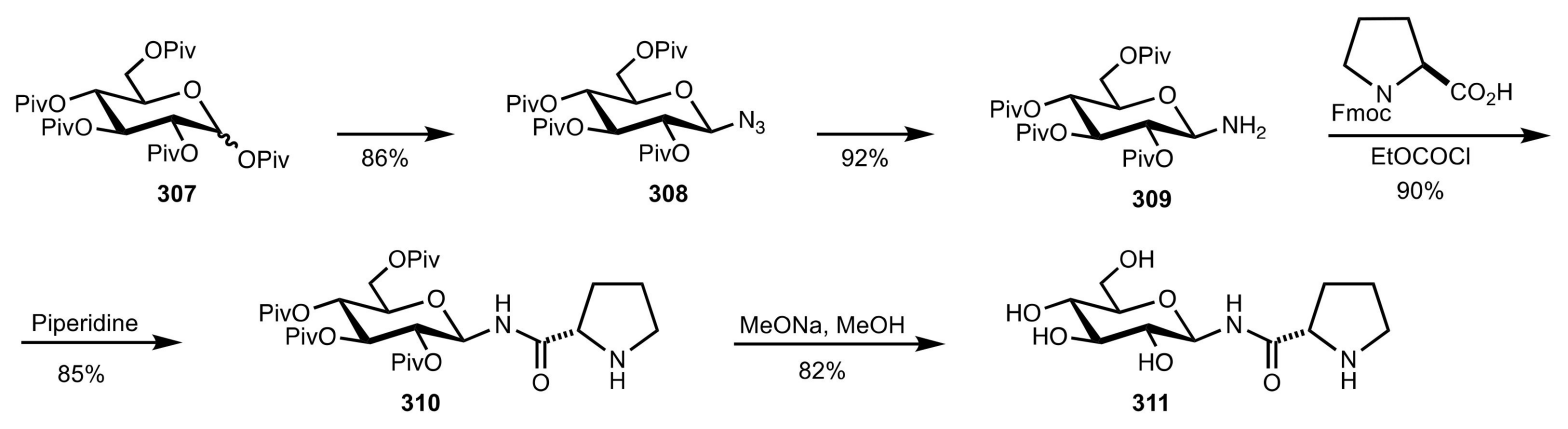
The synthesis of the sugar prolinamide organocatalysts **310** and **311**.

In 2013, Zhang and co-workers described [151] the preparation of the fully *O*-protected sugar prolinamide **310** and its free-OH derivative **311** (Scheme 85) from the known pivaloated glucosylamine **309**. Regrettably, the synthesis of the latter from the penta-*O*-pivaloyl d-glucopyranose **307** was not reported in the article [151] and the bibliographic references quoted therein did not deal with the preparation and analytical data of sugars **307–309**.

**Scheme 86:**
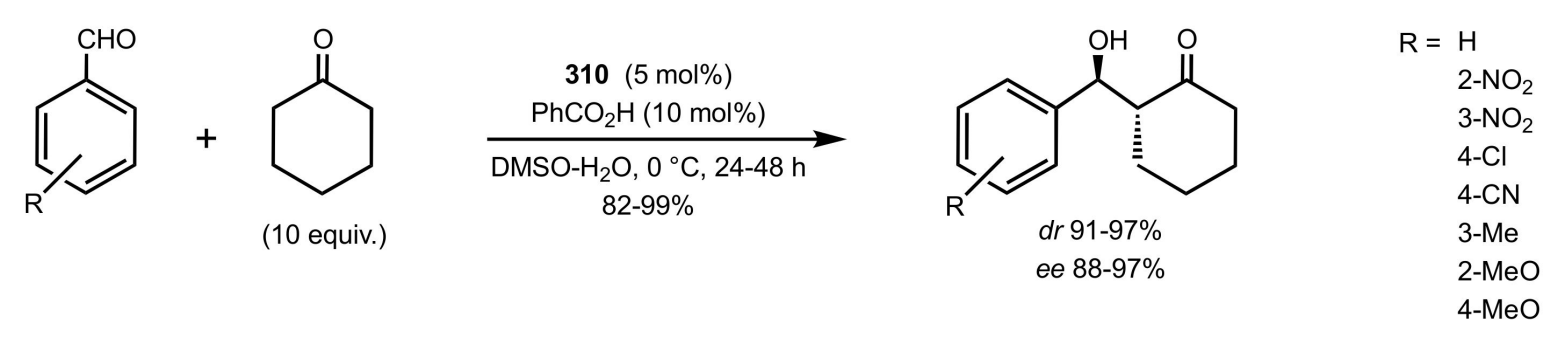
Aldol reactions catalysed by **310**.

The model aldol reaction between 4-nitrobenzaldehyde and cyclohexanone was conducted in various solvents and temperatures. Since the best results were observed when **310** was used as organocatalyst, this sugar prolinamide was employed to promote the aldol reaction of other aromatic aldehydes in DMSO-water at 0 °C (Scheme 86). In each case, the *anti* aldol was obtained in high yield and excellent stereoselectivity.

**Scheme 87:**
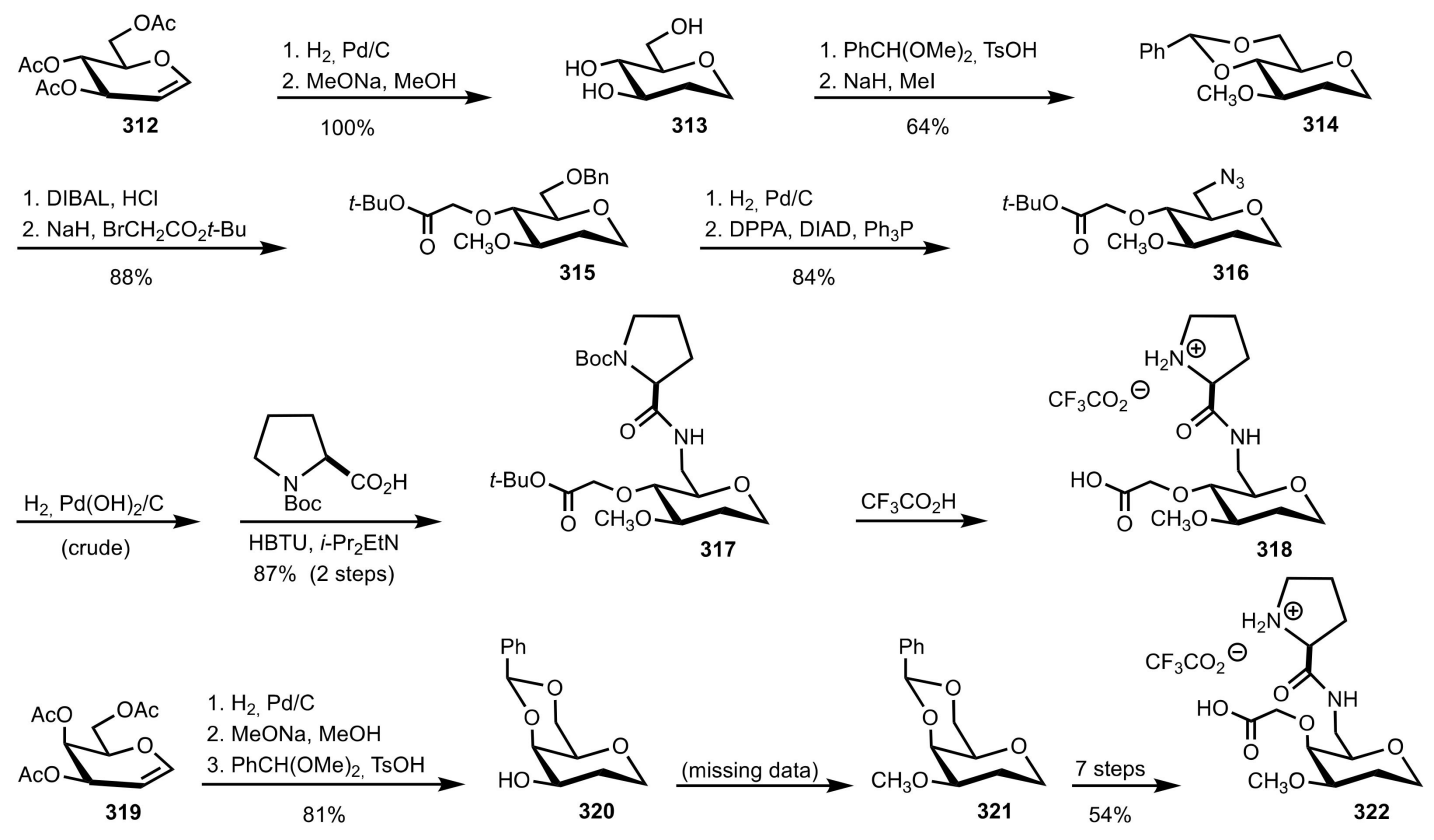
The synthesis of the sugar prolinamide organocatalysts **318** and **322**.

In 2017, Martín and co-workers reported [152] the synthesis and organocatalytic application of 14 sugar prolinamides, called “hybrid dipeptides”, containing an l- or d-proline moiety. The catalyst **318** (Scheme 87) was prepared from the known [153] 1,2-dideoxy-glucopyranose derivative **314**, in turn, obtained from the commercially available tri-*O*-acetyl-d-glucal **312** by hydrogenation, deacetylation by transesterification, benzylidenation and methylation. Regioselective reductive opening of the benzylidene ring afforded the corresponding 6-benzyl-4-hydroxy sugar that was alkylated to give **315**.

Debenzylation of the latter, followed by a Mitsunobu reaction with diphenylphosphoryl azide (DPPA), diisopropyl azodicarboxylate (DIAD) and triphenylphosphine, gave **316**. Reduction of the azide group to primary amine and coupling with *N*-Boc-l-proline led to the sugar prolinamide **317** that was isolated and characterized. The actual catalyst, i.e., **318**, was obtained by treatment with trifluoroacetic acid [152].

The d-*galacto* analogue **322** (Scheme 87) was obtained [152] via the same reaction sequence starting from **321**, although the experimental procedure for the synthesis of the latter compound as well as its analytical and spectral data were never described by Martín or other researchers. On the other hand, the synthesis of its precursor **320** from commercial tri-*O*-acetyl-d-galactal **319** was reported in 2008 by Ernst and co-workers [154].

**Scheme 88:**
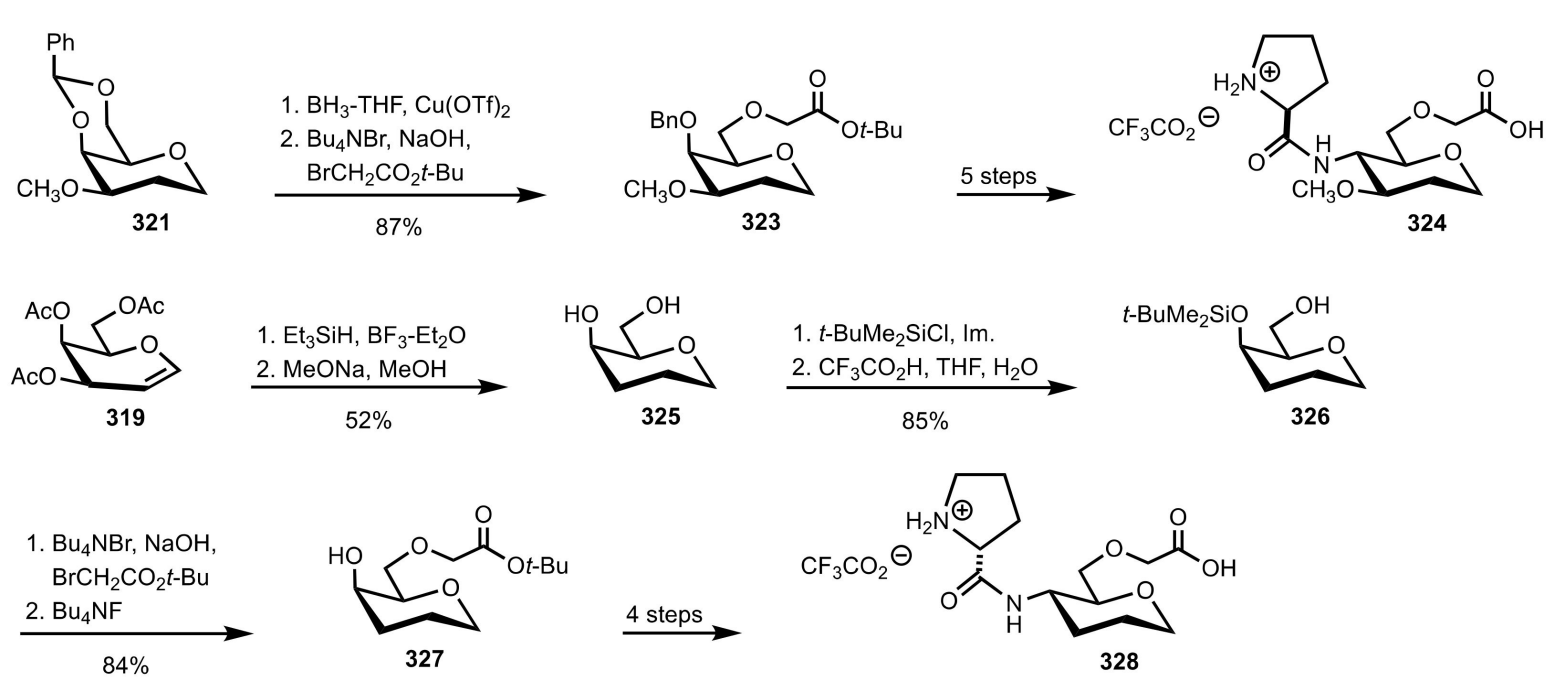
The synthesis of the sugar prolinamide organocatalysts **324** and **328**.

In order to install the proline unit at the 4-position, the benzylidene sugar derivative **321** (see Scheme 87) was treated with borane and copper(II) triflate to give the 4-benzyl-6-hydroxy sugar that was alkylated with *t*-butyl bromoacetate under phase-transfer conditions to afford **323** (Scheme 88). The usual reaction sequence allowed the preparation [152] of the organocatalyst **324** featuring an inversion of configuration at C-4 due to the Mitsunobu azidation.

The 1,2,3-trideoxy sugar prolinamide **328** (Scheme 88) was synthesised [152] from the known [155] 4-*O*-silylated sugar **326**, in turn prepared by deoxygenation of d-galactal **319** and deacetylation by transesterification to give the diol **325** as described [156]. Bis-silylation of the latter and chemoselective acid hydrolysis afforded [155] **326,** which was alkylated under phase-transfer conditions and desilylated to form **327** in good yield. The above illustrated reaction sequence allowed obtaining the organocatalyst **328** carrying a d-proline moiety.

**Scheme 89:**
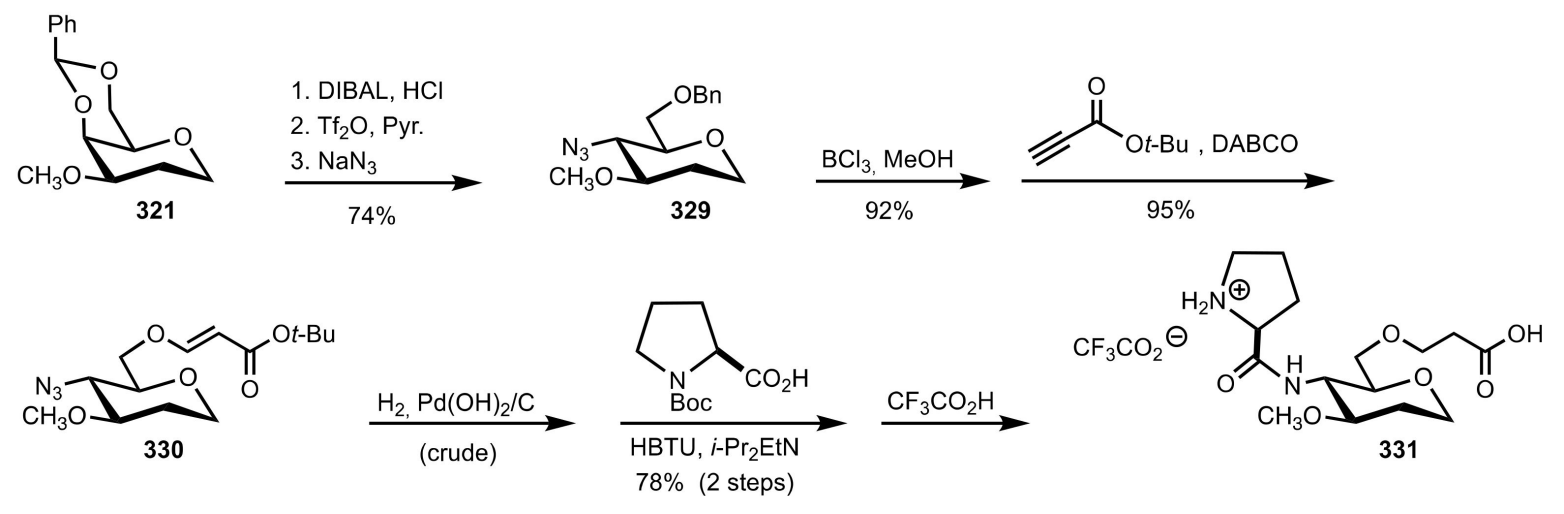
The synthesis of the sugar prolinamide organocatalyst **331**.

In the same article [152], Martín and co-workers also reported the synthesis of sugar prolinamides, such as **331** (Scheme 89), featuring a three-carbon chain carboxylic acid instead of the shorter acetic acid unit found in all the previously shown compounds (compare **324** in Scheme 88 with **331** in Scheme 89). The 4,6-*O*-benzylidene sugar **321** (see Scheme 88) was treated with DIBAL and HCl to give the 6-*O*-benzyl-4-hydroxy derivative that was activated as triflate and reacted with sodium azide to give **329**.

The benzyl ether of the latter was removed with BCl_3_ and the resulting alcohol coupled with *t*-butyl propiolate in the presence of 1,4-diazabicyclo[2.2.2]octane (DABCO) to afford the enol ether **330**. Reduction of the azide and alkene functions, followed by coupling with *N*-Boc-l-proline gave, after deprotection with trifluoroacetic acid, the organocatalyst **331**.

**Figure 25:**
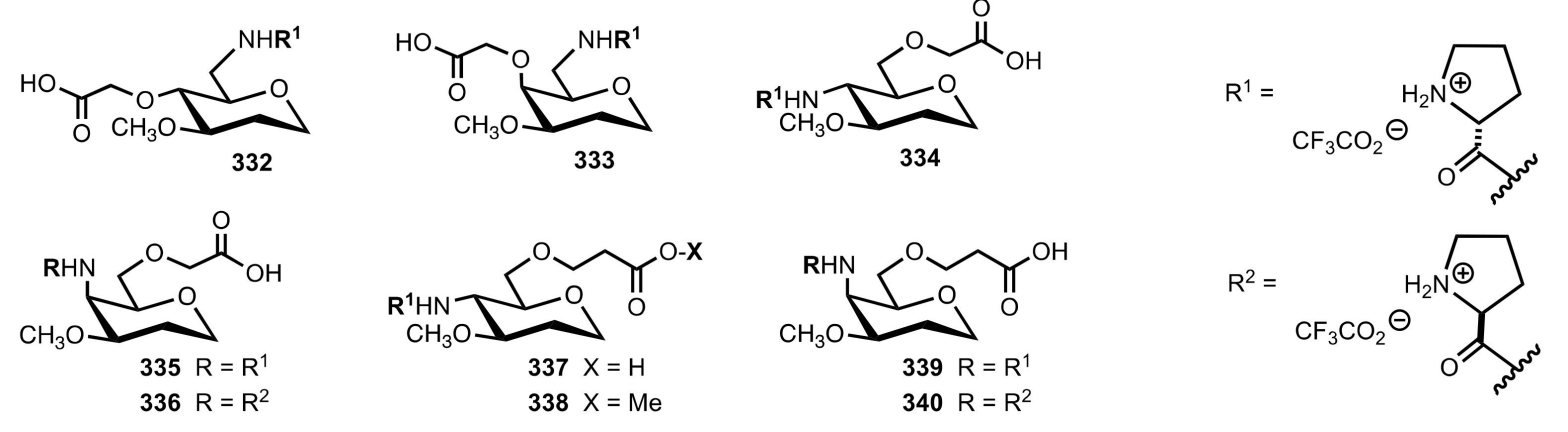
Other sugar d- and l-prolinamide organocatalysts prepared by Martín and co-workers.

The application of the synthetic approaches outlined in Scheme 87, Scheme 88 and Scheme 89 to different sugar substrates and/or the use of d-proline instead of l-proline, allowed the synthesis [152] of other sugar prolinamides (**332–340**, Figure 25).

The activity of the 14 sugar prolinamide organocatalysts (as trifluoroacetate salts) was screened by performing the Michael addition of propanal to *trans*-nitrostyrene in various solvents and in the presence of a base (*N*-methylmorpholine) to neutralize the ammonium salt [152]. The *syn* adduct was always obtained as the major compound, and the absolute configuration of the two stereocenters was related to the d- or l-proline moiety linked to the organocatalyst.

**Scheme 90:**
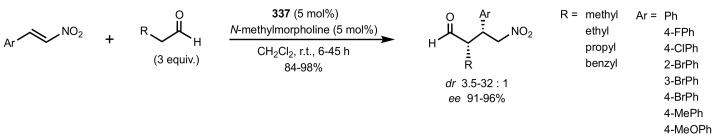
The Michael additions catalysed by **337**.

Since the best results were shown by **337** (see Figure 25), this compound was employed to catalyse the Michael additions at room temperature of four aliphatic aldehydes to differently substituted nitrostyrene derivatives (Scheme 90). The corresponding *syn* adduct was isolated in high yield and stereoselectivity. The authors performed also a theoretical conformational analysis for the organocatalysts **337** and **340** and found that the lowest-energy conformation of the former displayed a folded structure where the d-proline and the carboxylic acid moieties were in close contact. On the other hand, the lowest-energy conformation of **340** showed an unfolded structure where the l-proline and the carboxylic acid were far apart.

**Scheme 91:**
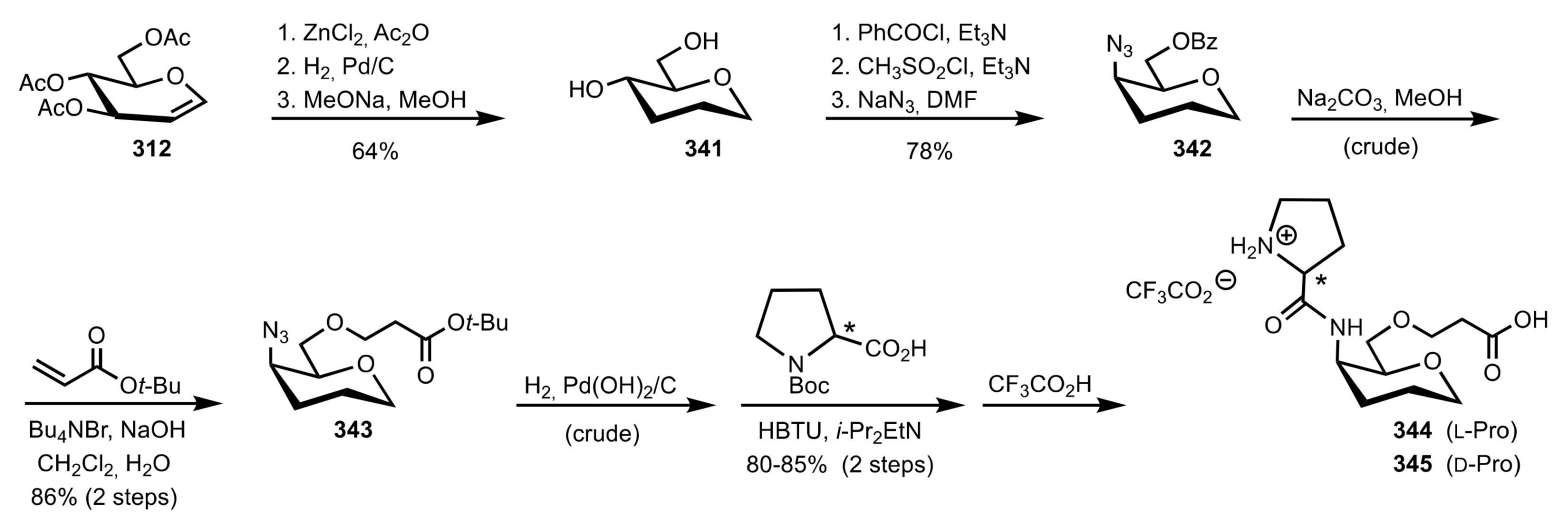
The synthesis of the sugar prolinamide organocatalysts **344** and **345**.

Aiming to modify the folded-unfolded conformations of the sugar prolinamides, Martín and co-workers described, two years later [157], the synthesis of analogues of the organocatalysts **337**, **339** and **340** (see Figure 25) lacking the methoxy group at the C-3 position. The compounds **344** and **345** (Scheme 91) were prepared from the diol **341**, easily obtained [158] by isomerization of the d-glucal **312**, hydrogenation and deacetylation by transesterification.

After regioselective benzoylation of the primary alcohol of **341** and activation of the secondary alcohol as mesylate, the diester was treated with sodium azide to afford the known azido-ester **342** [159]. Methanolysis of the benzoate ester and 1,4-conjugate addition of the resulting alcohol to *t*-butyl acrylate under phase transfer conditions gave **343** [157]. Reduction of the azide to amine function and coupling with *N*-Boc-d- or l-proline led, after *O*- and *N*-deprotection with trifluoroacetic acid, to the stereoisomers **344** and **345**.

**Scheme 92:**
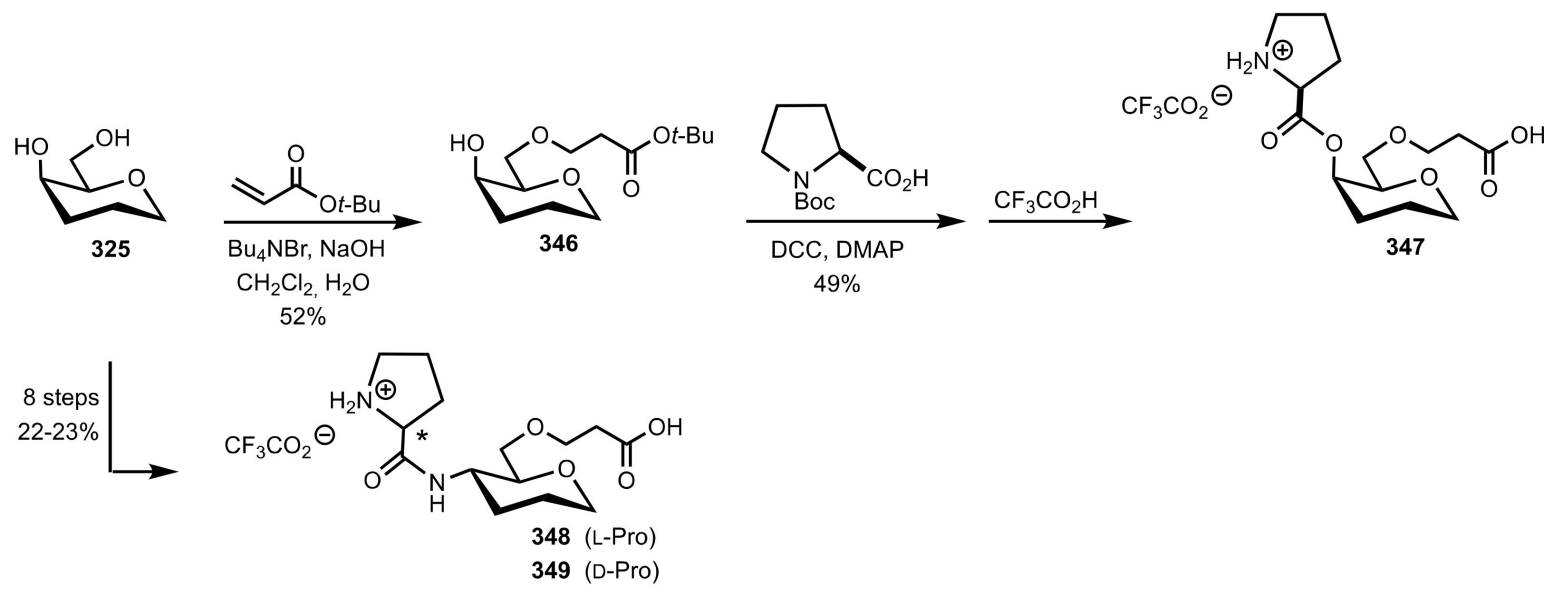
Syntheses of the organocatalysts **347–349**.

Application of the above-mentioned reaction sequence to the diol **325** (see Scheme 88) allowed preparation of the couple of stereoisomers **348** and **349** (Scheme 92) [157]. On the other hand, the 1,4-conjugate addition of **325** to *t*-butyl acrylate gave the alcohol **346** that was reacted with *N*-Boc-l-proline in the presence of DCC to afford, after deprotection, the sugar-proline ester organocatalyst **347** (Scheme 92).

The organocatalysts **344**, **345** (Scheme 91) and **347–349** (Scheme 92) were used to promote the Michael addition of propanal to *trans*-nitrostyrene in the presence of an equimolar amount of *N*-methylmorpholine. These experiments clearly showed that the new organocatalysts were more efficient than the previously reported analogues carrying a 3-methoxy group onto the pyranose unit. Moreover, amongst them, the sugar prolinamides **344** and **349** not only afforded the best enantioselectivity but were also complementary because one gave an enantiomer while the other provided the opposite enantiomeric adduct.

**Scheme 93:**
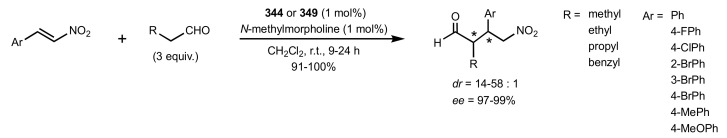
The Michael additions catalysed by **344** or **349**.

The use of the methyl ester of **344** (not shown) led to failure, thus, proving that the free carboxylic acid moiety is essential for the organocatalytic activity. Finally, the sugar-l-proline ester **347** (Scheme 92) was found to be less efficient than its l-prolinamide analogue **344**. Thus, the Michael additions of aliphatic aldehydes to substituted nitrostyrenes were performed [157] at room temperature in the presence of 1 mol% of the organocatalysts **344** and **349** (Scheme 93). The *syn* adducts were isolated in excellent yield, diastereo- and enantioselectivity, the sugar l-prolinamide **344** affording the *2R,3S* adduct and the sugar d-prolinamide **349** leading to the *2S,3R* enantiomer.

## 5. Variously Functionalized Carbohydrates

### 5.1. Sugar Pyrrolidines

**Scheme 94:**
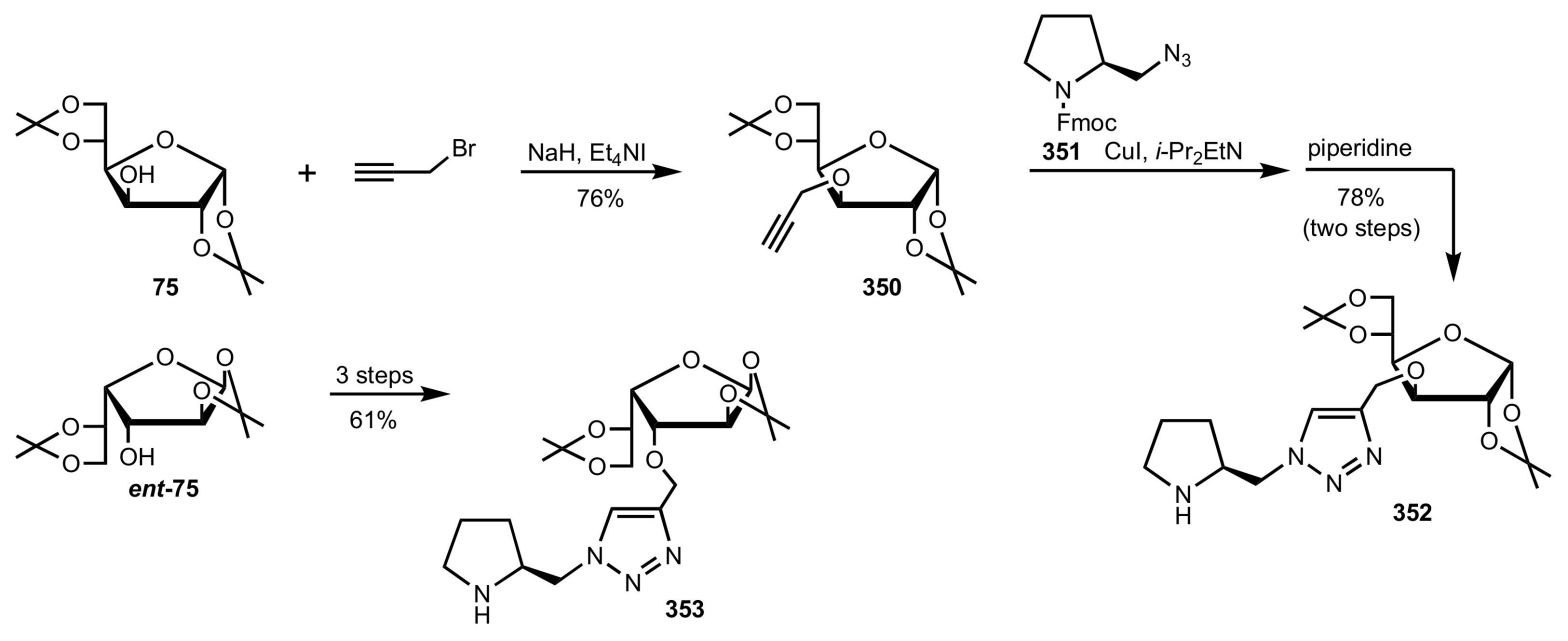
The synthesis of the sugar-pyrrolidine organocatalysts **352** and **353**.

With the aim to develop new organocatalysts featuring a chiral substituted pyrrolidine ring, Wang, Zhang and their co-workers synthesised [160] the sugar-pyrrolidines **352** and **353** from commercially available 1,2:5,6-di-*O*-isopropylidene-α-d- and l-glucofuranose **75** and ***ent*-75**, respectively (Scheme 94). The propargylation of **75** gave the alkyne **350** that was coupled with the Fmoc-protected azide **351**, prepared from the corresponding l-prolinol as described [161], through the copper-mediated azide-alkyne cycloaddition (CuAAC) to afford, after removal of the carbamate protecting group, **352** with a good yield.

**Scheme 95:**
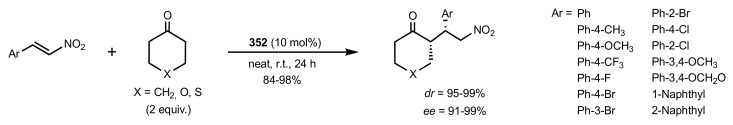
The Michael additions catalysed by **352**.

In the model Michael addition of cyclohexanone to nitrostyrene, it was found that the organocatalyst **352** gave a higher yield and stereoselectivity compared with its diastereomer **353**. Therefore, **352** was employed to catalyse the conjugate addition of cyclohexanone, tetrahydropyran-4-one and tetrahydrothiopyran-4-one to aromatic nitroalkenes in the absence of solvent (Scheme 95). The corresponding adducts were isolated in high yield and excellent diastereo- and enantioselectivity. On the other hand, the use of cyclopentanone and acetone as Michael donors led to the adducts in good yield but moderate stereoselectivity.

**Scheme 96:**
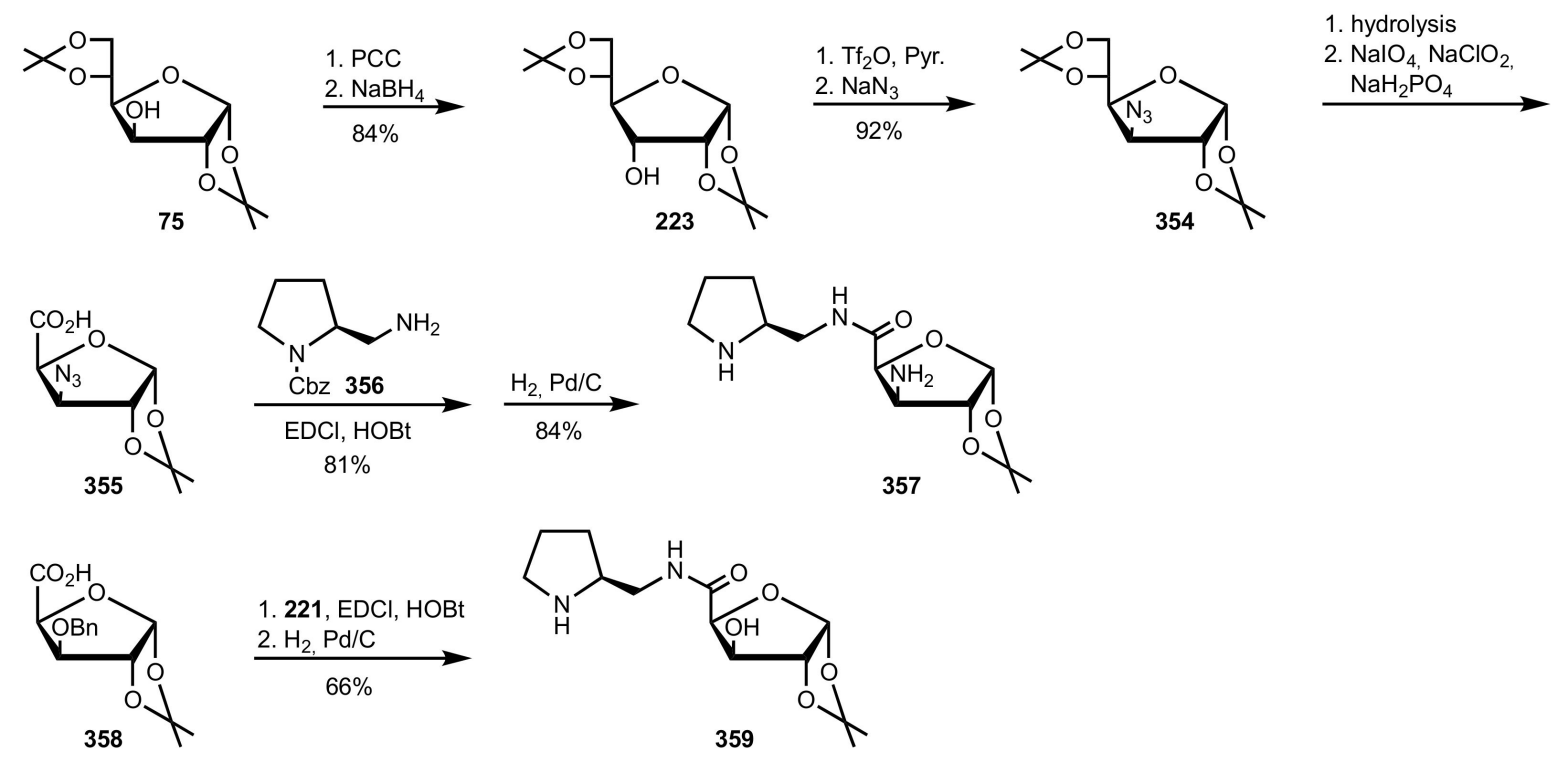
The synthesis of the sugar-pyrrolidine organocatalysts **357** and **359**.

Other sugar-pyrrolidine organocatalysts for the Michael addition were prepared in 2014 by Kumar and Balaji [162] starting from the d-xyluronic acid derivatives **355** and **358** (Scheme 96). The d-glucofuranose diacetonide **75** was first converted [163] into the epimer **223** by oxidation with pyridinium chlorochromate (PCC) and hydride reduction, and then triflation of **223** and a reaction with sodium azide afforded the 3-azido-d-glucofuranose **354** as described [163].

Regioselective acid hydrolysis of the latter and oxidation of the resulting diol afforded the acid **355**; however, the reaction conditions, yield and analytical data were not reported [164]. The azido-acid was coupled [162] with the amine **356** to give, after catalytic hydrogenation, the organocatalyst **357**. Unfortunately, the synthesis and analytical data of the proline-derived amine **356** were not provided [162]. The organocatalyst **359** was prepared in a similar way, but the synthesis and analytical data (or a correct bibliographic reference) of the acid **358** were missing [162].

**Scheme 97:**
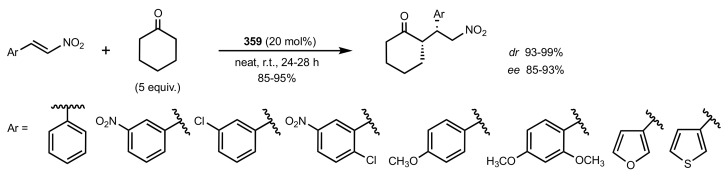
The Michael additions catalysed by **359**.

Preliminary experiments allowed establishing that the organocatalyst **359** was more efficient, in particular for the enantioselectivity, than the aminated analogue **357** and that the absence of solvent led to better results. Therefore, the addition of cyclohexanone to a series of aromatic or heteroaromatic nitroalkenes in the presence of 20 mol% of **359** was investigated (Scheme 97). In all cases, the yield and stereoselectivity were very high, whereas less satisfactory results were obtained when the cyclopentanone or acetone were used.

**Figure 26:**
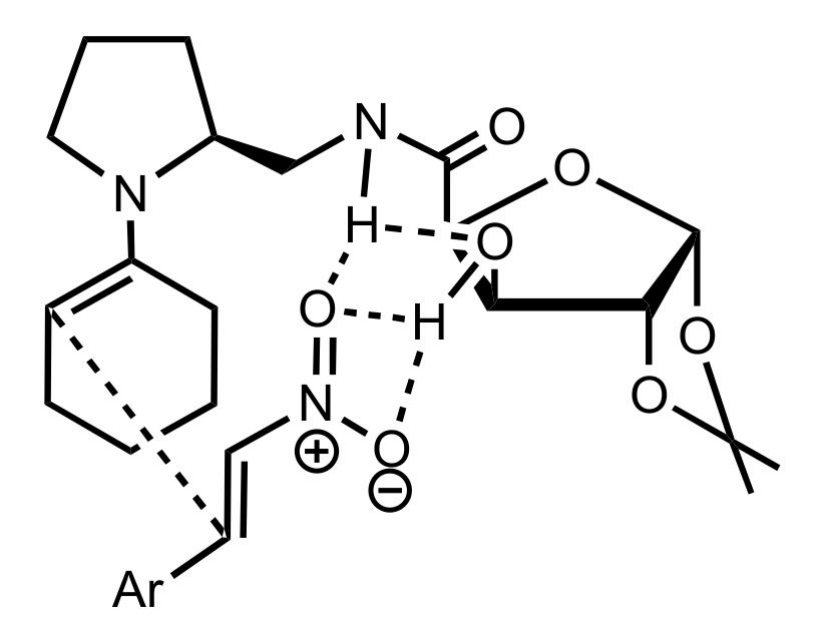
The proposed transition state for the Michael additions catalysed by **359**.

The stereochemical outcome of the reaction could be explained by considering the transition state shown in Figure 26. The secondary amine of the pyrrolidine moiety activates the ketone forming the corresponding enamine while the sugar unit controls the facial selectivity by stabilizing, together with the amide group, the nitroalkene through hydrogen bonding.

### 5.2. Sugar Pyridines

**Scheme 98:**
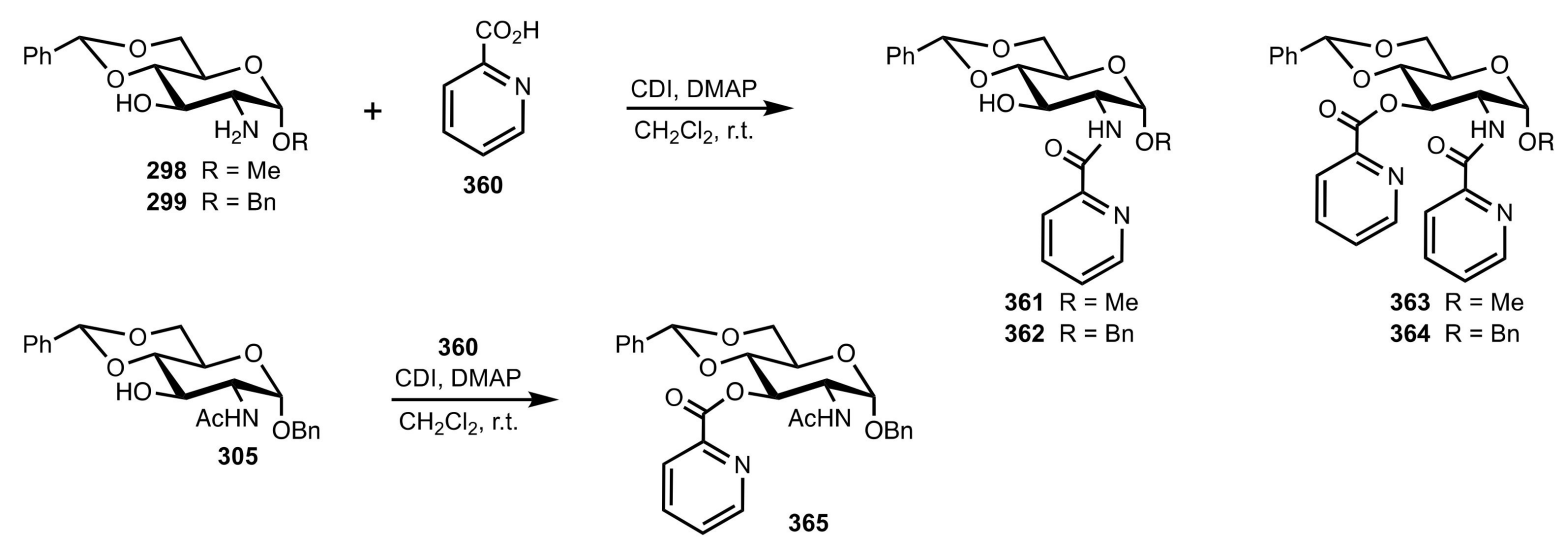
The synthesis of the sugar-pyridine organocatalysts **361–365**.

Some d-glucosamine-based mono- and dipyridine derivatives (**361–365**, Scheme 98) were exploited by Qian and co-workers to catalyse the reduction of imines with trichlorosilane [165]. It is worth noting that the same authors published, in the same year in the same journal, the synthesis of sugar aminoacids and their use as organocatalysts for the imine reduction (see Section 5.7). The organocatalysts **361–365** were prepared by coupling the methyl glycoside **298** and the benzyl glycosides **299** and **305** with picolinic acid **360** in the presence of carbonyldiimidazole (CDI) and 4-dimethylaminopyridine (DMAP). Unfortunately, although the target organocatalysts were fully characterized, the exact reaction and purification conditions were not indicated in the publication.

**Scheme 99:**
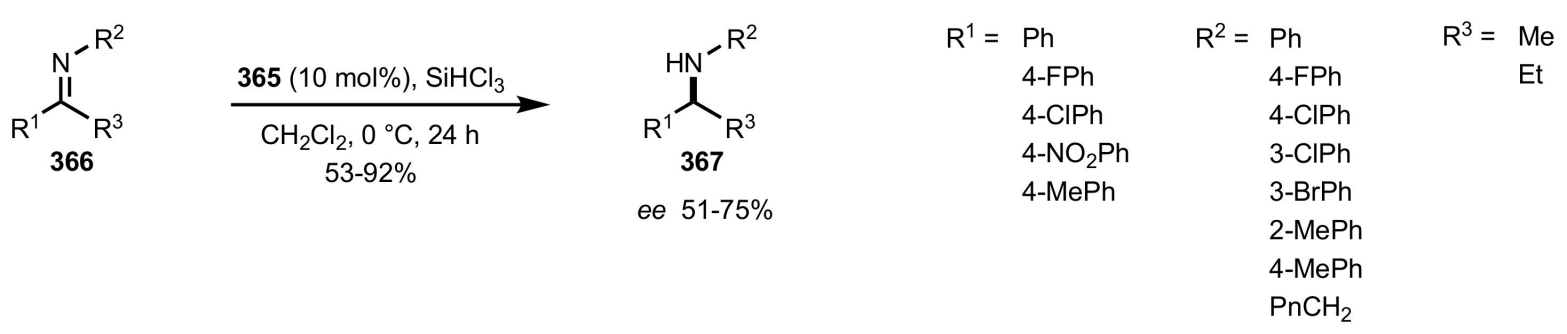
The reduction of imines catalysed by **365**.

A model imine reduction allowed establishing that the mono-pyridine derivative **365** afforded the best yield and enantioselectivity; therefore, this compound was then used to catalyse the reduction of a series of substituted imines **366** (Scheme 99). The (*S*)-configured amines **367** were obtained in moderate to good yield and moderate enantioselectivity.

**Figure 27:**
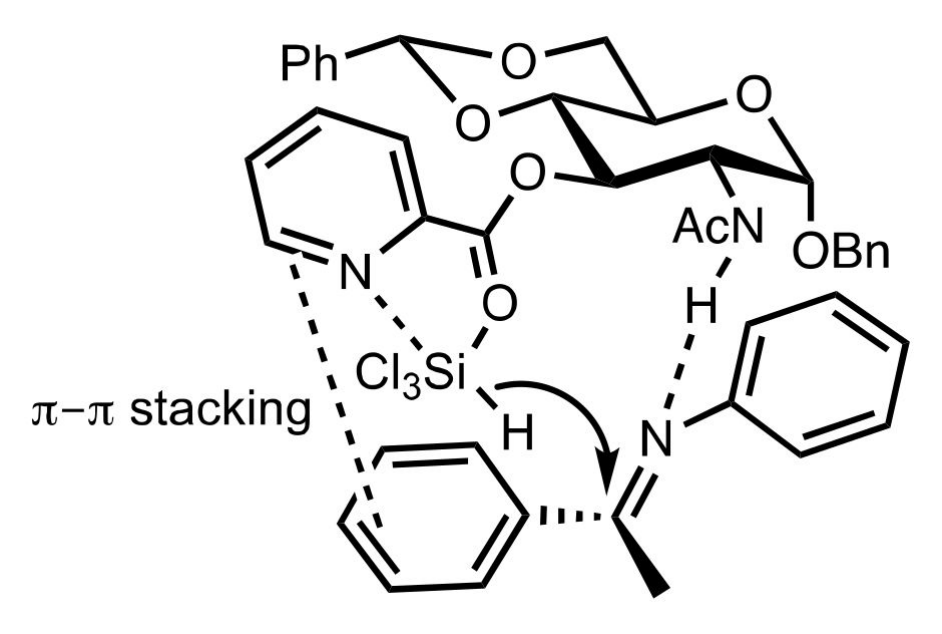
The proposed mechanism for the imines reduction catalysed by **365**.

The enantioselective organocatalysed reduction could proceed through the coordination of trichlorosilane to the carbonyl oxygen and the pyridine nitrogen of **365** together with the π–π stacking of the aromatic rings and activation of the imine by hydrogen bonding with the acetamide hydrogen (Figure 27).

### 5.3. Sugar Pyrimidines

**Scheme 100:**
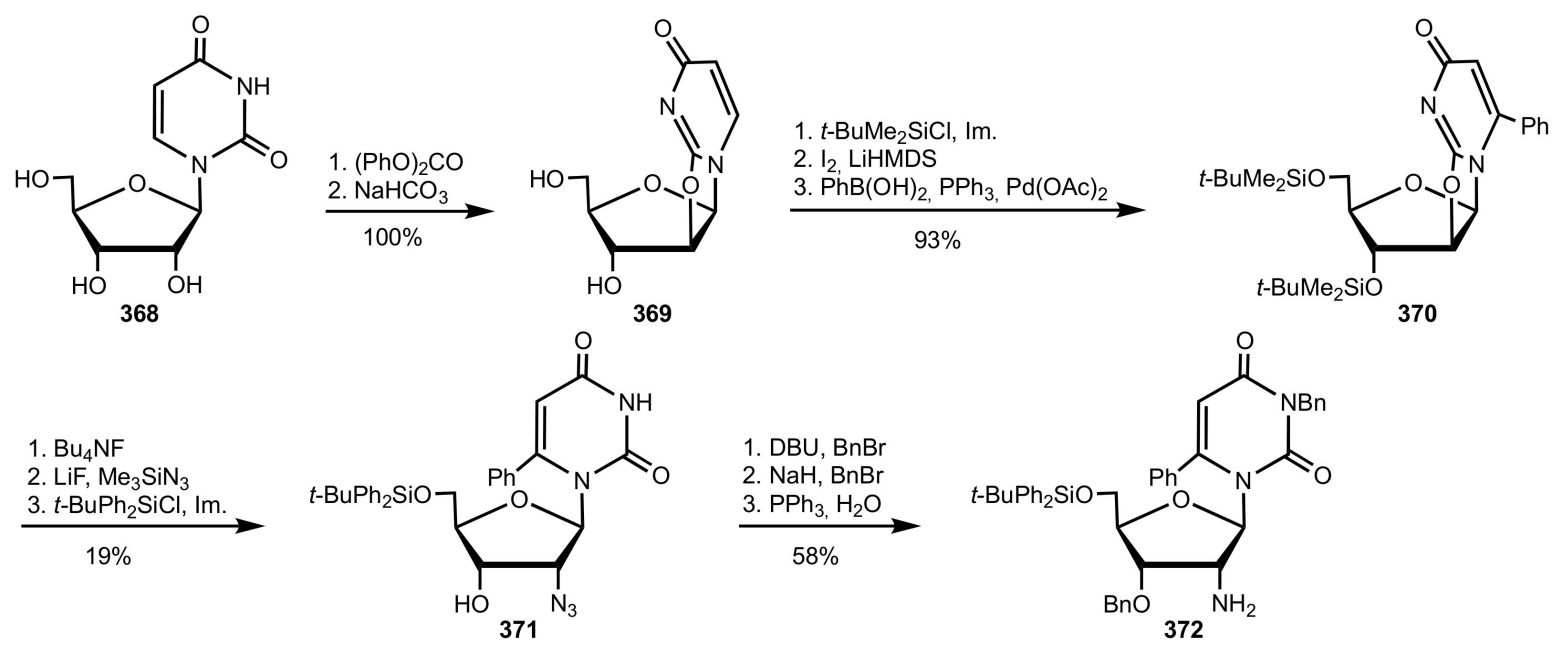
The synthesis of the 2′-aminouridine organocatalyst **372**.

In 2020, Yoshimura and co-workers developed new organocatalysts based on a 2-amino-ribonucleoside moiety in order to mimic the proline derivatives commonly used in organocatalysis [166]. The catalysts were designed to feature a primary amine function, bulk *O*-protecting groups and multiple asymmetric carbons (all located onto the d-ribofuranose unit). All the new compounds were synthesised from the cyclouridine **369** (Scheme 100), prepared by treatment of uridine **368** with diphenyl carbonate and NaHCO_3_ as described [167].

After silylation of the hydroxyl functions, the cyclouridine was iodinated at position 6 of the uracil moiety in the presence of a strong base and submitted to the Suzuki-Miyaura coupling with phenylboronic acid to give **370** with a high overall yield. The latter was desilylated and treated with lithium fluoride and trimethylsilyl azide to afford, after silylation of the primary alcohol, the 2-azido-uridine derivative **371**. To obtain the organocatalyst **372**, the benzylation of the pyrimidine nitrogen atom of **371** was conducted with 1,8-diazabicyclo[5.4.0]undec-7-ene (DBU) and benzyl bromide, while the hydroxyl group was benzylated with NaH and BnBr. Then, the azide was converted into the free amine by the Staudinger reaction.

**Scheme 101:**
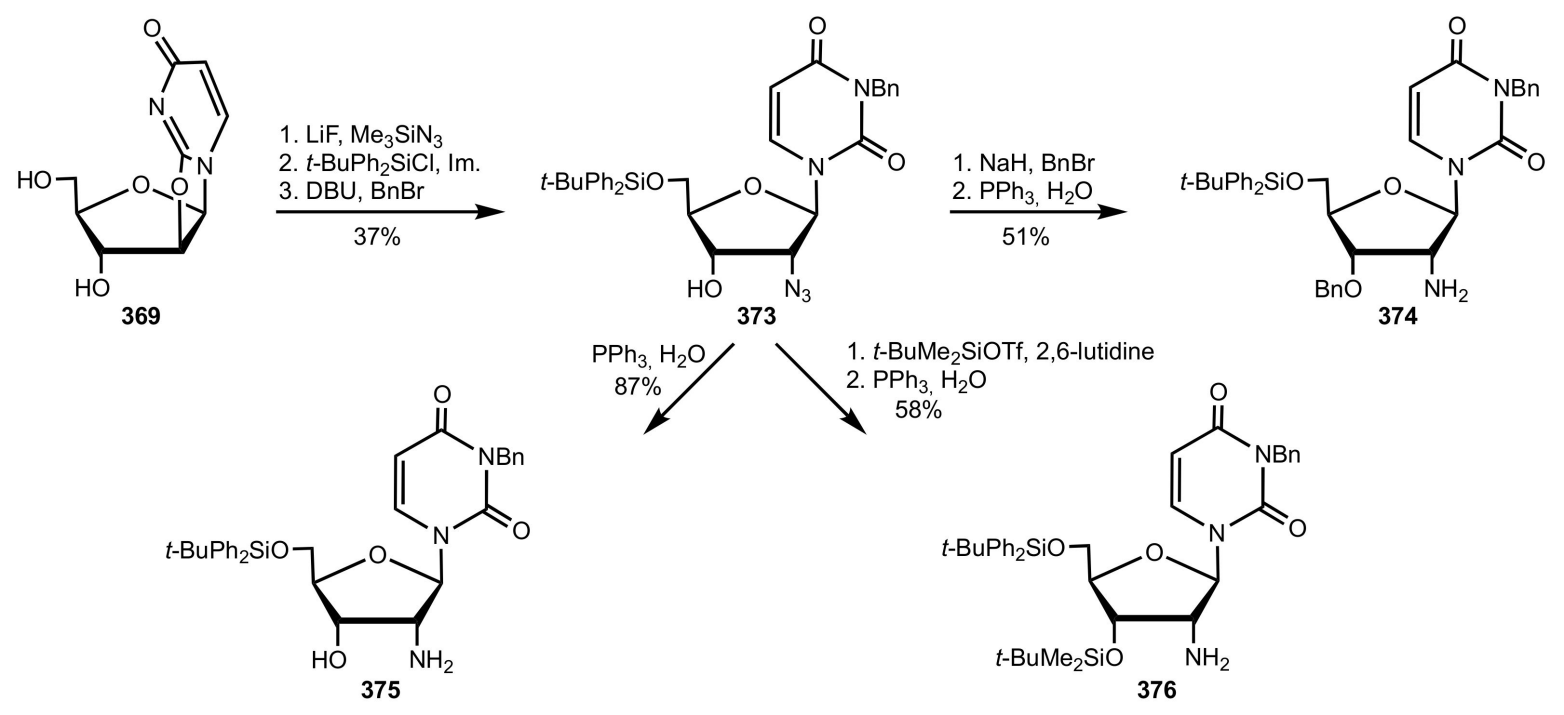
The synthesis of the 2′-aminouridine organocatalysts **374–376**.

The other organocatalysts were synthesised from the common intermediate **373**, in turn obtained from **369** by azidation at position 2 of the ribose moiety, silylation of the primary alcohol and chemoselective benzylation of the N-3 atom of the uracil moiety (Scheme 101). *O*-Benzylation of the azido-alcohol **373** and Staudinger reaction afforded **374,** whereas the direct conversion of the azide to amine group led to the amino-alcohol **375**. On the other hand, silylation of the secondary alcohol of **373** and the Staudinger reaction gave **376**.

**Scheme 102:**
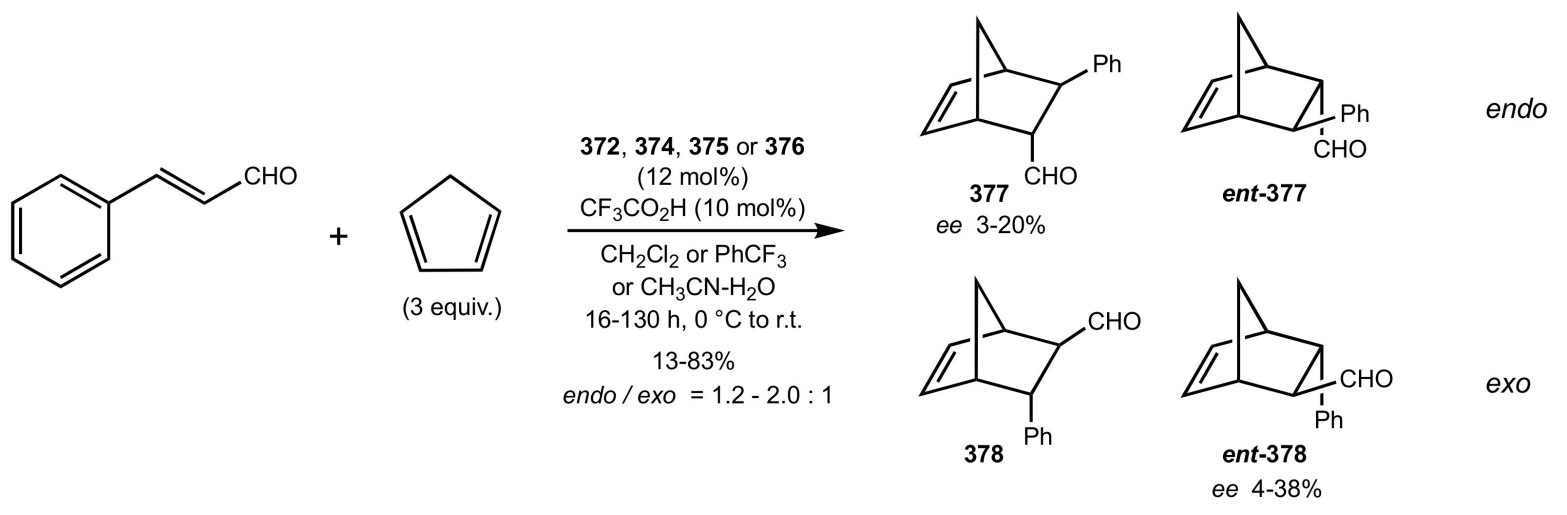
Diels–Alder reactions catalysed by **372** or **374–376**.

The four organocatalysts were employed to promote a single Diels–Alder condensation, i.e., the coupling of cinnamaldehyde with cyclopentadiene in different solvents (Scheme 102). In all cases, the *endo*/*exo* ratio of the adducts **377–378** was quite low (≤2:1) and the enantiomeric excess of the major enantiomer was very modest. The authors suggested that the disappointing stereochemical results arose from the high conformational flexibility of the prepared organocatalysts.

### 5.4. Sugar Tetrazoles

It is well known that α-aminoacids, e.g., l-proline, are efficient organocatalysts for the enantioselective aldol reaction but are poorly soluble in common solvents; therefore, these reactions must be performed in DMSO. Taking into account that the tetrazole ring is an isosteric replacement of the carboxylic acid moiety, the more soluble α-aminotetrazole derivatives have been used to catalyse aldol and Mannich reactions. In 2013, Nguyen Van Nhien and co-workers described [168] the synthesis of six carbohydrate-based α-aminotetrazoles starting from variously protected ketoaldoses (also known as aldosuloses).

**Scheme 103:**
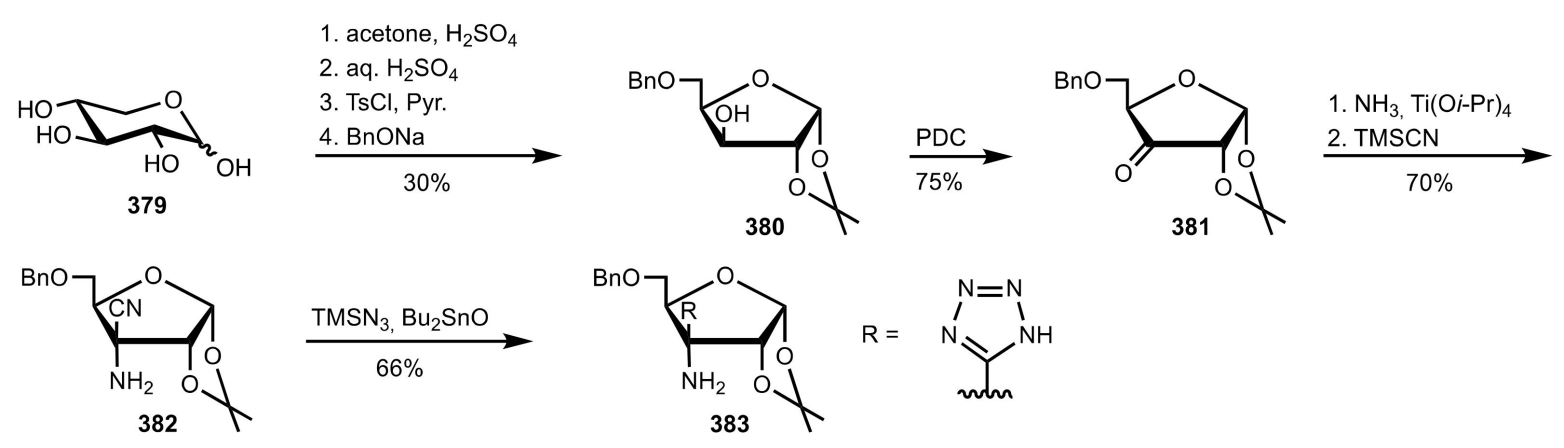
The synthesis of the sugar tetrazole organocatalyst **383**.

The first organocatalyst was prepared from d-xylose (**379**) through a known [169] reaction sequence constituted of bis-acetonation, regioselective hydrolysis to give the 1,2-*O*-isopropylidene derivative, tosylation at the primary position, displacement by the benzyloxide ion to afford the alcohol **380** and oxidation of the latter to the corresponding 3-ketoaldose **381** (Scheme 103). Treatment of **381** with methanolic ammonia in the presence of titanium tetra-isopropoxide and then with trimethylsilylcyanide as described [170] gave the α-aminonitrile **382** from which the α-aminotetrazole **383** was obtained [168] by reaction with trimethylsilylazide and dibutyltin oxide.

**Scheme 104:**
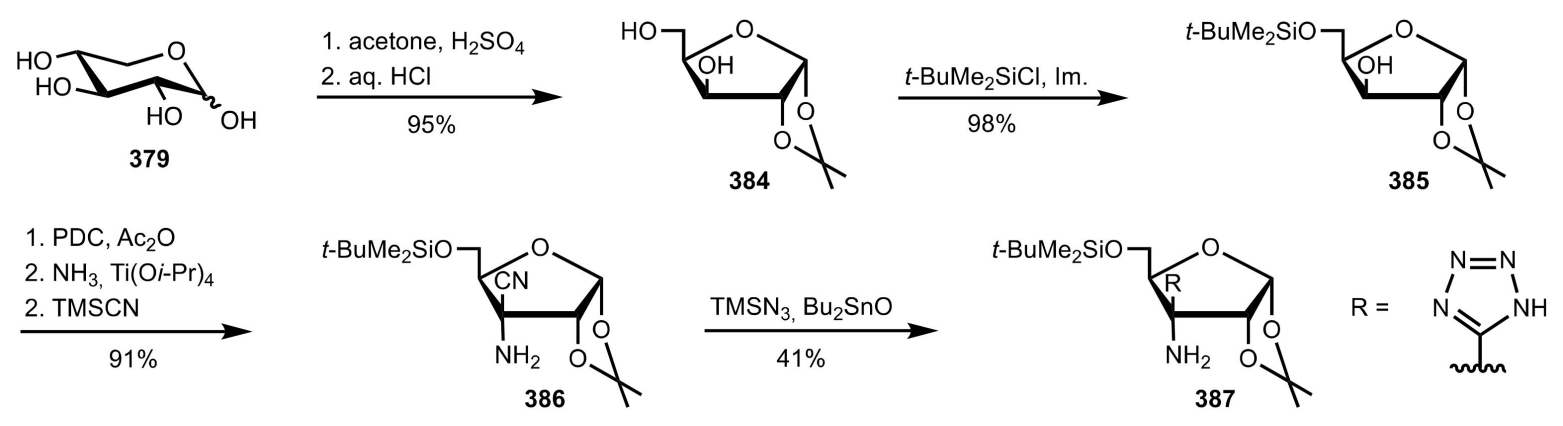
The synthesis of the sugar tetrazole organocatalyst **387**.

The α-aminotetrazole **387**, an analogue of **383** bearing a silyl instead of a benzyl group at the primary position, was prepared [168] from the known [171] alcohol **385**, in turn synthesised from d-xylofuranose mono-acetonide **384**, easily obtained by the bis-acetonation of d-xylose and regioselective hydrolysis (Scheme 104). Oxidation of **385** with pyridinium dichromate (PDC) and treatment of the crude ketoaldose with ammonia and titanium tetra-isopropoxide and then with TMSCN afforded the α-aminonitrile **386**. The latter gave the α-aminotetrazole **387** under the standard reaction conditions.

**Scheme 105:**
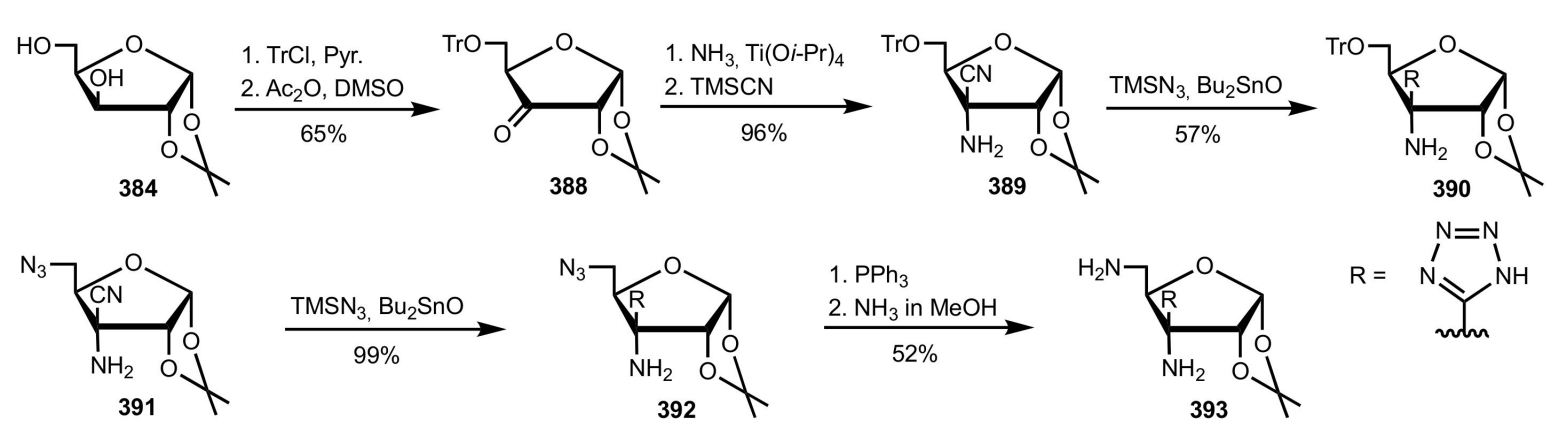
The synthesis of the sugar tetrazole organocatalysts **390**, **392** and **393**.

Another analogue of **383**, i.e., the 5-*O*-tritylated α-aminotetrazole **390**, was prepared from the known [172] 3-ketoaldose **388** exploiting the same reaction sequence described above (Scheme 105). The α-aminonitrile **389**, described in a previous article from the same research group [173], was converted into **390** in 57% yield [168]. The 5-azidated α-aminotetrazole **392** was prepared [168] from the α-aminonitrile **391**; however, the synthesis of the latter was not described, and references to previous works were also missing. The azido derivative **392** was also transformed [168] into the corresponding 5-amino α-aminotetrazole **393** by treatment with triphenylphosphine and ammonia.

**Scheme 106:**
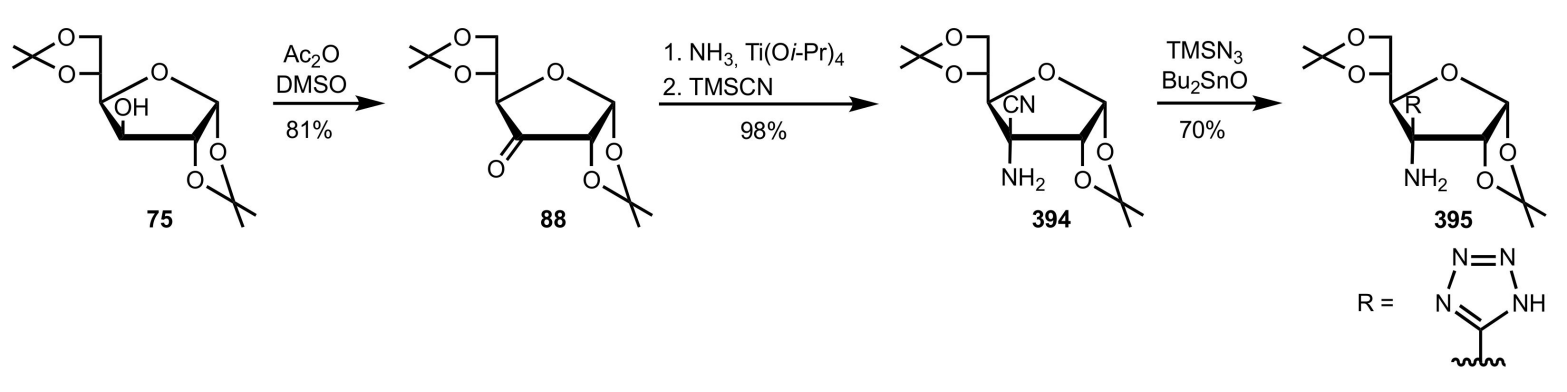
The synthesis of the sugar tetrazole organocatalyst **395**.

Finally, the α-aminotetrazole **395** was synthesised [168] from the known 3-ketoaldose **88** [174] via the already reported α-aminonitrile **394** [173] through the usual reaction sequence (Scheme 106).

**Scheme 107:**
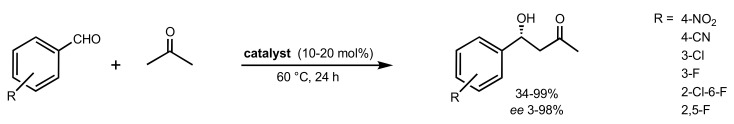
Aldol reaction catalysed by **383**, **387**, **390**, **392**, **393** or **395**.

The aldol reaction of acetone with 2-naphthaldehyde (not shown) and mono- or di-substituted benzaldehydes was conducted at 60 °C for 24 h in the presence of the six α-aminotetrazole derivatives (Scheme 107). The main aldol product, obtained with enantiomeric excess up to 98%, was always (*R*)-configured regardless of the employed organocatalyst, the only exception being the product formed from the 2-chloro-6-fluoro-benzaldehyde that featured the (*S*) configuration with all the organocatalysts. Moreover, it was found that most of the α-aminotetrazoles showed similarly good enantioselectivity, but **393** afforded the aldol adducts in low yield and enantiomeric excess (*ee* = 97–98% only in two cases).

**Figure 28:**
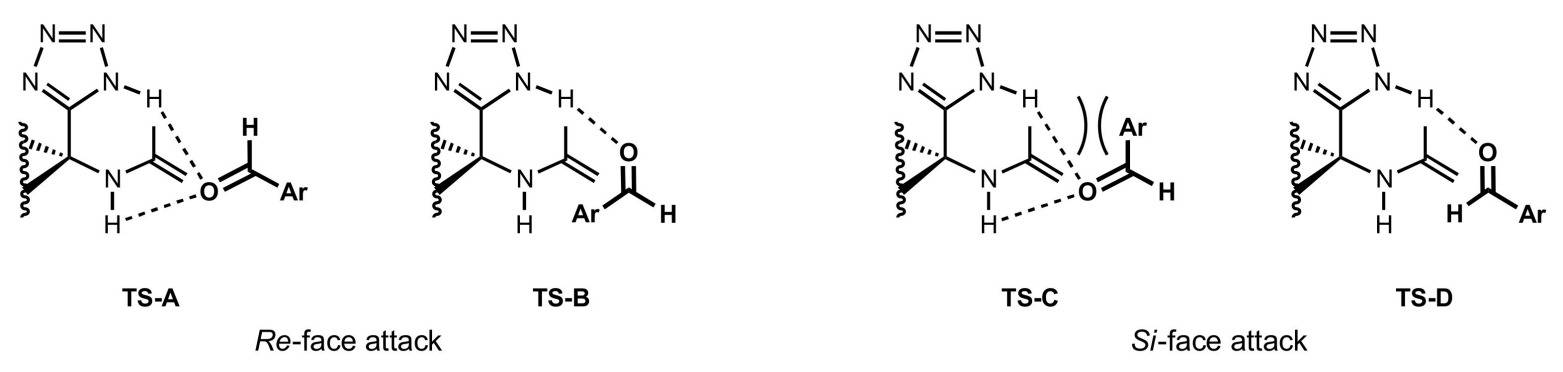
Transition states of the aldol reaction catalysed by the sugar tetrazole catalysts.

In order to rationalise the asymmetric induction observed with the six α-aminotetrazole organocatalysts, a density functional theory (DFT) study was performed. The results showed that the enamine formed between the sugar 3-amine and the acetone preferentially attacks the *Re* face of the aromatic aldehyde leading to the (*R*)-configured aldol as experimentally observed in most cases (Figure 28). In fact, the favoured transition state **TS-A** involves a second hydrogen bond (with the enamine NH group) and avoids the steric hindrance of the aryl ring with the enamine methyl group occurring in the transition state **TS-C**.

### 5.5. Sugar Diols

**Scheme 108:**
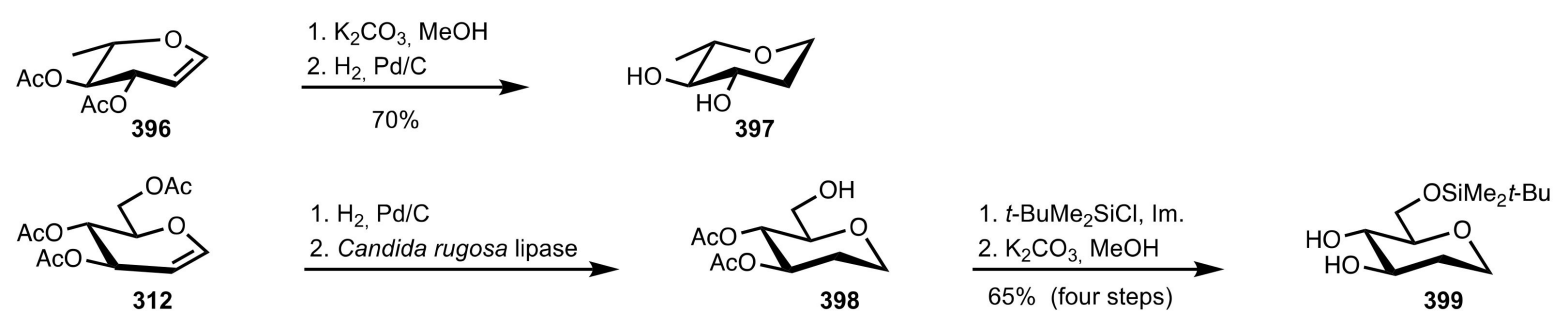
The synthesis of the sugar diol organocatalysts **397** and **399**.

Aiming to perform the enantioselective diboration of alkenes without the use of metal catalysts, Morken and co-workers exploited [175] easily available carbohydrate diols prepared from commercial glycals, such as the di-*O*-acetyl-l-rhamnal **396** and the tri-*O*-acetyl-d-glucal **312** (Scheme 108). To synthesize a couple of pseudo-enantiomeric *trans*-diequatorial-1,2-diols, the l-rhamnal **396** was deacetylated under basic conditions and then hydrogenated to give the l-deoxy-sugar **397**, which adopts a ^1^*C*_4_ chair conformation. Hydrogenation and selective deacetylation at the primary position of the d-glucal **312** afforded **398** that was silylated and fully deacetylated to give **399**, adopting the ^4^*C*_1_ chair conformation, with a 65% overall yield.

**Scheme 109:**
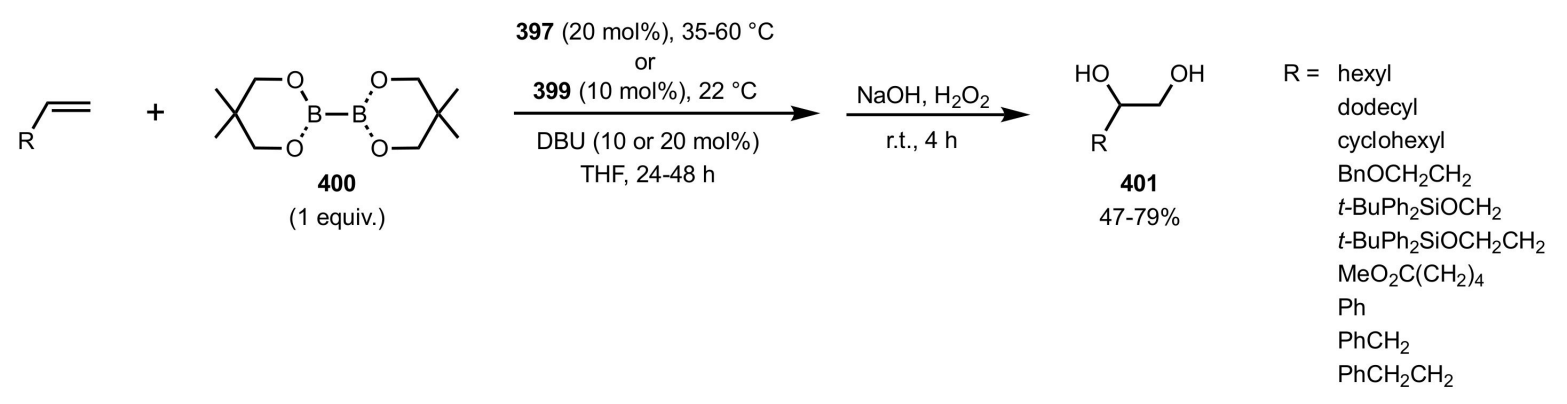
The diboration–oxidation of alkenes organocatalysed by sugar diols **397** and **399**.

A series of ten terminal alkenes were treated with neopentyl-glycol boronate **400** (B_2_(neo)_2_) in the presence of the organocatalyst **399,** and the resulting adducts directly oxidised with hydrogen peroxide to give the 1,2-diols **401** in moderate/good overall yield with an enantiomeric ratio up to 96:4 (Scheme 109). Five out of ten alkenes were submitted to the same reaction sequence using the organocatalyst **397**, which, however, proved to be less reactive because higher loading (20 mol%) and temperatures (35 or 60 °C) were required to obtain satisfactory yields.

On the other hand, **397** allowed for isolation of the diols **401** featuring exactly the opposite enantiomeric ratio in comparison with the corresponding diols prepared in the presence of **399**. It is worth noting that, in one case (tetradecene), the transformation was conducted on a 10 g scale. Finally, when an internal alkene (not shown) was employed as a substrate, the use of organocatalyst **397** or **399** led to the same inversion of enantiomeric ratio as observed for the terminal alkenes.

**Scheme 110:**
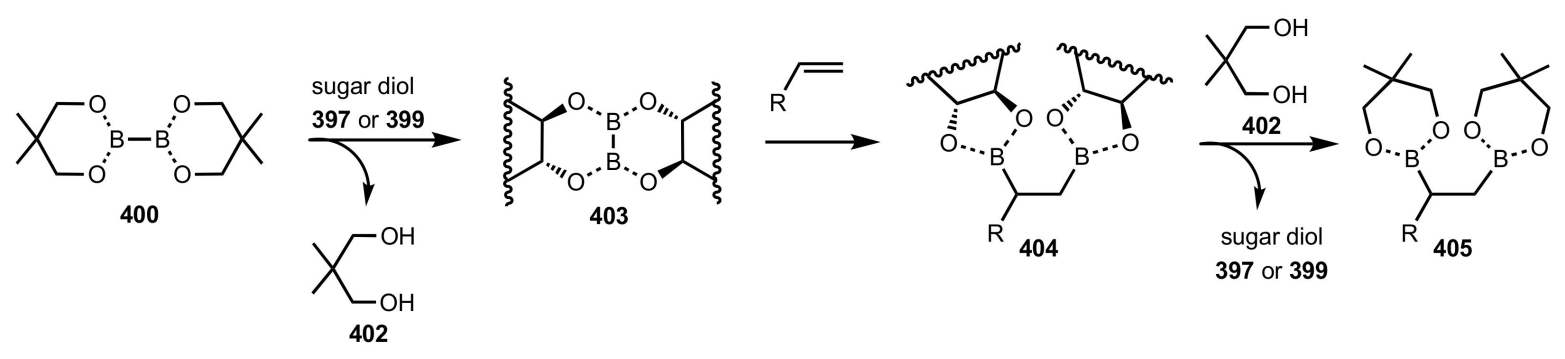
Mechanism of the diboration–oxidation of alkenes catalysed by **397** or **399**.

To explain the enantioselectivity observed for the diboration–oxidation of alkenes in the presence of the sugar diols, the authors suggested an exchange between the neopentyl-glycol moiety in B_2_(neo)_2_ **400** and the sugar organocatalysts **397** or **399** bearing a *trans*-diequatorial-1,2-diol group to form **403** (Scheme 110).

Such an exchange converts the more stable 1,1-bonded derivative **400**, featuring 120° O-B-O bond angles, into the more reactive 1,2-bonded diboron reagent **403** able to form a tetrahedral ate complex (four-coordinated boron centres), the intermediate that attacks the alkene to afford **404**. The subsequent exchange with the achiral neopentyl-glycol **402** leads to **405**, which can be oxidised to give the vicinal diol **401**, and recycles the chiral sugar diol **397** or **399**.

### 5.6. Sugar Carboxylic Acids

**Scheme 111:**
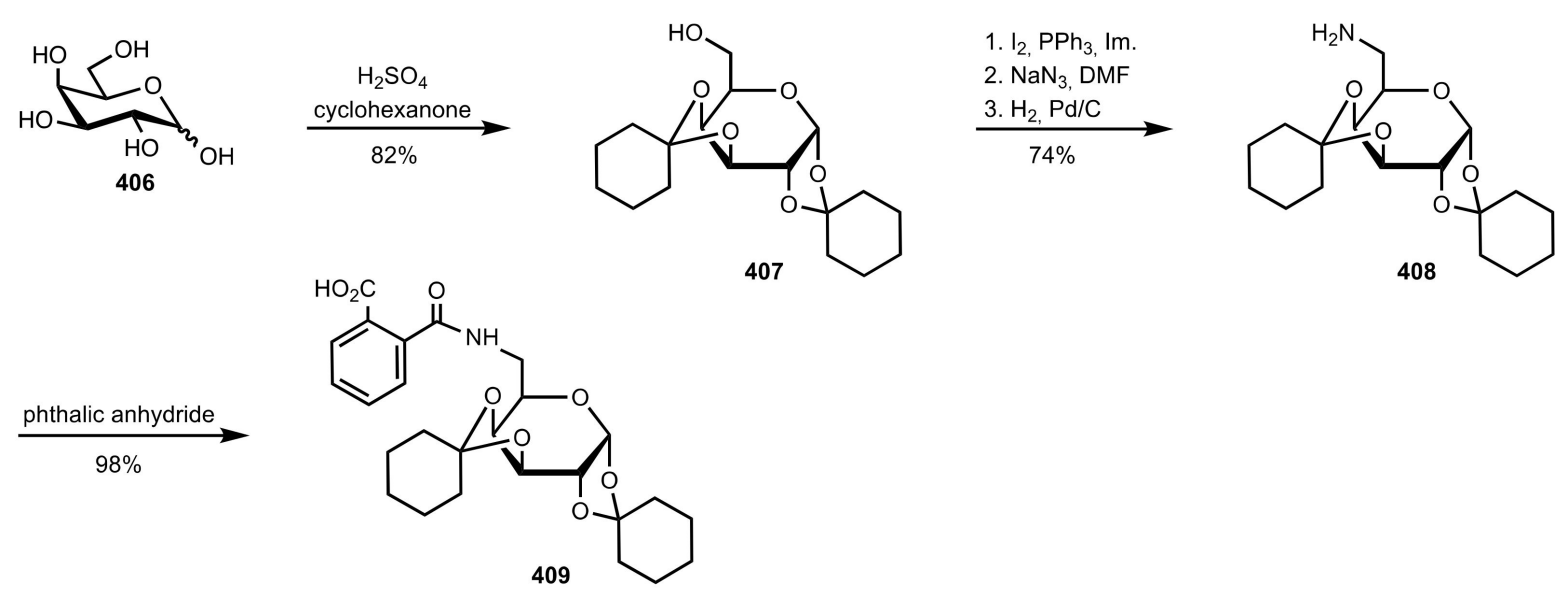
The synthesis of the sugar carboxylic acid organocatalyst **409**.

In order to catalyse the Michael-type addition of azlactones to divinyl-ketones, Amarante and co-workers prepared [176] the sugar-based carboxylic acid **409** starting from the d-galactose bis-ketal **407** synthesised as previously described [177] (Scheme 111). Then, the alcohol **407** was converted into the amine **408** by iodination, azidation and hydrogenation. Finally, treatment of **408** with phthalic anhydride led to the amide **409** bearing a free carboxylic acid function. The compounds **407–409** likely adopt a skew-boat and not a chair conformation as depicted in the article (see the relevant signals in their NMR spectra).

**Scheme 112:**
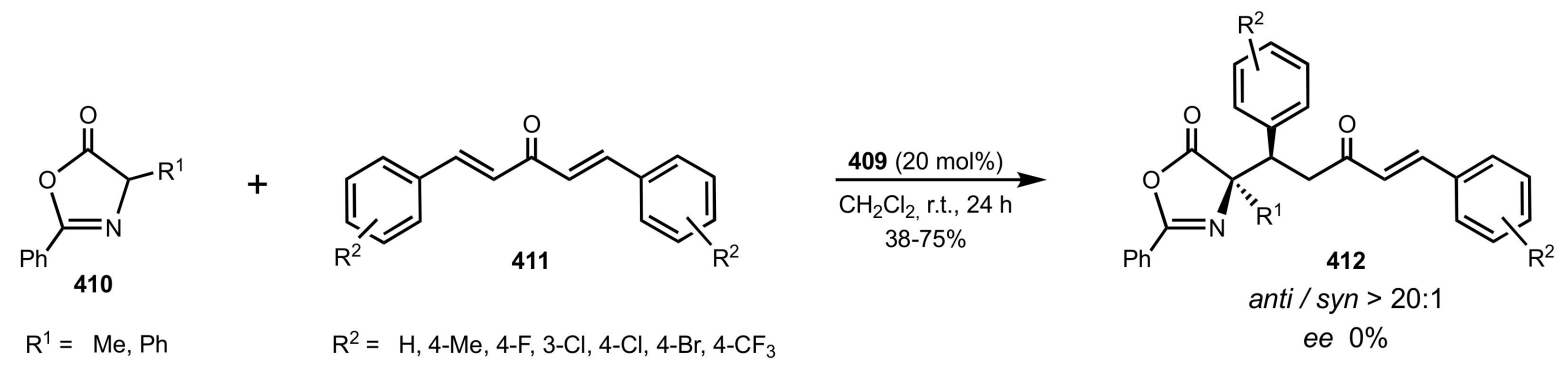
The Michael addition catalysed by **409**.

The addition of azlactones **410** to dibenzylideneacetone derivatives **411** in the presence of organocatalyst **409** at room temperature led to the mono-adduct **412** in variable yield but always high diastereoselectivity since the *anti/syn* ratio was higher than 20:1 (Scheme 112). However, in all cases, the compounds were obtained as racemic mixtures.

### 5.7. Sugar Aminoacids

**Scheme 113:**
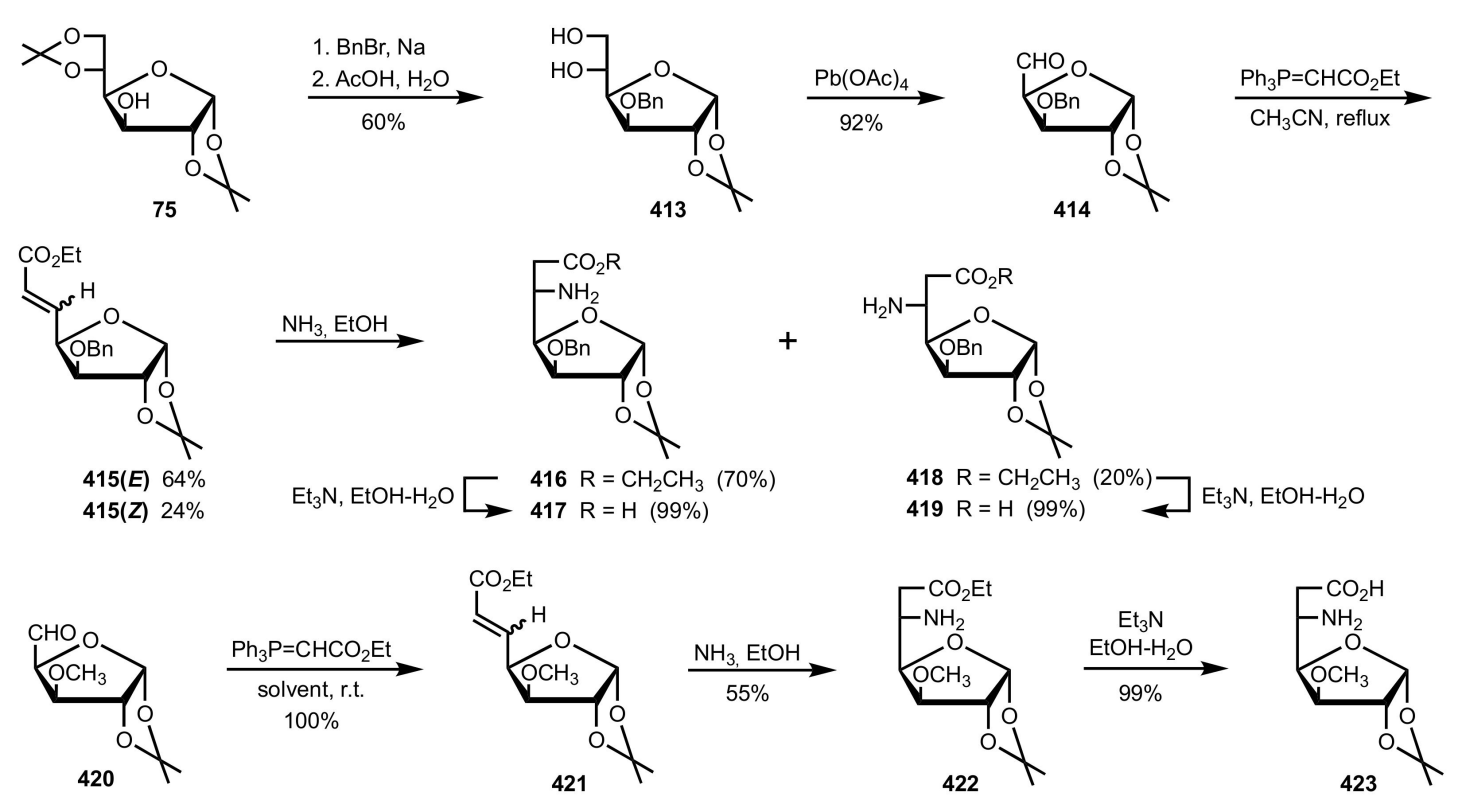
The synthesis of the sugar aminoacid organocatalysts **416**, **417**, **419** and **423**.

Tripathi and co-workers studied the aldol reaction of acetone with aromatic aldehydes in the presence of some sugar-based amino esters and amino acids as the organocatalysts [178]. These compounds were prepared by the 1,4-conjugate addition of ammonia to known sugar enoates **415** and **421** (Scheme 113). The 3-*O*-benzyl derivative **415** was obtained from the d-glucofuranose **75** by benzylation and selective hydrolysis of the 5,6-*O*-isopropylidene function to give the diol **413** [179] that was submitted to oxidative cleavage as previously described [180].

The Wittig reaction of the aldehyde **414** with a stabilized phosphorane afforded **415** as a mixture of *Z/E* isomers, which could be separated by column chromatography [181]. However, in previous papers on the same topic, Tripathi and co-workers performed the conjugate addition of amines to pure *E* and *Z* isomers and found that there was no improvement in stereoselectivity. Therefore, in the present work, the reaction with ammonia was carried out using an isomeric mixture of sugar enoate **415** to give the amino esters **416** and **418** in 70 and 20% isolate yield.

Basic hydrolysis of these esters led to the corresponding amino acids **417** and **419**, respectively. The same reaction sequence was applied to the 3-*O*-methyl sugar aldehyde **420**, which was converted into the known [182] conjugate ester **421** (the *Z/E* ratio depends on the solvent employed in the Wittig reaction) and then treated with ammonia in ethanol to give the (*S*)-configured amino ester **422** with a 55% isolated yield and the 5-epimer (25%). The compound **422** was not characterized but directly hydrolysed to afford the amino acid **423** in almost quantitative yield.

**Scheme 114:**
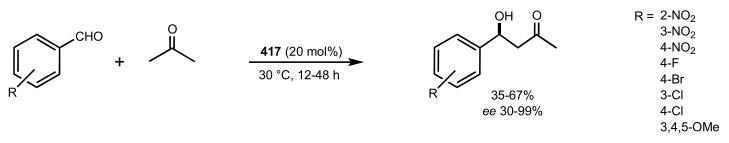
Aldol reactions catalysed by **417**.

The amino ester **416** and the three amino acids **417**, **419** and **423** were used as organocatalysts in the model aldol reaction between 3-nitrobenzaldehyde and acetone (reagent and solvent). It was found that the ester **416** did not catalyse the reaction at all even at 60 °C, whereas good results were obtained with the acid **417** (65% yield, *ee* = 90%). The amino acid **423** bearing a 3-*O*-methyl instead of a 3-*O*-benzyl group gave lower yield (55%) and poor enantiomeric excess (18%), while the (*R*)-configured amino acid **419** was found inactive at 30 °C but afforded the aldol in 55% yield and 68% *ee* at 60 °C. Therefore, only the amino acid **417** was employed in a series of aldol reactions of acetone with substituted benzaldehyde derivatives and 4-pyridinecarboxaldehyde (not shown) leading to the corresponding (*S*)-aldol with enantiomeric excess up to 99% (Scheme 114).

**Figure 29:**
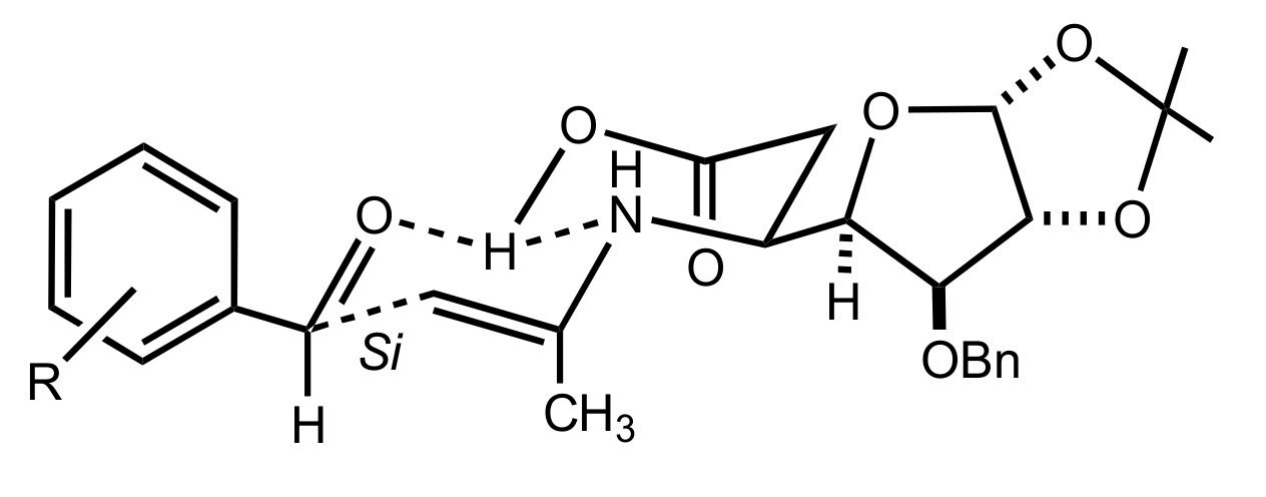
Favoured transition state leading to the (*S*)-configured aldol.

As in the case of proline-catalysed aldol reaction, the proposed mechanism involves the formation of an enamine between acetone and the amino acid catalyst and then its attack to the aldehyde from the *Si* face thanks to a bicyclic transition state stabilized by hydrogen bonds where the substituted phenyl ring is quasi-equatorial, i.e., avoiding the 1,3-diaxial interaction with the methyl group of the enamine (Figure 29).

**Scheme 115:**
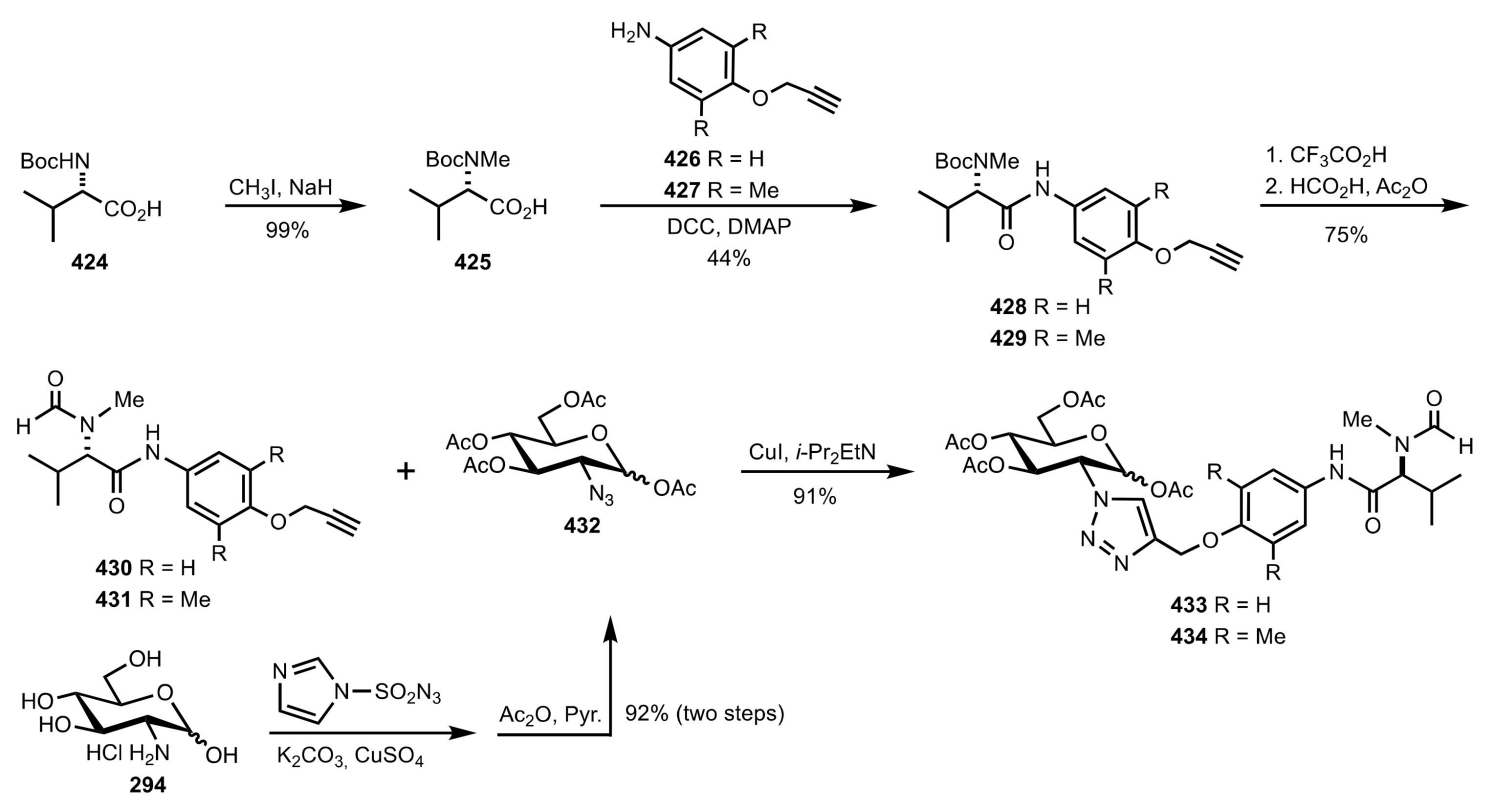
The synthesis of the sugar aminoacid organocatalysts **433** and **434**.

The Lewis-basic organocatalytic reduction of prochiral imines with trichlorosilane leads to chiral amines, which are useful building blocks for the synthesis of complex molecules. To this end, Qian and co-workers prepared [183] the two organocatalysts **433** and **434** in which a peracetylated glucose moiety was linked to the *N*-formyl-l-valine unit through a triazole ring (Scheme 115). The *N*-Boc-l-valine **424** was first *N*-methylated and then coupled under standard conditions to the aniline derivatives **426** and **427** bearing an *O*-propargyl group to afford the amides **428** and **429**.

The valine of these compounds was deprotected and transformed into the *N*-methyl-formamide derivatives **430** and **431**, which were submitted to the copper-mediated azide-alkyne cycloaddition (CuAAC) with the known 2-azido-2-deoxy-glucopyranose **432**, in turn prepared from the d-glucosamine hydrochloride (**294**) by a diazotransfer reaction using imidazole-1-sulfonyl azide in the presence of CuSO_4_ as described [184]. The resulting triazole derivatives **433** and **434** were isolated in high yield as ca. 2:1 mixtures of β- and α-d anomers.

**Scheme 116:**
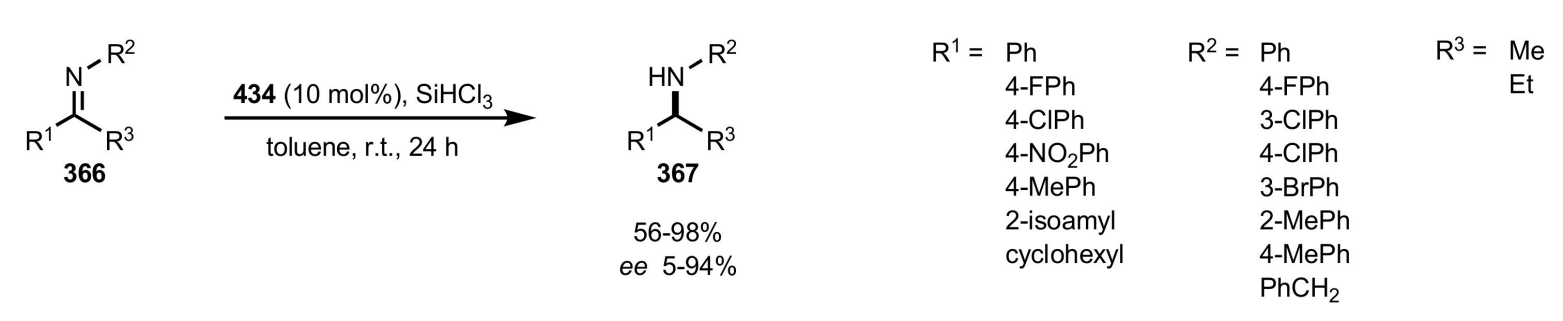
The reduction of imines catalysed by **434**.

The reduction of the *N*-phenyl-imine of acetophenone with trichlorosilane allowed selecting the best reaction conditions (room temperature in toluene) and to select the more efficient organocatalyst, i.e., **434**. Then, variously substituted imines **366** were submitted to the reduction in the presence of the latter catalyst to give the corresponding (*S*)-configured amines **367**, which were isolated in enantiomeric excess ranging from 5% to 94% (Scheme 116). The sugar-based organocatalyst **434** was also recovered by column chromatography and recycled five times. Some decrease in the yield but an almost unchanged enantiomeric excess even after five cycles were observed.

### 5.8. Sugar Imines and Iminium Salts

**Scheme 117:**
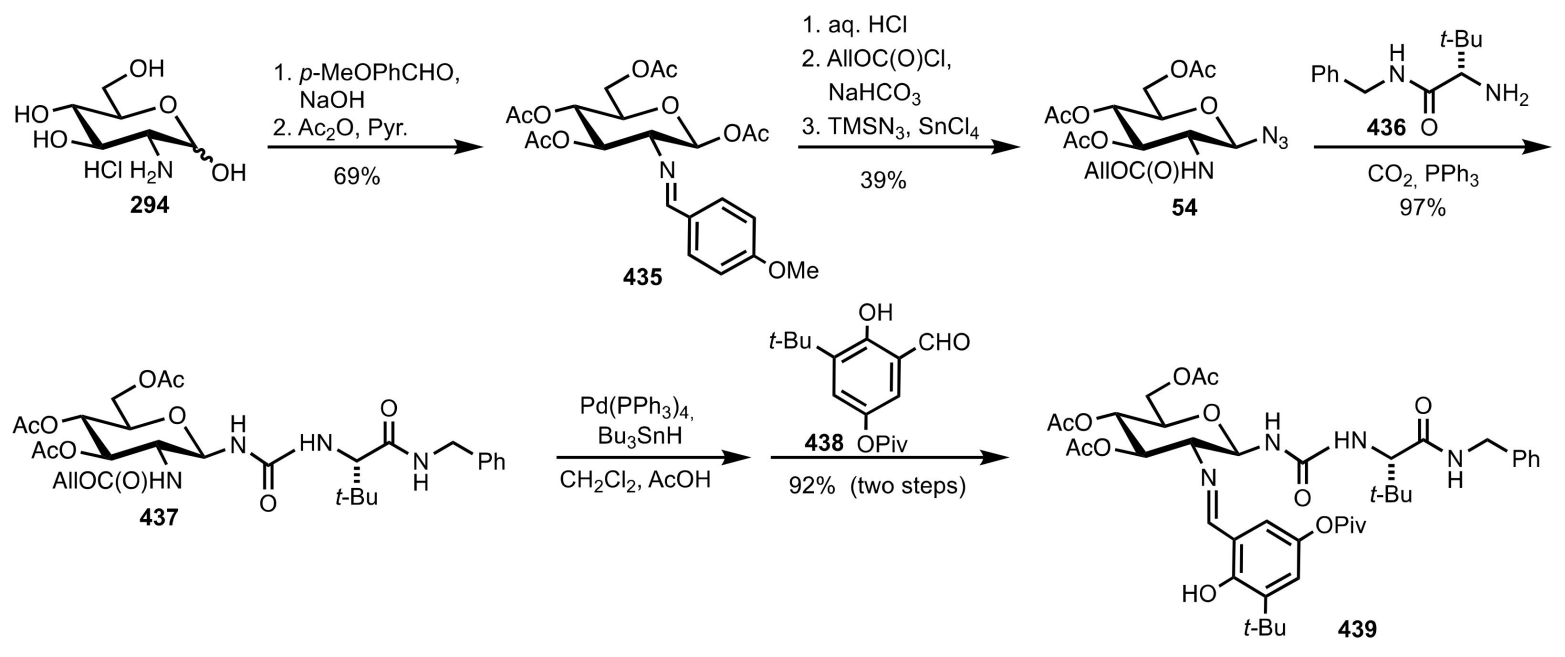
The synthesis of the sugar imine organocatalyst **439**.

Aiming to obtain new organocatalysts based on diaminated sugars instead of 1,2-*trans*-diamino-cyclohexane as reported by Sigman and Jacobsen [26], Kunz and co-workers described the preparation and use of several carbohydrates bearing imine and urea functions [185]. The d-glucosamine hydrochloride (**294**) was *N*-protected as imine and acetylated to obtain the tetra-acetate **435** as pure β-d-anomer (Scheme 117). Then, the imine was hydrolysed and the amine protected as allyloxycarbamate.

Upon treatment with trimethylsilyl azide (TMSN_3_) in the presence of a Lewis acid, the latter compound afforded the anomeric sugar azide **54** that was coupled with l-*t*-butyl-leucine amide **436** by means of a Staudiger–aza-Wittig reaction to give the urea **437** in almost quantitative yield. Upon Pd(0)-catalysed removal of the allyloxycarbonyl group, the resulting amine was condensed with salicylaldehyde **438** to afford the organocatalyst **439**.

**Figure 30:**
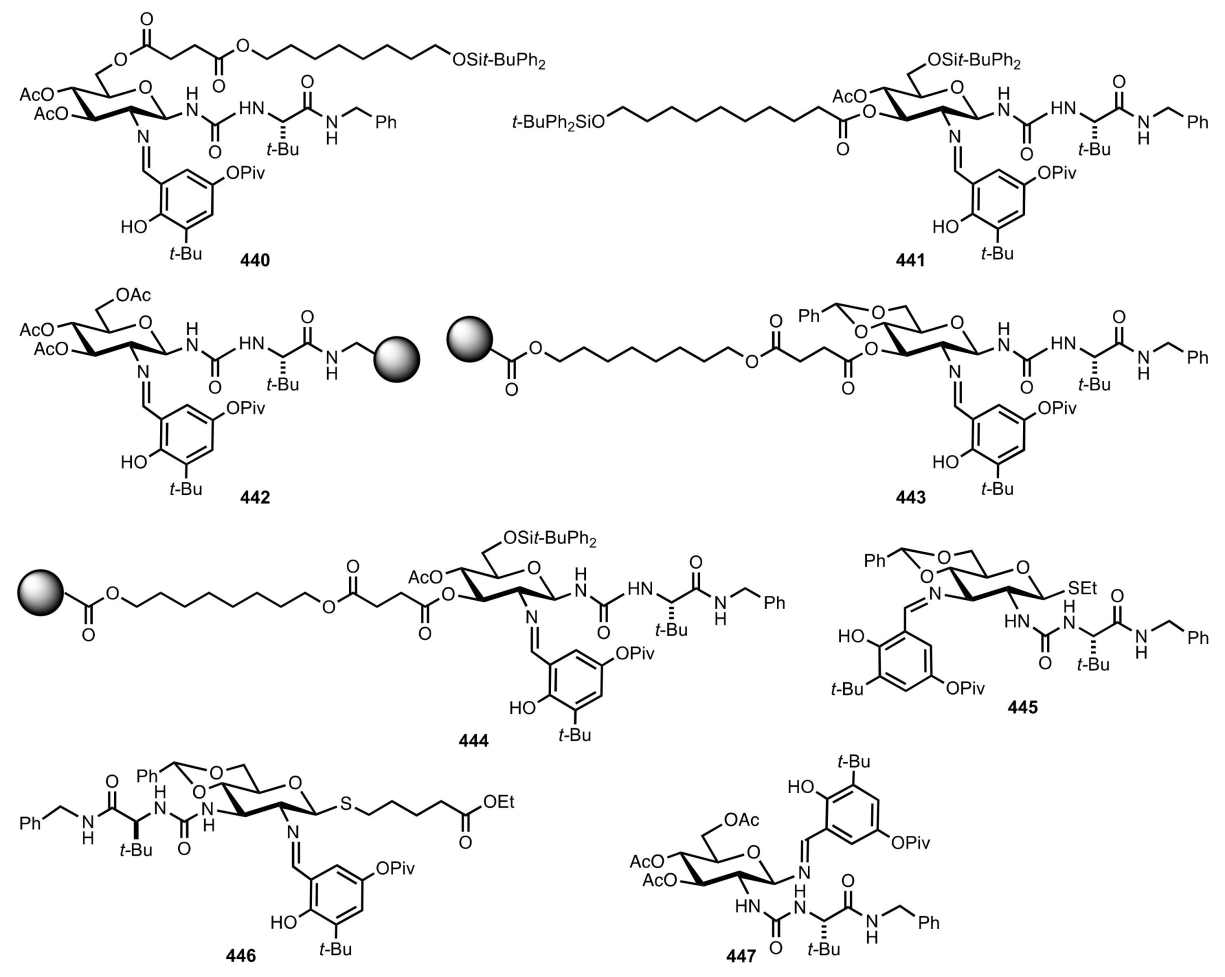
Other sugar imine organocatalysts used by Kunz and co-workers.

Unfortunately, only the synthesis of **439** was described in the paper, the syntheses of all the other catalysts outlined in Figure 30 were reported in three PhD theses but never described in subsequent articles.

**Scheme 118:**
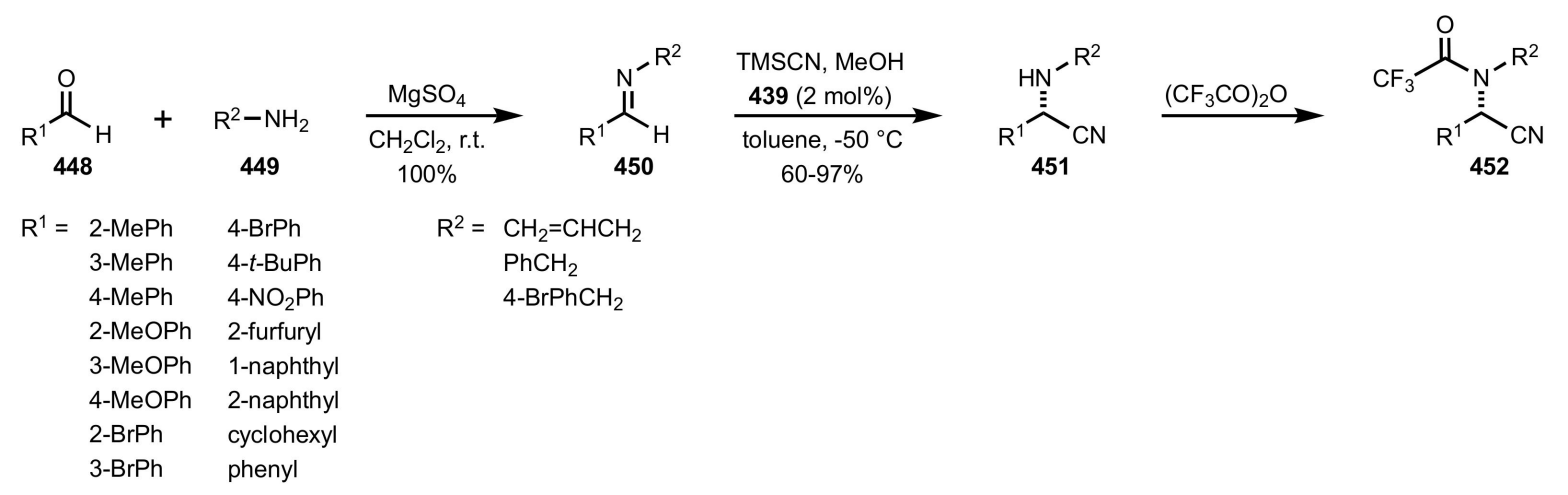
Strecker reaction catalysed by **439**.

The Strecker reaction was performed using the imines **450** prepared from allyl or (bromo)benzyl amine **449** and various aldehydes **448** or, only in one case, a ketone (acetophenone, not shown) (Scheme 118). Coupling of **450** with trimethylsilyl cyanide and methanol (for the in situ generation of HCN) at low temperature in the presence of organocatalyst **439** led to the (*S*)-configured α-amino-nitrile derivatives **451**, which, by reaction with trifluoroacetic anhydride, gave the corresponding amides **452** with enantiomeric excess up to 86%.

The model Strecker reaction between the *N*-allyl-benzylimine and TMSCN was exploited to compare the properties of the organocatalysts **440–447**. It was found that both the soluble and immobilized organocatalysts led to lower yield and much lower enantiomeric excess than those shown by **439**. Therefore, contrary to the original Jacobsen’s catalysts, the numerous polar groups present in these sugar-based catalysts act as basic centers for the TMSCN and disrupt the enantioselectivity of the addition. However, in the case of **439**, the higher acidity of the anomerically linked NH group was likely responsible for its good catalytic activity.

**Scheme 119:**
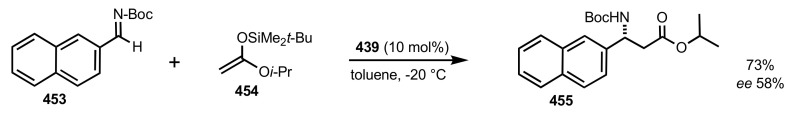
Mannich reaction catalysed by **439**.

The sugar derivative **439** was also employed to catalyse a single Mannich reaction, i.e., the addition of the silyl ketene acetal **454** to the *N*-Boc-imine **453** to give the β-aminoester **455** (Scheme 119). The best results were obtained in toluene at −20 °C; however, the yields were moderate and the enantiomeric excess did not exceed 58%, even by lowering the reaction temperature.

**Scheme 120:**
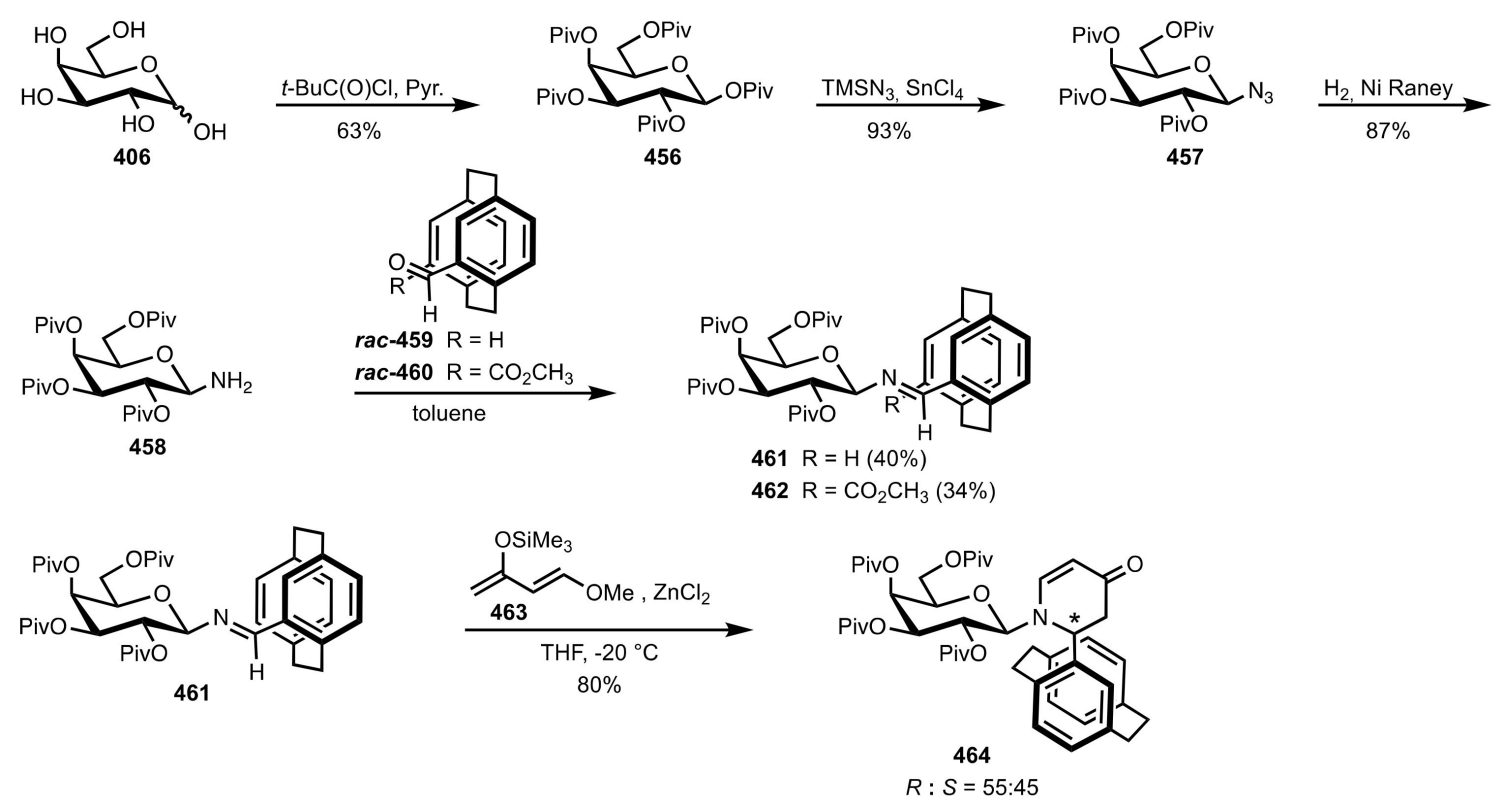
The synthesis of the sugar imine organocatalysts **461**, **462** and **464**.

The same year, Kunz and co-workers prepared [186] other sugar-based organocatalysts able to promote the Strecker reaction. In this case, the carbohydrates contained a planar-chiral [2.2]paracyclophane moiety and were prepared from the tetra-*O*-pivaloylated galactosyl amine **458**, in turn, synthesised in good yield from d-galactose (**406**) by pivaloylation, anomeric azidation and hydrogenation as reported in their previous article [187] (Scheme 120). The reaction with racemic aldehydes **459** and **460** led to the corresponding imines **461** and **462**, respectively. A third organocatalyst was obtained from **461** by reaction with the Danishefsky diene **463** to afford the piperidone derivative **464** as a mixture of diastereoisomers.

**Scheme 121:**
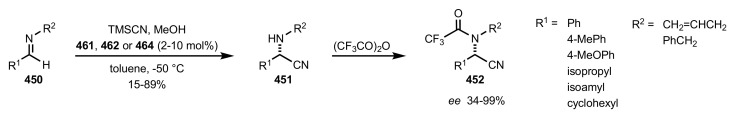
Strecker reactions catalysed by **461**, **462** and **464**.

The Strecker reaction was performed using the imines **450** prepared from allyl or benzyl amine and six aliphatic or aromatic aldehydes (Scheme 121). The coupling of **450** with trimethylsilyl cyanide/methanol was performed in the presence of organocatalyst **461**, **462** or **464** but the best yields and enantiomeric excess were obtained with the methyl ester derivative **462** and imines prepared from aliphatic aldehydes.

**Figure 31:**
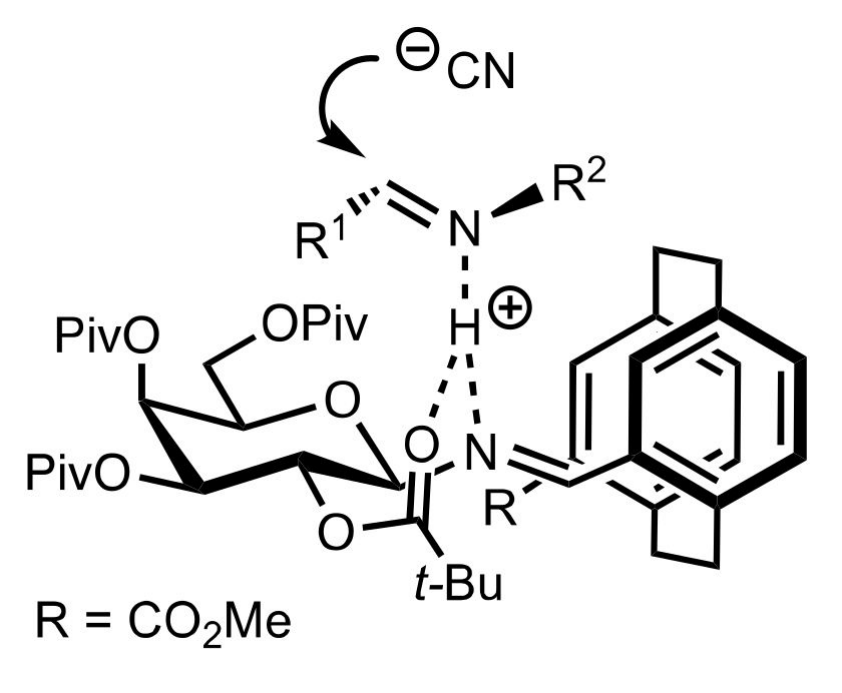
The proposed mechanism for the (*S*)-stereoselective formation of α-amino-nitriles.

The authors pointed out that the activity of these sugar-based organocatalysts is intriguing because they neither contain a Lewis acidic metal ion nor display hydrogen bond donor or Brønsted acid properties. On the other hand, they feature an asymmetric shielding of the double bond and a Lewis basic centre formed by the nitrogen atom of the imine group and the carbonyl oxygen of the pivaloate function at the O-2 position (Figure 31). The basic centre should trap the proton of the in situ formed HCN, and then the imine substrate should approach the reaction site by pointing the R^1^ group toward the back and left and the R^2^ group toward the front and right leading to the selective formation of the (*S*)-enantiomer **451**.

The exocyclic chiral iminium salts already used in asymmetric epoxidation led to moderate enantioselectivity [188]; therefore, Bulman Page and co-workers developed chiral dihydroisoquinolinium tetraphenylborate crystalline salts that afforded very high enantiomeric excess [189]. In another work [190], they synthesised dihydroisoquinolinium salts from carbohydrates in order to take advantage of their polar groups, stereochemical diversity and conformational rigidity. A first series of organocatalysts were prepared from the known [191] tetrabenzylated d-glucosyl- (**466**), d-mannosyl- and d-galactosylamine (not shown).

**Scheme 122:**
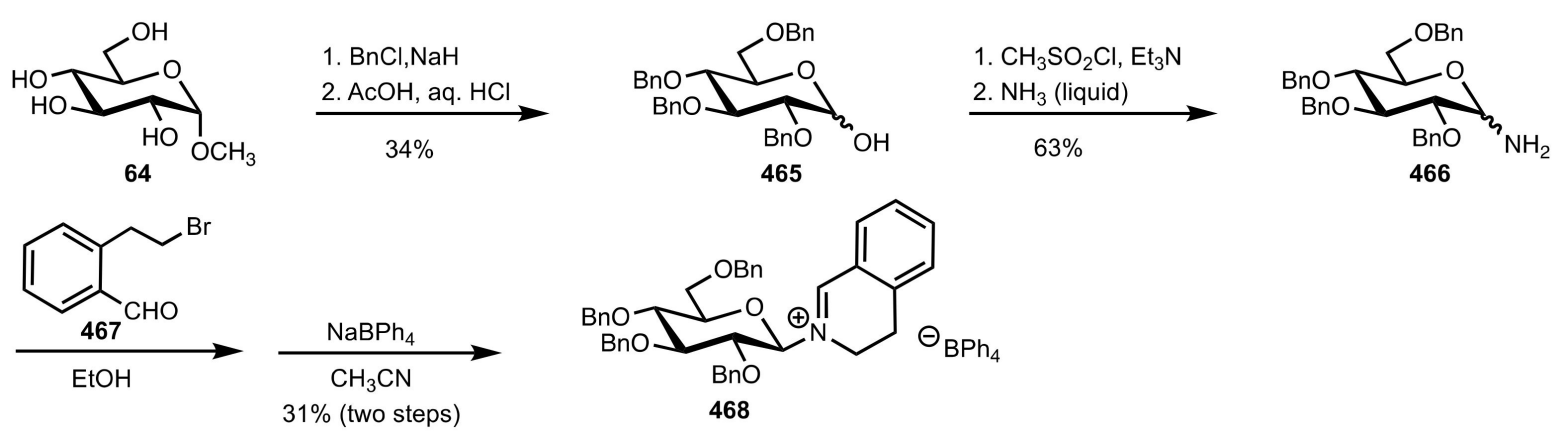
The synthesis of the sugar iminium salt organocatalyst **468**.

However, these known amines were synthesised from the corresponding, commercially available methyl α-d-pyranosides via another reaction sequence outlined in Scheme 122 for the d-*gluco* derivative. The methyl glucoside **64** was perbenzylated and submitted to acid hydrolysis to afford the hemiacetal **465** that was mesylated and treated at low temperature with liquid ammonia to give the glucosylamine **466** as a mixture of anomers. Reaction of the latter with 2-(2-bromoethyl)benzaldehyde (**467**) and subsequent anion metathesis by treatment with sodium tetraphenylborate gave the iminium salt **468** as a pure β-d anomer.

**Figure 32:**
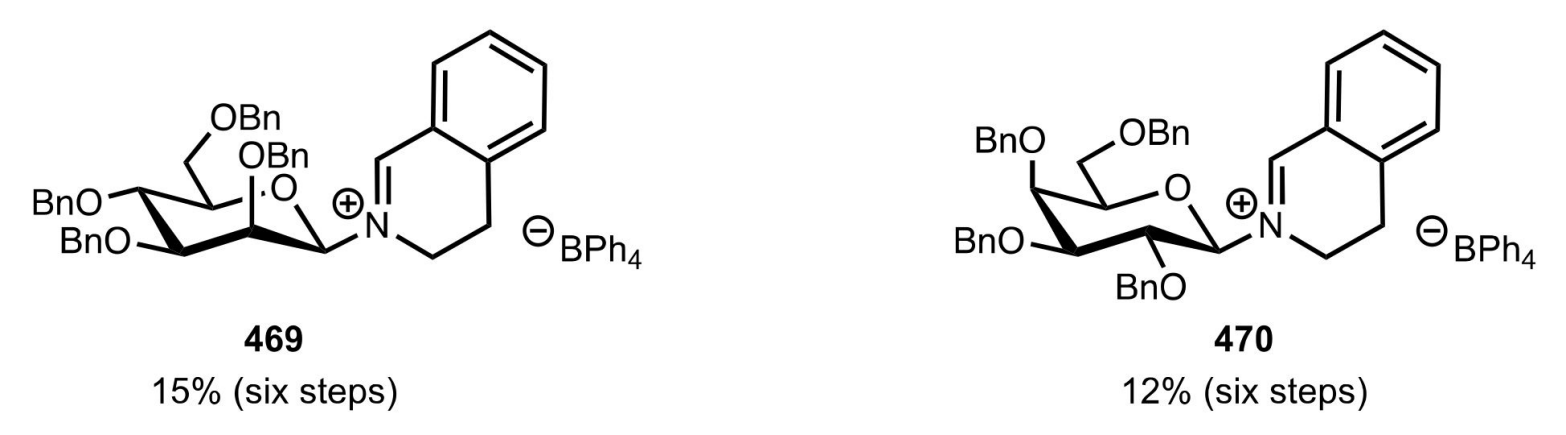
The sugar iminium organocatalysts **469** and **470**.

When the same reaction sequence was applied to the methyl α-d-mannopyranoside and α-d-galactopyranoside, the corresponding β-d anomeric iminium salts **469** and **470** were isolated (Figure 32).

**Scheme 123:**
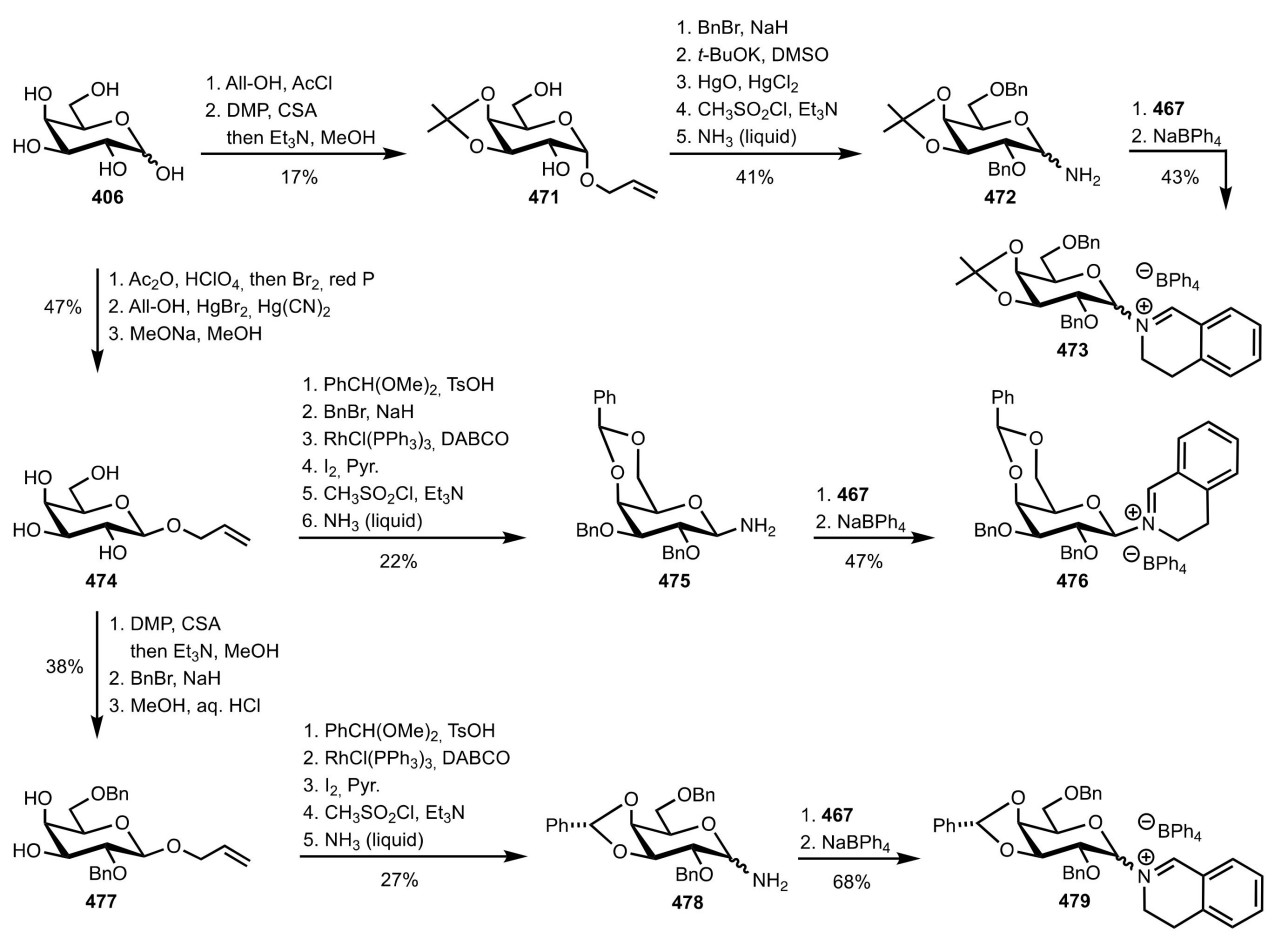
The synthesis of the sugar iminium organocatalysts **473**, **476** and **479**.

A second series of three iminium salts was constituted of d-galactopyranose units bearing an isopropylidene or benzylidene group, all prepared from allyl d-galactopyranosides (Scheme 123). The Fischer glycosidation of d-galactose (**406**) with allyl alcohol (26%) and then the acetonation (67%) in the presence of 2,2-dimethoxypropane (DMP) under the conditions described by Catelani and co-workers [192], afforded the α-d-galactoside **471** that was benzylated and converted into the hemiacetal by removal of the allyl group through basic isomerisation and hydrolysis of the resulting enol ether in the presence of mercury salts.

Mesylation and displacement by ammonia gave the galactosylamine **472**, which, treated with the bromo-aldehyde **467** and NaBPh_4_, led to the iminium salt **473** as an anomeric mixture. The iminium salts **476** and **479** were obtained starting from the allyl β-d-galactopyranoside **474**, in turn, prepared by acetylation of d-galactose, conversion into the anomeric bromide and glycosidation of the latter with allyl alcohol in the presence of mercury bromide and cyanide. The acetate function (participating neighbouring group) at the 2-position insured the formation of the 1,2-trans glycoside, i.e., the β-d anomer.

Deacetylation by transesterification with sodium methoxide in methanol led to **474** that was converted into the 4,6-*O*-benzylidene derivative and benzylated. Removal of the allyl group by treatment with the Wilkinson catalyst and then iodine afforded the hemiacetal from, which the anomeric amine **475** and then the pure β-d-configured salt **476** were obtained by the above-mentioned methods. Treatment of the allyl β-d-galactoside **474** with DMP, benzylation and hydrolysis of the isopropylidene group gave the diol **477** that was converted into a ca. 4:1 mixture of diastereoisomeric 3,4-*O*-benzylidene derivatives. The allyl group of the main *exo*-stereoisomer (X-ray analysis) was removed and the hemiacetal transformed into the amine **478** and the iminium salt **479** (anomeric mixture) through the standard methods.

**Scheme 124:**
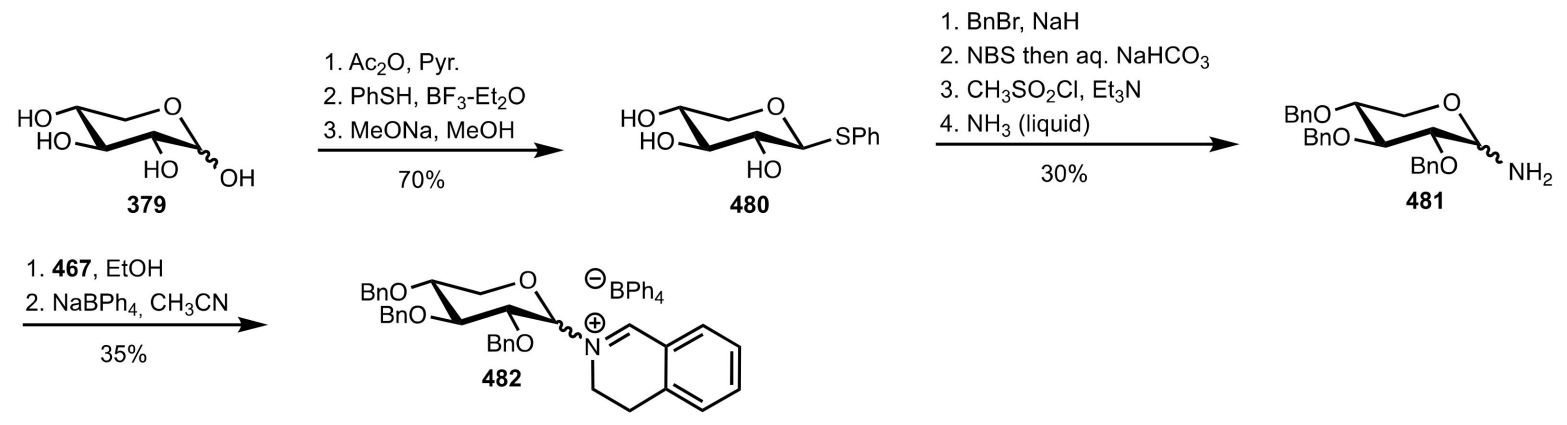
The synthesis of the sugar iminium organocatalyst **482**.

The iminium salt **482** was synthesised starting from d-xylose (**379**) that was easily converted into the phenyl thioglycoside **480** by peracetylation, glycosidation with thiophenol and boron trifluoride and deacetylation by transesterification (Scheme 124). After benzylation and removal of the thiophenyl group by iodination and basic hydrolysis, the resulting hemiacetal was mesylated and treated with liquid ammonia to give the glycosylamine **481** from, which the salt **482** (anomeric mixture) was obtained as described above.

**Scheme 125:**
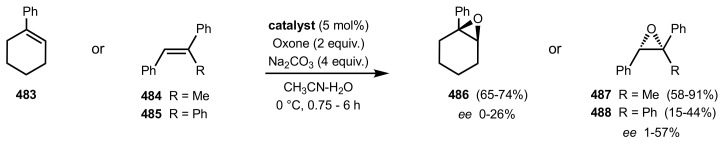
Asymmetric epoxidations catalysed by **468–470**, **473**, **476**, **479** or **482**.

The above-mentioned sugar-based iminium salts were employed as organocatalysts for the asymmetric epoxidation of three model alkenes, 1-phenylcyclohexene (**483**), *trans*-methylstilbene (**484**) and triphenylethylene (**485**), in the presence of Oxone and sodium carbonate to give the corresponding (*S*)-configured epoxides **486–488** (Scheme 125). In all cases, the best isolated yield and enantiomeric excess were found when the tetra-*O*-benzyl-d-galactose derivative **470** was used as the catalyst, whereas the d-*gluco* (**468**) and d-*manno* (**469**) stereoisomers were much less active.

On the other hand, slightly lower yield and *ee* values were observed with the 4,6-*O*-benzylidene-d-galactose derivative **476**. Although not pointed out by the authors, it appears that the properties of the organocatalyst were related to the d-*galacto* stereochemical series and pure β-anomeric configuration (the iminium salts **473**, **479** and **482** were anomeric mixtures).

## 6. Deprotected Monosaccharides

### 6.1. Neutral Sugars

**Figure 33:**
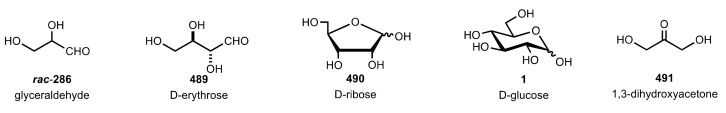
Aldoses and ketose used as organocatalysts in the hydration of α-amino-nitriles.

Aiming to study the hydration of α-amino-nitrile to α-amino-amide under prebiotic conditions, Beauchemin and co-workers performed [193] the reaction in the presence of very common, unprotected sugars (the four aldoses **1**, **286**, **489**, **490** and the ketose **491**) or simple aldehydes (formaldehyde and glycolaldehyde) that were erroneously considered as carbohydrates (Figure 33).

**Scheme 126:**
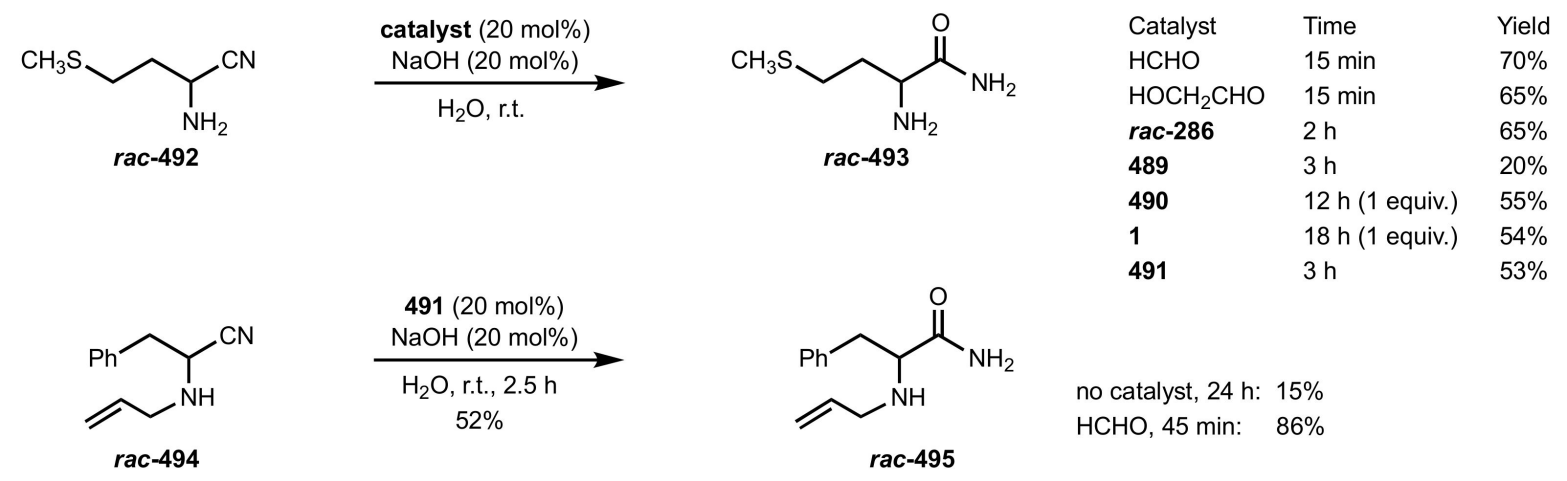
Organocatalysed hydration of α-amino-nitriles.

Since the non-catalysed transformation of nitrile ***rac*-492** into primary amide ***rac*-493** was very sluggish (19% after 12 h), the work demonstrated that low MW aldehydes, in particular the formaldehyde, play an important role in the catalytic hydration of amino-nitriles in an alkaline aqueous medium (Scheme 126). The reactions conducted in the presence of more complex polyhydroxy-aldehydes, such as d-ribose (**490**) and d-glucose (**1**) were less efficient since they required longer reaction time and equimolar amounts of sugars.

It is worth noting that the stereochemical aspects of this reaction were not explored. Moreover, the authors did not take into account that prolonged basic treatment at room temperature of sugar hemiacetals likely led to their epimerization and aldose-ketose isomerization. Therefore, the actual species responsible for the catalytic hydration is difficult to determine. Finally, the potential of the above-mentioned aldoses was not explored in the hydration of the secondary α-amino-nitrile ***rac*-494** where, in addition to simple aldehydes and ketones, only the ketose **491** was employed as catalyst.

### 6.2. Aminosugars

In 2008, Tripathi and co-workers reported [194] the aldol reaction between aromatic aldehydes and cyclohexanone or acetone catalysed by d-glucosamine (**294**), obtained by neutralization of its commercially available hydrochloride salt. The use of d-glucosamine as free base is surprising because an amino sugar in hemiacetal form is not stable upon prolonged periods at room temperature, and the amino group is able to react with the sugar aldehyde leading to an imine. Moreover, the basic epimerization of the aminosugar cannot be excluded. Therefore, the structure of the actual organocatalyst employed in these reactions is not clear.

**Scheme 127:**
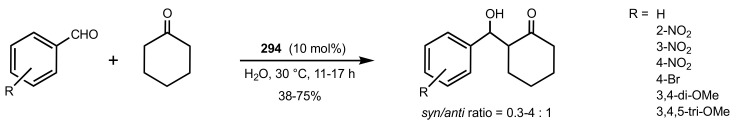
Aldol reactions catalysed by d-glucosamine (**294**).

Variously substituted benzaldehyde derivatives were condensed in water at 30 °C with cyclohexanone (1 equiv.) to give the corresponding aldol in moderate to good yield and a variable *syn/anti* ratio since it ranged from 0.3:1 in the case of 3,4-dimethoxy-benzaldehyde to 4:1 in the case of 4-nitro-benzaldehyde (Scheme 127). The same aldehydes were also reacted with acetone (1 equiv.) under the same conditions to afford the aldol adduct in 35–70% yield and enantiomeric excess from 10% to 54% (the absolute configuration was not established).

**Scheme 128:**
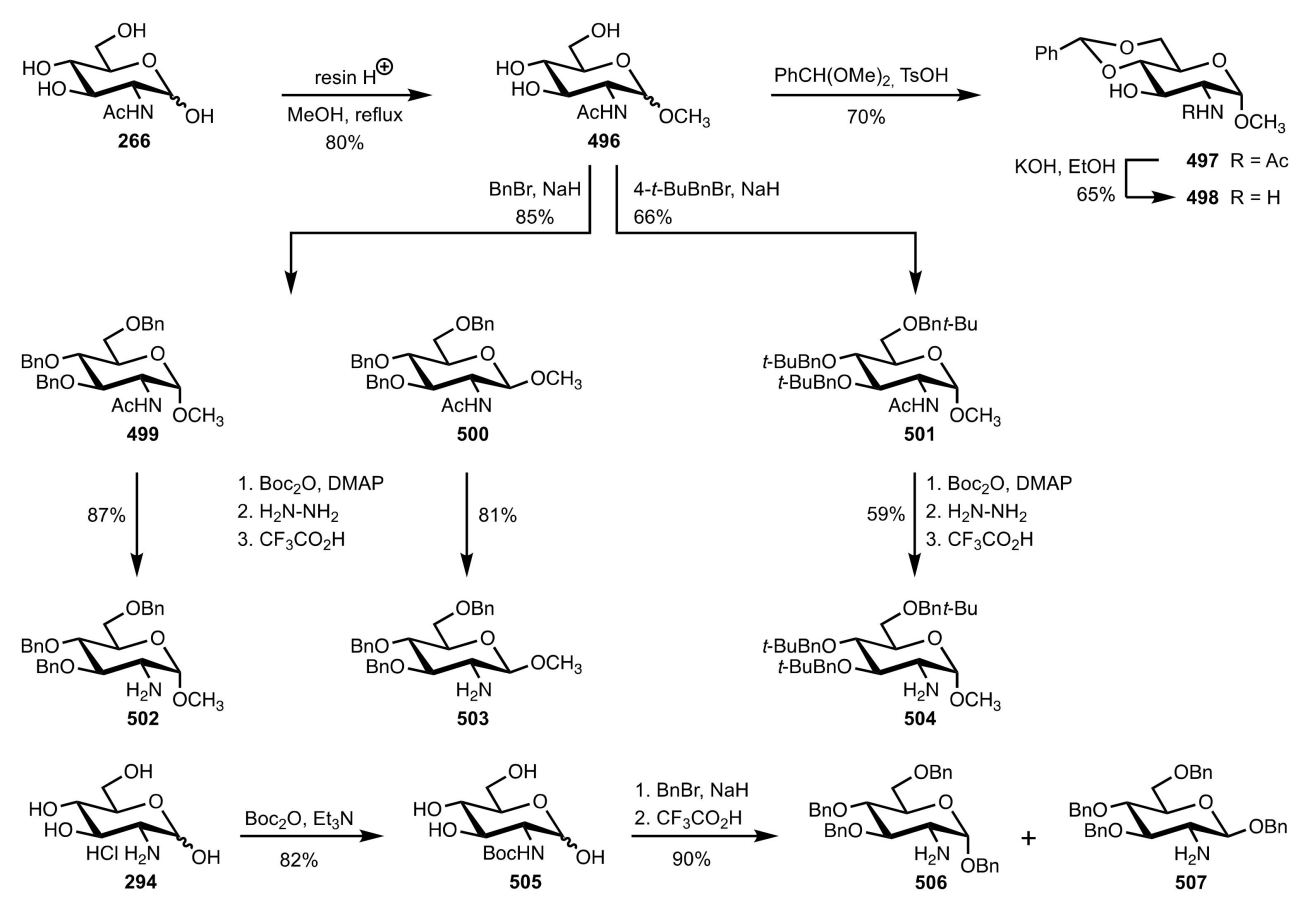
The synthesis of the d-glucosamine glycosides organocatalysts.

Later on, the aldol reactions shown in Scheme 127 were performed using *O*-protected d-glucosamine glycosides, which are chemically and stereochemically stable compounds. Agarwal and Peddinti reported [195] the synthesis of **498** and **502–504** starting from *N*-acetyl-d-glucosamine (**266**) (Scheme 128).

Fischer glycosidation of the latter gave **496** as an anomeric mixture, which was treated with benzaldehyde dimethyl acetal and *p*-toluenesulfonic acid to afford the benzylidene derivative **497** in good yield. Basic hydrolysis allowed the preparation of **498**. On the other hand, **496** was alkylated with benzyl bromide or 4-*t*-butylbenzyl bromide to give the benzylated α- and β-anomers **499** and **500** as well as the α-anomer **501**.

In order to obtain the free amine derivatives **502–504**, the compounds **499–501** were *N*-Boc protected and then treated with hydrazine to remove the *N*-acetyl group and trifluoroacetic acid to remove the *N*-Boc group. The benzyl glycosides **506** and **507**, analogues of the methyl glycosides **502** and **503**, respectively, were synthesised from **294** by protection of the amine function as carbamate (**505**), benzylation of the latter and acid treatment to give a 1:1.5 mixture of the two anomers **506** and **507** that were individually isolated by column chromatography.

**Scheme 129:**
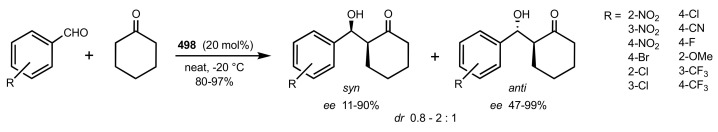
Aldol reactions catalysed by the d-glucosamine glycoside **498**.

After optimization of the reaction conditions, the aldol reaction between the substituted benzaldehyde derivatives and a large excess of cyclohexanone were performed at −20 °C in the absence of solvent using **498** as the organocatalyst, the d-glucosamine glycoside that gave the best results in the preliminary couplings (Scheme 129). The *syn/anti* ratio of the obtained adducts ranged from 0.8:1 to 2:1 and the enantiomeric excess was found to be 11–90% for the *syn* aldol and 47–99% for the *anti* aldol.

**Scheme 130:**
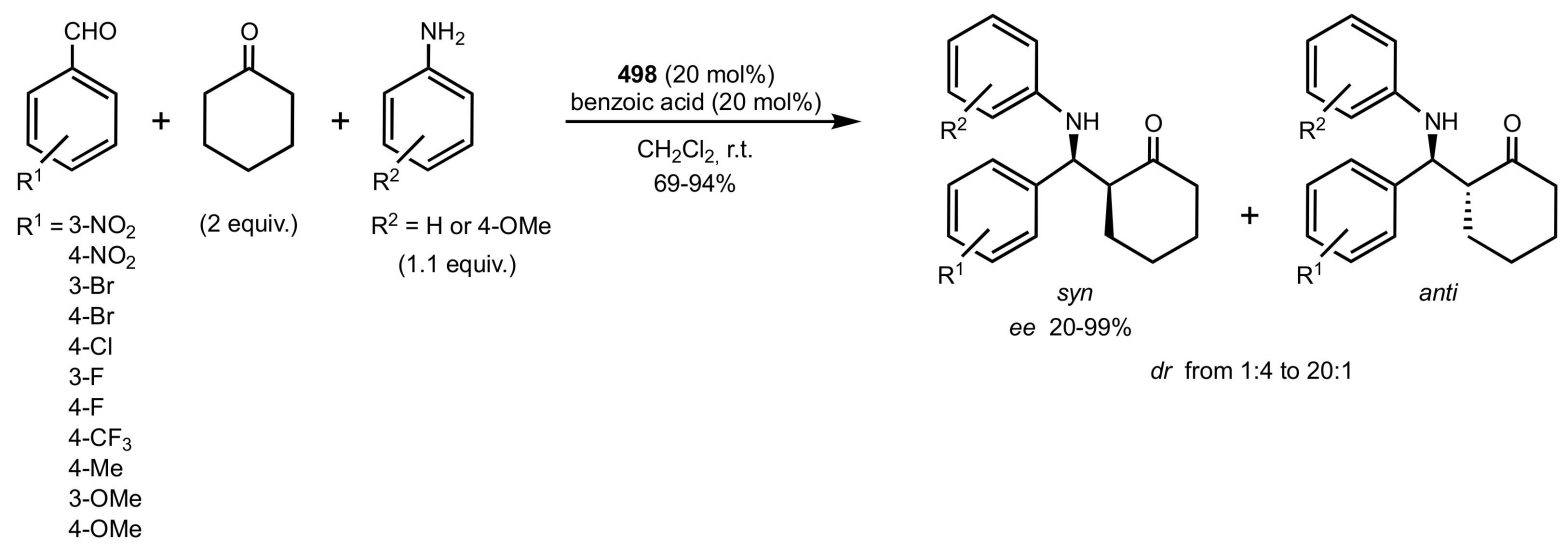
Mannich reaction catalysed by the d-glucosamine glycoside **498**.

A few years later, the same research team exploited [196] the d-glucosamine glycoside organocatalysts **498**, **502**, **506** and **507** to promote the Mannich reaction of substituted benzaldehydes with cyclohexanone and aniline or methoxy-aniline (Scheme 130). In this case, the best results were obtained using **498** as the catalyst.

**Figure 34:**
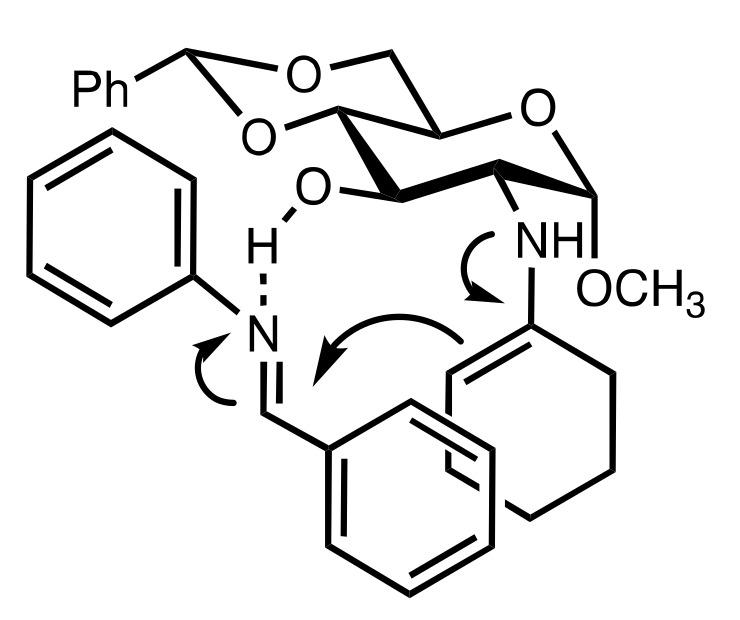
The proposed transition state of the Mannich reaction leading to the *syn* adduct.

The authors proposed that the reaction proceeds through the in situ formation of an enamine from cyclohexanone and the d-glucosamine derivative **498**. Then, the enamine reacts with the imine formed from the aromatic aldehyde and aniline (or anisidine) via the transition state shown in Figure 34. The hydroxyl group of the sugar catalyst activates the imine by hydrogen bonding and also allows the attack on the *Si* face of the imine leading to the *syn* adduct, i.e., the major compound isolated in most of the cases outlined in Scheme 130.

**Scheme 131:**
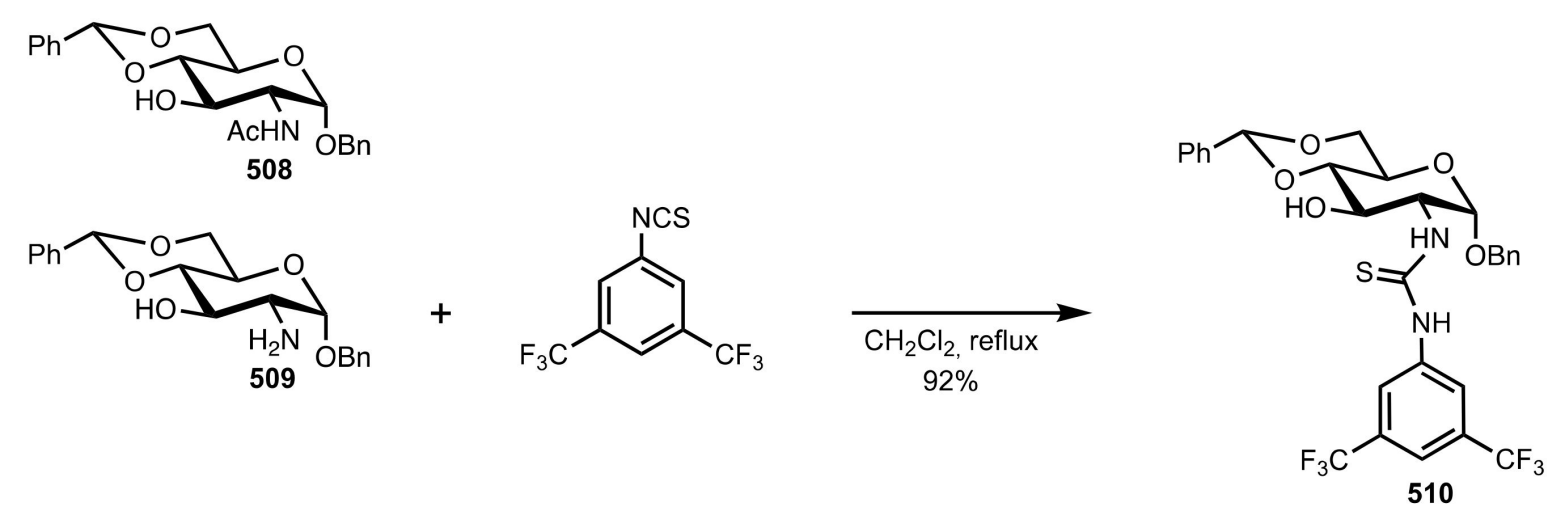
d-glucosamine glycosides organocatalysts **508–510** used for the aldol reaction.

**Scheme 132:**
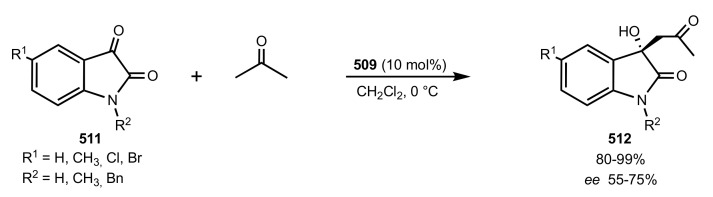
Aldol reaction catalysed by the d-glucosamine glycoside **509**.

The d-glucosamine hydrochloride (**294**), the benzylidene derivatives **497** and **498** (see Scheme 128), as well as the benzyl glycosides **508–510** (Scheme 131) were used by Chen and co-workers [197] to catalyse the aldol reaction between several isatin derivatives **511** and acetone (Scheme 132). The organocatalysts **508** and **509** were prepared [198] as described for the methyl glycoside analogues **497** and **498**; however, in the quoted article the yields were not indicated. On the other hand, the glucosamine thiourea derivative **510** was easily obtained by coupling **509** with 3,5-bis(trifluoromethyl)phenyl isocyanate.

Preliminary experiments allowed establishing that only the sugar organocatalysts bearing a free amine function are able to promote the aldol reaction. In a model reaction, the best results were obtained when the benzyl glycoside **509** was employed as catalyst. Therefore, **509** was chosen to catalyse the series of aldol reactions shown in Scheme 132. In all cases, the reaction afforded the (*S*)-configured enantiomer **512** as the major adduct in high yield. When acetophenone or cyclohexanone instead of acetone were used as substrate, a very poor enantiomeric excess was observed.

**Figure 35:**
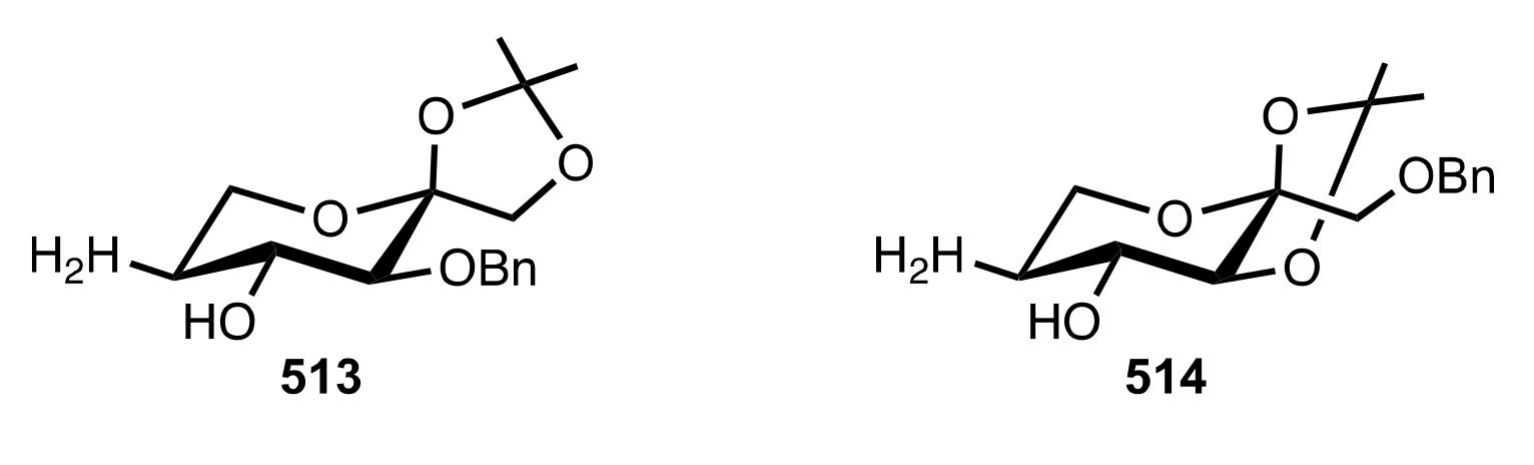
d-Fructose-derived organocatalysts **513** and **514**.

Other examples of organocatalysed aldol reactions were reported in 2013 by Fang and co-workers [199]. They exploited for the first time amino alcohols derived from d-fructose, such as **513** and **514** (Figure 35). Unfortunately, the preparation of these compounds was reported only in a Chinese patent and a Chinese journal [200].

**Scheme 133:**
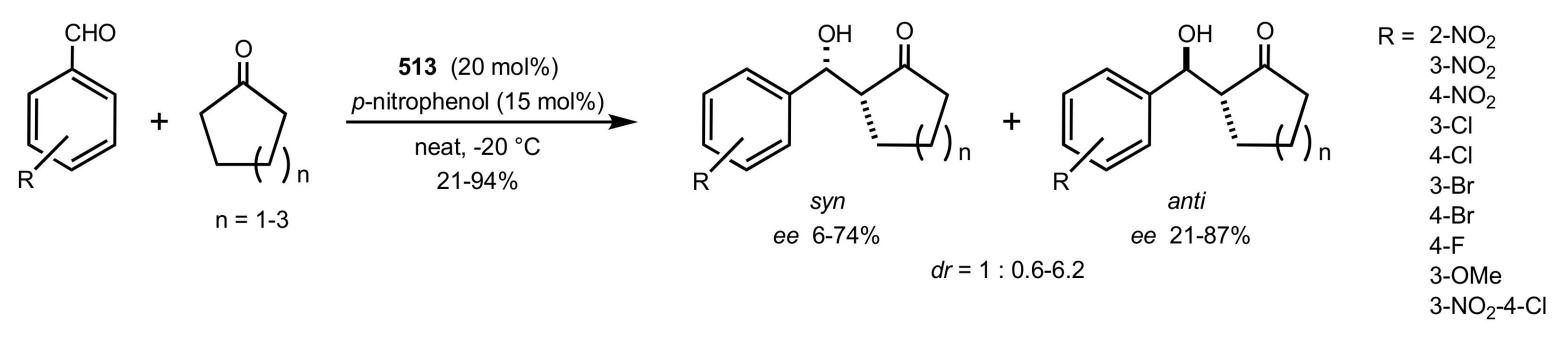
Aldol reaction catalysed by the organocatalyst **513**.

In a model reaction, the amino alcohol **513** was found to be the most stereoselective catalyst; therefore, it was then used to react several substituted benzaldehydes with three cycloalkanones (large excess) in the presence of *p*-nitrophenol (Scheme 133).

**Figure 36:**
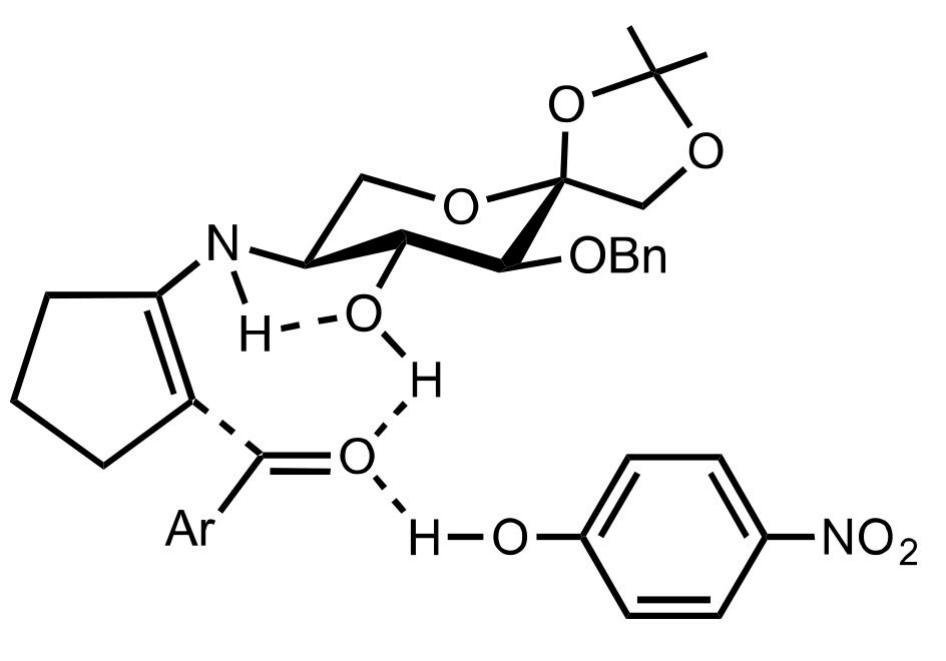
The proposed transition state of the aldol reaction catalysed by **513** and *p*-nitrophenol.

The proposed transition state of the reaction, which involves a double hydrogen bonding of the *p*-nitrophenol, is shown in Figure 36.

**Scheme 134:**
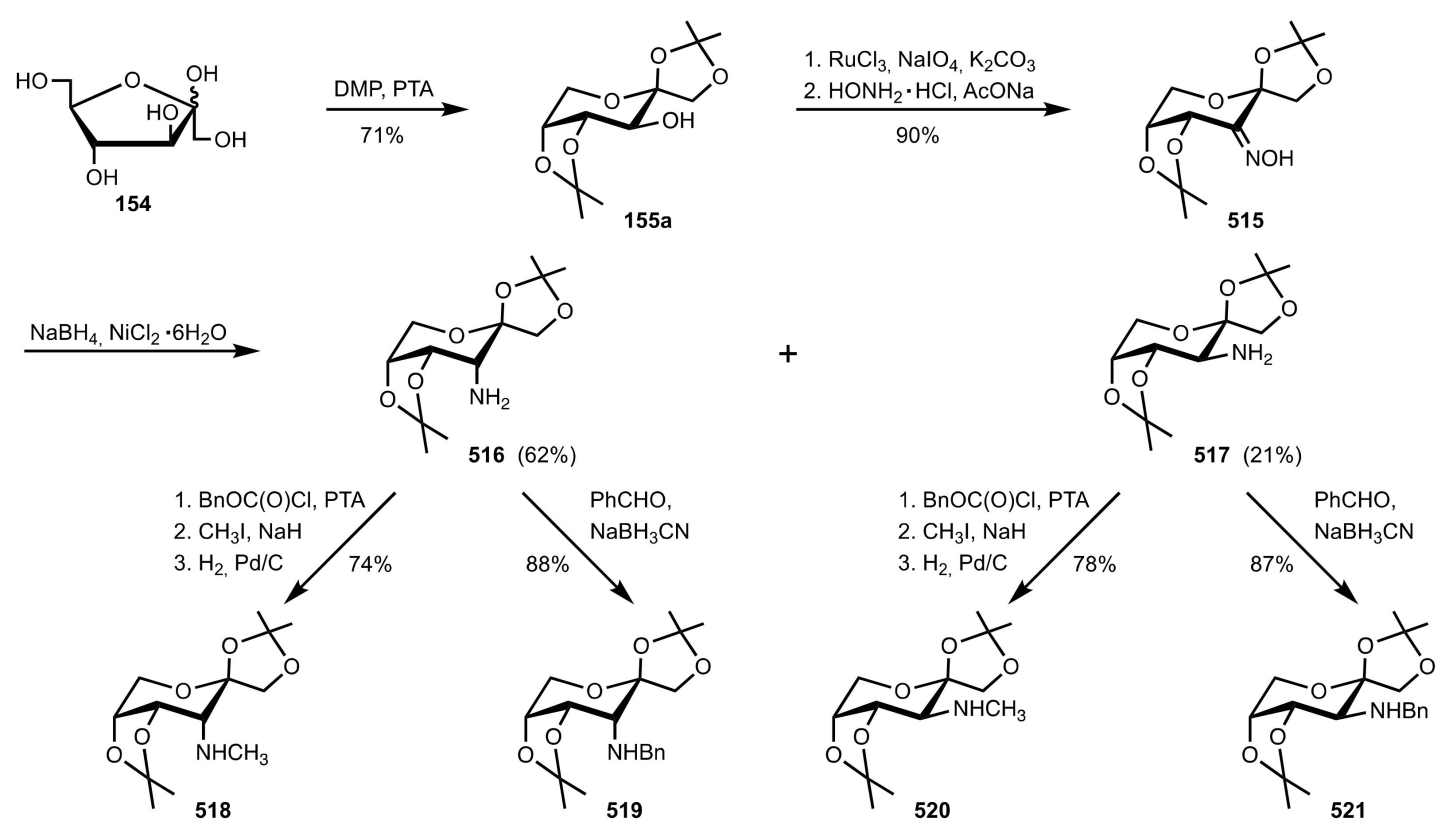
The synthesis of the d-fructose derived organocatalysts **516–521**.

Another research team envisaged the use of fructose-derived amines as organocatalysts for aldol reactions [201]. The organocatalysts **518–521** (Scheme 134) were prepared from the epimeric amines **516** and **517** that the authors erroneously called enantiomers and considered as α-d- and β-d-fructopyranose anomers instead of d-*ribo*-hex-2-ulose and d-*arabino*-hex-2-ulose (d-fructose) derivatives, respectively (see compounds **1a**, **2a** and following products of the series in their article [201]). The primary amines **516** and **517** were obtained by reduction of the known [202] oxime **515**, in turn prepared from d-fructose (**154**) by treatment with 2,2-dimethoxypropane (DMP) and phosphotungstic acid (PTA) to give the alcohol **155a**, oxidation to ketone and conversion of the latter into oxime (Scheme 134).

The amines **516** and **517** were transformed into the corresponding *N*-methyl derivatives **518** and **520** by protection as benzyloxycarbamate, methylation and catalytic hydrogenation. The *N*-benzyl derivatives **519** and **521** were easily obtained from the corresponding primary amine **516** and **517** by reductive amination in the presence of benzaldehyde and NaBH_3_CN.

**Scheme 135:**
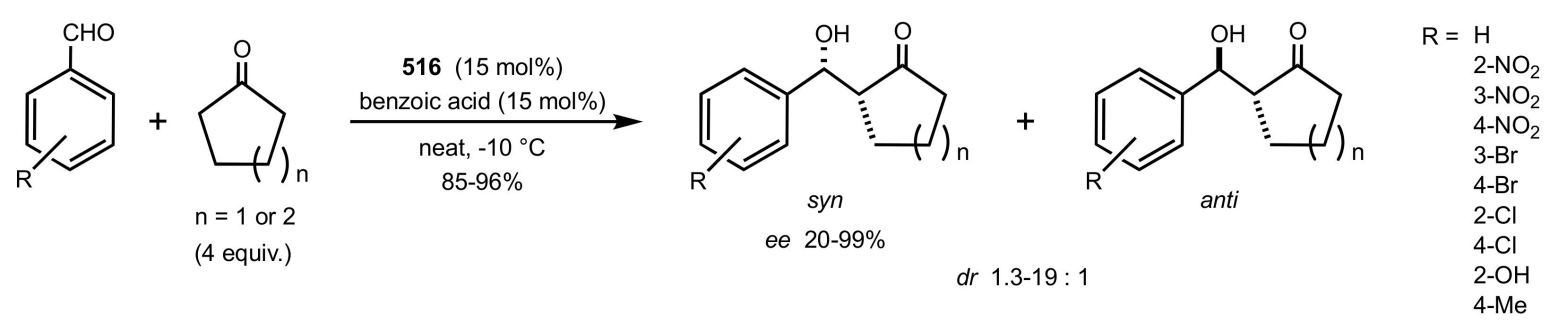
Aldol reaction catalysed by the organocatalyst **516**.

A model aldol reaction allowed establishing that the secondary amine organocatalysts **518–521** gave very poor chemical and stereochemical results, whereas both the primary amines **516** and **517** afforded the adduct in high isolated yield. Since better stereoselectivity was observed with **516**, the reaction of a series of substituted benzaldehydes with cyclopentanone or cyclohexanone was conducted only in the presence of this organocatalyst (Scheme 135). The major adduct was *syn*-configured in all cases with the exception of the coupling of 2-nitrobenzaldehyde with cyclohexanone where a *syn/anti* ratio of 1:2.2 was observed (*ee* of the *anti* adduct = 95%).

**Figure 37:**
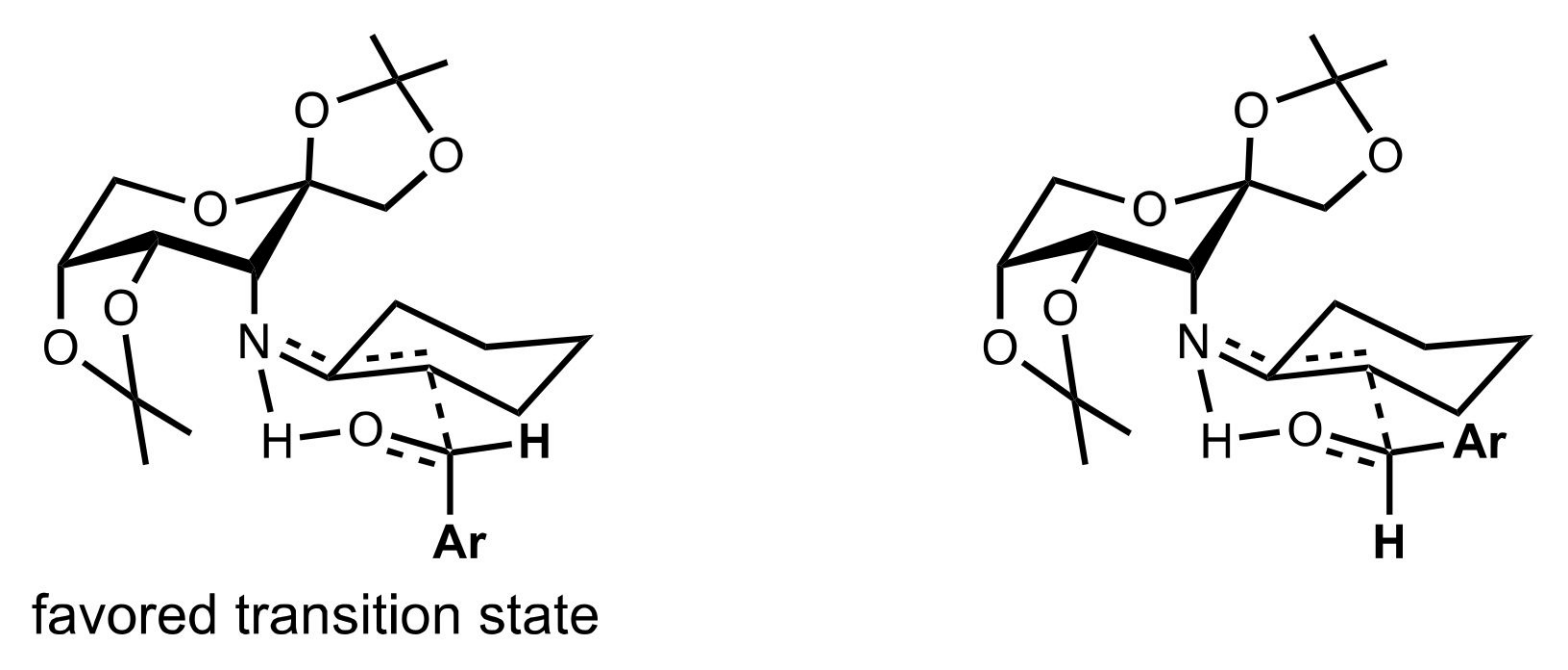
The proposed transition state of the aldol reaction catalysed by **516**.

The proposed six-membered transition state of the reaction, where the aldehyde is activated by hydrogen bonding with the NH group of the enamine, is shown in Figure 37.

**Scheme 136:**
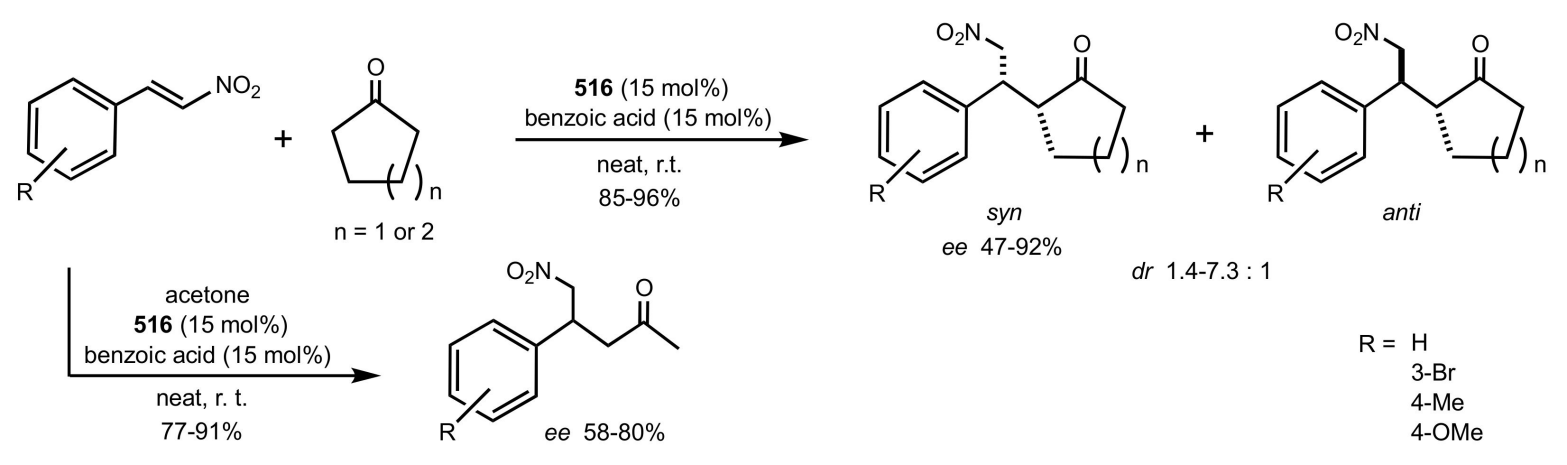
The Michael additions catalysed by the organocatalyst **516**.

In a second article published the same year [203], Bez and co-workers exploited the same fructose-derived organocatalysts **516–521** (see Scheme 134) to perform Michael additions of ketones to aromatic nitroalkenes. In this case, the secondary amines **518–521** were not active while the best results were observed when **516** was used as the catalyst (Scheme 136). In the reactions with cyclic ketones, the *syn*-adduct was formed as the major stereoisomer in moderate to good enantiomeric excess (the absolute configuration of the adducts was not established).

**Figure 38:**
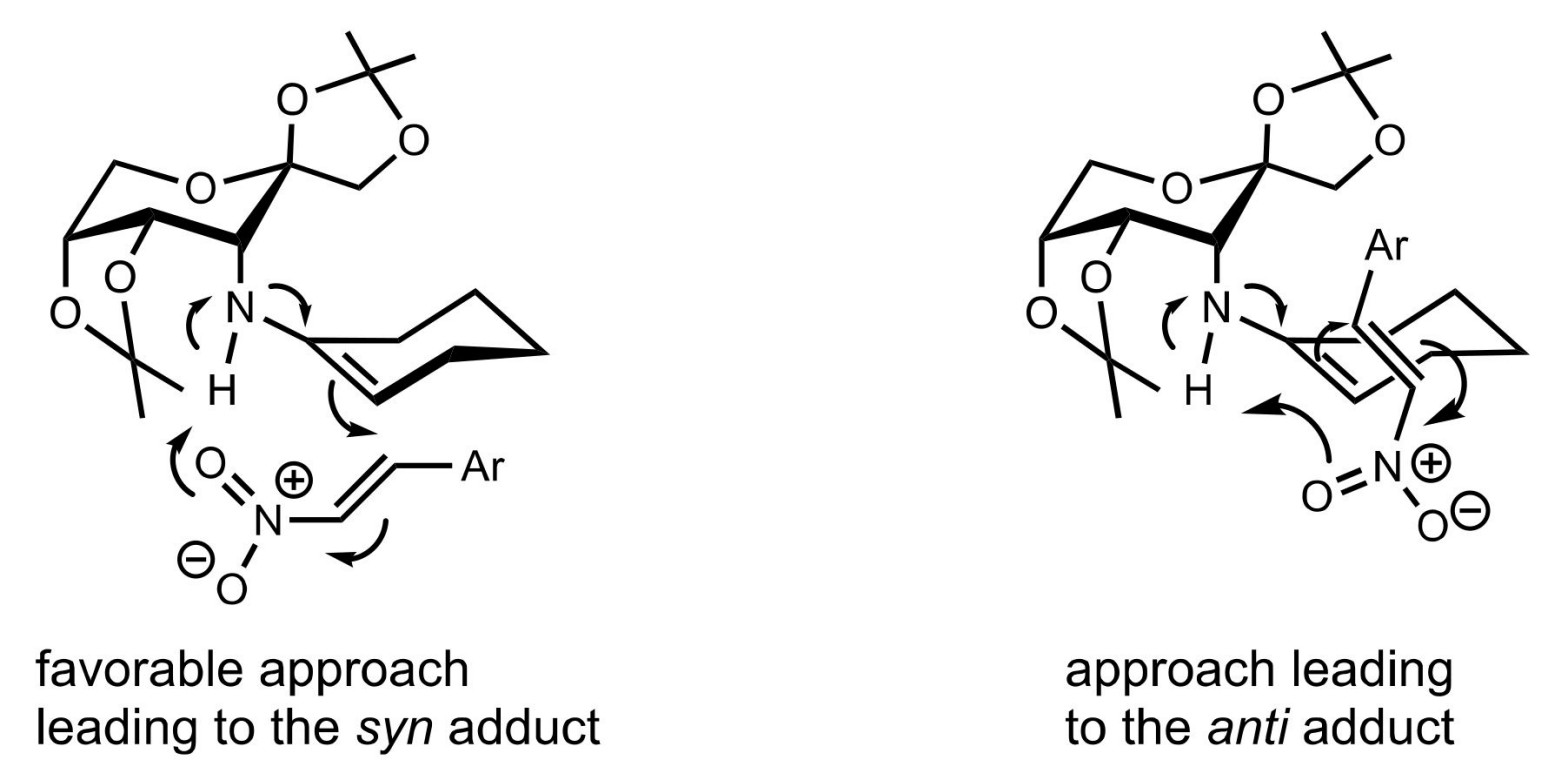
The proposed mechanism for the Michael additions catalysed by **516**.

To explain the *syn*-stereoselectivity, a mechanism was proposed by the authors (Figure 38).

## 7. Polysaccharides

The use of natural or modified polysaccharides, in particular chitosan, as organocatalysts over the last two decades has been described in four recent reviews [22,23,24,25]. It is worth noting that although many articles reported reactions organocatalysed by polysaccharides, most of them did not address the stereochemical issues because the obtained products were not chiral or, when chiral molecules were synthesised, the stereochemical outcome was not studied at all.

Thus, chitosan-based organocatalysts were employed in Knoevenagel [204,205,206,207,208,209] and aldol reaction [204,205,210,211], three-component reactions [212,213,214], aldehyde self-condensation [215,216], epoxide opening reactions [217,218], Strecker reaction [219], transamidation reaction [220], Petasis borono–Mannich reaction [221], Hantzsch-type condensation [222], in synthesis of dyes [223] and perimidines [224]. In addition to chitosan, also chitin [225] and modified cellulose [226] were employed to catalyse the Knoevenagel condensation. Another cellulose-based organocatalyst was used for the synthesis of α-aminophosphonates [227]. On the other hand, all the articles reporting on stereoselective organocatalysed reactions will be reviewed in the present section.

### 7.1. Chitosan

The chitosan is obtained by partial basic hydrolysis of the chitin (the homopolymer of *N*-acetyl-d-glucosamine linked through β-1,4 glycosidic bonds) isolated from various natural sources. Considering the variety of starting material and chemical treatment, it is important to indicate the percentage of the free amino groups, the molecular weight, and the polydispersity index for each batch of chitosan.

**Figure 39:**
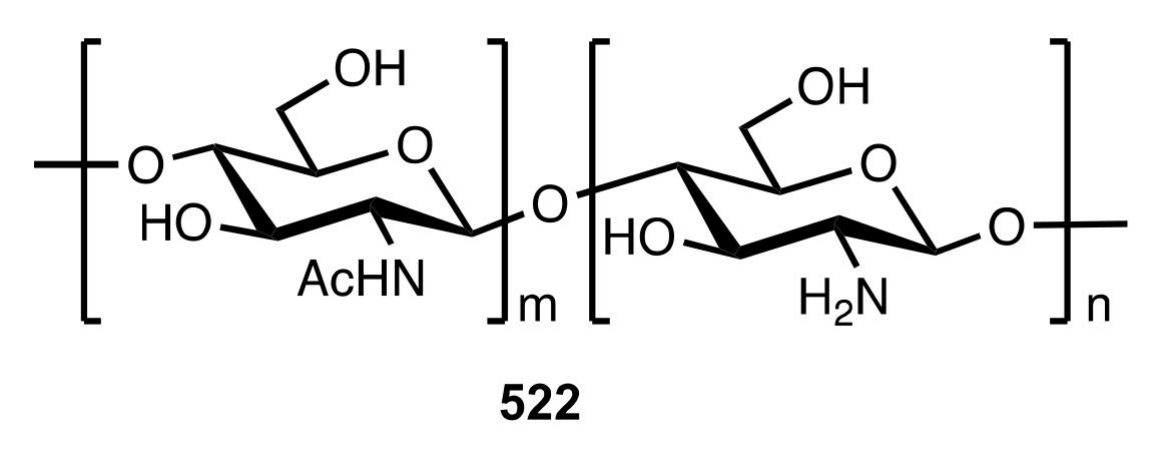
The structure of the chitosan.

In 2010, Ricci, Quignard and their co-workers described [228] the aldol reaction of model aldehydes and ketones employing the chitosan **522** (Figure 39) aerogel microspheres previously developed by some of the authors. They found that the aerogel formulation of chitosan was better than the commercially available chitosan or the chitosan hydrogel because it affords a material with defined molecular weight distribution, high surface area (up to 350 m^2^ per gram) and high accessibility to the free amine groups (up to 5.2 mmol per gram).

These features allow a better reproducibility of the reactions in respect to those conducted with commercial chitosan, whose molecular weight (MW) and composition depend on the natural source. Moreover, the solid, spongy morphology of the chitosan aerogel microsphere facilitates its handling and recycling.

Upon optimization of the reaction conditions using 4-nitrobenzaldehyde and cyclohexanone, it was found that chitosan **522** aerogel (MW = 700,000, acetylation degree = 8%) was more efficient than its monomeric derivative, i.e., d-glucosamine, and that the addition of weak acids (stearic acid or 2,4-dinitrophenol), which catalyse the formation of the enamine intermediate, increased the yield and enantioselectivity.

**Scheme 137:**
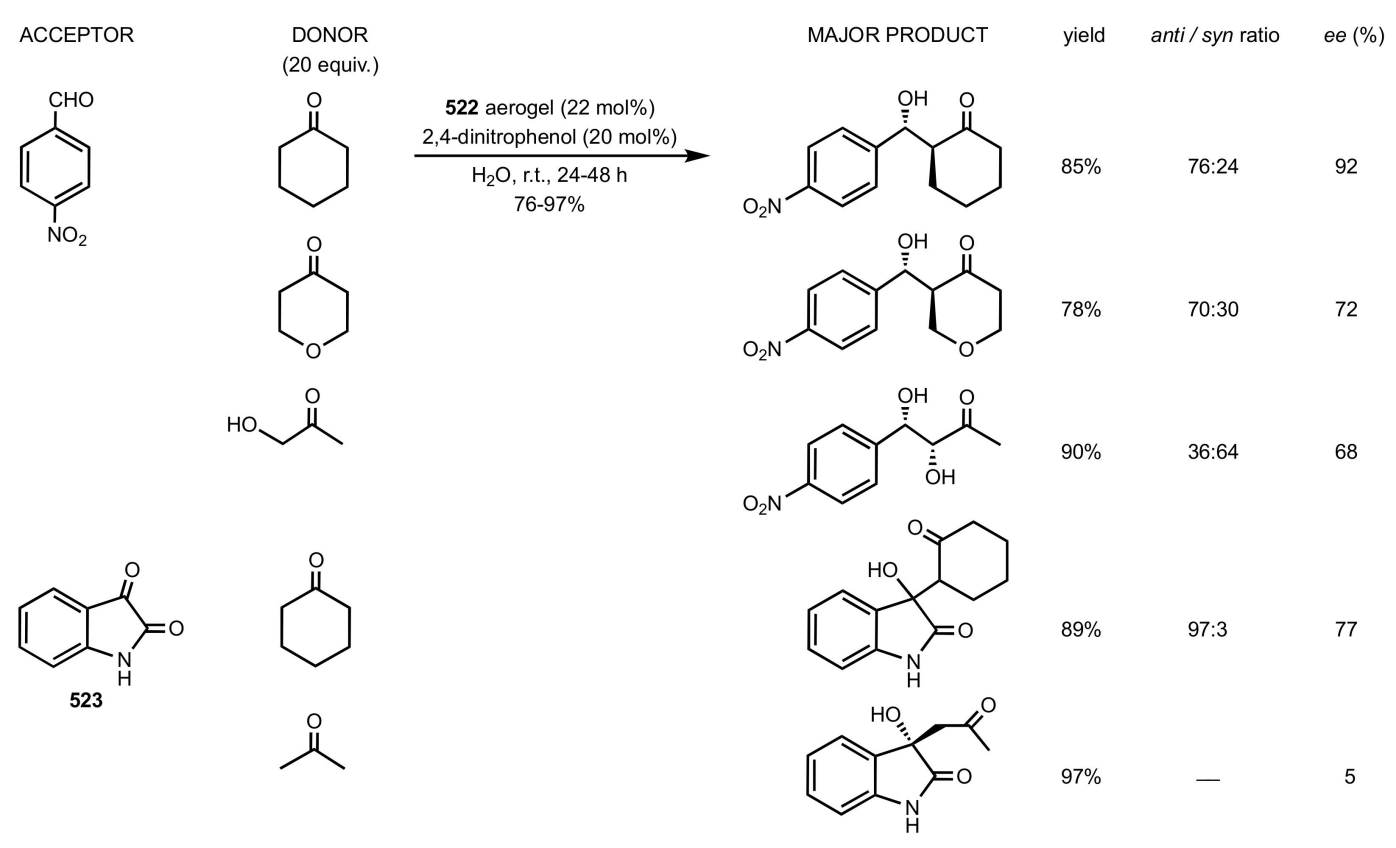
Aldol reactions catalysed by chitosan **522** aerogel.

Then, the 4-nitrobenzaldehyde and isatin **523** (Scheme 137) were reacted in water at r.t. with a large excess of various ketones in the presence of 22 mol% of chitosan **522** aerogel (based on the free amine content). In all cases, the adducts were formed in good isolated yield and enantioselectivity with the exception of the aldols obtained from isatin and acetone where the enantiomeric excess was extremely low. Finally, it was demonstrated that the chitosan microsphere organocatalyst could be easily recovered and recycled at least three times without a loss of chemical and stereochemical efficiency.

**Figure 40:**
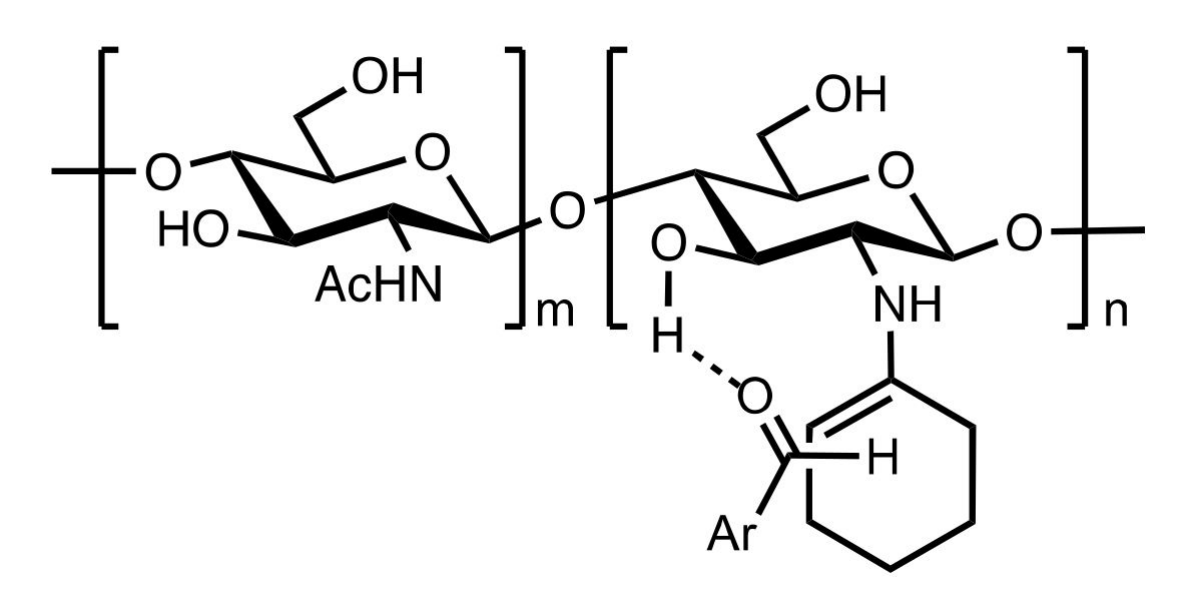
The proposed transition state for the addition of cyclohexanone to 4-nitrobenzaldehyde to give the *anti* aldol.

The authors suggested a reaction mechanism based on the attack of an enamine intermediate to the *Re*-face of the aldehyde that displays a hydrogen bond with the 3-OH group of the d-glucosamine unit (Figure 40).

**Scheme 138:**
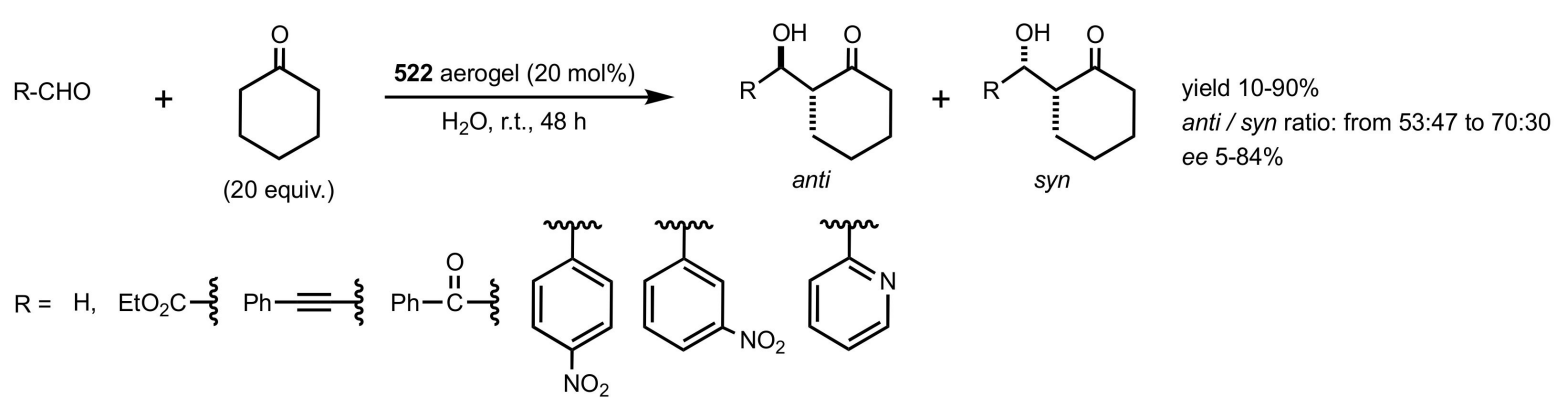
Aldol reactions catalysed by chitosan **522** aerogel.

Three years after their preliminary communication [228], Ricci, Quignard and their co-workers reported [229] further studies on the aldol reaction using the chitosan **522** aerogel microspheres (4 mmol/g accessible amino groups) as the organocatalyst. A series of aliphatic and aromatic aldehydes were condensed in water at r.t. with 20 equivalents of cyclohexanone in the presence of 20 mol% (based on the free amine content) of **522** aerogel but without any additive (Scheme 138). The aldol adducts, as mainly *anti* stereoisomers, were isolated in good yield except in the case of formaldehyde where only 10% of aldol was obtained. Despite the generally good yield, the adducts showed variable enantiomeric excess ranging from 5% to 84%.

**Scheme 139:**
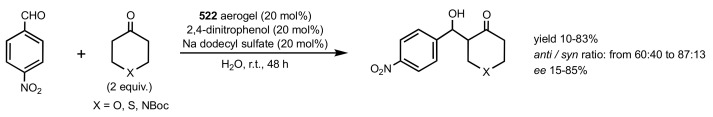
Optimized conditions for the aldol reactions catalysed by chitosan **522** aerogel.

Then, the authors envisaged the use of additives in order to reduce the very large excess of ketone donor (20 equiv.) used in the aldol reaction and also because some heterocyclic ketones are quite expensive. After experimentation, they found that in the presence of both a weak acid (2,4-dinitrophenol) and an anionic surfactant (sodium dodecyl sulphate, SDS) the reaction led to the aldols in satisfactory yield as well as enantio- and diastereoselectivity even when only two equivalents of ketone were employed (Scheme 139). Slightly worse results were obtained by using SDS with linoleic acid instead of 2,4-dinitrophenol as the additive.

**Scheme 140:**
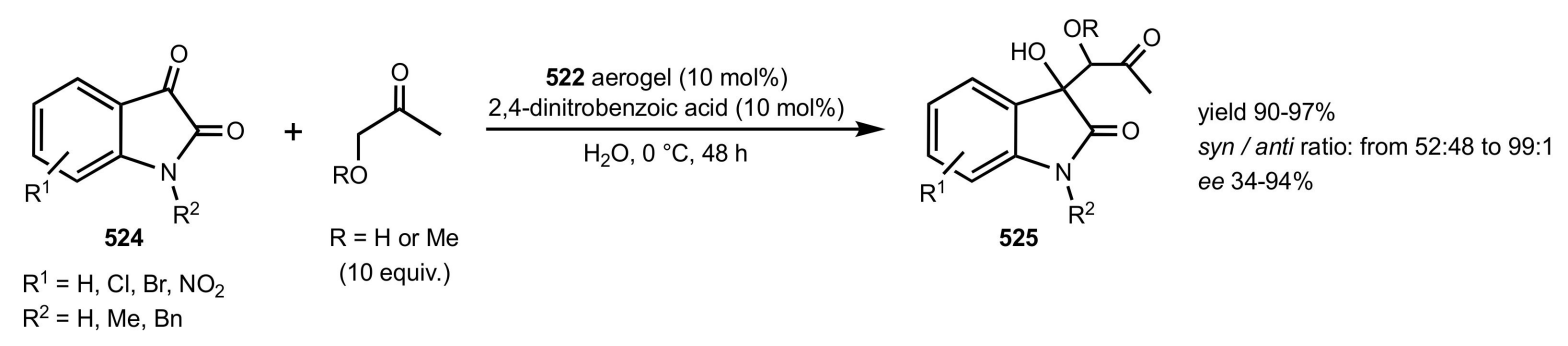
Aldol reactions catalysed by chitosan **522** aerogel.

In 2016, Ouyang and co-workers [230] exploited the same chitosan **522** aerogel [228,229] to perform the aldol reaction between hydroxyacetone and substituted isatin derivatives (Scheme 140). After some experimentation, not surprisingly water was found to be the best solvent for the aldol reaction catalysed by chitosan.

Moreover, the authors performed this reaction in the presence of various additives, such as carboxylic or sulphonic acids, phenols, l-proline, molecular sieves, and observed the best results when 2,5-dinitrobenzoic acid (10 mol%) was used as the additive at 0 °C. Having optimized the conditions, a series of *C*- and *N*-substituted isatin derivatives **524** were condensed with 10 equivalents of hydroxy- or methoxy-acetone to give, after 48 h at 0 °C, the corresponding 3-hydroxy-2-oxindole **525** in very high yield and, in some cases, excellent diastereo- and enantioselectivity.

**Scheme 141:**
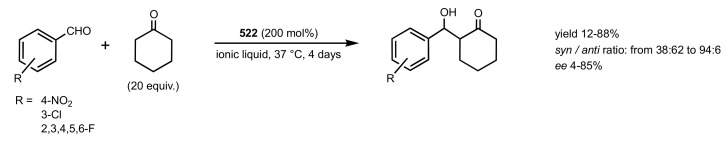
Aldol reactions catalysed by chitosan **522** in ionic liquids.

In addition to chitosan **522** aerogel, also a 10% (*w*/*w*) solution of commercially available chitosan (MW = 100,000–300,000) in various ionic liquids was used as the organocatalyst [231] for aldol reactions. Three aromatic aldehydes bearing electron-withdrawing substituents were condensed at 37 °C with 20 equivalents of cyclohexanone in the presence of two equivalents (based on the free amine content) of **522** using ten commercially available ionic liquids as the solvent (1-butyl-3-methylimidazolium or 1-ethyl-3-methylimidazolium acetate, bromide, chloride, hexafluorophosphate and tetrafluoroborate) (Scheme 141).

Despite the large amounts of chitosan **522**, the isolated yield and the diastereoselectivity of the aldol adducts were rather low, although, in some cases, the addition of 20 mol% of an acid (acetic or trifluoroacetic acid) allowed improvement in the results. High yield (88%) and *syn*/*anti* ratio (94:6) were observed only when 3-chlorobenzaldehyde was employed; however, the enantiomeric excess for both the *syn* (30%) and *anti* (6%) adducts were very low. Finally, when the reaction between the 4-nitrobenzaldehyde and cyclohexanone was performed in water, the aldols were recovered in only 13% yield (the reaction did not take place at all in pure DMSO).

### 7.2. Chitosan-Cinchona Alkaloids Conjugates

**Scheme 142:**
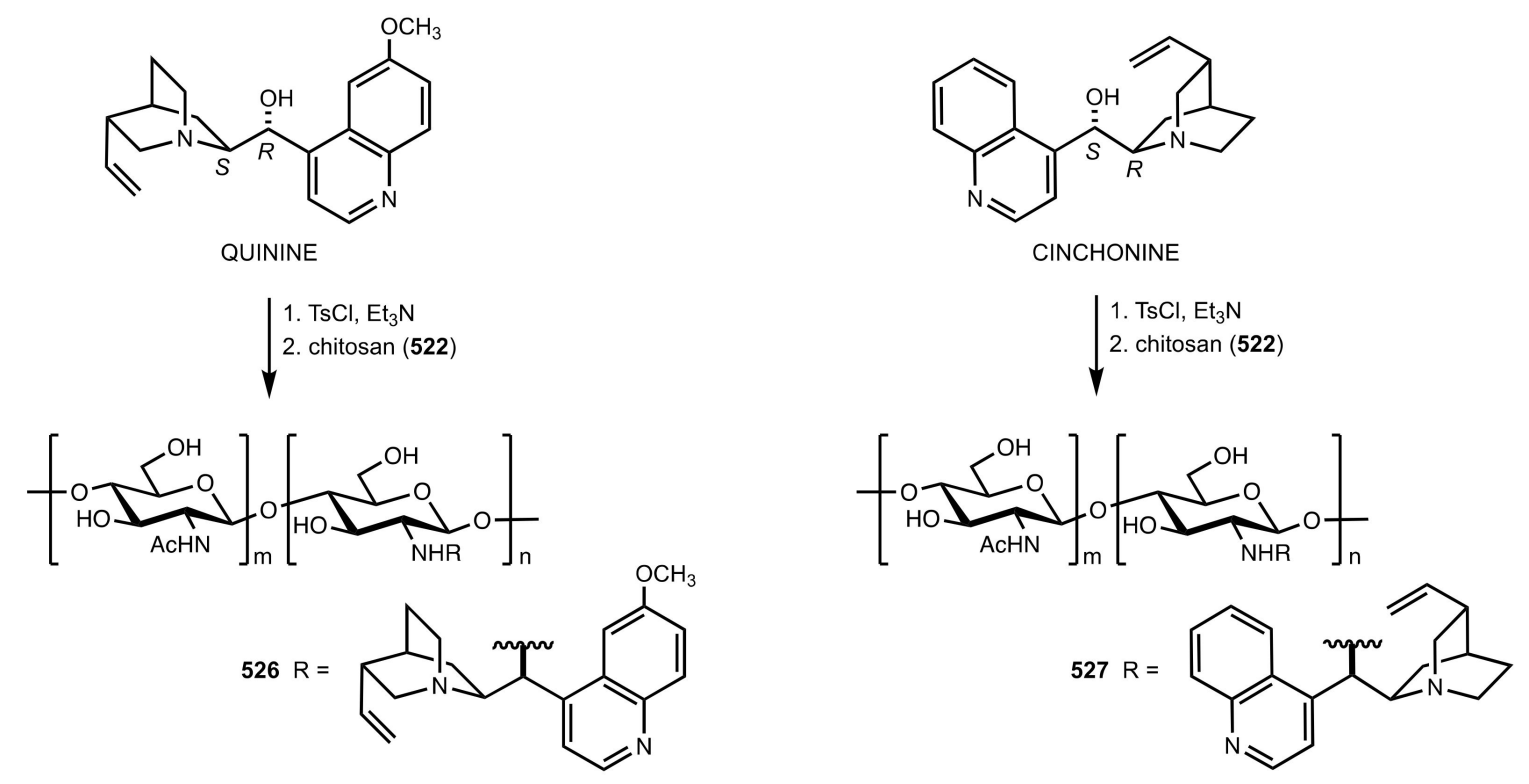
The synthesis of chitosan-quinine **529** and chitosan-cinchonine **530** organocatalysts.

Cui and co-workers studied [232] the Michael addition of 1,3-dicarbonyl compounds to *N*-benzyl-maleimide using chitosan bearing cinchona alkaloid units (**526** and **527**, Scheme 142). To prepare the two organocatalysts, the commercially available quinine and its pseudo-enantiomer cinchonine were first tosylated and then allowed to react (S_N_2 reaction) with commercial chitosan **522** (degree of deacetylation: 85%, MW and polydispersity index not reported) to give the corresponding functionalised chitosan **526** and **527** (characterized only by IR analysis). It is worth noting that the authors erroneously drew the structure of cinchonine and the absolute configuration of the C-9 alkaloid carbon of the organocatalysts **526** and **527**.

**Scheme 143:**
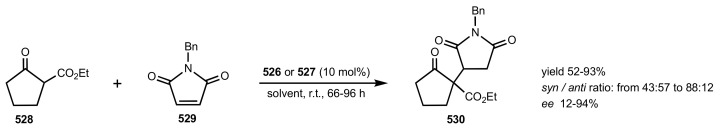
The optimization of the Michael addition to *N*-benzyl-maleimide.

Aiming to find the suitable solvent for the Michael addition in the presence of **526** or **527** (10 mol%), the β-ketoester **528** was coupled with a slight excess of maleimide **529** at room temperature (Scheme 143). It was also found that the two organocatalysts gave the adduct **530** with opposite configuration in a similar enantiomeric excess, although the actual stereochemistry of **530** was not established.

**Figure 41:**
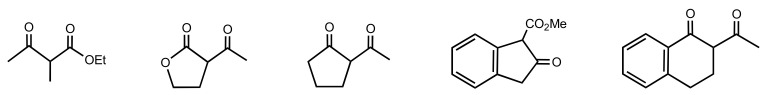
1,3-Dicarbonyl compounds employed in the Michael addition to *N*-benzyl-maleimide.

After the optimization of the reaction conditions, the five dicarbonyl compounds shown in Figure 41 were reacted with **529** in toluene (r.t., 68–78 h) to give the corresponding adducts in good yield and stereoselectivity. However, it was not stated if, also in these cases, the two organocatalysts **526** and **527** afforded the same products with opposite configurations.

**Scheme 144:**
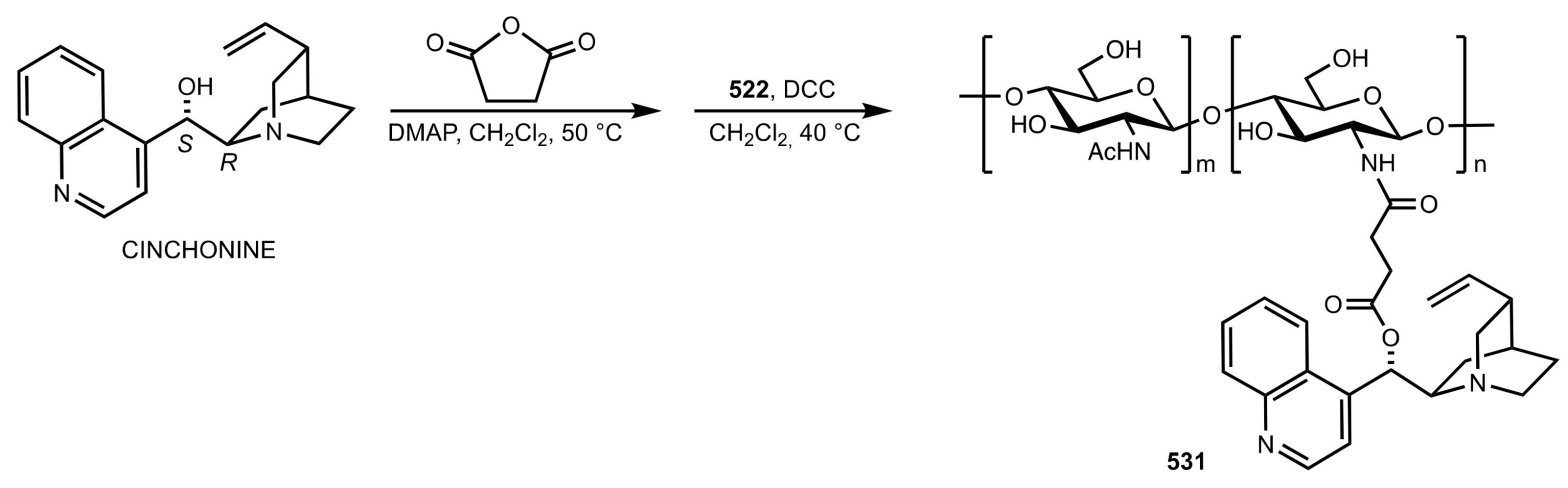
The synthesis of the chitosan-cinchonine organocatalyst **531**.

Three years after their work [232] on the use of chitosan-supported cinchona alkaloids, Cui and co-workers reported [233] another organocatalyst constituted of cinchonine units, retaining the C-9 oxygen and the original configuration, linked to the chitosan through a four-carbon chain (Scheme 144). The cinchonine was esterified with succinic anhydride and dimethylamino-pyridine to give the corresponding free acid that was reacted with chitosan (degree of deacetylation, MW, and polydispersity index not reported) in the presence of dicyclohexylcarbodiimide (DCC) to give **531**, characterized only by IR analysis (loading not reported). Once more, the authors erroneously drew the absolute configuration of the C-9 cinchonine carbon atom.

**Scheme 145:**
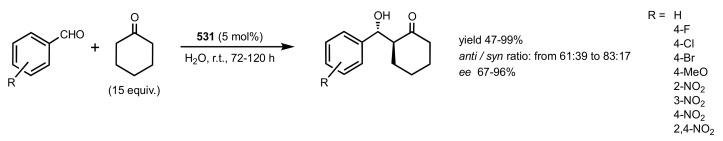
The aldol reactions catalysed by chitosan-cinchonine **531**.

Several aromatic aldehydes were condensed in water with a large excess of cyclohexanone in the presence of **531** (5 mol%) to give, after 3–5 days at r.t., the corresponding aldols in moderate to good yield and stereoselectivity (in one case the enantiomeric excess was 96%) (Scheme 145). The organocatalyst **531** was also recycled five times without loss of chemical activity and diastereoselectivity, although the enantioselectivity slightly decreased (*ee* from 96% to 84%).

**Scheme 146:**
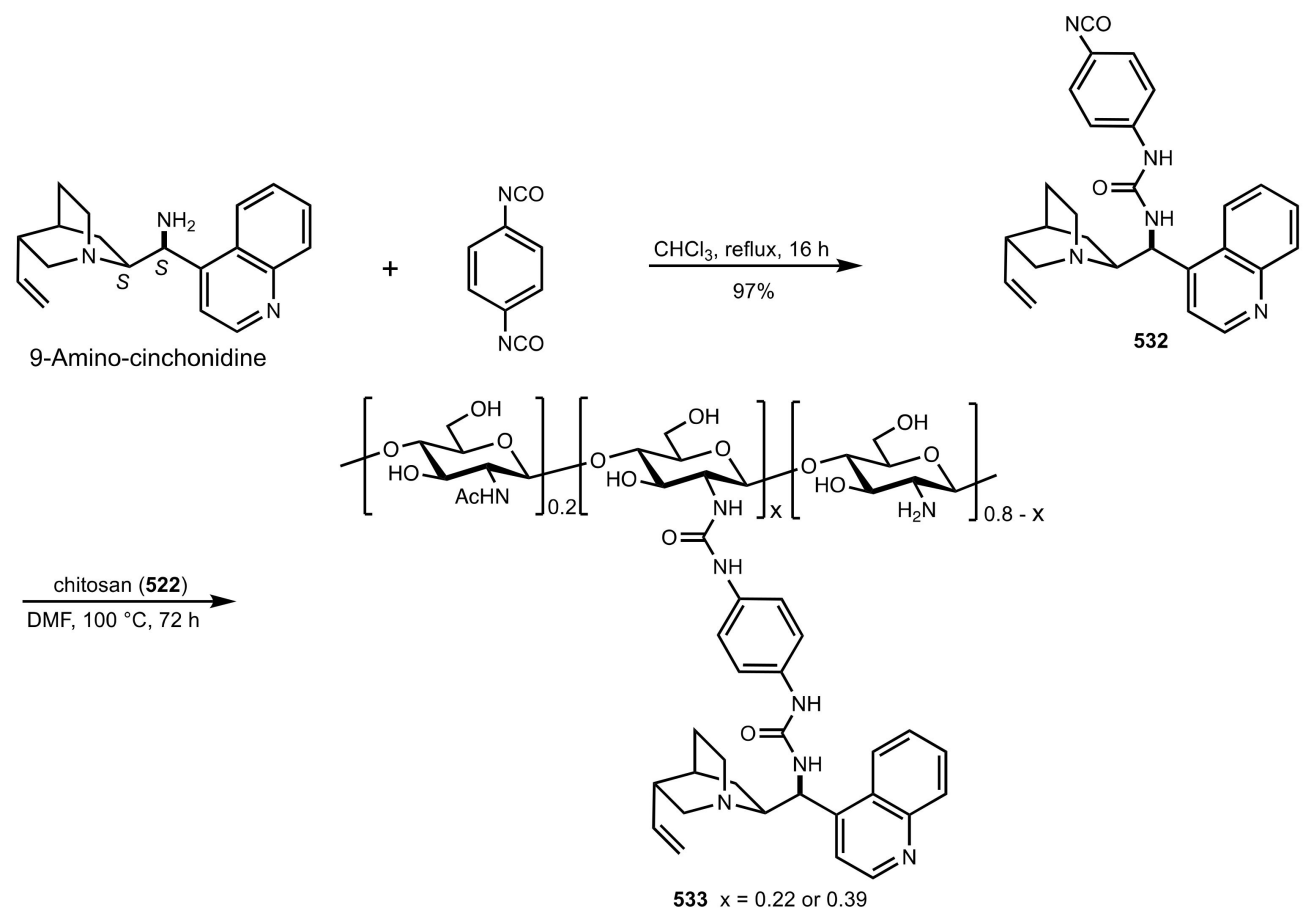
The synthesis of the chitosan-aminocinchonidine **533** organocatalyst.

In their 2020 article [234], Itsuno and co-workers reported the synthesis of a chitosan-linked cinchona alkaloid that was then employed as organocatalyst for the Michael addition. The 9-amino-9-deoxy-cinchonidine was coupled with an equimolar amount of 1,4-phenylene diisocyanate to afford the urea-isocyanate derivative **532** in almost quantitative yield (Scheme 146). Variable amounts of **532** were reacted at high temperature with chitosan **522** (degree of deacetylation = 80%, MW and polydispersity index not reported) to give the chitosan-cinchonidine derivative **533** with different loadings (established by elemental analysis). For comparison purposes, the monomeric analogue of the organocatalyst **533** was also prepared starting from 1,3,4,6-tetra-*O*-acetyl-d-glucosamine.

**Scheme 147:**
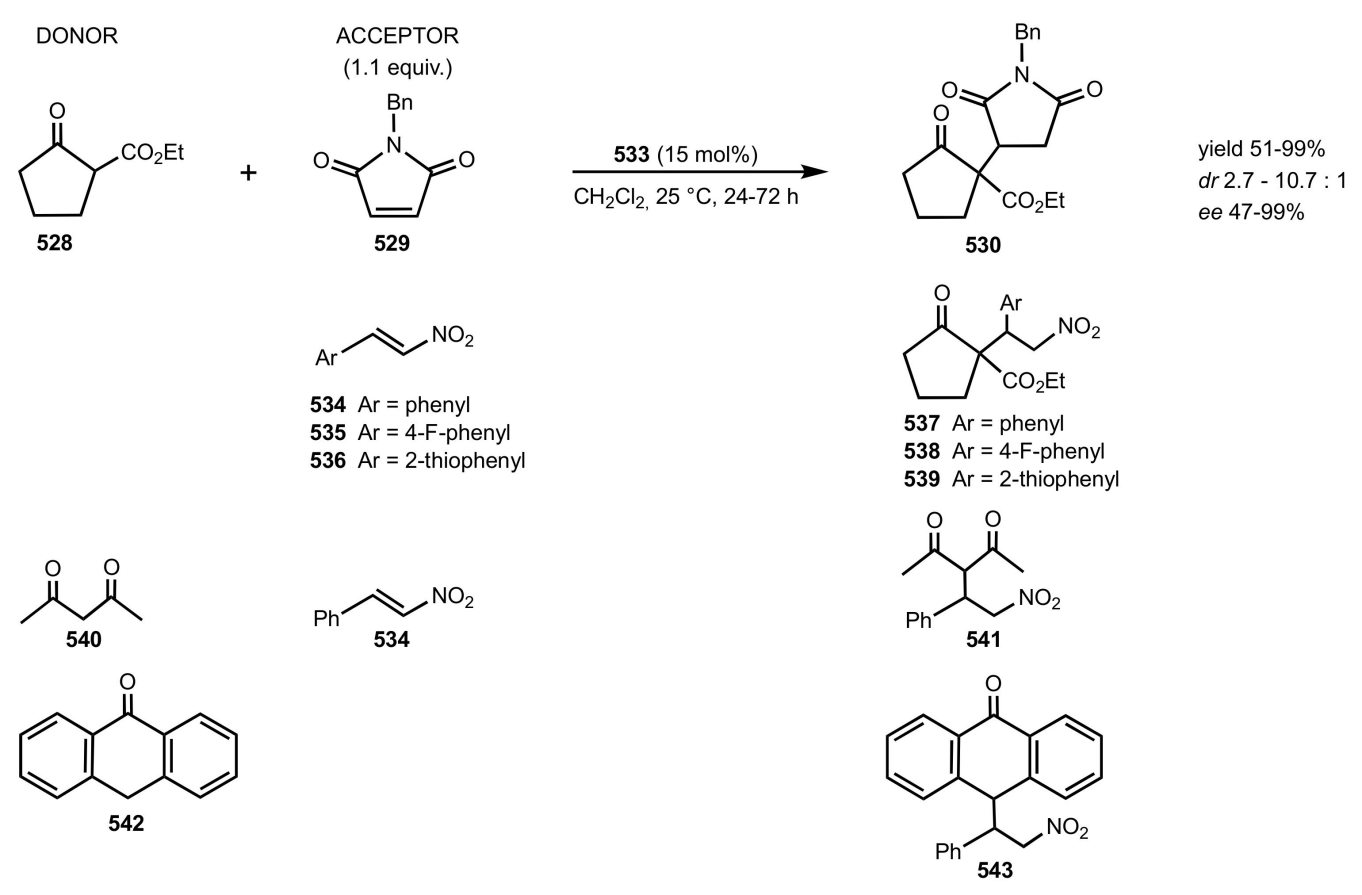
The Michael addition catalysed by the chitosan-aminocinchonidine **533** organocatalyst.

Three mono- and dicarbonyl compounds were reacted with four Michael acceptors under the reaction conditions previously optimised using **528** and **534**, i.e., dichloromethane as the solvent and 15 mol% of **533** with higher loading (x = 0.39) (Scheme 147). When the organocatalyst **533** was recycled (up to three times), they observed no significant loss of yield and enantioselectivity, whereas the diastereoselectivity decreased from 9.3:1 to 3.9:1.

### 7.3. Functionalized Chitosan

**Scheme 148:**
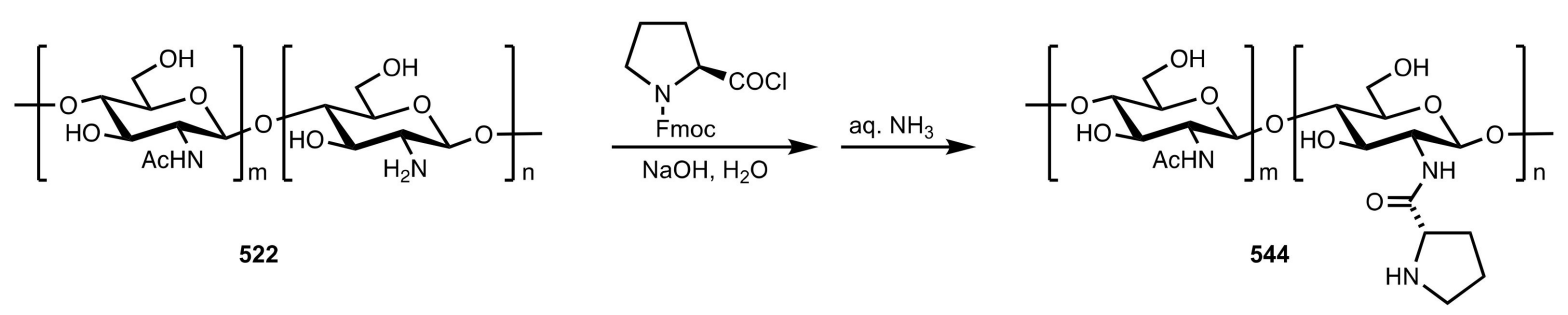
The synthesis of the chitosan prolinamide **544** organocatalyst.

The chitosan prolinamide organocatalyst **544** was prepared [235] by the reaction of commercially available chitosan **522** with the acyl chloride of the Fmoc-l-proline followed by the basic removal of the carbamate group (Scheme 148). Unfortunately, the reaction conditions, yield and characterization data were missing, and only the loading was mentioned (2 mmol per gram of polymer).

An initial screening of the reaction conditions using 4-nitrobenzaldehyde and cyclohexanone allowed establishing that the organocatalyst **544** was more efficient in water than in organic solvents. Moreover, even better results were observed when the reaction was conducted in aqueous micelles formed by addition of ionic or non-ionic surfactants.

**Scheme 149:**
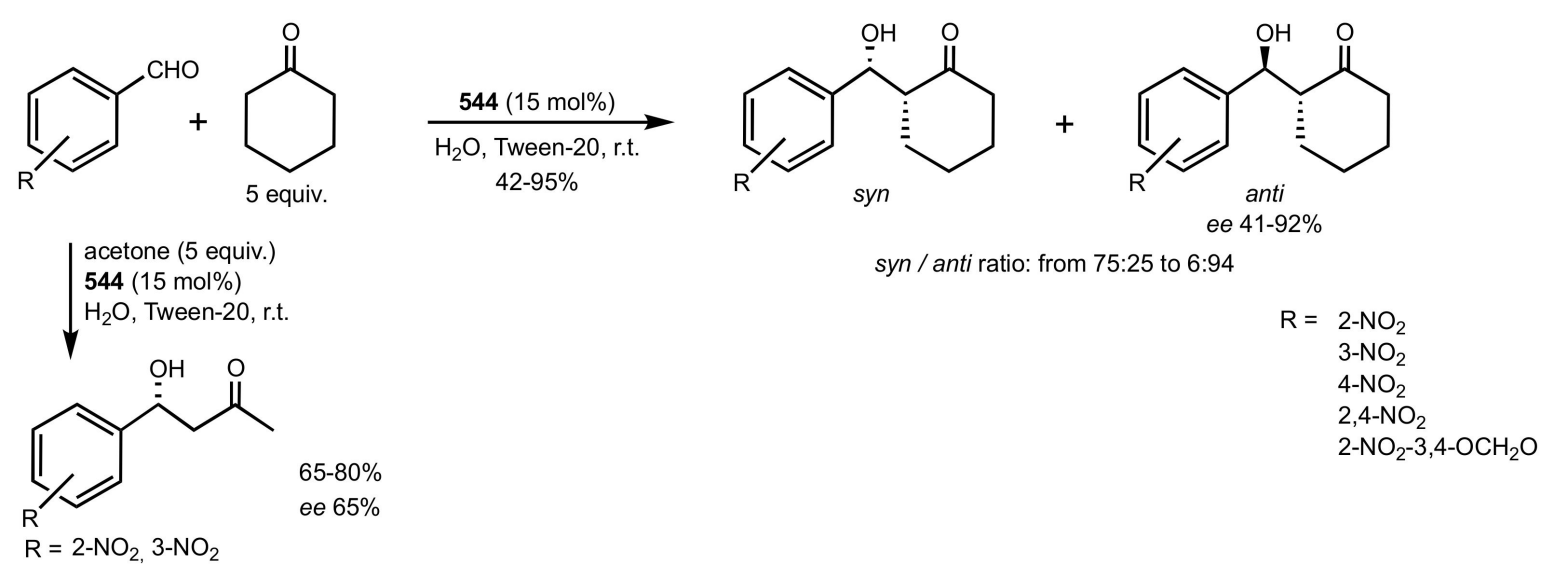
Aldol reactions catalysed by chitosan prolinamide **547**.

Then, a few nitrobenzaldehydes and 4-formyl-pyridine (not shown) were reacted with an excess of cyclohexanone in the presence of Tween-20 (a polysorbate) and 15 mol% of **544** (based on the proline content) to give, in most cases, the *anti*-aldol as the major adduct with enantiomeric excess up to 92% (Scheme 149). The aldol reaction was performed also with acetone using only the *ortho*- and *meta*-nitrobenzaldehyde. After one hour of reaction, the adduct was isolated in good yield and moderate enantiomeric excess.

**Scheme 150:**
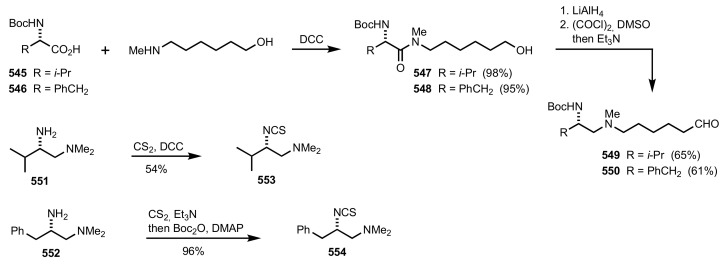
The synthesis of the chiral isocyanates.

In 2017, Andrés, Pedrosa and their co-workers described [236] the synthesis of eight chitosan derivatives bearing a thiourea moiety linked to the 2-amino group through a chiral or achiral chain featuring different length and substituents (aromatic or aliphatic). The chiral aldehydes **549** and **550** were obtained from Boc-protected l-valine (**545**) and l-phenylalanine (**546**) by amidation with 6-(*N*-methylamino)-hexanol, reduction of the amide function and Swern oxidation of the primary alcohol (Scheme 150). The chiral isothiocyanates **553** and **554** were prepared by reaction of the diamines **551** and **552** with either carbon disulfide (CS_2_) and dicyclohexylcarbodiimide (DCC) or CS_2_ in triethylamine followed by treatment with di-*t*-butyl dicarbonate and dimethylamino-pyridine (DMAP) as reported in their previous article [237].

**Scheme 151:**
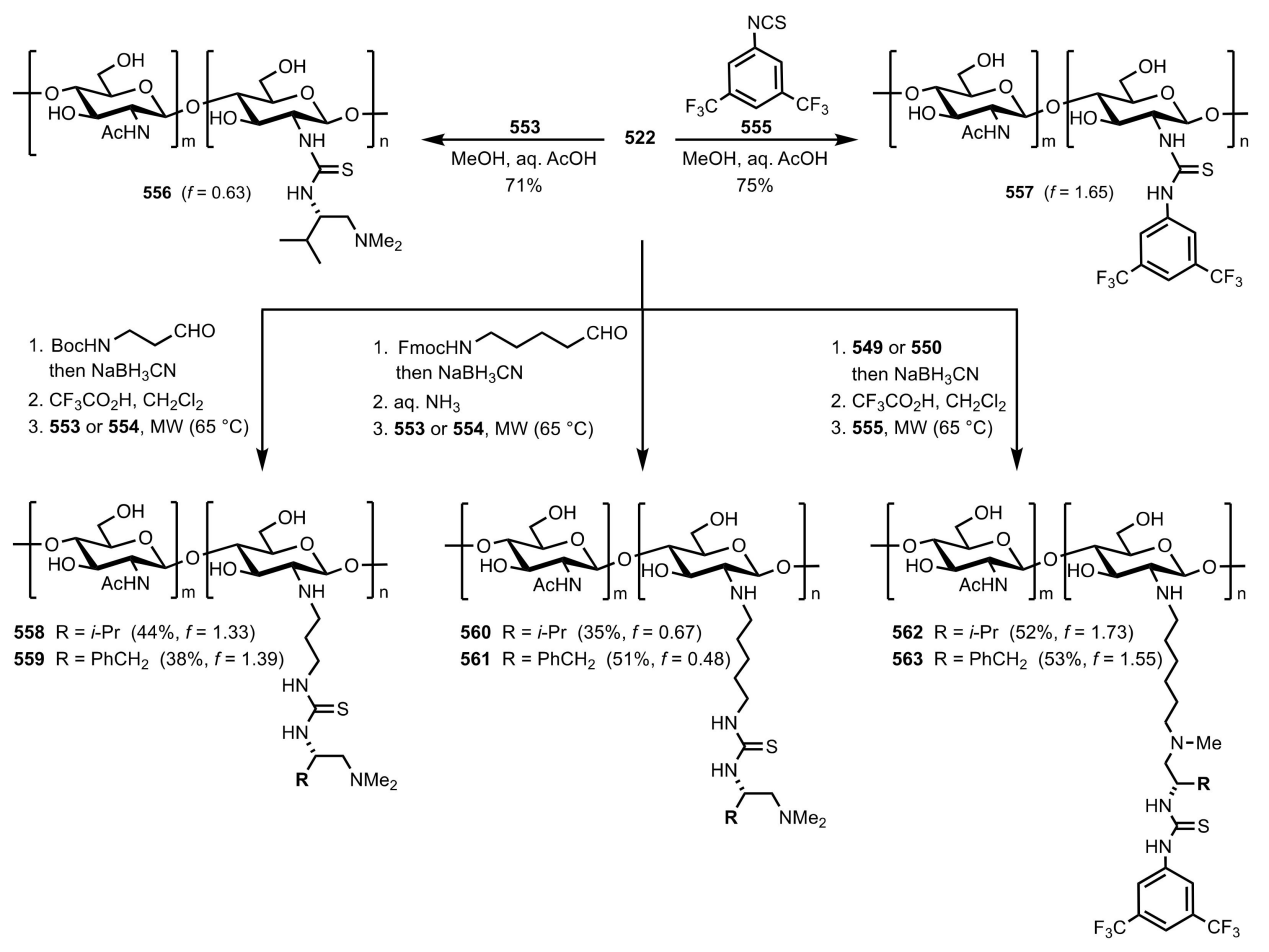
The synthesis of the chitosan-thiourea organocatalysts.

To prepare the organocatalysts, the commercial chitosan **522** (degree of deacetylation: 94.5%, MW = 600–800 kDa) was treated with the chiral isothiocyanate **553** or the aromatic isothiocyanate **555** to afford the chitosan-thioureas **556** and **557** (Scheme 151), respectively, displaying different *f* values (effective functionalization, in mmol/g, calculated by elemental analysis of the sulphur content). The chitosan **522** was also submitted to reductive amination with achiral and chiral *N*-protected aminoaldehydes to afford, after acid or basic treatment, the corresponding free amine derivatives that were reacted with the isocyanates **553–555** to give the desired chitosan-thioureas **558–563** in good overall yield (*f* values from 0.48 to 1.73).

The eight organocatalysts **556–563** (10 mol%) were first employed in the addition of nitromethane (six equiv.) to the *N*-Boc-benzaldimine (aza-Henry reaction) at room temperature without solvent. It was found that, like the chitosan **522**, the thiourea derivatives **556** and **557** were totally unreactive, whereas the catalysts **558–563** afforded the adducts in good yield (62–72%) and enantioselectivity (*er* from 82:18 to 93:7).

**Scheme 152:**
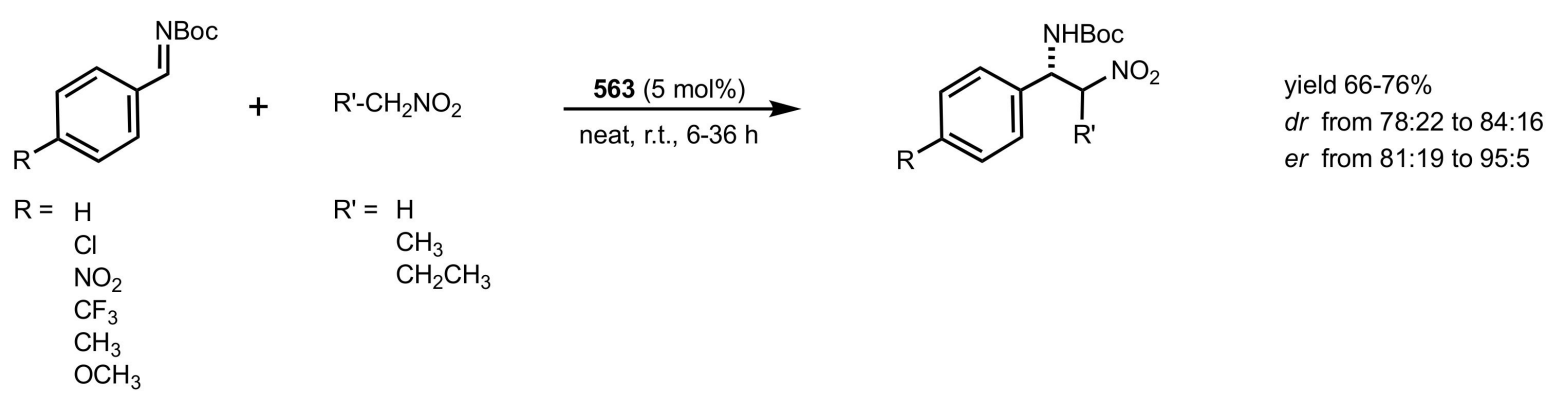
The Aza–Henry reaction catalysed by the chitosan-thiourea organocatalyst **566**.

As the best results were obtained with **563**, this compound was used at 5 mol% to catalyse the aza-Henry reaction between variously substituted aromatic imines and three simple nitroalkanes (Scheme 152). The imines bearing electron-withdrawing groups were significantly more reactive (reaction time: 6–9 h) than those containing electron-donor substituents (20–36 h). The organocatalyst **563** was also recycled four times without any loss of enantioselectivity, although the yield decreased from 74% to 64%.

### 7.4. Functionalized Amylose and Cellulose

**Scheme 153:**
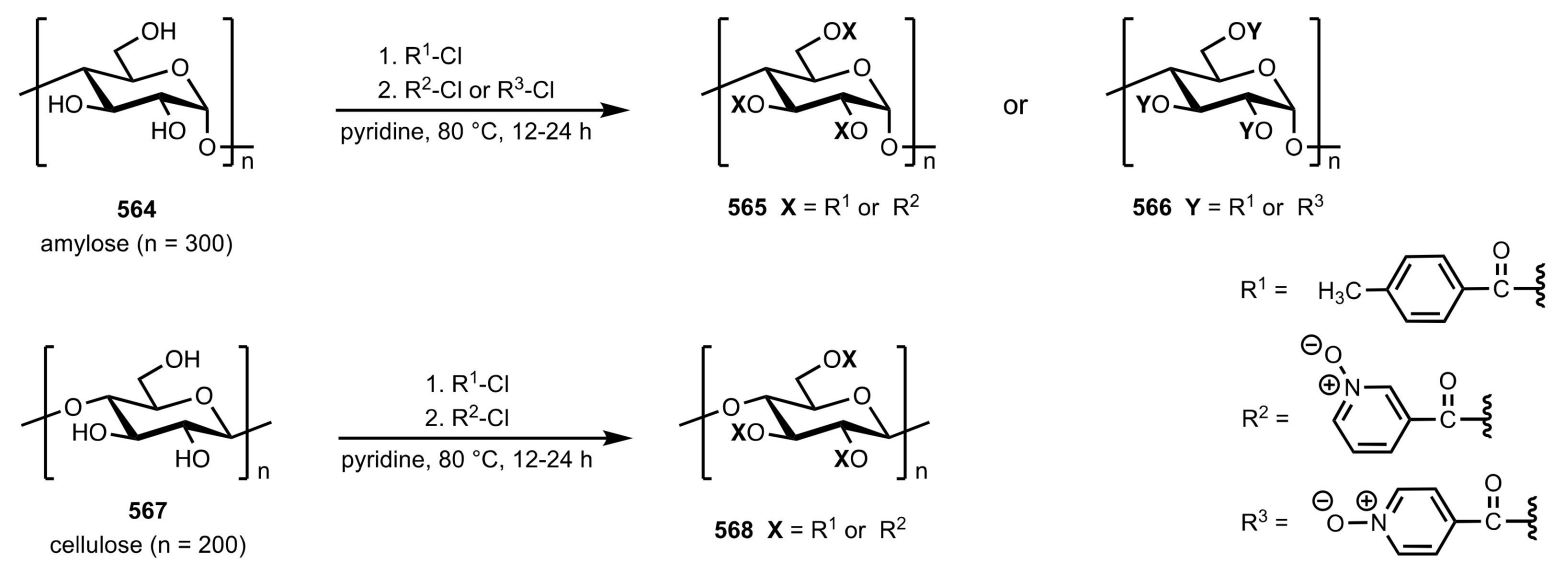
The synthesis of the amylose and cellulose pyridine oxide organocatalysts.

In 2011, Ikai and co-workers reported the first synthesis of amylose- and cellulose-based organocatalysts aiming to catalyse the enantioselective allylation of aldehydes [238]. The commercially available amylose **564** (degree of polymerization = 300) was treated in pyridine at high temperature first with variable amounts of 4-methylbenzoyl chloride (R^1^-Cl) and then with the acyl chloride of nicotinic (R^2^-Cl) or isonicotinic acid *N*-oxide (R^3^-Cl) to afford the amylose esters **565** or **566**, respectively (yield not given) (Scheme 153).

Thus, the polysaccharide **565** could be obtained with three different R^1^/R^2^ ratios ranging from 4:1 to 2:1 (established by elemental analysis), whereas the amylose derivative **566** was prepared only with a fixed 2:1 R^1^/R^3^ ratio. When commercially available cellulose **567** (degree of polymerization = 200) was treated as reported for the preparation of **565**, the corresponding polysaccharide ester **568** with three different loadings of pyridine *N*-oxide moieties was obtained (R^1^/R^2^ ratios from 4:1 to 2:1). For comparison purposes, a monomeric analogue of **566** and **568** was also prepared by reaction of d-glucose with 4-methylbenzoyl chloride and the acyl chloride of isonicotinic acid *N*-oxide (R^1^/R^2^ = 3:1).

**Scheme 154:**
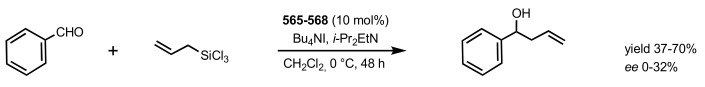
The allylation of benzaldehyde catalysed by the amylose and cellulose pyridine oxide organocatalysts.

The activity of the organocatalysts **565–568** was evaluated in one model reaction, i.e., the addition of allyltrichlorosilane (1.2 equiv.) to benzaldehyde in the presence of tetrabutylammonium iodide (1.2 equiv.) and Hünig’s base (five equiv.) in dichloromethane to give the corresponding secondary alcohol (Scheme 154). The adduct was recovered in moderate yield in all cases but unsatisfactory enantioselectivity was observed for the amylose catalyst **565** (13–32%) and the cellulose analogue **568** (2–11%), while the amylose derivative **566** showed no enantioselectivity at all.

Interestingly, the major adduct was (*R*)-configured in the presence of organocatalyst **565** and (*S*)-configured when **568** was used, regardless of the pyridine *N*-oxide content. As indicated also by the circular dichroism studies performed by the authors, this result suggests that the ester units in **565** and **568** are arranged in opposite chiral environments inside the polysaccharide backbone. Finally, it is worth noting that the monomeric glucose-based organocatalyst gave results very close (55% yield, 8% *ee*) to those observed for the cellulose-based catalyst **568**.

## 8. Phase-Transfer Catalysts

The use of a catalyst allowing the interphase transfer of chemical species to promote reactions between reactants in two immiscible phases was proposed in the 1960s by Mąkosza [239] and the term “phase transfer catalysis” (PTC) was introduced by Staks in 1971 [240]. Tetraalkyl-ammonium or -phosphonium salts were used in the early works but various onium salts [241], including chiral catalysts [242,243,244], were then developed.

Alternatives to ionic phase transfer catalysts, macrocyclic polyethers (crown ethers) were also used as neutral ligands capable of complexing and transporting alkali metal cations into an organic phase [245,246]. The use of chiral crown ethers in PTC was first proposed by Cram and Sogah in 1981 [247], and after this pioneering work many chiral macrocycles [8], including compounds whose chirality is introduced by one or more sugars [5,6,7], were prepared and assayed in asymmetric catalysis.

### 8.1. Sugar Ammonium and Triazolium Salts

**Scheme 155:**
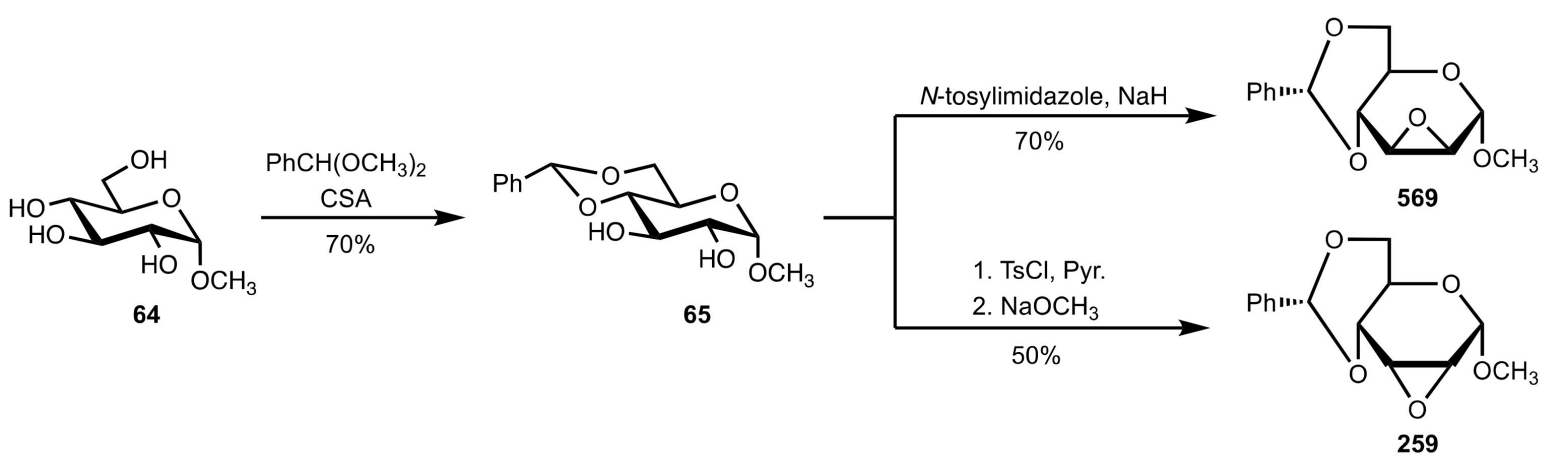
The synthesis of protected sugar epoxides.

Chiral ammonium and triazolium salts were synthetized either by grafting an amine to the carbohydrate scaffold in different positions by regioselective opening of epoxides or using an amino sugar [248]. The d-*manno* (**569**) [249] and d-*allo* (**259**) [250] epoxides were prepared from the same diol **65**, obtained by treatment of **64** with α,α-dimethoxytoluene and 10-camphorsulfonic acid (CSA) under two different reaction conditions (Scheme 155).

**Scheme 156:**
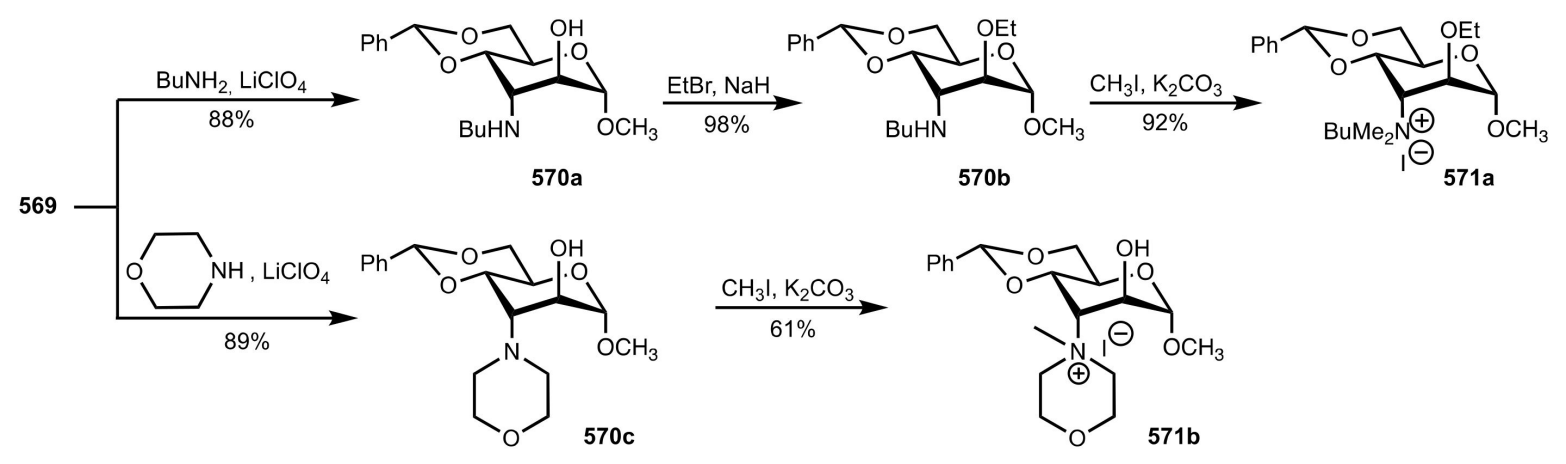
The synthesis of altrose-based quaternary ammonium salts.

Altrose-based quaternary ammonium salt **571a** was prepared from **569** by oxirane opening with butylamine, *O*-alkylation and quaternization with methyl iodide, whereas **571b** was obtained by reaction of **569** with morpholine followed by quaternization (Scheme 156) [248].

**Figure 42:**
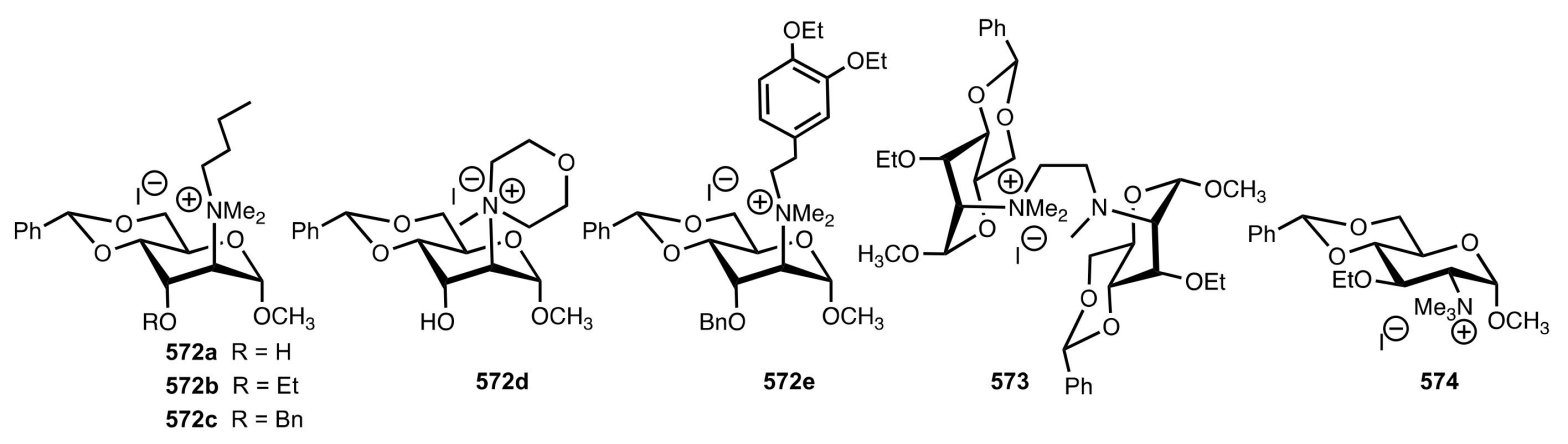
Sugar-based quaternary ammonium salts.

Epoxide **259** (see Scheme 155) was the starting material for the preparation of catalysts having an axial ammonium function in position 2, such as the d-*altro* configured **572a-e** and **573** (Figure 42) [248]. The glucose-based quaternary ammonium salt **574** was obtained at a 90% yield from methyl 4,6-*O*-benzylidene-α-d-glucosamine (**498**, see Scheme 128) [251] by selective alkylation of the OH group with ethyl bromide and quaternization of the amine with methyl iodide [248].

**Scheme 157:**
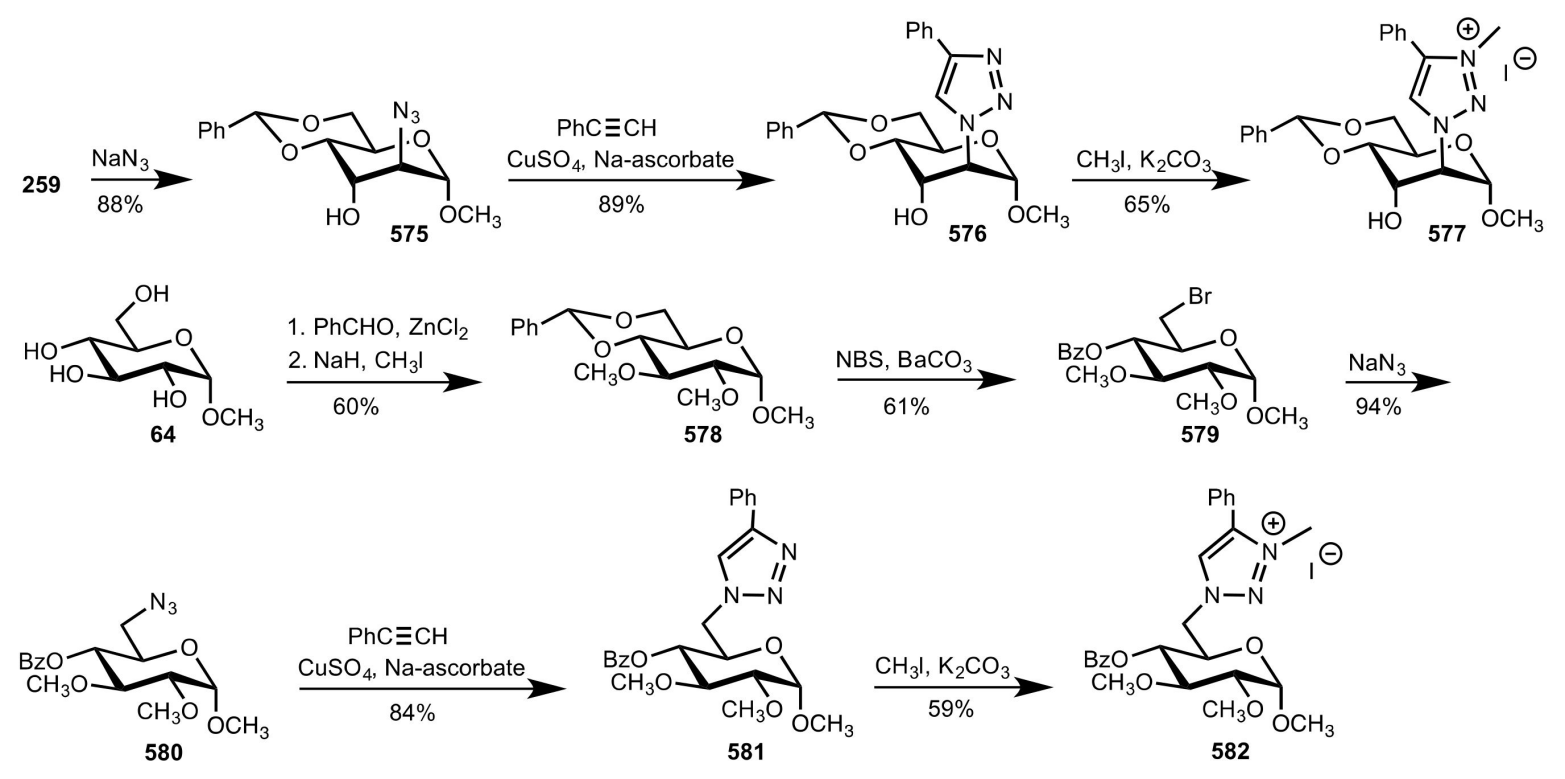
The synthesis of sugar triazolium salts.

In order to have positively charged sugars, the triazolium salts **577** and **582** were also prepared [248]. The d-*altro* derivative **577** (Scheme 157) was obtained from epoxide **259** by reaction with sodium azide followed by copper(I)-catalysed 1,3-dipolar cycloaddition (CuAAC) with phenylacetylene and quaternization with methyl iodide. The same reaction sequence was applied [248] to the known [46] azido sugar **580**, which was, in turn, easily synthesised [47] from sugar bromide **579** (Scheme 157).

**Scheme 158:**
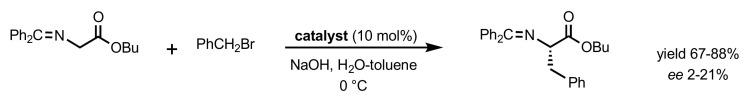
Alkylation of glycine ester catalysed by quaternary ammonium (**571a,b**, **572a-e**, **573**, **574**) or triazolium (**577** and **582**) salts.

The sugar-based quaternary ammonium (**571a,b**, **572a-e**, **573**, **574**) or triazolium (**577**, **582**) salts were used as liquid–liquid phase transfer catalysts for the alkylation of *N*-(diphenyl)methylene glycine *tert*-butyl ester with benzyl bromide (Scheme 158) [248]. Although the yields were acceptable (67–88%), the enantiomeric excesses were rather disappointing (2–21%). The best result was obtained with phase transfer catalyst **572e** (21% in favour of the (*S*)-enantiomer).

### 8.2. Sugar Crown Ethers

Crown ethers are another class of phase-transfer catalysts generally used for the reactions in solid–liquid systems. Crown ethers containing sugar moieties in the structure were prepared from alditols, monosaccharides, disaccharides and oligosaccharides.

#### 8.2.1. Alditol-Based Crown Ethers

**Scheme 159:**
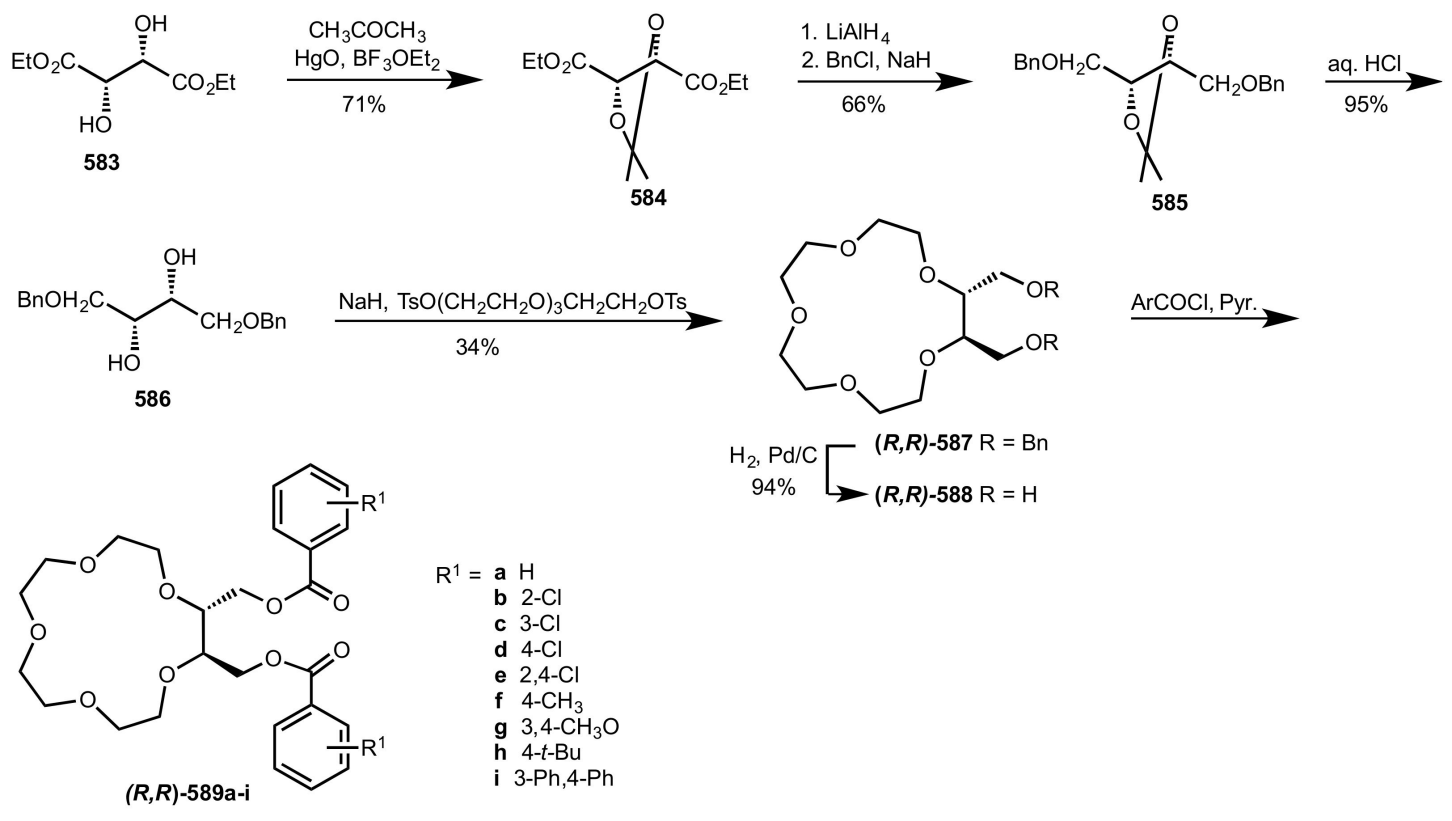
The synthesis of chiral crown ethers from l-tartaric ethyl ester.

**Scheme 160:**
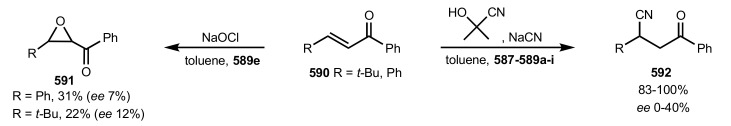
The epoxidation and cyanide addition to enones catalysed by **587**, **588** and **589a-i**.

The 1,2-bis (hydroxymethyl)-15-crown-5 (***R,R***)-**588**, its dibenzyl ether (***R,R***)-**587** and the esters (***R,R***)-**589a-i** were prepared from l-tartaric ethyl ester **583** via standard chemical transformations (Scheme 159) while the (*S,S*)-enantiomers were obtained through the same reaction sequence from d-tartaric ethyl ester. All these compounds were tested as catalysts for the epoxidation of unsaturated ketones **590** (Scheme 160) and the cyanide addition to the latter [252].

The catalyst **589e** was the only one that allowed the epoxidation reaction; however, both yields and *ees* were low. In fact, 1-benzoyl-2-phenyloxirane (**591**, R = Ph) and (3-(*tert*-butyl)oxiran-2-yl)(phenyl)methanone (**591**, R = *t*-Bu) were obtained with a 31% yield, 7% *ee* and 22% yield, 12% *ee*, respectively (Scheme 160). In the case of the Michael addition, all the catalysts **587**, **588** and **589a-i** were effective since the adducts were obtained in 83–100% yields.

Using 20 mol% of catalyst at 20 °C, the enantiomeric excesses were between 0 (with **587**) and 17% (with **589e**). It is noteworthy, however, that in the (*S,S*) series, the diol **588** preferentially formed the (−)-enantiomer, while all the catalyst esters **589** led to the formation of an excess of the (+)-enantiomer. The best results were obtained with **589e** (100 mol%) at −40 °C since **592** (R = *t*-Bu) was obtained in quantitative yield and 40% *ee* (Scheme 160).

**Scheme 161:**
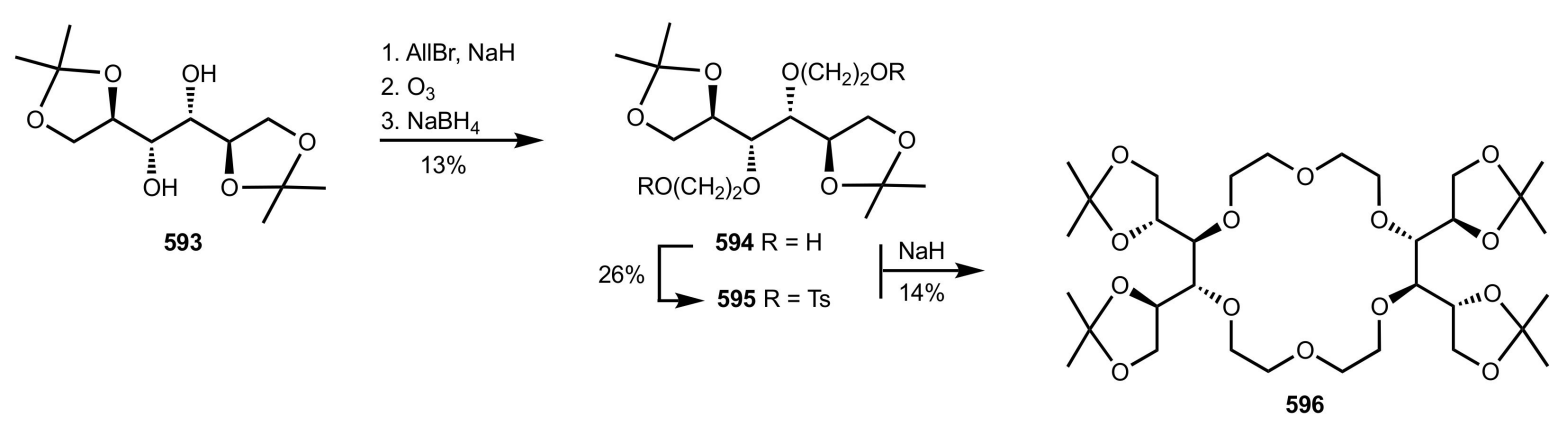
The synthesis of bis-diisopropylidene 18-crown-6 **599**.

Stoddart and co-workers described the synthesis of crown ethers whose structure is based on two alditol units [253]. The bis-diisopropylidene 18-crown-6 **596** was obtained by reacting an equimolar mixture of **594** and **595**, prepared from commercially available 1,2:5,6-di-*O*-isopropylidene-d-mannitol (**593**), in the presence of NaH (Scheme 161). Unfortunately, some analytical data were reported, but the experimental procedures were not provided.

**Scheme 162:**
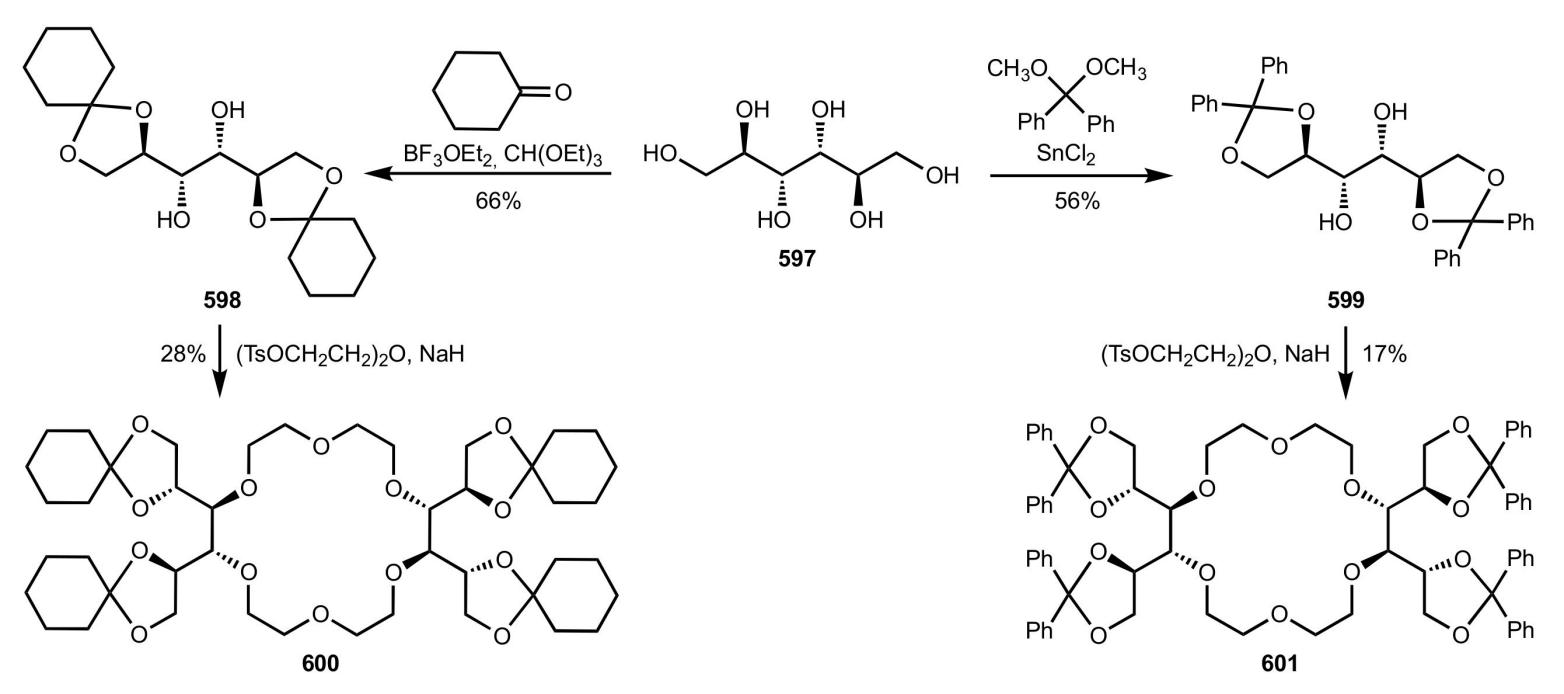
Crown ethers **600** and **601** synthesised by Bakó and co-workers.

Another synthetic approach to crown ethers was implemented by Bakó and co-workers to prepare **600** and **601**, whose mannitol moieties are protected either by cyclohexylidenes or diphenylmethylidenes [254]. 1,2:5,6-Di-*O*-cyclohexylidene-d-mannitol (**598**) [255] and 1,2:5,6-di-*O*-diphenylmethylidene-d-mannitol (**599**) [256] were obtained from commercially available d-mannitol (**597**) using cyclohexanone or benzophenone dimethyl acetal, respectively. Crown ethers **600** and **601** were then synthesised by reaction with diethylene glycol ditosylate in the presence of NaH in 28% and 17% yield, respectively (Scheme 162).

**Scheme 163:**
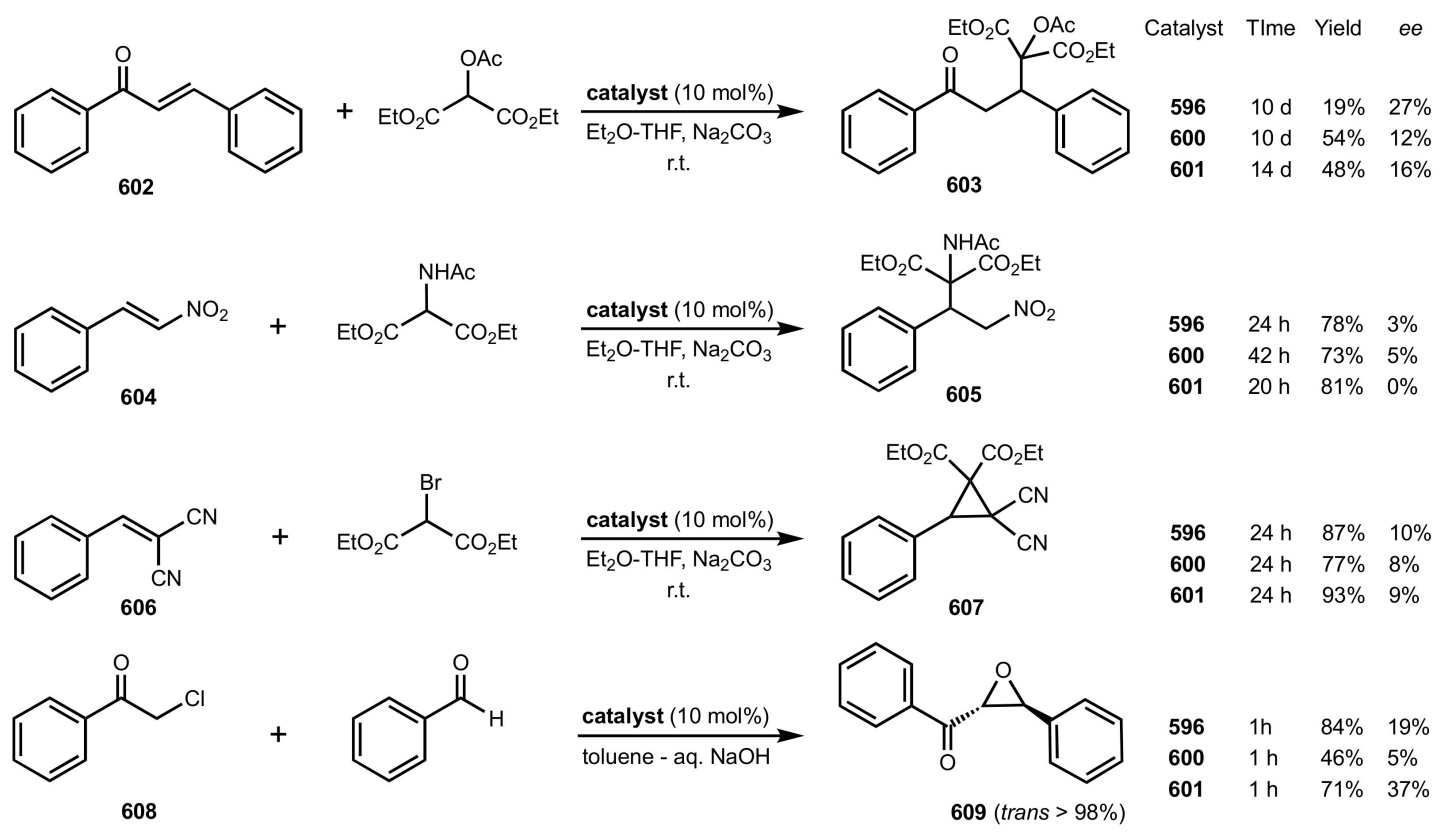
The Michael additions and Darzens condensation catalysed by **596**, **600** and **601**.

The mannitol-based crown ethers **596**, **600** and **601** were used as phase transfer catalysts in Michael additions and in a Darzens condensation (Scheme 163). The conjugate addition of diethyl acetoxymalonate to *trans*-chalcone **602** required 10 to 14 days to reach completion under solid–liquid conditions. The Michael adduct **603** was obtained in moderate yields (19–54%) and poor enantioselectivities (12–27%). Addition of diethyl acetamidomalonate to β-nitrostyrene (**604**) under the same conditions afforded, after 20–24 h, the adduct **605** but none of the chiral crown ether led to a significant asymmetric induction (0–5% *ee*).

Poor enantioselectivity was also obtained for Michael-initiated ring closure reactions giving rise to cyclopropane **607**. The 18-crown-6 type macrocycles were also tested in the Darzens condensation between 2-chloroacetophenone (**608**) and benzaldehyde under liquid–liquid reaction conditions. The *trans*-epoxyketone **609** was obtained in all cases (*de* > 98%), but the enantioselectivity of this isomer remained modest (5–37%) [254].

**Scheme 164:**
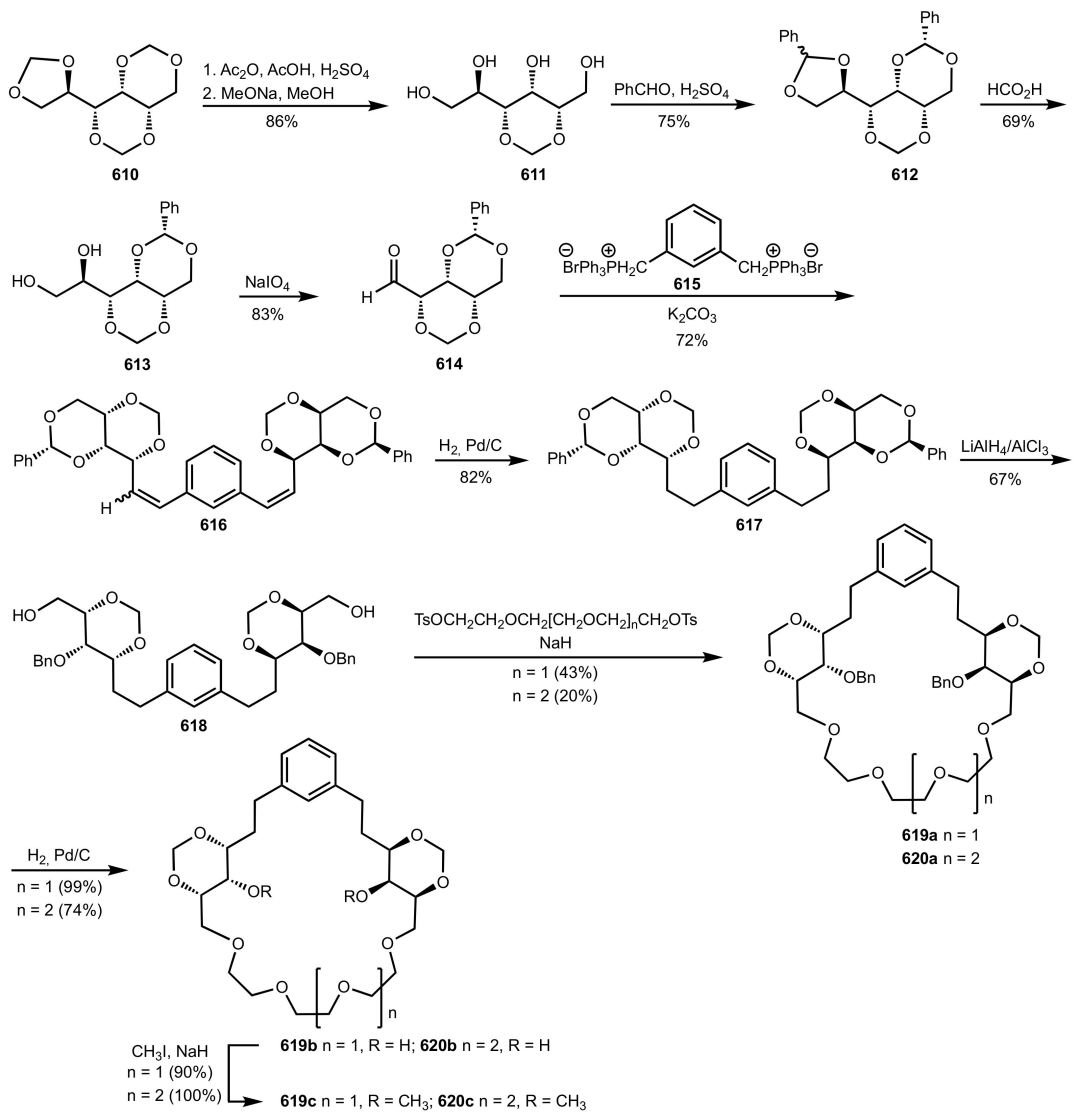
The synthesis of the alditol-based crown ethers **619c** and **620c**.

Alditol-based crown ethers incorporating apolar residues were prepared from d-glucitol [257] or d-xylose (Scheme 164) [258]. Acetolysis of tri-*O*-methylene-d-glucitol **610** followed by deacetylation gave the tetrol **611**, which was treated with benzaldehyde in the presence of sulphuric acid to afford the dibenzylidene derivative **612**. The dioxolane benzylidene acetal was then hydrolyzed to give the diol **613** that, after oxidation with sodium periodate, led to the aldehyde **614** in 83% yield.

Wittig reaction of the latter with the bis-phosphonium bromide **615 [259]** gave **616,** which was hydrogenated and submitted to regioselective reductive opening reaction of the benzylidene groups to afford **618**. This diol gave the macrocycles **619a** or **620a** upon reaction with the appropriate ditosylated ethylene glycol derivatives. Hydrogenolysis of the benzyl ethers afforded **619b** and **620b** that were then methylated to give **619c** and **620c** [258].

**Scheme 165:**
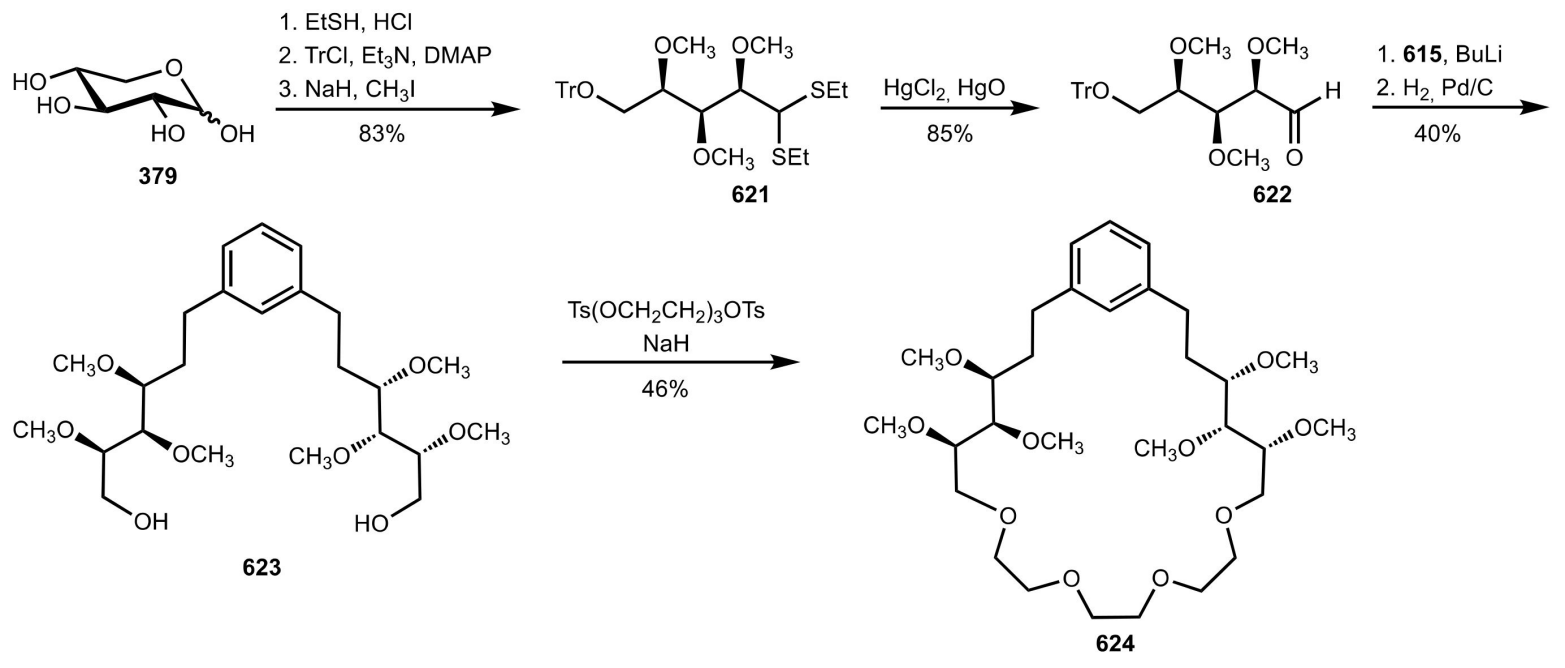
The synthesis of the permethylated crown ether **624**.

Using a similar strategy, the permethylated compound **624** was obtained in seven steps from d-xylose (**379**) (Scheme 165) [258].

**Scheme 166:**
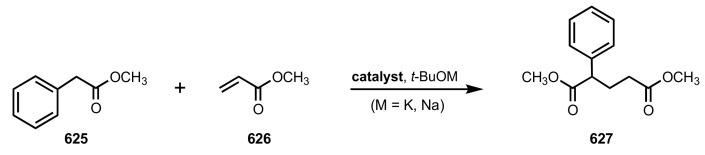
The model Michael addition catalysed by crown ethers **619a-c**, **620a,b** and **624**.

The crown ethers **619a-c**, **620a,b** and **624** were employed as solid–liquid chiral phase transfer catalysts in the model Michael addition of methyl phenylacetate **625** to methyl acrylate **626** in the presence of sodium or potassium *t*-butoxide to afford the adduct **627** (Scheme 166). Under classical conditions (toluene as the solvent, −78 °C, 2 equiv. of **625**, 5 mol% of the catalyst), no reaction took place with the methylated crown ethers **619c** and **620c,** whereas a low yield (16%) and very low enantioselectivity (<2%) were observed with **619b**.

The catalyst **619a** gave the adduct in good yield (92%) but very low enantioselectivity (<2%). The best results were obtained with **620a** and **624** (90 and 83% yield, 58 and 53% *ee*, respectively). These two catalysts, although being derived from l- (**620a**) or d-xylose (**624**) gave rise to an asymmetric induction in favor of the same (*S*)-enantiomer.

#### 8.2.2. Aldose-Based Crown Ethers

**Scheme 167:**
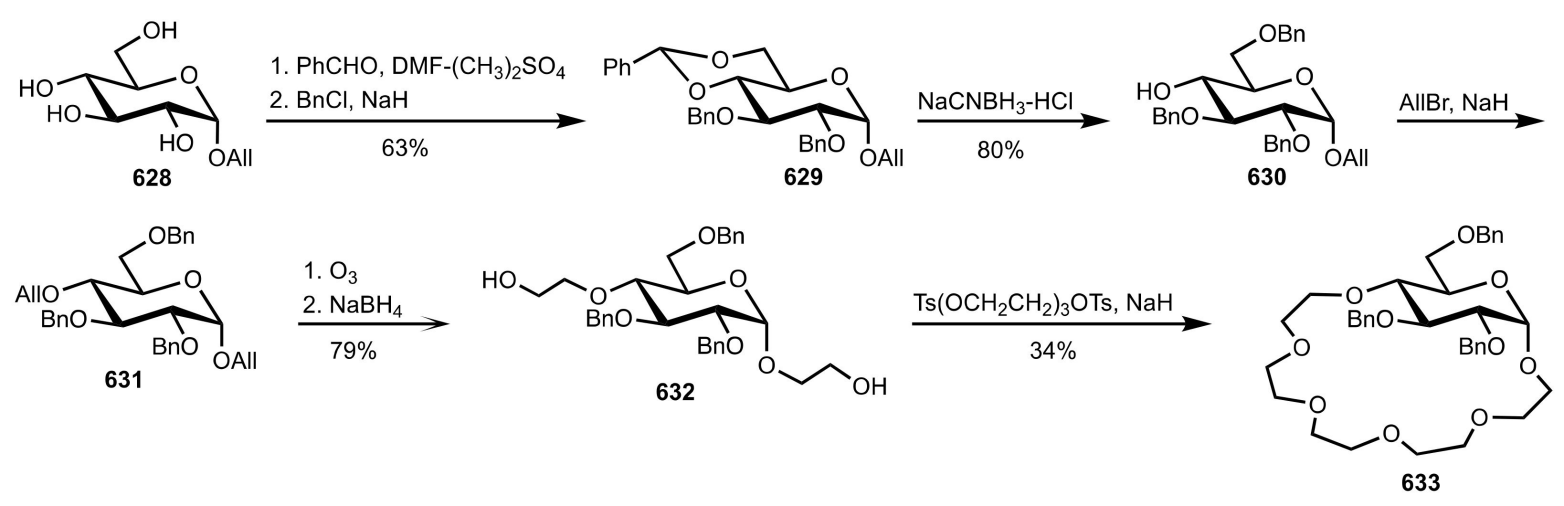
The synthesis of the glucose-based crown ether **633**.

Nair and co-workers envisaged to link a crown ether to a glucose unit through its 1- and 4-hydroxy groups [260]. Regioselective protection of position 4 and 6 of the commercially available allyl glucopyranoside **628** followed by benzylation of the remaining free hydroxyl groups was achieved with a 63% yield (Scheme 167). The regioselective reductive ring opening of the 4,6-*O*-benzylidene acetals with sodium cyanoborohydride—hydrogen chloride afforded **630** whose position 4 was allylated (yield not given). Ozonolysis the di-allylated compound **631** followed by a reductive work-up gave the diol **632** in 79% yield. Subsequent cyclization reaction with triethylene glycol ditosylate afforded the gluco-20-crown-6 **633**.

The catalytic activity of **633** in the model Michael addition of methyl phenylacetate **625** to methyl acrylate **626** (see Scheme 166) was examined using potassium *tert*-butoxide as the base and toluene as the solvent. At −78 °C, with 2.6 equiv. of methyl phenylacetate and 6 mol% of the base and the crown ether, the compound **627** was obtained at a 30% yield and 63% *ee* in favour of the (*S*)-enantiomer, whereas no reaction was observed when 1.3 equiv. of **625** and 33 mol% of base and crown ether **633** were used. Surprisingly enough, under the latter conditions (at 30 °C), the (*R*)-enantiomer was mainly obtained (*ee* = 47%, 15% yield); whereas, under the former conditions (same temperature), the reaction did not proceed. No explanation was provided to justify these observations.

**Scheme 168:**
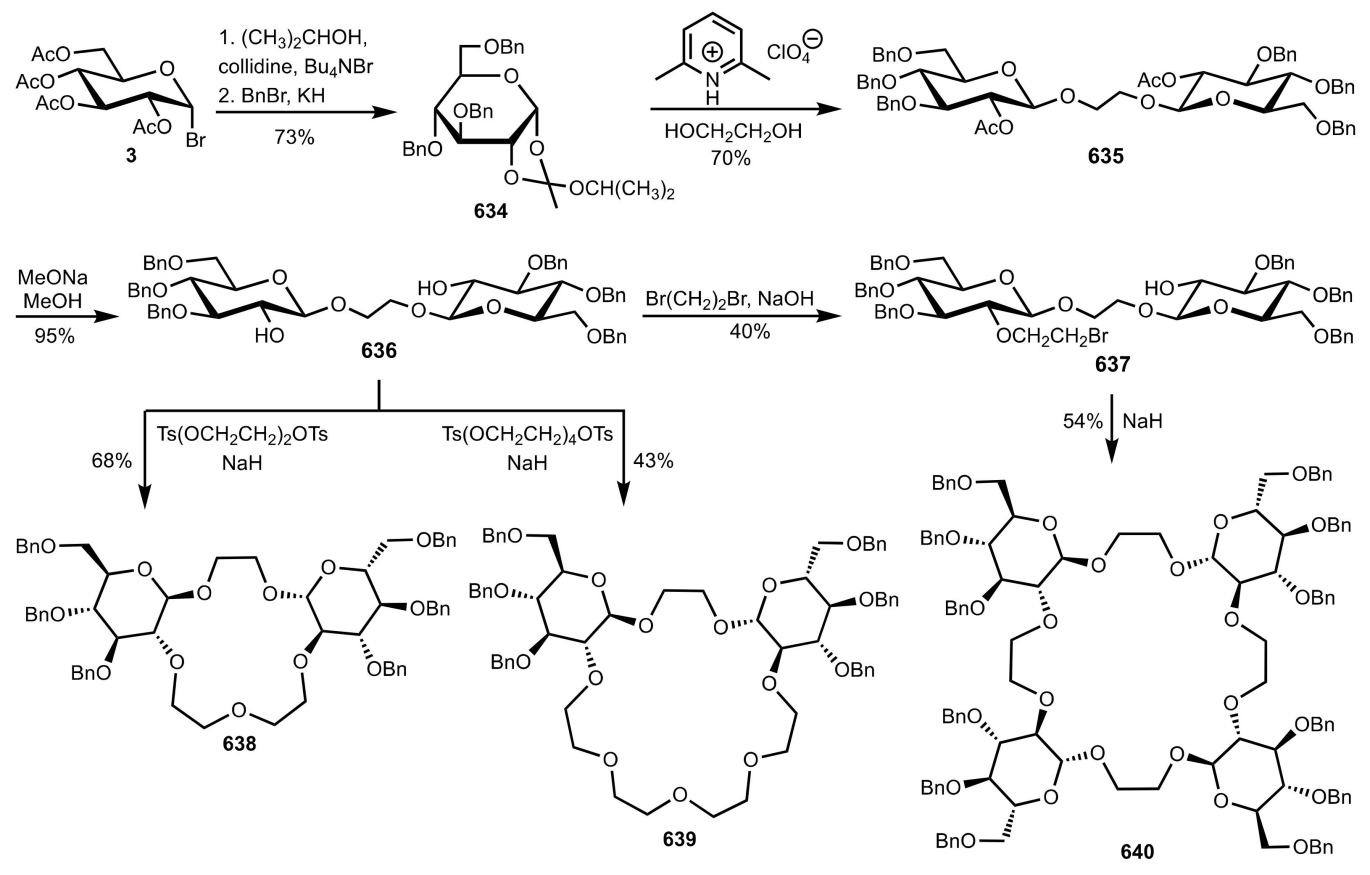
The synthesis of crown ethers bearing two and four d-glucose units.

Penadés and co-workers described the synthesis of chiral macrocycles with different cavity shapes bearing two or four d-glucose units from the same compound **636** obtained by glycosylation of ethylene glycol with two equiv. of orthoester **634** and then deacetylation (Scheme 168) [261]. The glycosyl donor **634** was easily obtained from the glucosyl bromide **3** (see Scheme 1) in 73% yield [262].

The reaction of **636** with di- or tetra- ditosylated ethyleneglycol gave the 15-crown-5 **638** or the 21-crown-7 **639** in 68 and 43% yield, respectively. To prepare the crown ethers containing four glucose units, the monoalkylation of symmetric compound **636** was conducted under phase transfer conditions to afford **637,** which was submitted to intermolecular cyclization to give the 24-crown-8 **640** in 54% yield.

The crown ethers **638–640** were tested as solid–liquid chiral phase transfer catalysts (5–6 mol%) in the model Michael addition (see Scheme 166) of **625** (1.3 equiv.) to methyl acrylate **626** in the presence of potassium *t*-butoxide (0.3–2 equiv.), using toluene as the solvent at low temperature (−78 °C), to afford the adduct **627** in good yield (72–81%). Crown ethers **638** and **640** gave a selectivity in favour of the (*S*)-enantiomer (*ee* = 40 and 24%, respectively), whereas the macrocycle **639** led to the opposite enantiomer (12% *ee*). This difference was not been commented by the authors.

**Scheme 169:**
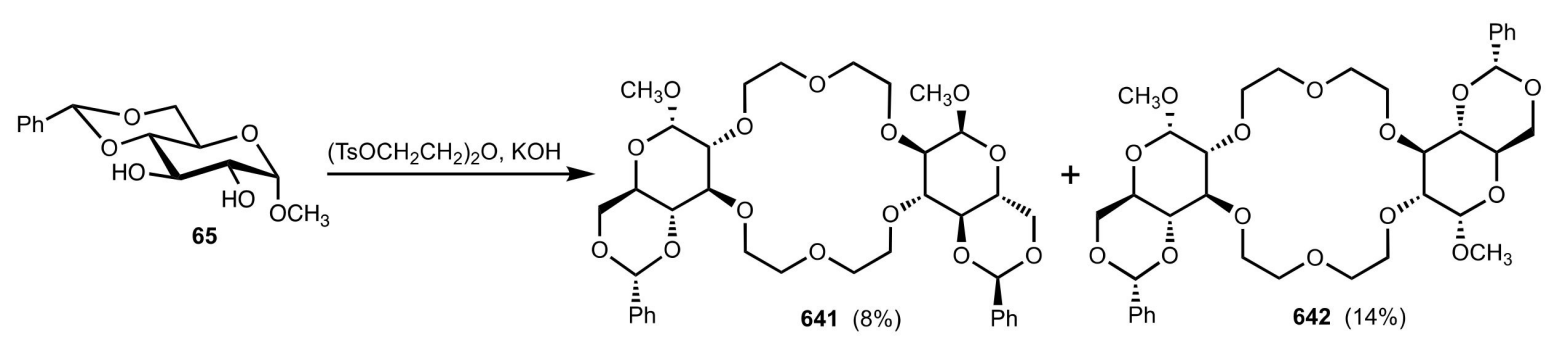
The synthesis of d-glucose-based *C*_2_-symmetric crown ethers.

Bakó and co-workers reported the synthesis of crown ethers incorporating two glucopyranoside units and exhibiting a *C*_2_ symmetry axis (Scheme 169). To this end, they applied an approach similar to that employed to prepare crown ethers bearing two mannitol units. The glucopyranoside **65** (see Scheme 155) was reacted with diethylene glycol ditosylate in a liquid/liquid two-phase reaction to give the isomers **641** and **642**, which were isolated at 8% and 14% yield, respectively [263].

**Figure 43:**
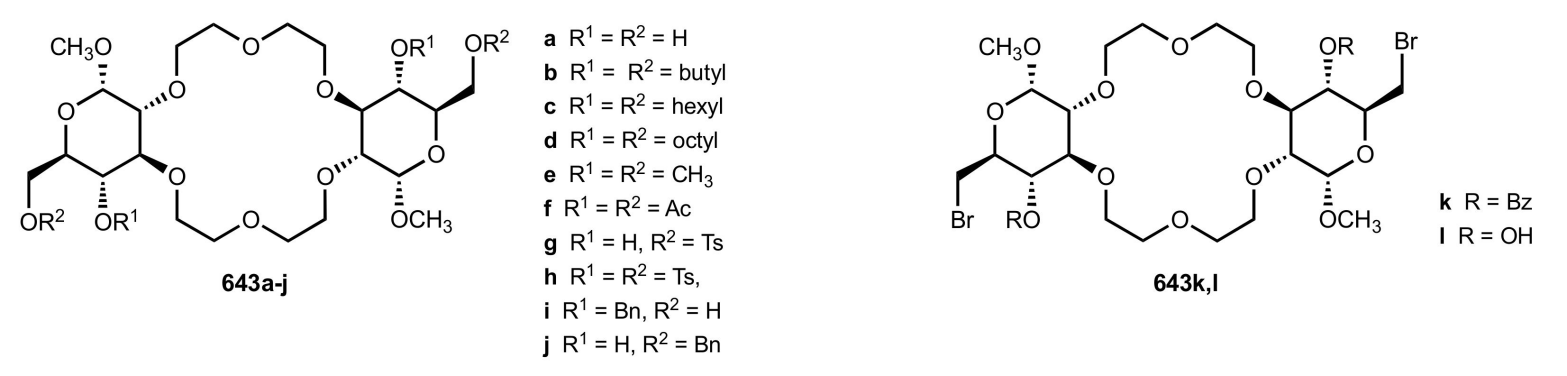
The synthesis of a series of *C*_2_-symmetric crown ethers.

From **642**, various manipulations of the protective groups allowed for the preparation of a large number of derivatives (**643a-l**, Figure 43). The hydrolysis of the benzylidene protecting group afforded the tetrol **643a** [263], which, by alkylation with butyl- [264], hexyl- or octyl bromide [265] in the presence of aqueous sodium hydroxide and tetrabutylammonium bromide in the case of the hexyl derivative, led to the compounds **643b-d** with 84%, 46% and 77% yields, respectively. Methylation of the hydroxyl groups of **643a**, performed with dimethyl sulphate, gave the permethylated crown ether **643e** in 85% yield [264]. Compound **643a** was also acetylated [263] (Ac_2_O, pyridine) or ditosylated (tosyl chloride and pyridine) to afford **643f** (76%) or **643g** (66%). The tetratosyl derivative **643h** was also used in catalysis; however, its preparation and characterization were not reported.

The reductive opening of the 4,6-benzylidene acetal of **642** (LiAlH_4_-AlCl_3_) led to a mixture of the benzyl ethers in position 4 (**643i**, 34%) or in position 6 (**643j**, 6%) of the glucose units [263]. Treatment of **642** with *N*-bromosuccinimide, barium carbonate and benzyl peroxide led to **643k** in 88% yield. Surprisingly, the compound could be efficiently debenzoylated to give **643l** (89% yield) by treatment with sodium methoxide without giving rise to a nucleophilic substitution or elimination of the bromine atoms in the primary positions [264].

All these crown ethers anellated to glucose units were effective as catalysts in the model Michael addition described in Scheme 166 and always led to the (*S*)-configured compound **627** (Table 1) [265,266].

The highest asymmetric induction was obtained with the crown ethers bearing fully alkylated sugar units (Table 1, entries 3–8), and, among these catalysts, the butyl derivative **643b** gave the best results (entry 4). Interestingly, the authors observed that the enantiomeric excess depended on the reaction time. Indeed, after one minute, an excess of 84% was observed using **643b** as the catalyst, while the *ee* was 80% after 8 min and 76% after 16 min. This observation led to the hypothesis that deprotonation of the reaction product by the crown potassium base complex and reprotonation of the anion formed could lead to the decrease of the enantiomeric excess. This hypothesis was confirmed by experiments of deracemization of **627** in the presence of the catalysts **643b** and **643c** [265].

**Scheme 170:**
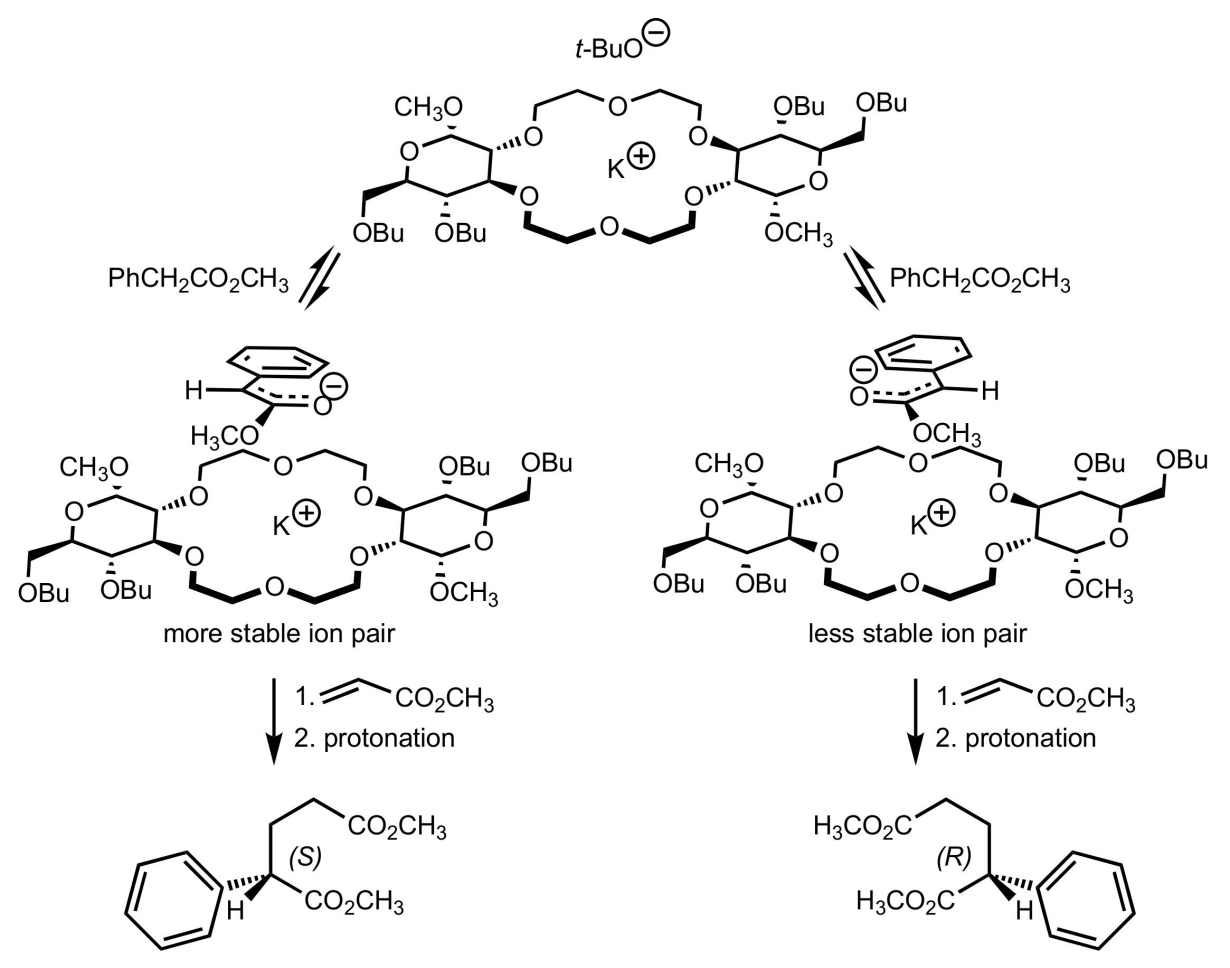
The proposed mechanism for the (*S*)-enantioselectivity of the Michael addition.

The authors also performed molecular modelling studies that supported a mechanism indicating that the (*S*)-enantioselectivity of the reaction was governed by the relative stability of the enolate/potassium-crown ether ion pairs. The addition, under thermodynamic control conditions, of the most stable ion pair to the methyl methacrylate led to the major (*S*)-enantiomer (Scheme 170) [265].

#### 8.2.3. Disaccharide and Oligosaccharide-Based Crown Ethers

**Scheme 171:**
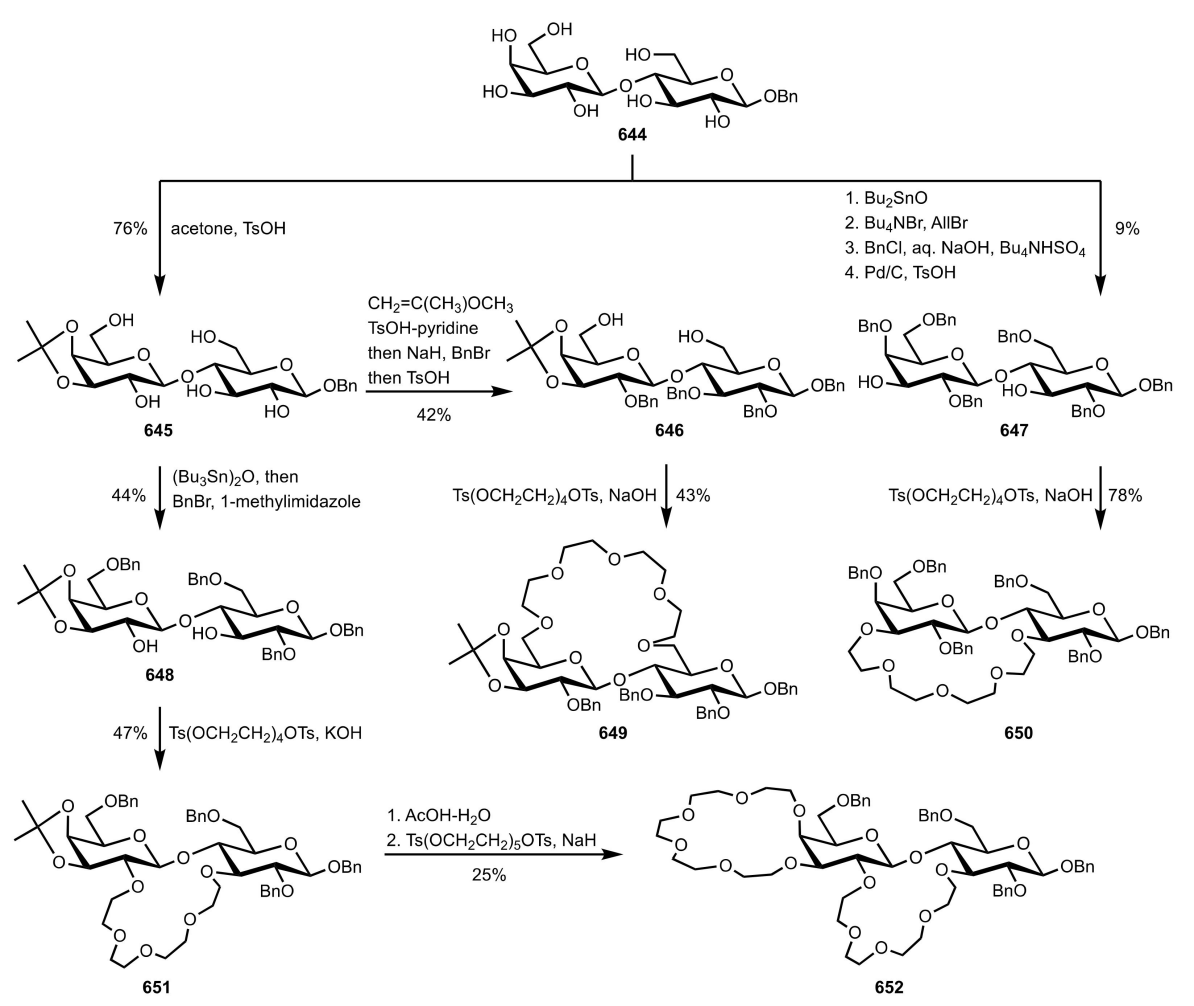
The synthesis of d-lactoside-based crown ethers.

In order to obtain more rigid crown ethers than those synthesised using monosaccharide and alditol derivatives, the research group of Penadés and Martin-Lomas envisaged to introduce a disaccharide into the macrocyclic ethers [267]. Such derivatives were prepared from the commercially available benzyl β-d-lactoside (**644**) (Scheme 171). In order to build the macrocycle between the postions 3 and 2′ of the lactoside unit, **648** was prepared by mono-isopropylidenation of **644** [268] and regioselective benzylation mediated by tributyltin oxide [269].

Reaction with tetraethylene glycol ditosylate in the presence of potassium hydroxide gave **651** in 47% yield [267]. A second 18-crown-6 ether moiety was built onto the lactoside unit of **651** by hydrolysis of the acetonide protecting group and reaction with pentaethylene glycol ditosylate to give **652**. Taking advantage of a one pot procedure involving kinetic acetonation of **645** with 2-methoxypropene, benzylation and mild acid hydrolysis, the 6,6′-dihydroxy derivatives **646** was prepared with a 42% yield and then reacted with tetraethylene glycol ditosylate to afford **649** [270].

Finally, the synthesis of **650** required the preparation of the 3,3′-dihydroxy derivative **647**, which was obtained in a 9% overall yield via stannylation with dibutyltin oxide followed by treatment with allyl bromide, benzylation under phase transfer conditions and, finally, deallylation [267].

**Scheme 172:**
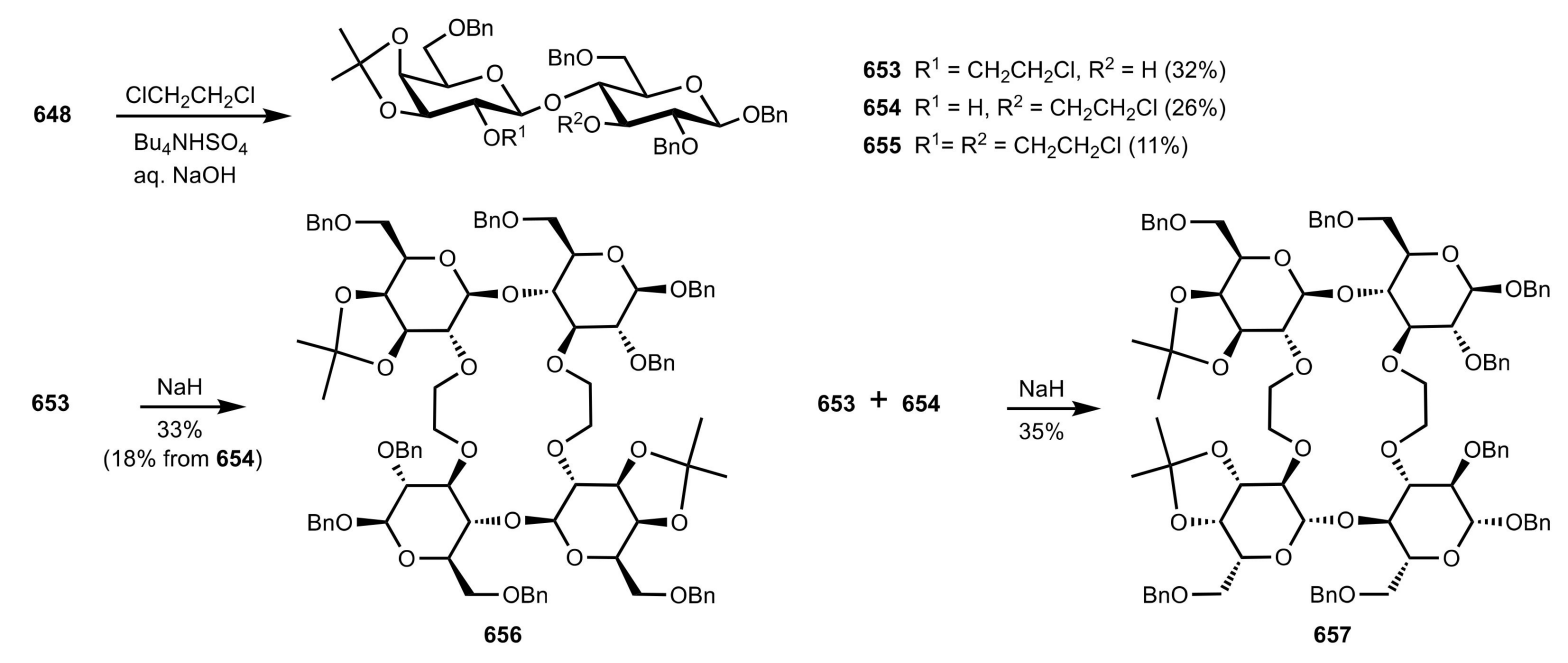
The synthesis of crown ethers containing two d-lactoside units.

The same team reported the synthesis of the *C*_2_-symmetric 18-crown-6 **656** and **657** featuring two lactoside units in the macrocycle. These compounds were both obtained from the mono-alkylated derivatives **653** and **654** by self-condensation of **653** or condensation of **653** and **654** (Scheme 172) [271].

All crown ethers incorporating a lactoside unit, except **652**, catalysed the model Michael addition described in Scheme 166 with some enantioselectivity. The yields varied between 8% and 98% and the enantiomeric excesses between 7% and 70%. The authors demonstrated that, under the chosen conditions, no racemization took place. In addition, the **656** and **657** catalysts predominantly led to the (*R*)-enantiomer, while **649–651** favored the formation of the (*S*)-configured compound [271,272].

**Scheme 173:**
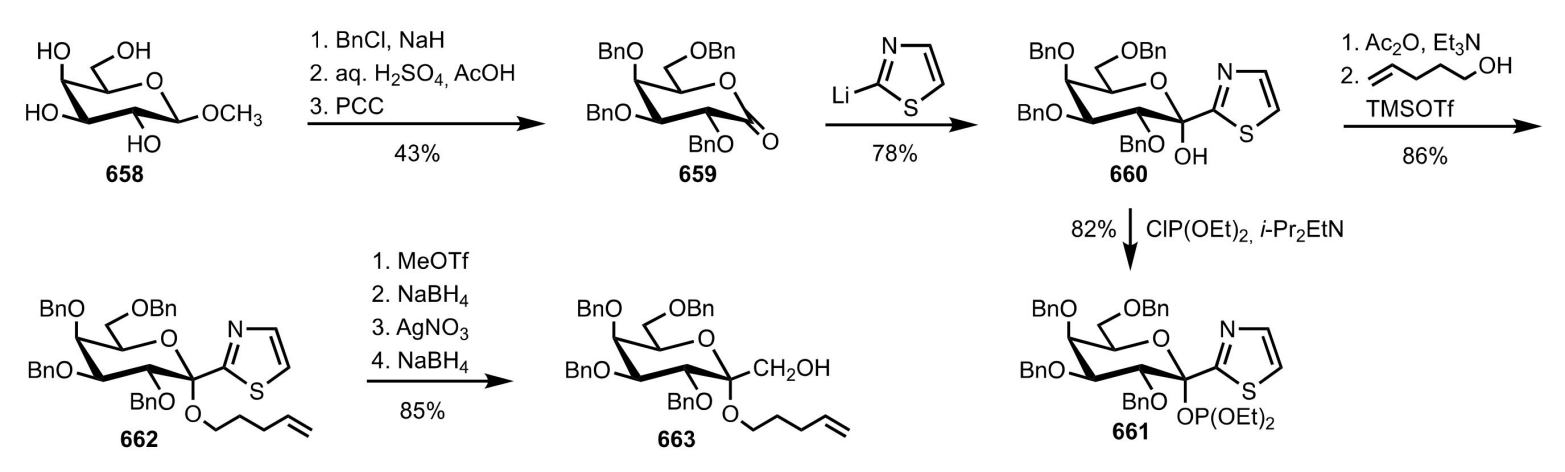
The synthesis of the glycosyl donor and glycosyl acceptor required for the preparation of the di-, tri- and tetrasaccharidic ketosides.

Following a totally different approach, oligoketoside-based crown ethers were prepared by Dondoni and Marra [273] from the di-, tri- and tetrasaccharidic ketosides obtained [274] from the already known [275] d-*galacto*-configured thiazolylketose [276] **660** (Scheme 173). The latter compound was prepared from the d-galactonolactone **659**, in turn, synthesised [275] by oxidation of the corresponding tetra-*O*-benzyl-galactopyranose (obtained by benzylation of methyl β-d-galactoside **658** and acid hydrolysis as described [277]) by the addition at low temperature of 2-lithiothiazole.

The key intermediate **660** was transformed into an efficient glycosyl donor, the anomeric phosphite **661**, as well as into the glycosyl acceptor **663** by acetylation, glycosidation with 4-penten-1-ol to give **662**, with a three-step conversion [278] of the thiazole ring into the formyl group through *N*-methylation, reduction to thiazolidine, silver-assisted hydrolysis, and hydride reduction to primary alcohol (Scheme 173).

**Scheme 174:**
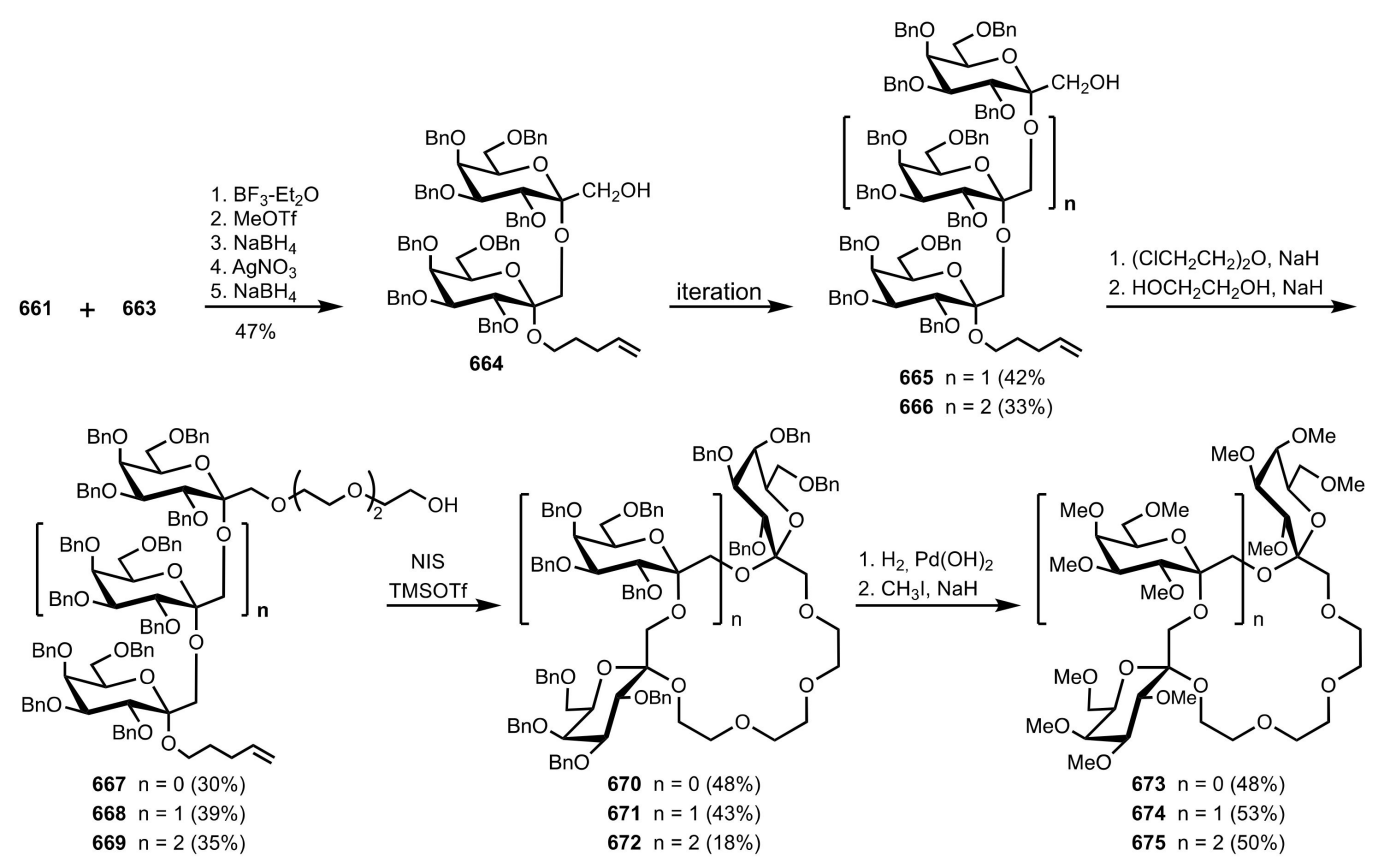
The synthesis of the oligoketoside-based crown ethers.

The coupling of the phosphite **661** with the alcohol **663** in the presence of boron trifluoride afforded the corresponding thiazolyl-disaccharide as pure α-d anomer [274] (Scheme 174). The usual thiazole-to-aldehyde conversion, followed by reduction, led to the disaccharidic alcohol **664**, which was glycosylated with **663** and then transformed into the corresponding trisaccharidic alcohol **665**. Further iteration of the above reaction sequence gave the tetrasaccharide **666**.

To prepare the crown ether derivatives, the chain of **664–666** was elongated to afford **667–669** by reaction with bis(2-chloroethyl) ether and coupling of the resulting alkyl chlorides with ethylene glycol [273]. Upon *N*-iodosuccinimide activation of the anomeric pentenyl group, the alcohols **667–669** gave the benzylated cyclic *O*-glycosides **670–672**. From these compounds, a second set of crown ethers (**673–675**) was obtained by hydrogenolysis and methylation (Scheme 174) [273].

The six crown ethers **670–675** were tested as chiral hosts in the model Michael addition (see Scheme 166) of methyl phenylacetate (two equiv.) to methyl acrylate in the presence of sodium or potassium *t*-butoxide in toluene at −78 °C to afford the adduct **627** in good to high isolated yield (60–94%) although the observed enantiomeric excesses were in general low or moderate (5–65%) [273]. It was found that the benzylated disaccharidic 15-crown-5 ether **670** gave the best results (*ee* = 55% in favour of (*R*)-**627**) when *t*-BuONa was employed, whereas its methylated analogue **673** was well suited for the use together with *t*-BuOK (65% *ee*) leading to the opposite enantiomer. Contrary to expectations, the trisaccharidic 18-crown-6 ethers **671** and **674** were not better hosts for the potassium cation (*ee* = 5% and 45%, respectively).

### 8.3. Sugar Aza-Crown Ethers

**Scheme 175:**
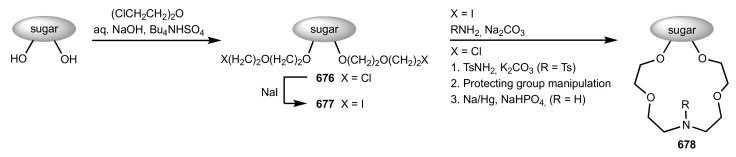
The general synthetic approach to sugar-based aza-crown ethers.

A large variety of monoaza-analogues of crown-ether were prepared, most of them featuring a side chain, which held additional coordinating sites. These structures were named nitrogen-pivot lariat ether by Gokel in reference to the use of a lasso in the American West [279]. The synthesis of this class of chiral crown ethers was based on alditols, glycals or aldoses with two free hydroxyl groups that were alkylated with bis(2-chloroethyl)ether in a liquid–liquid two-phase system (Scheme 175).

The exchange of chlorine to iodine atom was then performed with NaI to afford the bis-iodo derivatives whose reaction with primary amines in the presence of Na_2_CO_3_, gave the aza-crown ethers **678**. In the particular case of the *N*-tosyl or NH derivatives the cyclisation was done from the bis-chloro derivatives using *p*-toluenesulfonamide in the presence of K_2_CO_3_. Treatment with sodium amalgam gave the free NH aza-crowns.

#### 8.3.1. Alditol-Based Aza-Crown Ethers

**Figure 44:**
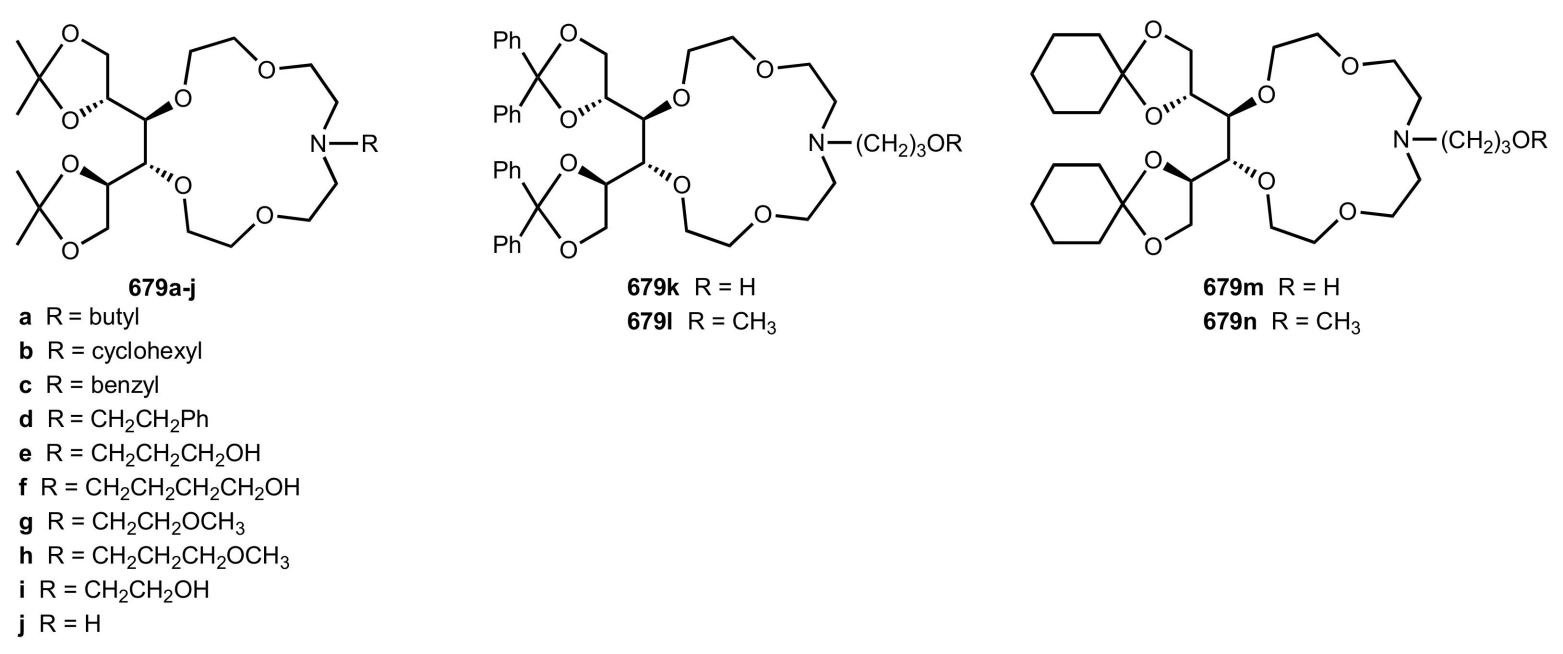
Series of prepared mannitol-based aza-crown ethers.

Following the general strategy described above, the mannitol-based aza-crown ethers **679a-j** [280], **679k,l** and **679m,n** [254] were prepared from 1,2:5,6-di-*O*-isopropylidene-d-mannitol (**593**), 1,2:5,6-di-*O*-diphenylmethylidene-d- mannitol (**599**) [256] and 1,2:5,6-di-*O*-cyclohexylidene-d-mannitol (**598**) [255], respectively (Figure 44).

The di-*O*-isopropylidene-d-mannitol-based aza-crown ethers **679a-j** were used as phase transfer catalysts in the Michael addition of 2-nitropropane to chalcone **602** [280]. Compounds **679a-j** showed a low activity in this reaction conducted under two-phase liquid–liquid conditions since low yields (31–39%) were obtained after 48 h and the asymmetric induction was modest (6–67%). The best result (*ee* = 67% in favor of the (*R*)-enantiomer) was observed with the compound unsubstituted on the nitrogen atom **679j** (38% yield).

Concerning the nitrogen-substituted compounds, the best catalyst was the *N*-3-hydroxypropyl derivative **679e**, which led to the Michael adduct with a 39% yield and 40% enantiomeric excess [280]. The same crown ether was also used to catalyse the addition of nitropropane to other aromatic and heteroaromatic chalcone analogues, but the results were also modest and, in all cases, worse than those obtained with the d-glucose-based aza-crown ethers (see Michael additions in Section 8.3.3.2) [281]. Likewise, the crown ethers **679e** and **679i** (whose synthesis and characterization were not described) gave poorer results than their analogues derived from d-glucose in the epoxidation reaction of chalcones with *tert*-butylhydroperoxide (see Epoxidation of enones in Section 8.3.3.2) [282].

The aza-crown ethers **679k,l** and **679m,n** were used as catalysts in asymmetric Michael additions and in a Darzens condensation [254]. While the addition of diethyl acetoxymalonate to *trans*-chalcone **602** required 10 days and gave **603** with a 24–39% yield and 11–38% enantiomeric excess, the Michael addition of diethyl acetamidomalonate to β-nitrostyrene (**604**) required 24–48 h and led to **605** with a 57–81% yield and 14–65% *ee*, the best asymmetric induction (*ee* = 65%) being obtained with the organocatalyst **679l**. The di-*O*-diphenylmethylidene protected aza-crowns **679k** and **679l** afforded the best results in the Michael-initiated ring closure reaction of diethyl bromomalonate with benzylidenemalonitriles. For instance, the cyclopropane **607** was obtained with 85% and 84% enantiomeric excess using catalysts **679k** or **679l**, respectively.

#### 8.3.2. Glycal-Based Aza-Crown Ethers

**Scheme 176:**
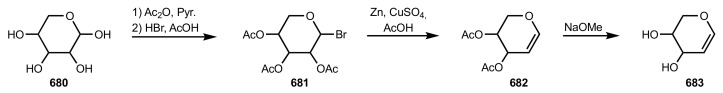
The general synthetic approach to deprotected glycals.

**Figure 45:**
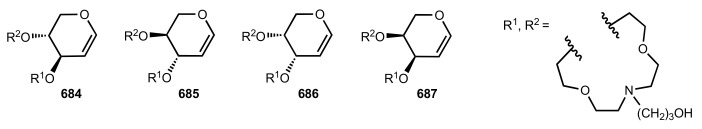
Glycal-based aza-crown ethers prepared by Bakó and co-workers.

Taking advantage of their extensive experience in the preparation of crown ethers and aza analogues, Bakó and co-workers prepared monoaza-15-crown-5 ethers using glycals obtained from l- and d-xylose and l- and d-arabinose [283]. The glycals **683** were obtained by conventional methods involving the preparation of the peracetylated glycosyl bromides **681**, which, when treated with activated zinc in acetic acid, gave rise to an elimination reaction (Scheme 176). After deprotection, the diols **683** were used as starting material to prepare the aza-crown ethers **684–687** (Figure 45) according to the general method described in Scheme 175.

**Scheme 177:**
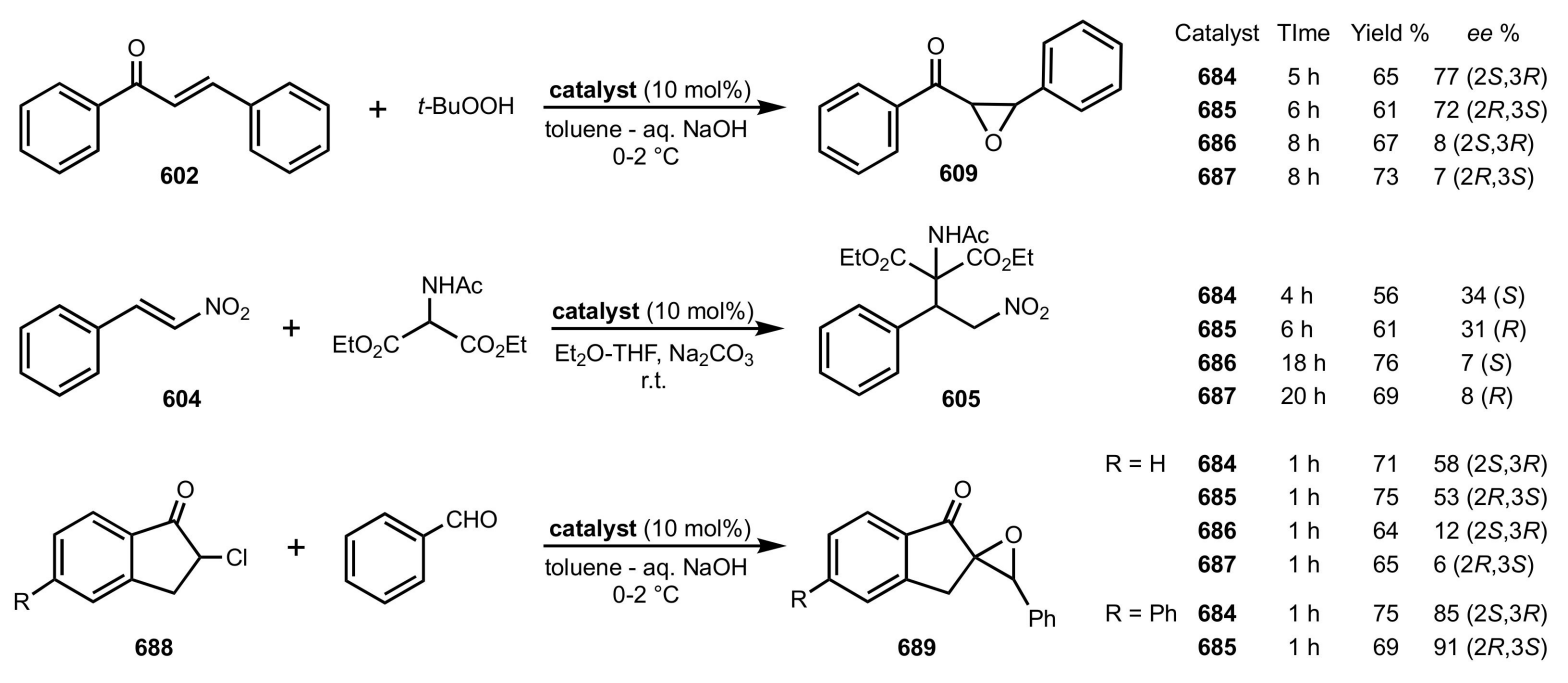
Epoxidations, Michael additions and Darzens reactions catalysed by **684–687**.

The two enantiomeric pairs of lariat ethers were tested as phase transfer catalysts in liquid–liquid and solid–liquid systems for epoxidation, Michael or Darzens reactions [283] (Scheme 177). The epoxidation reaction of the chalcone **602** carried out with *tert*-butylhydroperoxide in a liquid–liquid two phases system gave exclusively the *trans*-epoxyketone **609**. Using the d-xylal-based catalyst **684**, the (2*S*,3*R*)-configured epoxyketone was obtained (77% *ee*), whereas the l-xylal-based macrocycle afforded the (2*R*,3*S*) enantiomer (72% *ee*). The d- or l-arabinal-based crown ethers **686** or **687** gave poor enantioselectivities (*ee* = 8 and 7%); however, in this case as well, the selectivity was reversed.

The same trend was observed for the Michael reaction between diethyl acetamidomalonate and β-nitrostyrene **604,** which took place with modest enantiomeric excesses. On the other hand, in the Darzens reaction involving α-chloroketones **688** and benzaldehyde, enantiomeric excesses of 85 (2*S*,3*R*) and 91% (2*R*,3*S*) were observed for the *trans*-epoxyketones **689** obtained from the phenyl-substituted 2-chloroindanone **688** (R = Ph) using the d- or l-xylal-based catalyst **684** or **685**, respectively. Even if some enantiomeric excesses were modest, this work is interesting because it demonstrated that the configuration of the sugar unit annulated to the crown ether determined the stereochemical preference of the reaction.

#### 8.3.3. Aldose-Based Aza-Crown Ethers

##### 8.3.3.1. Synthesis

**Scheme 178:**
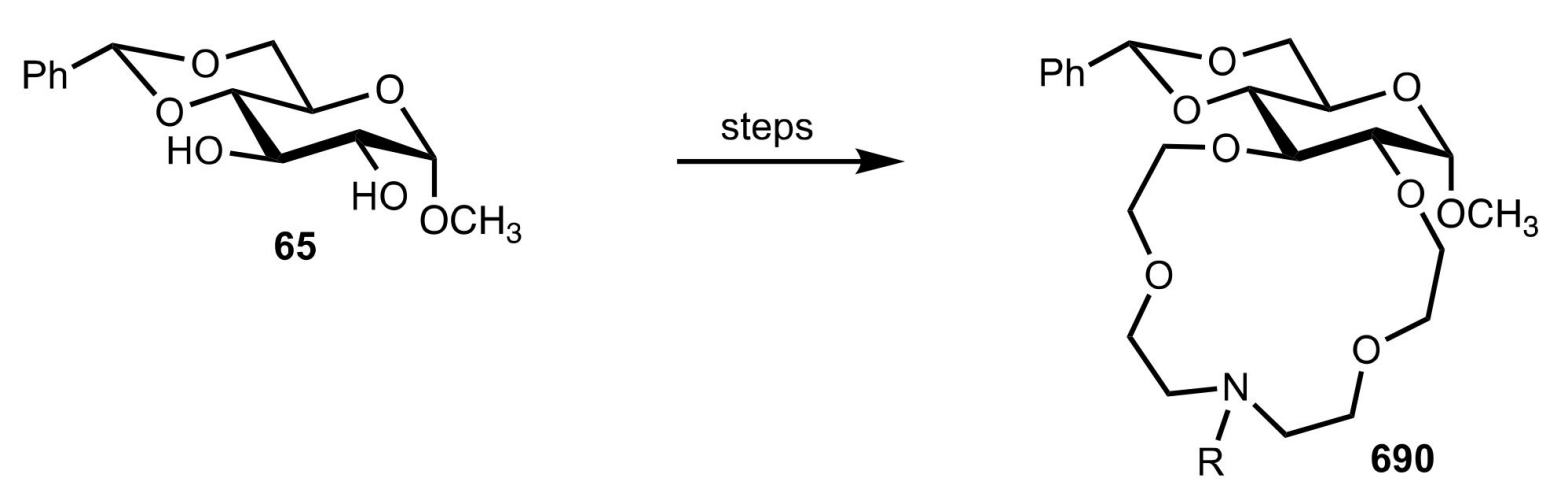
The general synthesis of glucose-based aza-crown ethers (for R, see Table 2).

In most of the structures of the numerous aldose-based aza-crown ethers described, the polyether chain is connected to the C-2 and C-3 atoms of variously protected α- or β-glycosides. These aza-crown ethers were prepared through the general method outlined in Scheme 175, i.e., reaction of the sugar diol with two (2-iodoethyl)ether chains followed by coupling with primary amines. Starting from the 4,6-*O*-benzylidene derivative **65** (Scheme 178), a large number of lariat ethers **690**, variously functionalized on the nitrogen atom, were prepared this way (Table 2).

**Scheme 179:**
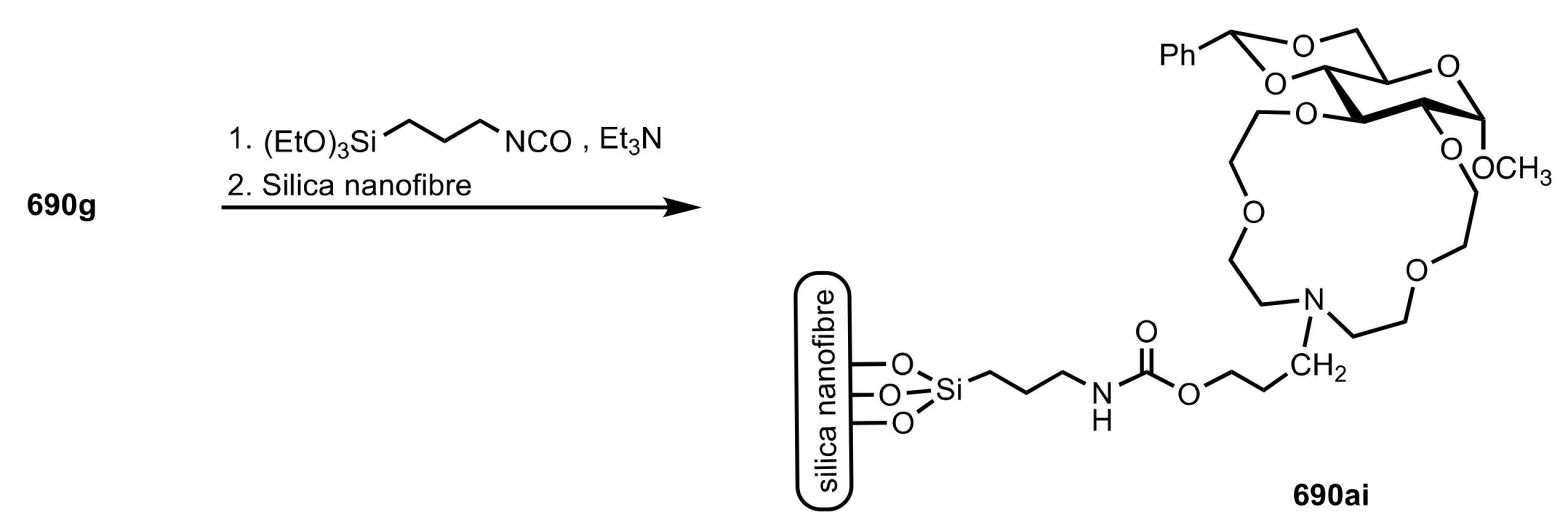
The synthesis of the supported glucose-based aza-15-crown-5 ether.

In order to facilitate the recycling of the catalyst, the supported glucose-based aza-15-crown-5 ether **690ai** was prepared from **690g** by reaction with 3-(triethoxysilyl)propyl isocyanate and then silica nanofibre [292] (Scheme 179).

**Figure 46:**
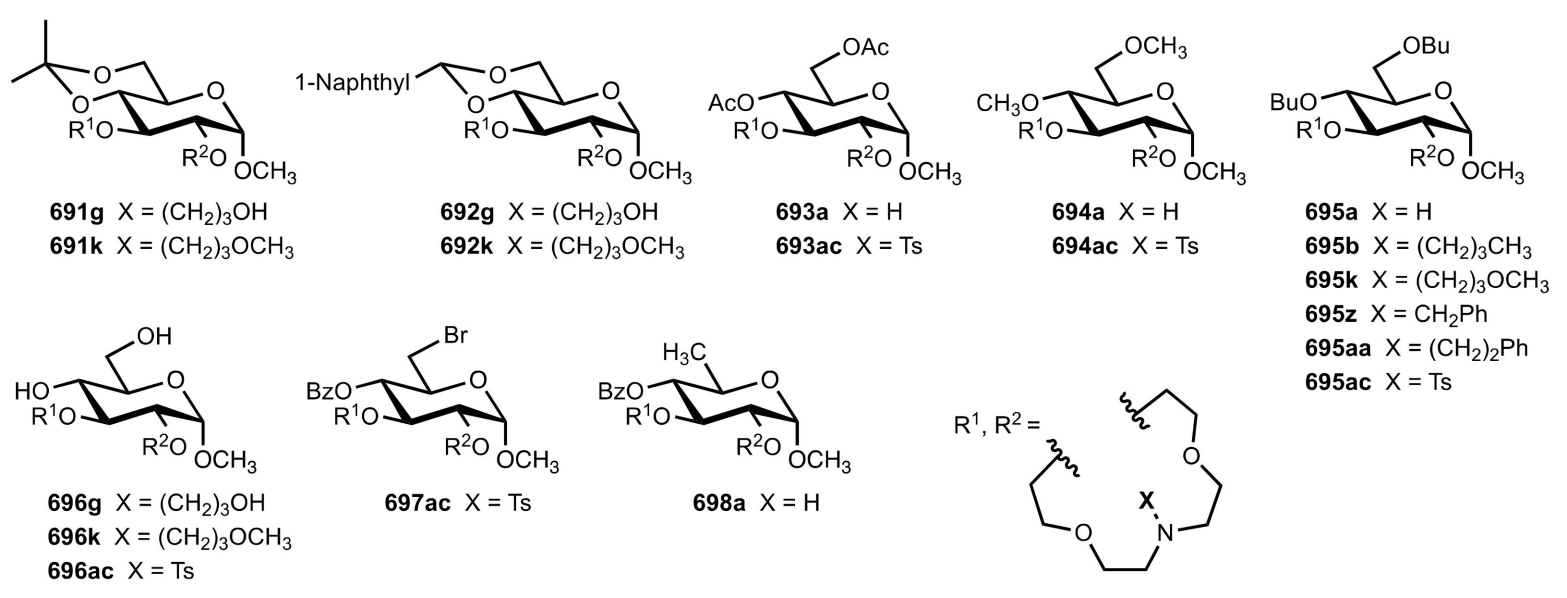
Newly synthesised aza-crown ethers bearing a methyl α-d-glucoside moiety.

Other aza-crown ethers incorporating a methyl α-d-glucoside unit were prepared from suitably protected glucosides (Figure 46). Using the methodogy outlined in Scheme 175, **691g,k** and **692g,k** were obtained from methyl 4,6-*O*-isopropylidene-α-d-glucopyranoside or methyl 4,6-*O*-(1-naphthyl)methylene-α-d-glucopyranoside [293]. **696g,k** were obtained by hydrogenation of **690g,k** [293], while the *N*-tosyl derivative **696ac** was prepared by acid hydrolysis of **690ac** [284].

The latter compound was also treated with NBS in the presence of BaCO_3_ to afford **697ac**, which, upon treatment with sodium amalgam, gave **698a**. The methylated, butylated and acetylated *N*-tosyl derivatives **694ac**, **695ac** and **695ac** were obtained by alkylation or acylation of **696ac** and the cleavage of the tosyl group led to **693a**, **695a** and **694a** [284]. Alternatively, the butylated compound **695a** was also prepared from methyl 4,6-di-*O*-butyl-α-d-glucopyranoside [294] through the general method developped by Bakó and co-workers (Scheme 175). This approach made it possible to synthesize also the macrocycles **695b,k,z,aa** [294].

**Scheme 180:**
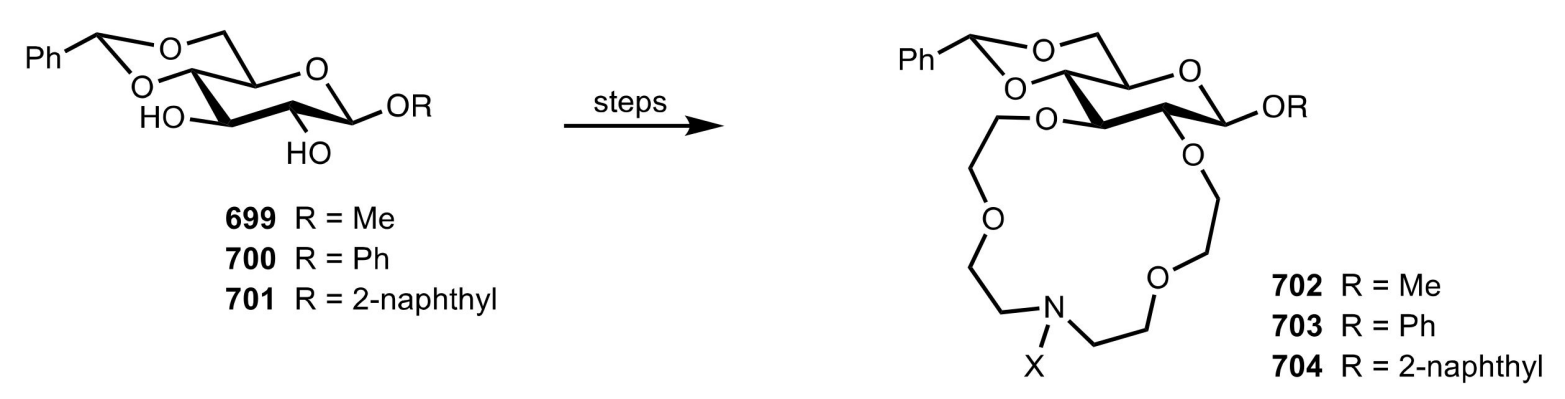
The general synthesis of aza-crown ethers bearing a β-d-glucoside moiety (for X, see Table 3).

**Scheme 181:**
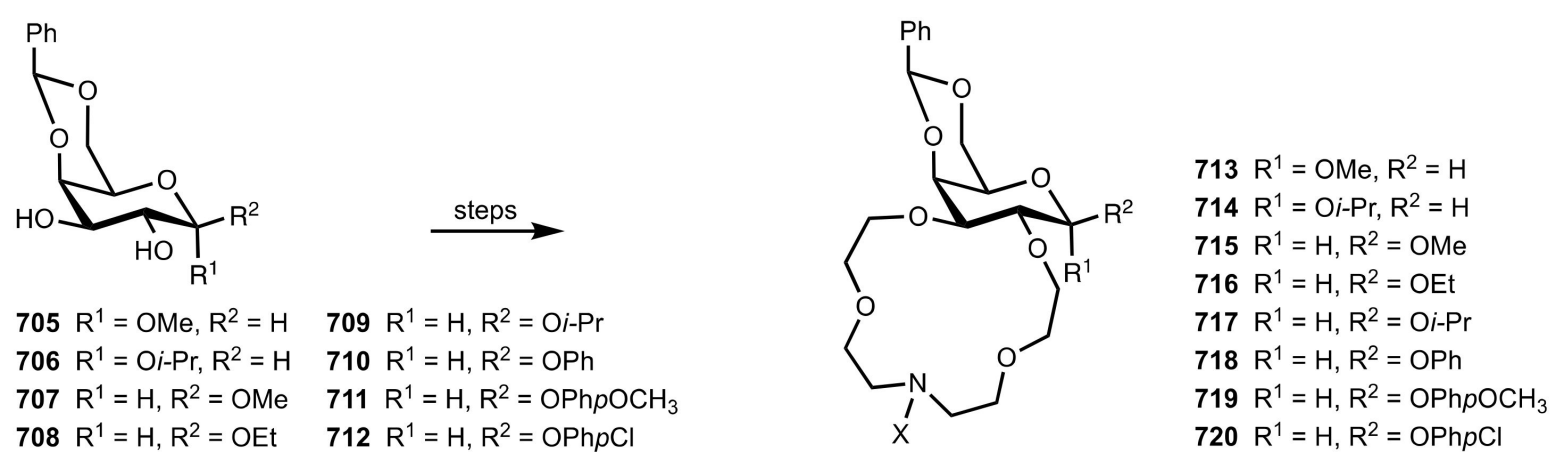
The general synthesis of aza-crown ethers bearing a d-galactoside moiety (for X, see Table 4).

Following the general method described in Scheme 175, many other aza-crown ethers were prepared starting from the β-d-glucosides **699** [295], **700** [296] and **701** [297] (Scheme 180, Table 3) and from α- or β-d-galactosides (Scheme 181, Table 4).

**Figure 47:**
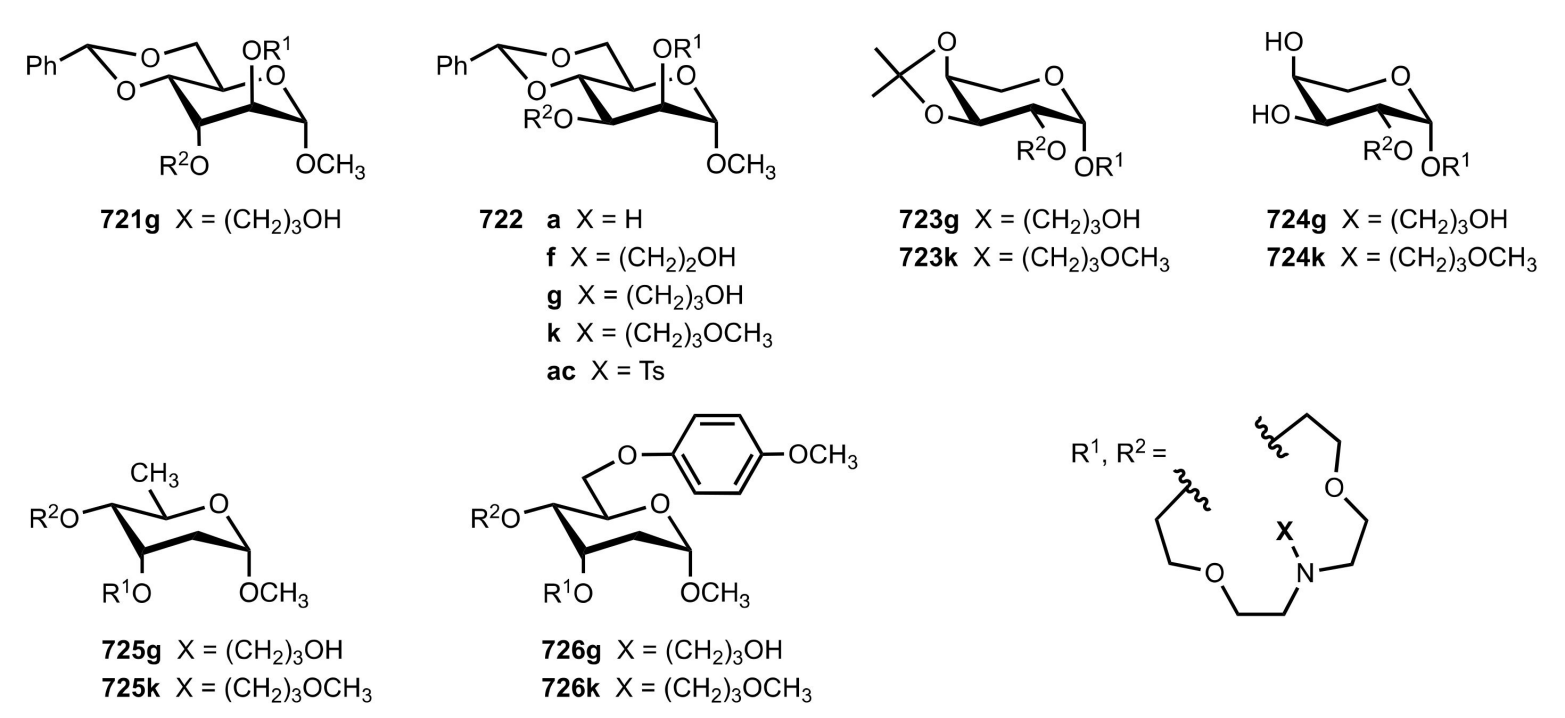
Aza-crown ether prepared by Bakó and co-workers from various glycosides.

Other aza-crown ethers were prepared from d-altrose (**721**) [304], d-mannose (**722**) [305], l-arabinose (**723** and **724**) [306], 2,6-dideoxy-d-*ribo*-hexopyranose (**725**) and 2-deoxy-d-*ribo*-hexopyranose (**726**) derivatives [307] (Figure 47). The sugar 3,4-diols required for the synthesis of **725** and **726** were both prepared from the epoxide **259** (see Scheme 155) through a four-step and two-step reaction sequence, respectively [307].

**Scheme 182:**
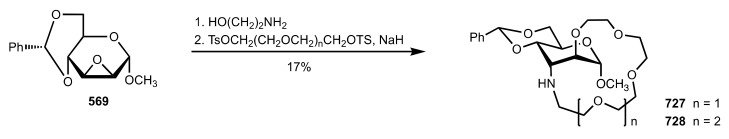
The synthesis of the altrose-based crown-amines.

Another series of aza-crown ethers, featuring the nitrogen atom directly linked to the sugar unit, was prepared by regioselective epoxide opening of **569** with ethanolamine followed by cyclisation with tri- or tetraethylene glycol ditosylate to afford the altrose-based crown-amines **727** and **728** (Scheme 182) [307].

**Figure 48:**
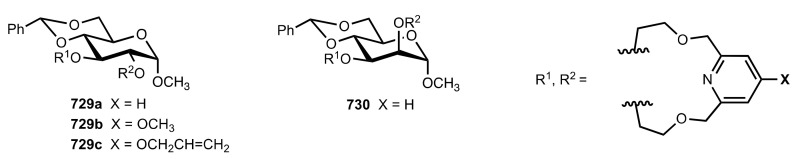
Aza-crown ether containing a pyridine ring prepared by Bakó and co-workers.

Bakó and co-workers described another class of aza-crown ether featuring a pyridine ring in the macrocycle backbone in order to have a more rigid structure (Figure 48). The synthesis of **729** and **730** was conducted starting from methyl 4,6-*O*-benzylidene-α-d-glucopyranoside or 4,6-*O*-benzylidene-α-d-mannopyranoside, respectively, and 2,6-pyridine-dimethyl ditosylates [308].

##### 8.3.3.2. Catalysis

Having prepared a multitude of aza-crown ethers incorporating various monosaccharides and chains on the nitrogen atom, Bakó and co-workers published, in 2010, a review in which they gathered general conclusions about the effectiveness of these macrocycles on asymmetric catalysis for Michael reactions, epoxidation of enones or Darzens condensations [6]. Their conclusions are collated below and supplemented with more recent work.

Michael additions

**Scheme 183:**
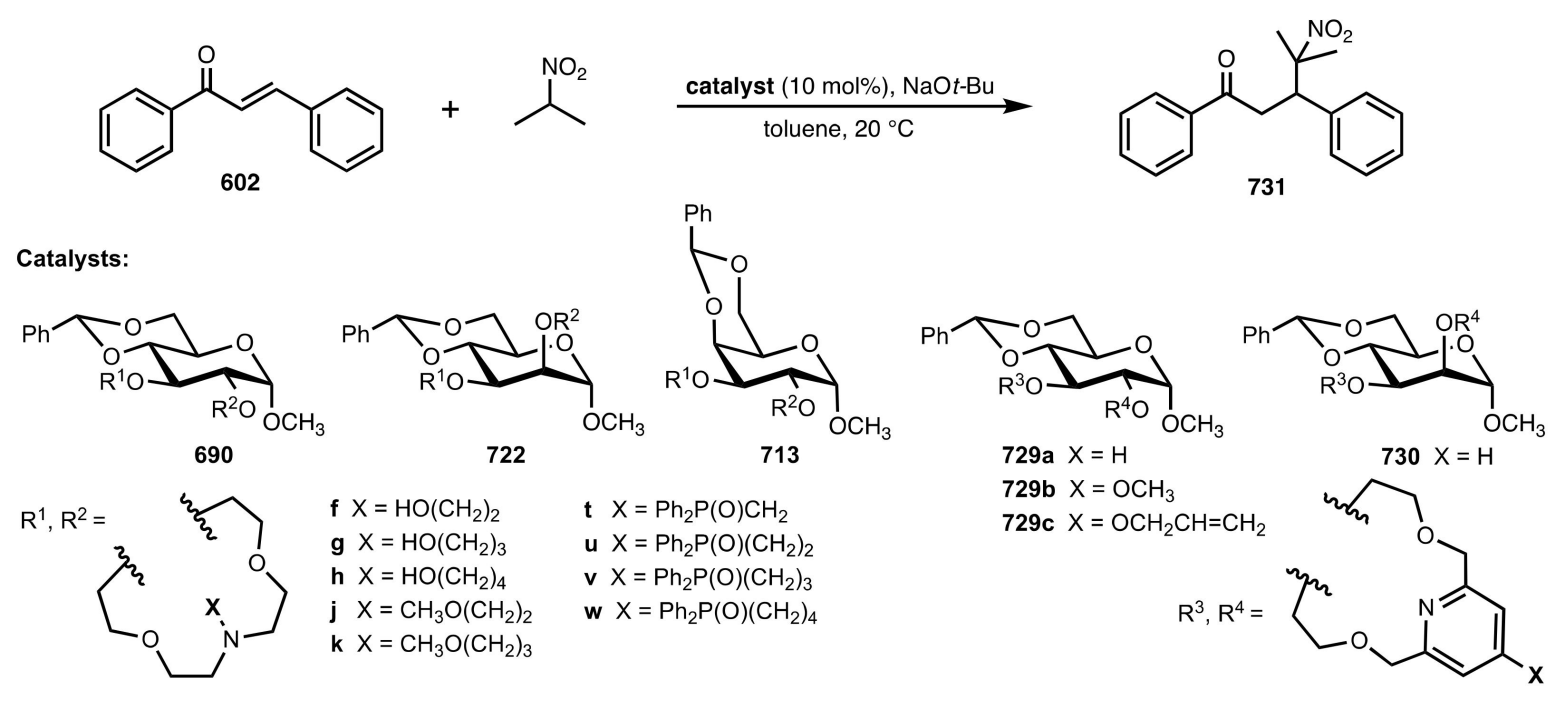
The Michael addition catalysed by glucoside-, mannoside- and galactoside-based aza-crown ethers.

The most studied Michael reaction was the addition of 2-nitropropane to chalcone **602**. The reaction was performed in a two-phase solid–liquid system using toluene as solvent in the presence of sodium *tert*-butoxide at room temperature (Scheme 183). As the catalyst, the above-described crown ethers containing α-d-glucoside (**690–698**, **729**), [281,284,285,286,288,289,293,294,308,309,310,311,312,313] β-d-glucoside (**703**) [298], α-d-mannoside (**722**, **730**) [305,308,309] or α-d-galactoside (**713**) [286] moieties were employed.

Amongst these, the catalysts displaying a methyl α-glycoside protected by a benzylidene group in positions 4 and 6, conferring a certain rigidity to the monosaccharide, were the most efficient. Some results are given in Table 5. The comparison of the various monosaccharides incorporated in the aza-crown ethers allows to conclude that, concerning the enantioselectivity, the most effective is d-glucose followed by d-mannose, d-galactose and d-altrose. d-Glucopyranoside- and d-galactopyranoside-based macrocycles mainly gave the (*R*)-enantiomer while the mannoside-based aza-crown ethers led to the (*S*)-enantiomer (see the Table 5 footnote).

The highest enantioselectivities were obtained with catalysts whose chain on the nitrogen atom ends with hydroxy or methoxy functions or is a phosphinoxidoalkyl chain. The length of the chain connecting the nitrogen atom to the terminal function had a great influence on the enantioselectivity. A three-carbon atom spacer proved to be the best choice for the chain ending with an alcohol function (Table 5, entries 1, 2, 8, 9 and 10), whereas the four-carbon atom chain connecting the phosphine oxide function proved to be optimal (Table 5, entries 4–7).

**Scheme 184:**
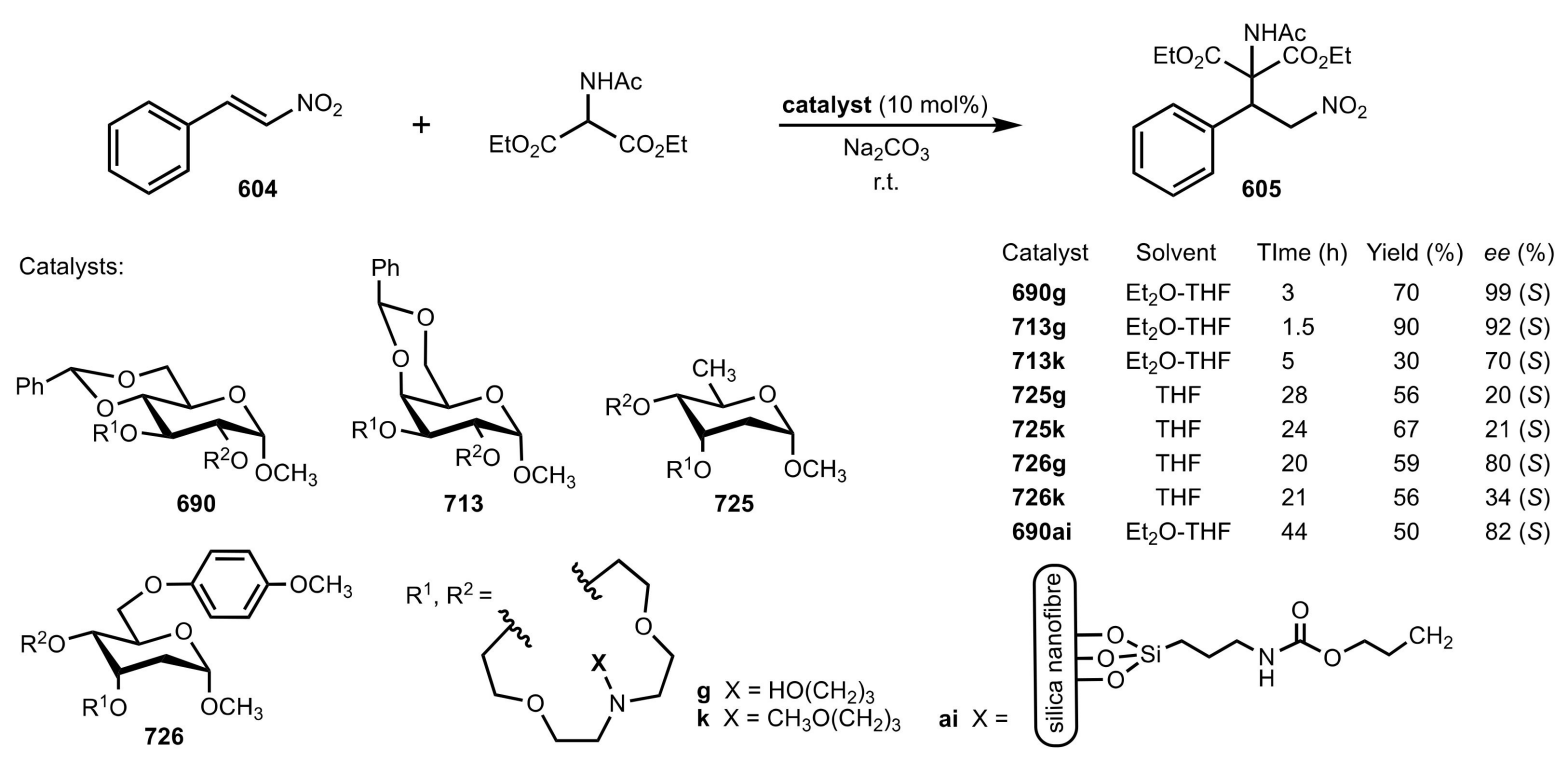
Other Michael additions catalysed by sugar-based aza-crown ethers.

Other Michael reactions, such as the addition of malonates to *trans*-nitrostyrene **604** [289,292,302,304,311,312,314] or *trans*-chalcones [299], were conducted. For the addition of diethyl acetamidomalonate (Scheme 184), a high enantiomeric excess (99%) was observed using the glucopyranoside-based crown ether **690g** [311], while the galactose derivative **713k** or the 2-deoxy-d-ribose-based aza-crown ethers **725** and **726** gave poorer results [303,307]. This reaction was also performed in the presence of the silica nanofiber supported catalyst **690ai** [292] giving rise to (*S*)-**605** with 82% *ee* after 44 h. The authors claimed that this catalyst could be recovered by filtration and reused without regeneration, but no experimental data were given.

The addition of glycine esters to various Michael acceptors was also achieved with more or less success [290,300,301].

Cyclopropanation

The asymmetric Michael-initiated ring closure (MIRC) reaction has also been extensively studied by Bakó and co-workers. They developed this reaction of diethyl bromomalonate with chalcone (**602**), 2-arylidene-malononitriles (**733**), 2-benzylidene- 1,3-diphenyl-1,3-propanediones (**735**) [303,315,316], 2-arylidene-1,3-indandiones (**737**) [316] and α-cyano-vinylsulfones (**739**) [297] under solid–liquid phase transfer catalytic conditions.

**Scheme 185:**
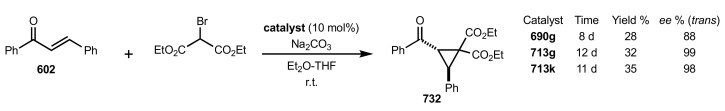
The Michael-initiated ring closure reaction catalysed by sugar-based aza-crown ethers.

The reaction of bromomalonate with **602** was conducted employing the crown ethers incorporating an α-d-galactoside unit (**713g** and **713k**) [303] or a methyl α-d-glucopyranoside unit **690g** (Scheme 185) [315,316]. The reaction times were long (8 to 12 days) and the yields were moderate due to the formation of a few by-products. The *trans* isomer of the cyclopropane derivatives **732** was obtained with high diastereoselectivity (up to 98%). Enantiomeric excess of 98% or 99% using galactose-based macrocycles (**713g** and **713k**) were obtained, whereas the crown ether incorporating a glucose unit **690g** was less efficient with regard to enantioselectivity (*ee* = 88%). Other chalcones, substituted on the phenyl groups, gave poorer enantioselectivities.

**Scheme 186:**
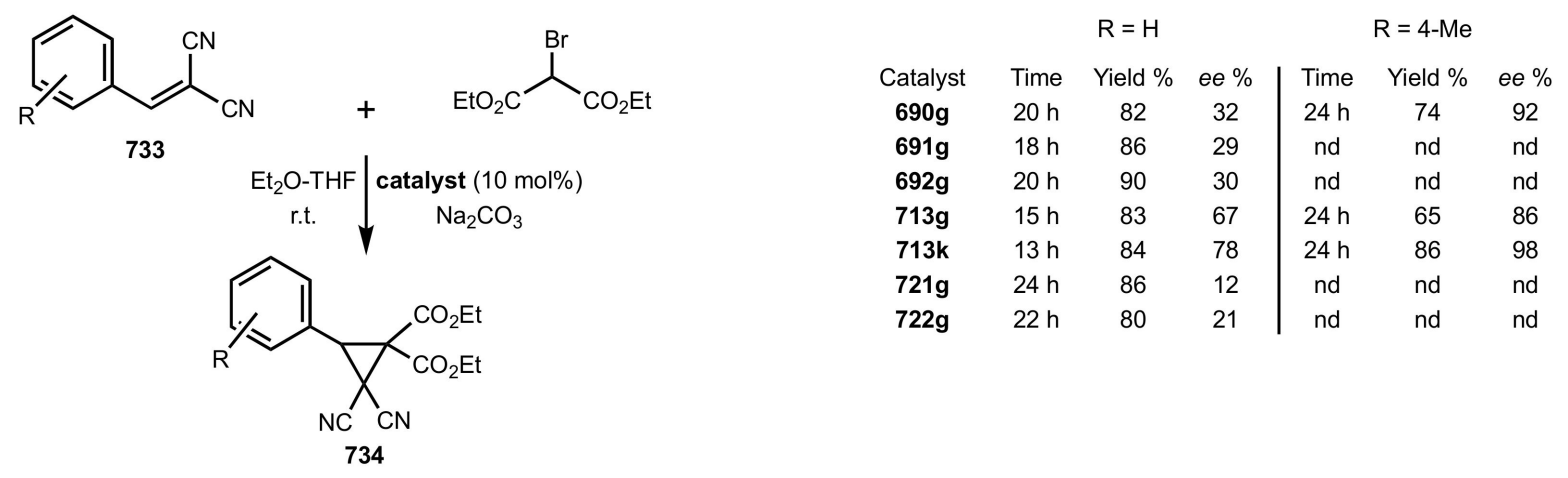
Cyclopropanation catalysed by sugar-based aza-crown ethers (nd: not determined).

Cyclopropanation of 2-benzylidene-malononitriles **733** with diethyl 2-bromomalonate was also investigated using crown ethers incorporating a glucose (**690g**, **691g** and **692g**) [315,316], a mannose (**722g**), an altrose (**721g**) [316] or a galactoside unit (**713g** and **713k**) [303] as catalysts. For the unsubstituted derivative (Scheme 186, R = H), the best catalysts were the galactose-based lariat ethers **713g** and **713k** that gave enantioselectivities of 67 and 78%, respectively, whereas the glucose-based crown ethers **690g**, **691g** and **692g** afforded **734** with enantiomeric excesses of 32%, 29% and 30%, respectively.

These results indicated that the 4,6-*O*-protecting group (benzylidene, isopropylidene or (1-naphthyl)methylene) had no significant impact on the asymmetric induction. The authors also showed that the results vary greatly with the substitution of the 2-benzylidene-malononitriles (Scheme 186).

**Scheme 187:**
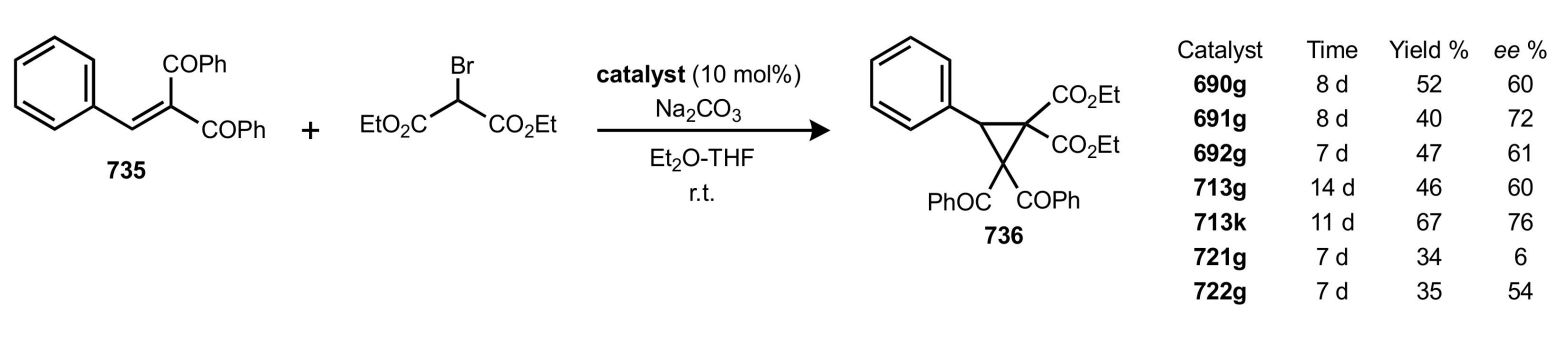
Cyclopropanation catalysed by sugar-based aza-crown ethers.

The same catalysts were used for the MIRC reaction between diethyl bromomalonate and 2-benzylidene-1,3-diphenyl-1,3-propanediones **735** (Scheme 187) [303,315,316]. The cyclopropane derivative **736** was isolated in low to medium yields (34–67%). The highest enantioselectivity (76%) was obtained when the galactose-based catalyst **713k** was used.

**Scheme 188:**
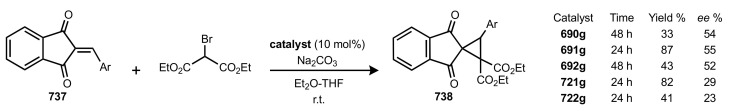
Cyclopropanation of 2-arylidene-1,3-indandiones catalysed by aza-crown ethers.

The reaction of diethyl bromomalonate with 2-arylidene-1,3-indandiones **737** was also investigated in the presence of d-glucose, d-mannose- and d-altrose-based catalysts (Scheme 188) [316]. However, the cyclopropane derivatives **738** were obtained with moderate to low enantioselectivity.

**Scheme 189:**
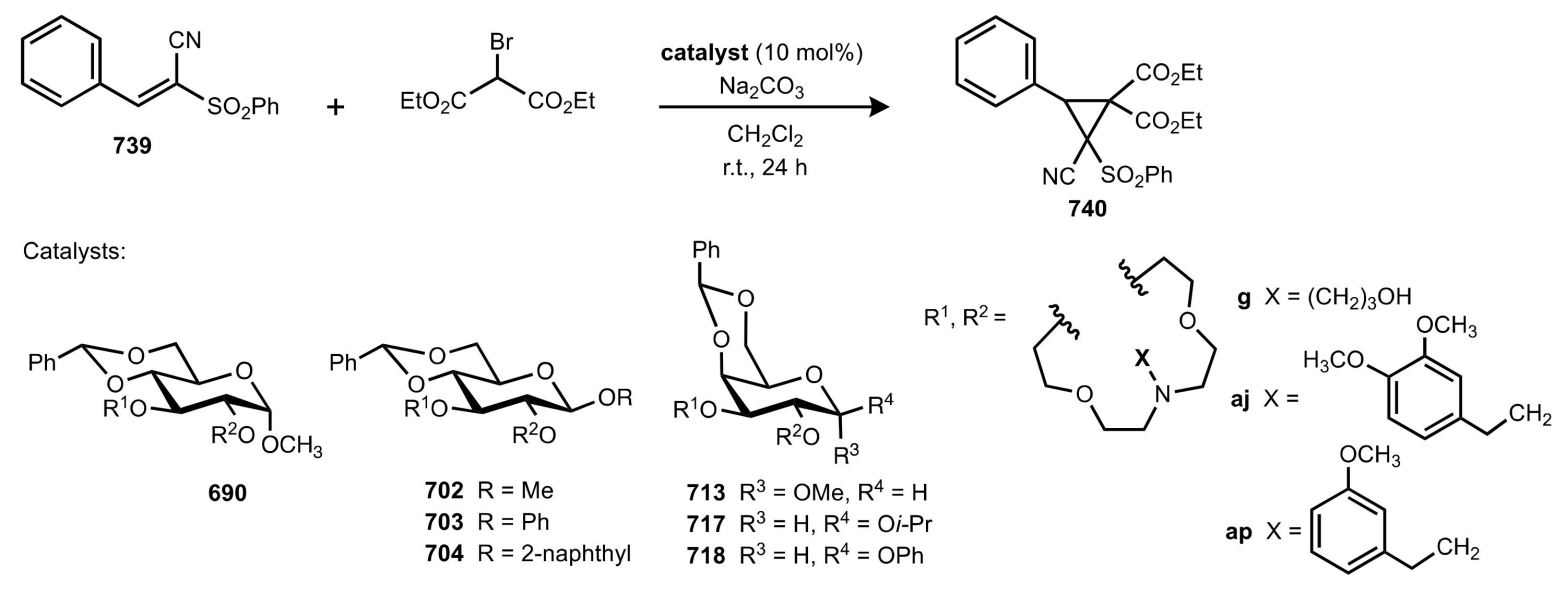
The Michael-initiated ring closure reaction catalysed by sugar-based aza-crown ethers.

In order to make maximum use of their phase transfer catalysts, Bakó and co-workers also studied the cyclopropanation reaction of α-cyano-vinylsulfones [297]. 4,6-*O*-Benzylidene-d-glucopyranoside- and d-galactopyranoside-based macrocycles were tested for the reaction of (*E*)-3-phenyl-2-(phenylsulfonyl)acrylonitrile (**739**) with diethyl bromomalonate (Scheme 189, Table 6). The *trans* cyclopropane **740** (i.e., bearing the phenyl and phenylsulfonyl groups on the opposite side of the ring) was obtained with enantiomeric excesses between 18 and 80%.

In general, macrocycles featuring a 2-(3,4-dimethoxyphenyl)ethyl (Table 6, entries 2, 3, 5, 7) or 2-(2-methoxyphenyl)ethyl side chain (Table 6, entry 8) led to higher *ee* values than those bearing an hydroxypropyl side arm (Table 6, entries 4, 6). Likewise, with regard to enantioselectivity, the d-galactose-based crown ethers are slightly more efficient than the d-glucose-based ones.

Other cyclopropane derivatives synthesised from α,β-unsaturated cyanosulfones containing substituted phenyl, naphthyl, pyridyl, furyl and thienyl groups were obtained in good yields and enantioselectivities up to 85%.

Epoxidation of enones

A large number of sugar-based crown ethers were tested for their efficacy in the asymmetric epoxidation of enones. These included methyl α-d-glucopyranosides whose positions 4 and 6 were either protected by a benzylidene (**690**) [282,289,317], isopropylidene (**691**) and (1-naphthyl)methylene group (**692**) or free (**696**) [293], the phenyl 4,6-*O*-benzylidene-β-d-glucopyranoside **703**, two 4,6-*O*-benzylidene-α-d-galactopyranosides (**713**, **714**) [282,302], several 4,6-*O*-benzylidene-β-d-galactopyranosides (**715–720**) [302], the methyl 4,6-*O*-benzylidene-α-d-altropyranoside **721g** [304] and the methyl 4,6-*O*-benzylidene-α-d-mannopyranosides **722** [305,317].

**Scheme 190:**
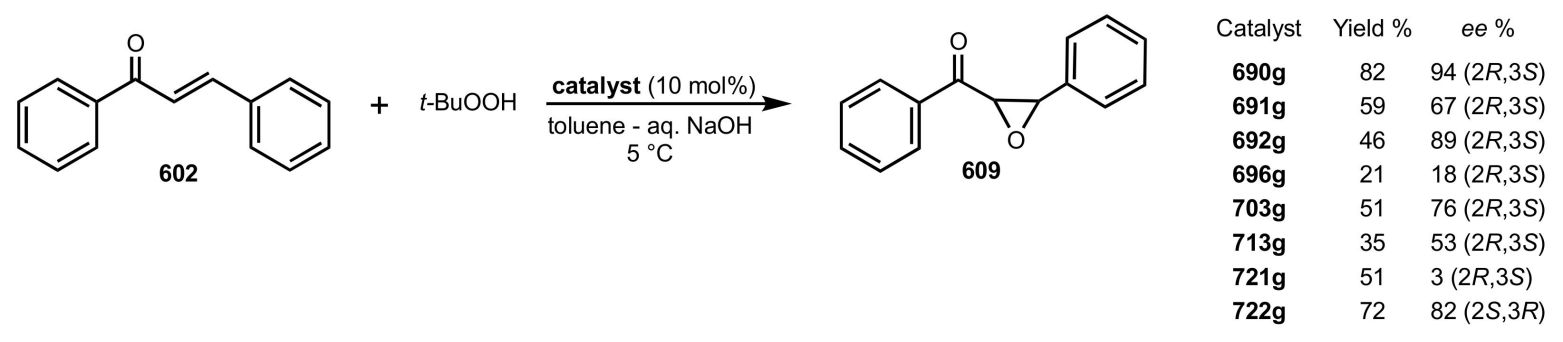
The epoxidation of chalcone catalysed by sugar-based aza-crown ethers functionalised with hydroxypropyl side arm.

The influence of the chain linked to the nitrogen atom of the carbohydrate-based lariat ethers on the performance of these catalysts was also studied. It appeared that the most efficient catalysts for the epoxidation of *trans*-chalcone **602** using *tert*-butylhydroperoxide were those with a hydroxypropyl side arm (Scheme 190). All the macrocycles featuring a d-glucose unit (**690**, **691**, **692**, **696** and **703**) led mainly to the (2*R*,3*S*)-configurated enantiomer except **690a**, which provided the (2*S*,3*R*)-epoxide with an enantiomeric excess of 28%.

This enantiomer was also obtained as major product in the presence of the d-mannose-based catalysts **722g,** while the d-altrose-based crown ether **721g** gave no asymmetric induction (*ee* = 3%). In order to provide an explanation to these results, a theoretical study using molecular modeling and density functional theory (DFT) calculations was performed [304].

The aza-crown ethers **729** and **730**, containing a pyridine ring, were also used as phase transfer catalysts for this reaction [308]. The chiral macrocycles incorporating a glucopyranoside unit **729a-c** promoted the formation of the (2*R*,3*S*)-epoxide **609** with modest enantioselectivities (*ee* = 25–54%) and yields (36–40%), whereas the use of the mannopyranoside-based crown ether **730** gave rise to the opposite enantiomer in 39% yield and 47% enantiomeric excess.

Darzens condensation

**Scheme 191:**
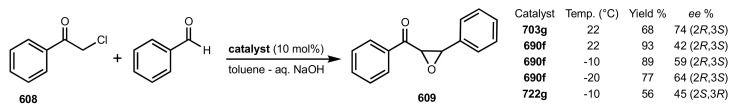
Darzens condensation catalysed by aldose-based aza-crown ethers.

The Darzens condensation is another well-known method for the synthesis of epoxy ketones. Bakó and co-workers conducted this reaction starting from 2-chloroacetophenone (**608**) and benzaldehyde under liquid–liquid reaction conditions in the presence of a large number of aldose-based aza-crown ethers (Scheme 191) [286,289,293,298,299,302,305,313]. In all cases, the *trans*-epoxide **609** was formed with complete diastereoselectivity, whereas the best enantioselectivity (*ee* = 74%) was obtained using the lariat ether anellated to the 4,6-*O*-benzylidene-β-d-glucopyranoside **703g** at room temperature [298,299].

In order to improve the enantioselectivity, other reactions were performed at lower temperature employing the catalysts based on methyl 4,6-*O*-benzylidene-α-d-glucopyranoside **690f** and **690g [286,313]**. Thus, when **690f** was the catalyst, the enantiomeric excess increased from 42% at 22 °C to 59% at −10 °C and 64% at −20 °C in favor of the (2*R*,3*S*)-isomer. As in the case of the epoxidation reaction (see Epoxidation of enones in Section 8.3.3.2), the opposite enantiomer was enantioselectively obtained in the presence of **722g** [305]. The same team extended the application of their organocatalysts to other aromatic or heteroaromatic α-chloroacetyl derivatives [318,319,320].

## 9. Conclusions

The chemical and stereochemical behaviours of hundreds of carbohydrate-based organocatalysts, including a few unmodified monosaccharides and oligosaccharides, have been studied over the last three decades. In particular, the interest of researchers has been centred on the synthesis and application of sugar thioureas, sugar ketones and sugar-based (aza)crown-ethers. Most organocatalysts have been employed to perform classical organic transformations, such as aldol reaction, the Mannich reaction and Diels–Alder cycloaddition.

Although these old reactions are still very useful and can benefit from chiral organocatalysts, it is fair to state that almost all the enantioselective reactions outlined in the present review were performed using extremely simple substrates. Moreover, in the vast majority of cases, the catalyst loading was rather high (10–20 mol%) and the reaction solvents were chosen exclusively on the basis of the chemical and stereochemical optimization of the catalysed reaction—the “green” characteristics of the solvent not being taken into consideration.

It can be also mentioned that the recycling of these quite complex catalysts was not always described in the published articles. Therefore, despite the huge effort required for their synthesis, the actual potential of highly functionalized sugar-based organocatalysts remains largely unexplored. In our opinion, it may be interesting to also exploit these catalysts in the diastereoselective preparation of complex compounds from simpler chiral substrates, including carbohydrates, aminoacids and other natural molecules.

## Figures and Tables

**Table 1:** The Michael addition of methyl phenylacetate (**625**, 1.3 equiv.) to methyl acrylate (**626**) in the presence of the crown ethers bearing two glucose units (6 mol%) and potassium *tert*-butoxide (0.34 equiv.). The reactions were conducted at −78 °C in toluene for 8 min if not specified otherwise.

Entry	Catalyst	Yield (%)	*ee* % (*S*)
1	**642**	59	17
2	**643a**	44	7
3	**643b** ^a^	82	84
4	**643b**	100	80
5	**643b** ^b^	100	76
6	**643c**	69	46
7	**643d**	69	29
8	**643e**	100	76
9	**643f**	75	17
10	**643g**	67	0.4
11	**643h**	31	1.7
12	**643i**	66	0.2

^a^ 1 min, ^b^ 16 min.

**Table 2:** A series of glucose-based aza-crown ethers synthesised by Bakó and co-workers.

Compound	R	Ref.	Compound	R	Ref.
**690a**	H	[284]	**690r**	(CH_2_)_4_P(O)(OEt)_2_	[285]
**690b**	butyl	[286]	**690s**	(CH_2_)_5_P(O)(OEt)_2_	[285]
**690c**	decyl	[287]	**690t**	CH_2_P(O)(Ph)_2_	[288]
**690d**	cyclohexyl	[287]	**690u**	(CH_2_)_2_P(O)(Ph)_2_	[288]
**690e**	allyl	[289]	**690v**	(CH_2_)_3_P(O)(Ph)_2_	[288]
**690f**	(CH_2_)_2_OH	[287]	**690w**	(CH_2_)_4_P(O)(Ph)_2_	[288]
**690g**	(CH_2_)_3_OH	[286]	**690x**	(CH_2_)_5_P(O)(Ph)_2_	[288]
**690h**	(CH_2_)_4_OH	[286]	**690y**	C_6_H_5_	[287]
**690i**	CH_2_CH(OH)CH_2_OH	[289]	**690z**	CH_2_C_6_H_5_	[287]
**690j**	(CH_2_)_2_OCH_3_	[286]	**690aa**	(CH_2_)_2_C_6_H_5_	[286]
**690k**	(CH_2_)_3_OCH_3_	[289]	**690ab**	(CH_2_)_3_C_6_H_5_	[289]
**690l**	(CH_2_)_3_O(CH_2_)_2_OCH_3_	[289]	**690ac**	Ts	[284]
**690m**	(CH_2_)_3_N(CH_3_)_2_	[289]	**690ad**	(CH_2_)_3_NHC(S)NHPh	[289]
**690n**	CH_2_CO_2_CH_3_	[287]	**690ae ^a^**	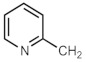	[290]
**690o**	CH_2_P(O)(OEt)_2_	[285]	**690af**	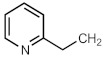	[291]
**690p**	(CH_2_)_2_P(O)(OEt)_2_	[285]	**690ag**	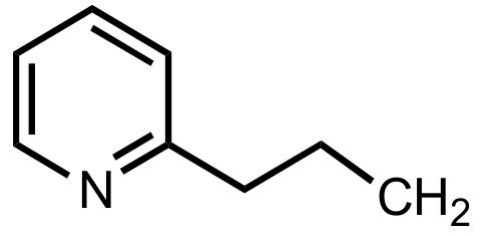	[289]
**690q**	(CH_2_)_3_P(O)(OEt)_2_	[285]	**690ah ^a^**	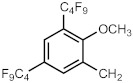	[290]

^a^ aza-crown ethers **690ae** and **690ah** were characterised only by ^1^H NMR analysis.

**Table 3:** A series of β-d-glucoside-based aza-crown ethers synthesised by Bakó and co-workers.

Compound	X	Ref.	Compound	X	Ref.
**702aj**	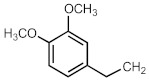	[297]	**703ak**	CH*i*-Pr_2_	[298,299]
**703a**	H	[300] ^a^	**703al**	CH_2_-cyclohexyl	[298,299]
**703b**	butyl	[298,299]	**703am**	hexyl	[299]
**703d**	cyclohexyl	[298] ^a^	**703an**	(CH_2_)_2_CH_3_	[301] ^a^
**703f**	(CH_2_)_2_OH	[298,299]	**703ao**	(CH_2_)_4_OCH_3_	[301] ^a^
**703g**	(CH_2_)_3_OH	[298,299]	**703ap**	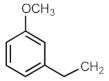	[301]
**703j**	(CH_2_)_2_OCH_3_	[298,299]	**703aq**	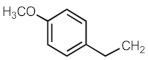	[299]
**703k**	(CH_2_)_3_OCH_3_	[299]	**703ar**	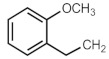	[299]
**703z**	CH_2_C_6_H_5_	[298,299]	**703as**	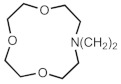	[299]
**703aa**	(CH_2_)_2_C_6_H_5_	[298,299]	**704g**	(CH_2_)_3_OH	[297]
**703af**	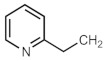	[301] ^a^	**704aj**	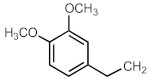	[297]
**703aj**	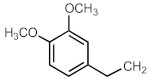	[301]			

^a^ in some cases [298,300,301] the experimental procedures and analytical data were missing.

**Table 4:** A series of d-galactoside-based aza-crown ethers synthesised by Bakó and co-workers.

Compound	X	Ref.	Compound	X	Ref.
**713b**	butyl	[286]	**714g**	(CH_2_)_3_OH	[302]
**713c**	decyl	[287]	**715g**	(CH_2_)_3_OH	[302]
**713g**	(CH_2_)_3_OH	[303]	**716g**	(CH_2_)_3_OH	[302]
**713j**	(CH_2_)_2_OCH_3_	[286]	**717g**	(CH_2_)_3_OH	[302]
**713k**	(CH_2_)_3_OCH_3_	[303]	**718g**	(CH_2_)_3_OH	[302]
**713z**	CH_2_C_6_H_5_	[287]	**718aj**	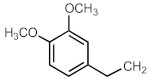	[297]
**713aa**	(CH_2_)_2_C_6_H_5_	[286]	**719g**	(CH_2_)_3_OH	[302]
**713aj**	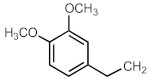	[297]	**720g**	(CH_2_)_3_OH	[302]
**713as**	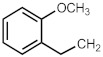	[297]			

**Table 5:** The enantioselective Michael addition shown in Scheme 183.

Entry	Catalyst	Time (h)	Yield (%)	*ee* (%)	Ref.
1	**690f**	20	51	62 (*R*) ^a^	[286,313]
2	**690g**	28	53	85 (*R*) ^a^	[286,293,309]
3	**690j**	40	45	87 (*R*)	[286,310]
4	**690t**	48	39	60 (*R*)	[288]
5	**690u**	48	41	74 (*R*)	[288]
6	**690v**	48	32	77 (*R*)	[288]
7	**690w**	32	43	94 (*R*)	[285,288,310]
8	**722f**	44	32	70 (*S*)	[305]
9	**722g**	52	37	92 (*S*)	[305,309]
10	**722h**	58	40	63(*S*)	[305]
11	**722k**	50	37	77 (*S*)	[305]
12	**713j**	38	34	52 (*R*)	[286]
13	**729a**	24	48	72 (*S*)	[308]
14	**729b**	30	47	76 (*S*)	[308]
15	**729c**	30	51	67 (*S*)	[308]
16	**730**	25	50	80 (*R*)	[308]

^a^ Initially, the absolute configuration was erroneously assigned as (*S*) by NMR analysis on the basis of the chemical shifts in the presence of Eu(hfc)_3_ observed for a crystalline compound whose structure was determined by X-ray diffraction [312]. The positive optical rotation of this compound was first attributed to the (*S*)-enantiomer [286,313] and then, without comments from the authors, to the (*R*)-enantiomer [293,309,310]. Therefore, doubts still exist about the stereochemical assignements given in certain articles.

**Table 6:** The Michael-initiated ring closure reaction shown in Scheme 189.

Entry	Catalyst	Yield (%)	*ee trans* (%)
1	**690g**	89	50
2	**690aj**	94	73
3	**702aj**	91	58
4	**704g**	88	18
5	**704aj**	90	35
6	**713g**	85	62
7	**713aj**	93	80
8	**713ap**	95	76
9	**717g**	93	61
10	**718g**	91	43
11	**718aj**	90	72
